# Development of the European Health Interview Survey - Physical Activity Questionnaire (EHIS-PAQ) to monitor physical activity in the European Union

**DOI:** 10.1186/s13690-015-0110-z

**Published:** 2015-12-02

**Authors:** Jonas D. Finger, Jean Tafforeau, Lydia Gisle, Leila Oja, Thomas Ziese, Juergen Thelen, Gert B. M. Mensink, Cornelia Lange

**Affiliations:** Robert Koch Institute (RKI), Department of Epidemiology and Health Monitoring, General-Pape-Strasse 62-66, D-12101 Berlin, Germany; Scientific Institute of Public Health (IPH), Brussels, Belgium; National Institute for Health Development (NIHD), Tallinn, Estonia

**Keywords:** European health interview survey, Physical activity monitoring, Europe

## Abstract

**Background:**

A domain-specific physical activity questionnaire (EHIS-PAQ) was developed in the framework of the second wave of the European Health Interview Survey (EHIS). This article presents the EHIS-PAQ and describes its development and evaluation processes.

**Methods:**

Research institutes from Belgium, Estonia and Germany participated in the Improvement of the EHIS (ImpEHIS) Grant project issued by Eurostat. The instrument development process comprised a non-systematic literature review and a systematic HIS/HES database search for physical activity survey questions. The developed EHIS-PAQ proposal was reviewed by survey experts. Cognitive testing of the EHIS-PAQ was conducted in Estonia and Germany. The EHIS-PAQ was further tested in a pilot survey in Belgium, Estonia and Germany in different modes of data collection, face-to-face paper and pencil interview (PAPI) and computer assisted telephone interview (CATI).

**Results:**

The EHIS-PAQ is a rather pragmatic tool aiming to evaluate how far the population is physically active in specific public health relevant settings. It assesses work-related, transport-related and leisure-time physical activity in a typical week. Cognitive testing revealed that the EHIS-PAQ worked as intended. The pilot testing showed the feasibility of using the EHIS-PAQ in an international health interview survey setting in Europe. It will be implemented in all 28 European Union Member States via European Union implementing regulation in the period between 2013 and 2015. This will be a first opportunity to get comparable data on domain-specific physical activity in all 28 EU MS and to publish indicators at the EU level.

**Conclusions:**

The EHIS-PAQ is a short, domain-specific PA questionnaire based on PA questions which have been used in large-scale health interview surveys before. It was designed by considering the respondents’ perspective in answering PA questions.

**Electronic supplementary material:**

The online version of this article (doi:10.1186/s13690-015-0110-z) contains supplementary material, which is available to authorized users.

## Background

**Fig. 1:**
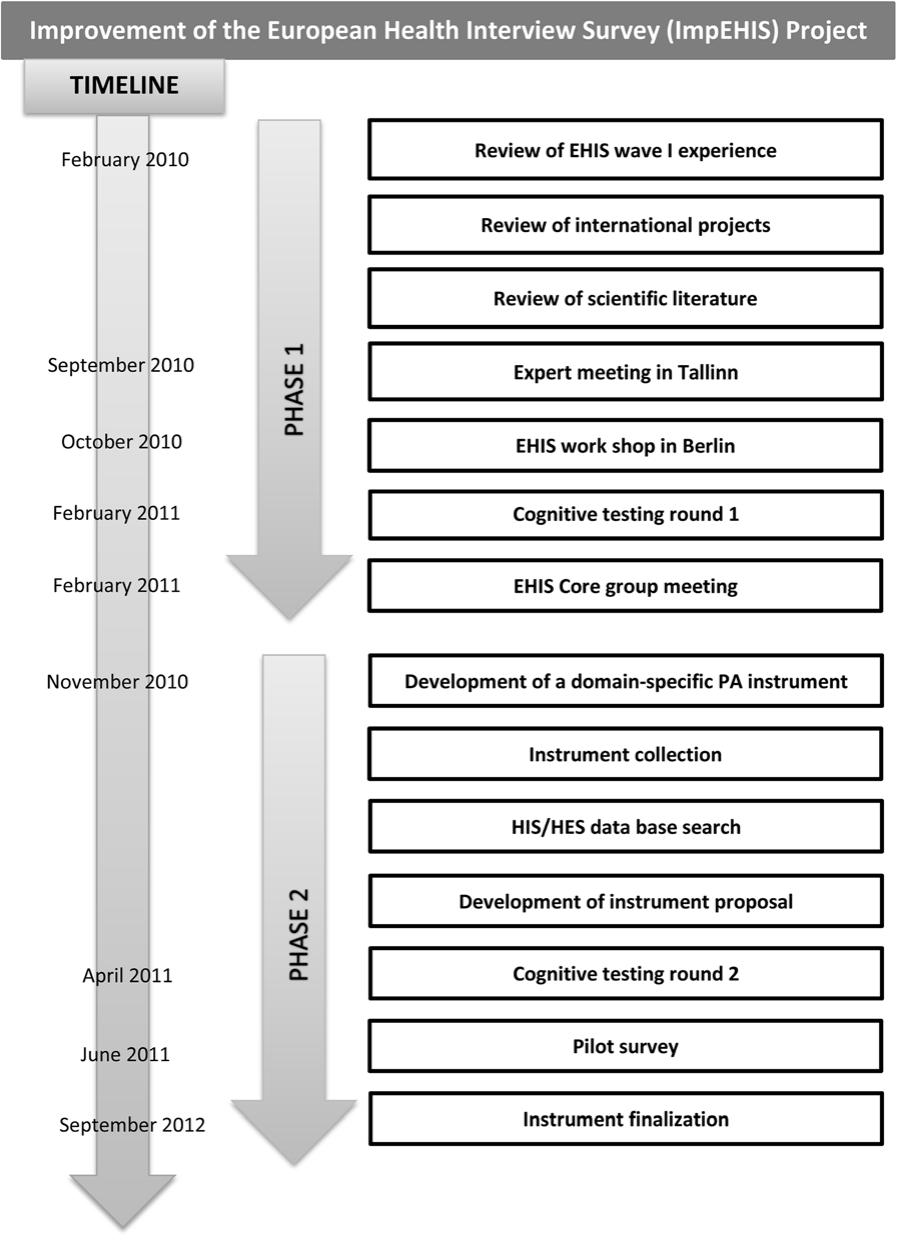
Outline of the Improvement of the European Health Interview Survey (ImpEHIS) project

The European Health Interview Survey (EHIS) is an integral part of the European Commission activities to establish a common framework for the systematic collection and production of European public health statistics. EHIS data are used for calculating indicators for the European Core Health Indicator (ECHI) – shortlist [1, 2]. Physical activity (PA) is included in this ECHI list as one of the major health determinants [3]. In the first EHIS wave carried out in European Union Member States (EU MS) between 2006 and 2010, PA was measured with a modified version of the International Physical Activity Questionnaire – Short Form (IPAQ-SF) [4]. The IPAQ-SF assesses total PA level by collecting information on the number of days and the duration of vigorous-intensity PA, moderate-intensity PA and walking, as well as the duration of sitting on weekdays in the last seven days. The evaluation of the first EHIS wave revealed difficulties in several EU MS with the implementation of the PA instrument used in the survey questionnaire. Eurostat consequently issued a Grant project to revise and improve the PA module and other problematic modules, namely alcohol consumption and mental health, for the second EHIS wave (ImpEHIS project 2010–2011, Grant agreement n° 10501.2099.007‐2009.890). The project started in February 2010 and ended in September 2012. Figure 1 outlines the work packages that were conducted in the EHIS PA module improvement process.

The first phase of the ImpEHIS project aimed to identify the major problems that occurred with the implementation of the EHIS wave 1 instrument. A survey was launched among the EHIS Technical Group members, a group of public health experts who implement the EHIS in their country in which all EU MS are represented, of which 60–70 % (depending on the PA question) indicated that the PA questions should be ‘removed’ , ‘completely changed’ or ‘adapted’ [5].

In response to this result, experts in physical activity assessment were invited to discuss how the EHIS PA module could be improved for the second EHIS wave. One Finish expert had a background in health promotion PA intervention studies, one Estonian expert in military PA studies, one German expert in population health monitoring and one Swedish expert was part of the IPAQ study group. An outcome of this experts’ meeting was that only two validated instruments were effectively available, which had already been used in international large-scale population studies: 1) the IPAQ-SF and 2) the Global Physical Activity Questionnaire (GPAQ) [4, 6]. The GPAQ, however, had rarely been used before in high-income countries and in the EU, and it was considered to be too long for the EHIS; it comprises 16 questions. The IPAQ-SF was seen as the best alternative to obtain valid PA information, and some experts suggested that the modification of the IPAQ-SF in the first EHIS wave might have been responsible for the difficulties that arose. The original IPAQ-SF provides activity examples for each intensity dimension (moderate/vigorous) the respondents are requested to classify their activities in. Those activity examples were removed when the IPAQ-SF was used in the EHIS wave 1. The expert recommendation was thus to use the IPAQ-SF in the next wave again, but this time in its original version [7].

Next, delegates of the EHIS Technical Group, EFTA and EU Candidate Countries, European Commission, Eurostat, OECD and World Health Organization (WHO) were invited to an EHIS workshop and the recommendation of the PA expert group to use the original IPAQ-SF was presented to the delegates. Many of them did not agree with the proposal and it was thus decided that the IPAQ-SF amongst other PA instruments should be tested first, by using the methodology of cognitive interviewing, to find out whether the problems which were reported by the EU MS for the first EHIS wave appear again when using the IPAQ-SF in its original version [5, 7].

As a result, an international cognitive testing study was conducted in different European cultural settings in Belgium, Estonia, Germany and in the UK to test whether the IPAQ-SF perform as intended with different segments of the survey population. The results are described in detail elsewhere [7, 8]. The outcome was that similar difficulties that were reported by the EU MS for the first EHIS wave were observed again. More specifically, respondents reported difficulties to distinguish between the different PA intensity levels, to indicate durations of activities they usually did more or less unconsciously such as walking and sitting, and to combine multiple activities to answer a single question on overall PA. Consequently, the EHIS Core Group, (a group of national population health survey experts set up by Eurostat) decided that a short, domain-specific PA questionnaire should be developed to enable the estimation of three standalone indicators for 1) work-related PA, 2) transport-related (commuting) PA and 3) leisure-time PA.

The purpose of this article is to describe the ‘Phase 2’ of the ImpEHIS project (Fig. 1). More specifically, it aims at presenting the domain-specific EHIS PA questionnaire and describing the development and evaluation process.

## Methods

### Instrument development process

Firstly, published systematic reviews on physical activity questionnaires [9–11] as well as the review of the scientific literature and international projects accomplished by the Estonian study group in the Phase 1 of the project (see Fig. 1) [5] were used to identify available PA questionnaires. A booklet was produced sorting the PA questionnaires according to the instrument types: (a) total PA, (b) occupational PA and (c) leisure-time PA questionnaires. In addition, information from the validation studies for each instrument was collected and included in the booklet.

Secondly, a systematic search for PA survey questions was conducted using the ‘HIS/HES Database’ [12]. The HIS/HES Database presents an inventory of national and multi-country health surveys implemented in EU Member States as well as EFTA countries, EU Candidate Countries and USA, Canada and Australia. The research question was: Which PA questions have been used so far in international HIS/HES surveys? The eligibility criteria were that we searched for (a) ‘all surveys’ including health interview surveys (HIS), health examination survey (HES) and HIS/HES combined, (b) in ‘all regions’ , (c) in ‘national and international surveys’ , and (d) for ‘survey years 1992 until 2008’ because the HIS/HES Database at the time of study covered this period. While searching in the HIS/HES Database, we reviewed all questions in the area of ‘life style factors’ with topic codes ‘411- daily activities’ , ‘412 - physical activity’ , ‘413 - leisure time activities’ and in the area ‘Living and working conditions’ with the topic codes ‘502 - working conditions’ and ‘504 - workplace exposures’. The identified questions were sorted according to the PA question types: work-related PA 1994–2007 (*n* = 56), household PA 1992–2008 (*n* = 28), transport-related PA 1997–2008 (*n* = 30), leisure-time PA 1992–2008 (*n* = 106), sedentary behavior 1995–2008 (*n* = 24), and generic and other PA questions 1992–2008 (*n* = 122). Six booklets were produced (one for each question type) which are presented in the Additional file 1.

The PA questionnaire collection booklets were used to produce a short list of questions for the PA domains being covered by the instrument proposal. The main instrument eligibility criteria were that the questions should be short and easy to understand, suitable for different cultural contexts and various modes of data collection, reliable and valid and they should have already been used before in large-scale population surveys. Two researchers screened all identified PA questionnaires for the eligibility criteria. The amount of questionnaires was reduced and a short list of questions for each PA domain (work-related PA, *n* = 10; transport-related PA, *n* = 6; and leisure-time PA, *n* = 14) was compiled. The short list was used to develop a domain-specific PA instrument proposal. In the development process the experiences from the cognitive testing round 1 (the major problems respondents had when answering the IPAQ-SF) were used to design a user-friendly questionnaire.

### Instrument evaluation process

The instrument evaluation process comprised the following steps: (a) instrument review, (b) cognitive testing and (c) pilot survey.

#### Instrument review

The domain-specific PA instrument proposal was reviewed by the EHIS Core Group and EHIS Technical Group as well as by two physical activity experts (one Estonian expert from the previous expert group and one additional German expert). The EHIS Core Group gave their approval before the instrument was cognitive tested.

#### Cognitive testing

The aim of the cognitive testing study was to examine whether the questions of the domain‐specific PA proposal work as intended when being administered. More specifically, the aim was to collect qualitative information on the questionnaire and its underlying concepts by assessing (a) the respondents’ comprehension of the questions, their understanding and thought processes when answering the questionnaire and (b) their appreciation of the questionnaire in terms of simplicity, sensitivity, adequacy of answer categories and certainty with their answers.

Two study groups from Estonia and Germany agreed upon a standardized data‐collection and analysis strategy, applying the methodology of cognitive testing. Standardized materials and methods were developed for the translation of questionnaires, the cognitive interview probe sheets, materials for the interviewers training and sheets for the data analysis. A common age‐sex roster with the age-group strata 15–19, 20–39, 40–59 and 60+ years was used to select test persons in each country. The total study sample comprised 29 participants, 15 face-to-face interviews were conducted in Estonia and 14 in Germany. The study was approved by the Board of the Federal Commissioner for Data Protection Berlin, Germany. Respondents were informed about the study objectives, the interview process and the applicable data protection guidelines (anonymous data processing and record keeping). Each participant gave informed written consent before enrolling for the study. All interviews were tape-recorded and transcribed. The transcripts were translated into English and entered in the standardized data analysis sheet. Both study groups prepared a country report.

#### Pilot testing

A pilot study was conducted in Belgium, Estonia and Germany to examine the feasibility of administering the EHIS-PAQ in different cultural settings in Europe and in two different modes of data collection, i.e. face-to-face paper and pencil interview (PAPI) and computer assisted telephone interview (CATI). Standard operation procedures (SOPs) were agreed upon in a coordination meeting with all parties. Guidelines were shared for sampling (quota sample), fieldwork practicalities, interviewers’ training and debriefing. The English language source questionnaire was translated into the target languages (French, Estonian, German) using a common translation protocol. Moreover, feedback questions were included at the end of the interview to collect information on the respondents’ perspective with regard to answering the PA questions. In total 167 individuals above 15 years of age participated in the pilot survey, 50 in Belgium (PAPI), 42 in Germany (PAPI), 40 in Germany (CATI) and 35 in Estonia (PAPI). The study was approved by the Board of the Federal Commissioner for Data Protection Berlin, Germany. Respondents were informed about the study objectives, the interview process and the applicable data protection guidelines (anonymous data processing and record keeping). Each participant gave informed written or oral (CATI) consent before enrolling for the study.

## Results and discussion

### The European health interview survey – physical activity questionnaire

**Fig. 2:**
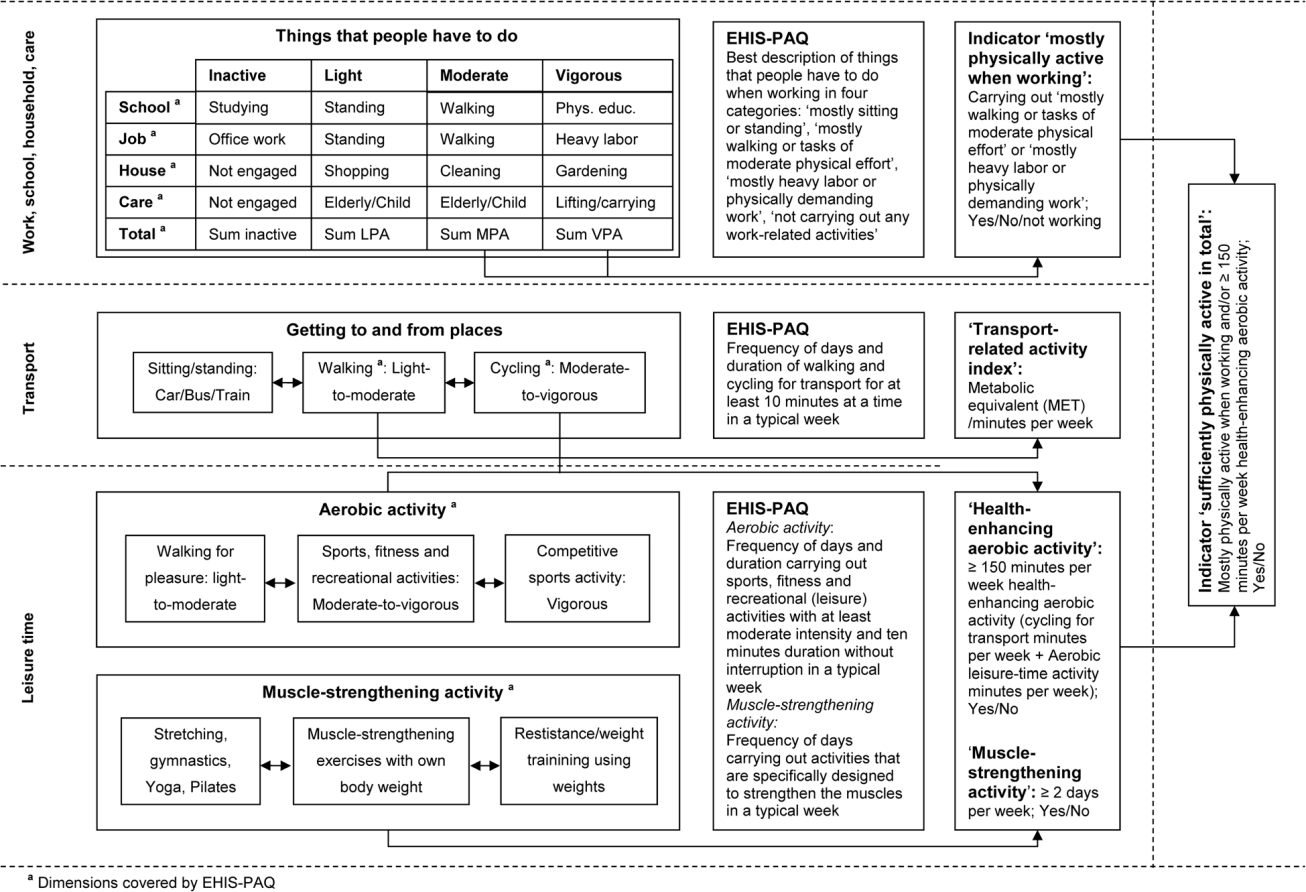
Conceptual model for the instrument development

The conceptual model in Fig. 2 presents the PA settings the European Health Interview Survey-Physical Activity Questionnaire (EHIS-PAQ) covers. Furthermore, it presents the general concepts of the EHIS-PAQ questions and shows which indicators can be calculated from EHIS-PAQ data.

**Fig. 3:**
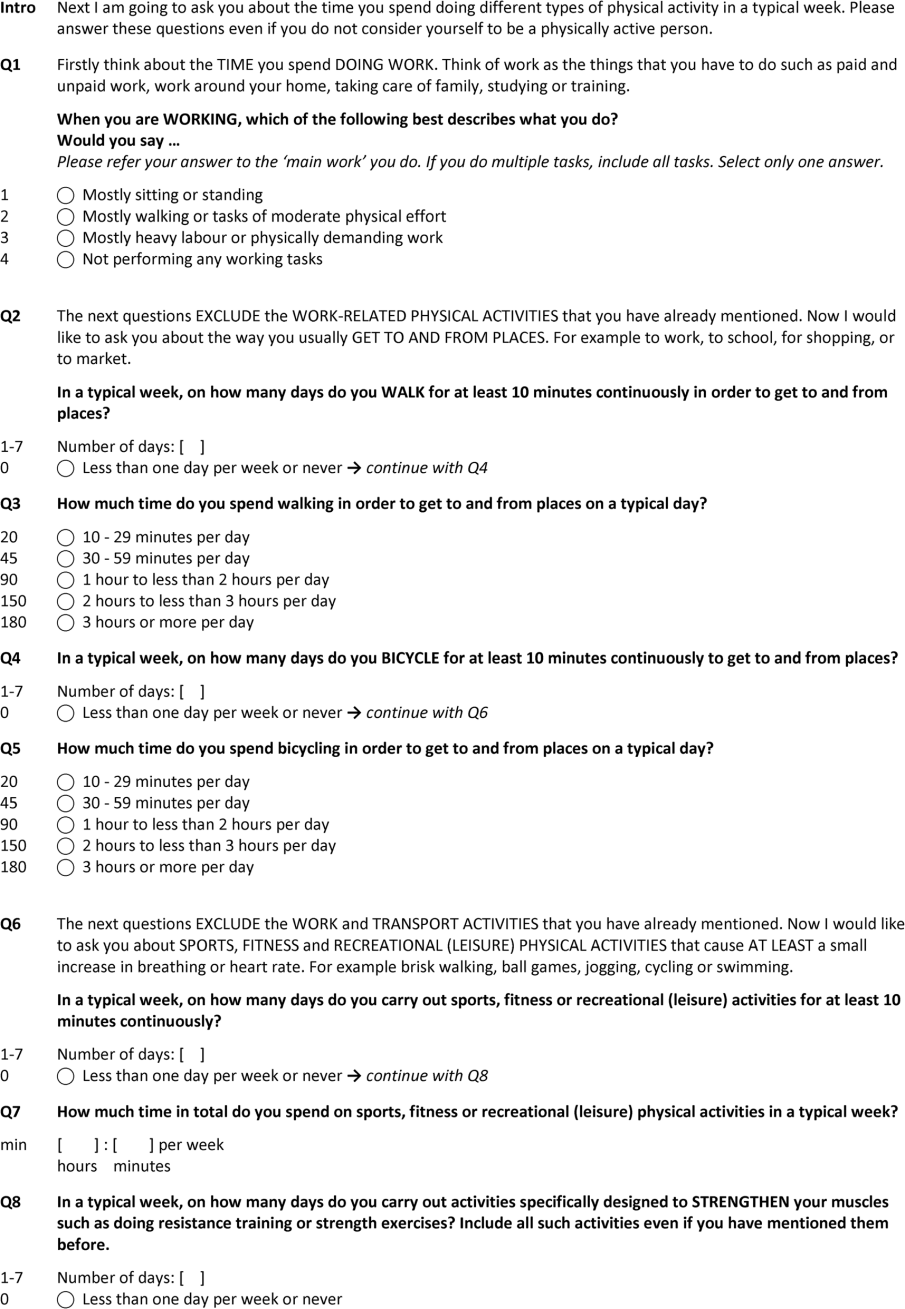
European Health Interview Survey-Physical Activity Questionnaire, EHIS-PAQ (including code book)

The final version of the EHIS-PAQ is illustrated in Fig. 3. Instead of measuring total PA like the IPAQ-SF, where respondents must combine multiple activities of their day course to answer a single question, the EHIS-PAQ is a rather pragmatic tool aiming to evaluate how far the population is physically active in specific public health relevant settings. Many PAQs use setting-specific approaches to categorise activities according to specific domains of PA. This requires less mental effort (calculation and memorizing) for the respondents and activities are easier to remember, as such probing may reduce recall bias [13].

A common setting-specific differentiation is to distinguish occupational and leisure time PA [14]. The EHIS-PAQ guides the respondents through different PA settings using instructions that are similar to those of other domain-specific PA questionnaires (i.e. GPAQ, IPAQ-Long Form) [6, 15]. Respondents are requested to report their work-related PAs first and to exclude them when answering the subsequent questions. Work-related PA is assessed with a question extracted from the Behavior Risk Factor Surveillance Survey (BRFSS-USA) physical activity questionnaire [16]; this question was selected because it has demonstrated high reliability and validity [17–19]. The work-related PA definition used refers in line with the GPAQ to a broad understanding of ‘work’ including all the things that respondents have to do as a part of their daily work activities. ‘Doing work’ includes not only paid and unpaid work, work around the respondents’ home, taking care of family, studying or training, but also seeking a job, doing volunteer work or care for the elderly.

The following section focuses on commuting and active travelling in order to get to and from places. All distances a person travels in order to get to and from places should be considered. Transport-related walking and bicycling is assessed with questions that are similar to those of the GPAQ and IPAQ-Long Form [6, 15]. However, in contrast to the GPAQ, walking and cycling are split in separate questions, with possible answers to choose from several duration categories. Response categories are implemented because respondents indicated in the cognitive testing study that it was difficult for them to report the “exact” duration of daily activities they usually do more or less unconsciously.

The last section focuses on ‘sports, fitness and recreational (leisure) activities’ , the primarily health enhancing type of PA [14]. From an epidemiological perspective it is reasonable to distinguish between aerobic and muscle-strengthening PAs [20]. This distinction also entered into the current PA guidelines for Americans [21] and was adopted by the WHO in 2010 [22]. Sports, fitness and recreational activities are assessed in line with the aerobic PA definition as recommended by WHO or CDC [21, 22]. However, the distinction between moderate- and vigorous-intensity PA is removed and the question refers to ‘at least moderate-intensity’ PA instead. The total sports, fitness and recreational activities’ duration in a typical week is assessed with an open question, since respondents in the cognitive testing study indicated that these activities are usually planned and structured and therefore quite easy to remember. Finally, muscle-strengthening PA is assessed with an adapted version of the muscle-strengthening PA question of the US National Health Interview Survey (NHIS) – Adult Core Questionnaire since 1997 [23], which was tested in the first cognitive testing round (Fig. 1) and performed well in the study. The leisure-time PA domain distinguishes between ‘aerobic’ and ‘muscle-strengthening’ activities.

### Results of the instrument evaluation

#### Cognitive testing

In total, 29 individuals from Estonia (EE) and Germany (DE) participated in the cognitive testing study of the EHIS-PAQ, 14 men and 15 women. Most of the respondents (83 %) perceived the questions as being clear and easy to answer (10/14 in Germany and 14/15 in Estonia).

##### Work-related PA (Q1)

19 respondents indicated that Q1 was easy to answer (9 in DE and 10 in EE), 7 that it was difficult to answer (3 in DE and 4 in EE) and 3 gave no clear answer (2 in DE and 1 in EE). It was difficult to answer for respondents who worked part time in a paid job and also did household chores and took care for their family in the remainder or when the working tasks varied substantially by the level of physical effort.

##### Transport-related PA (Q2-5)

The underlying concept of the questions on ‘walking for travel’ (Q2, 3) was correctly understood by all respondents. Respondents included the following walks ‐ to tram/bus stops or railway station, to the market, to the shops, to friends and from one village to another. All examples mentioned by the respondents met the intended underlying concept of the question. 9 out of 10 respondents who bicycled to get to and from places understood the questions as it was intended. Most of those respondents indicated that they knew the regular routes they bicycled very well, that they know the distances and how long the routes take.

##### Leisure time PA (Q6-8)

23 respondents reported that they engage in sports, fitness or recreational activities (11 in DE and 12 in EE). 19 out of those 23 respondents indicated that they were able to include all physical activities they do in their leisure time (8 in DE and 11 in EE), 3 respondents in DE and one in EE did not provide an answers. 16 out of 23 respondents who reported that they engage in leisure-time PA indicated that it was easy to provide an exact duration for their activities on a weekly basis (Q7), 2 respondents indicated that it was difficult (1 in DE and 1 in EE) and 5 did not provide an answer. Reported reasons for difficulties were ‘uncertainty of what is a typical week’ (in DE) and ‘performing activities on a daily rather than weekly basis’ (in EE). About half of all respondents indicated that they performed muscle-strengthening PA. Generally, muscle-strengthening activities were defined more or less according to the underlying concept of Q8, i.e. working out in the gym, lifting weights, but also yoga, Pilates and aerobics were included.

##### Reference periods (Q3, Q5, Q7)

Most respondents who walked or cycled for travel in DE as well as in EE indicated that they prefer answering the questions on the walking and cycling duration (Q3 and Q5) on a daily basis. The main reason was that transport-related PAs were reported to be daily activities (i.e. commuting to and from work) and it would be easier to report them on a daily basis. Most respondents who reported that they engage in sports, fitness or recreational activities in DE as well as in EE indicated that they prefer to report the duration of their activities on a weekly basis instead of a daily basis. The main reason was that their leisure-time activities are usually planned on a weekly basis and it would hence be easier to report them on a weekly basis.

#### Pilot survey

The pilot survey revealed that the EHIS-PAQ worked in general as intended in both modes of data collection. Common patterns regarding PA correlates, such as age, gender and educational level, were observed that are known from the literature [24]. The results of the pilot survey according to the different PA domains are reported in the Additional file 2. Men had more often physically strenuous jobs than women, and higher educated individuals were more often inactive at work than intermediate and lower educated people (Additional file 2: Table S1). Students showed the highest average bicycling time per week. Higher educated persons showed the highest average bicycling time per week but the lowest average walking time per week compared to intermediate and lower educated persons (Additional file 2: Table S2). Higher educated persons performed more often aerobic leisure time PA than the intermediate and lower educated persons (Additional file 2: Table S3). Higher educated individuals and those with ‘(very) good’ self-rated health met more often the health-enhancing aerobic PA guidelines than intermediate and lower educated persons and those with ‘(very) poor to fair’ self-rated health, respectively (Additional file 2: Table S4).

On the feedback question, ‘Where you able each time to choose an answer category that accurately describes what you do?’ , 152 out of 161 respondents (94 %) indicated that the ‘answer provided (almost) fully correspond to my state’.

### Data analysis guidelines and proposed outcome indicators

Research in the last decades has mainly focused on (health-enhancing) leisure-time PA. Work-related PA was less often in the focus [25]. However, people active at work are less active in their leisure time and vice versa [26–28]. Possibly because physically active workers are less active in leisure time to recover from physical work, whereas office worker may compensate their lack of PA at work with sports and exercise in leisure time. Consequently, focusing on leisure-time PA in population studies seems inadequate, because they mainly tap people with sedentary jobs and higher socioeconomic position. In contrast the PA behavior of physically active workers is not captured adequately [29]. On a country level, if only leisure-time PA is measured, the populations in high income countries with large tertiary sectors may appear more active than those in middle income countries with small tertiary and larger agricultural sectors. Hence, a balanced PA monitoring on an EU level needs to include both work-related PA and leisure-time PA at the same time. Furthermore, transport-related PA, which is modifiable without requesting an extra investment of money and time, has been increasingly in the focus of politicians and health promoters [30]. The conceptual model (Fig. 2) illustrates which indicators can be derived from EHIS-PAQ data; more detailed analyzing guidelines for the proposed PA indicators are presented in Table 1. Many different indicators can be calculated based on the EHIS-PAQ. The indicators presented in the analyzing guidelines are examples which we consider to be important public health-relevant indicators.

**Table 1:** Analyzing guideline for the European Health Interview Survey-Physical Activity Questionnaire, EHIS-PAQ

Indicator	Definition	Calculation	Label	Reporting
Work-related PA	Proportion of individuals doing mostly tasks with at least moderate physical effort when working	Generate WRPA = 1 if Q1 == 2 OR Q1 == 3;	Label WRPA ‘Mostly physically active when working’	Percentage of individuals being mostly physically active when working (WRPA = 1)
		Replace WRPA = 2 if Q1 == 1;	1 = Yes	
		Replace WRPA = 3 if Q1 == 4	2 = No	
			3 = Not working	
Transport-related PA	Quintiles of transport-related physical activity in MET^a^/minutes per week	Enter data for Q3/Q5 as shown in Fig. 3;	Label TRPA ‘Transport-related physical activity in MET/minutes per week’	Upper quintile limits of the transport-related physical activity index
		Generate WalkMin = Q2 x Q3; replace WalkMin = 0 if Q2 == 0		
		Generate CycleMin = Q4 x Q5; CylcleMin = 0 if Q4 == 0		
		GenerateTRPA = WalkMin x 3.3 + CycleMin x 6		
Health-enhancing PA				
Aerobic PA guideline compliance	Proportion of individuals performing at least 150 min aerobic physical activity per week ^b^	Enter data for Q7 in minutes per week;	Label HEPA ‘Aerobic PA at least 150 min per week’^b^	Percentage of individuals performing at least 150 min aerobic physical activity per week^b^ (HEPA = 1)
		Replace Q7 = 0 if Q6 == 0;	1 = Yes	
		Generate AEROB = Q7 + CycleMin;	2 = No	
		Generate HEPA = 1 if AEROB ≥ 150;		
		Replace HEPA = 2 if AEROB < 150^b^		
Muscle- strengthening PA guideline compliance	Proportion of individuals performing at least 2 days per week muscle-strengthening physical activity^c^	Generate MSPA = 1 if Q8 ≥ 2;	Label MSPA ‘Muscle-strengthening physical activity at least 2 days per week’^c^	Percentage of individuals performing at least 2 days per week^c^ muscle-strengthening physical activity (MSPA = 1)
		Replace MSPA = 2 if Q8 < 2^c^	1 = Yes	
			2 = No	
Total PA	Proportion of individuals being sufficiently physically active in total	Generate TPA = 1 if HEPA == 1 OR WRPA == 1;	Label TPA ‘Sufficiently physically active in total’	Percentage of individuals being sufficiently physically active in total (TPA = 1)
		Replace TPA = 2 if HEPA == 2 AND WRPA ≠ 1	1 = Yes	
			2 = No	

^a^MET, metabolic equivalent (one MET corresponds to the energy expenditure at a state of complete rest). The MET values 3.3 for walking and 6 for cycling are adapted from the IPAQ-Long Form data analyzing guidelines [34]

^b^Cut-of-point is for the age group 18+ years, for the age group 15–17 years ≥ 60 min per day should be used

^c^Cut-of-point is for the age group 18+ years, for the age group 15–17 years ≥ 3 days per week should be used

#### Work-related PA

Being sedentary or inactive in general has been identified as a risk factor for ill-health [31, 32]. Thus, it is recommended to construct a work-related PA indicator which compares individuals who ‘mostly sit or stand’ when working with those who perform mostly tasks of ‘at least moderate physical effort’. Two different study populations can be applied when analyzing the data: (a) the workforce population only and (b) the total study population aged 15 years and older.

#### Transport-related PA

At average pace, walking requires about half of the energy expenditure compared to cycling [33]. A way to combine both activities into a single index is to multiply the time spent walking and cycling with respective weighting factors (metabolic equivalent, MET values) collected in the Physical Activity Compendium [33]. In line with the IPAQ-Long Form data processing guideline we propose to use 3.3 METs for walking and 6.6 METs for cycling [34] (see Table 1). These factors correspond to the average energy expenditure of PAs relatively to the energy expenditure at complete rest [33].

#### Health-enhancing PA

PA guidelines have changed several times during the past decades. According to the current health-enhancing PA guidelines of the WHO [22], adults 18 years and older should perform at least 150 min of moderate-intensity aerobic PA and at least 2 sessions of muscle-strengthening PA per week. Adolescents 15–17 years of age should perform at least 60 min of at least moderate-intensity aerobic PA per day and at least 3 times per week muscle-strengthening PA. For constructing an indicator which estimates aerobic PA guideline compliance based on the EHIS-PAQ, the information on transport-related and leisure-time PA can be combined. Whether walking can be counted as moderate-intensity aerobic PA depends upon the walking pace [33]. Walking minutes can be included with the weighting factor 0.5 when calculating the aerobic PA guideline compliance (a more conservative option is to not include walking). In the analysis guidelines, Table 1, we decided not to include walking because a recent study indicates that self-reported moderate PA is more strongly related to objective information (assessed with accelerometer and heart rate monitor) when walking is excluded [35]. The muscle-strengthening PA guideline compliance indicator distinguishes individuals performing muscle-strengthening PA at least twice per week – age group: 18 years and older, and three times per week – age group: 15–17 years, from other respondents.

#### Total PA

Although EHIS-PAQ was explicitly not designed to measure total PA, it is possible to distinguish individuals who are insufficiently physically active regarding both, the health-enhancing aerobic PA and the work-related PA indicators from those who are active according to the work-related and/or the health-enhancing PA indicator (see Table 1). It is also possible to define a total physical activity categorical indicator with four categories: (a) ‘insufficient work-related PA and insufficient health-enhancing aerobic PA’ , (b) ‘sufficient health-enhancing aerobic PA and insufficient work-related PA’ , (c) ‘sufficient work-related PA and insufficient health-enhancing aerobic PA’ , (d) ‘sufficient health-enhancing and work-related PA’.

### Qualifying the EHIS-PAQ in comparison to IPAQ-SF

The major difference of the EHIS-PAQ compared to IPAQ-SF is that it assesses PA in public health-relevant settings (work, transport, leisure). A major limitation of setting-specific approaches is that for some people it can be difficult to define the settings (i.e. leisure time for retired people) [8]. The IPAQ-SF assesses total PA level on a MET (metabolic equivalent) basis and request to sum up all moderate and vigorous activities on a day course. From an European public health monitoring perspective a setting-specific approach is preferable because the health benefits across PA domains are different (i.e. health-enhancing aerobic PA may comply more often with leisure-time PA than work-related PA [36, 37]), health promotion policies and interventions to enhance PA often use settings-specific approaches and information is needed to evaluate and monitor such initiatives on an European level [38–40], it is easier for respondents to recall activities in specific settings than reporting total PA level (less activities have to be recalled and added at once) [13], and total PA expressed in METs is an abstract construct that is difficult to understand for policy makers. Although the EHIS-PAQ is not designed to construct a total PA MET index, MET calculations are possible for the domains transport-related PA (for combining walking and bicycling into a composite index) and for the leisure-time domain. For the work-related PA domain information on working duration is missing, it is possible however to use the information in EHIS on employment status (whether respondents are employed in part-time or full-time) to estimate METs for the work-related PA domain for composing a total PA index for the working population. A recent systematic review on IPAQ-SF validation studies showed that the validity of total PA MET indices in the majority of studies was lower than the acceptable standard [41]; this finding raises concerns of what is the added value of total PA indices on a MET basis.

### The future perspective of EHIS

Although the EHIS-PAQ is adapted from questions which were validated before and which had been used in population health surveys, there is a need to evaluate the validity and reliability of the EHIS-PAQ. Two validation studies have been conducted between 2014 and 2015 in Germany and in Belgium. In Germany the study was conducted by a study group at the University of Regensburg in which the EHIS-PAQ was tested against objective measures (accelerometer, sub-maximal bicycle ergometer test and hand grip test), more comprehensive subjective PA instruments (IPAQ-Long Form and PA dairy) and also the reliability was tested. In Belgium, in the 2014 nutrition survey a validation study was implemented with a comparable methodology used by the study group in Regensburg. The results will be published soon.

While not all EU MS participated in the first EHIS wave with a full survey, the second wave is compulsory for all EU MS since 2013. According to the Commission regulation (EU) on ‘Community statistics on public health and health and safety at work’ (No 1338/2008), all EU MS are thus legally committed to carry out the second EHIS wave between 2013 and 2015. The EHIS variables (including the EHIS-PAQ) are stipulated in the EHIS Commission regulation (EU) No 141/2013. The methodological manual for the second EHIS wave, containing an example questionnaire, conceptual guidelines and interviewer instructions, is available at the Eurostat website [42]. This will be a first opportunity to get comparable data on domain-specific physical activity in all 28 EU MS and to publish indicators at the EU level. The data collection for the EHIS wave 2 will be completed in all countries in 2016. The EHIS wave 2 data are expected to become available for the scientific community at the end of 2017 or beginning of 2018.

## Conclusions

The PA module used in the first EHIS wave did not work as intended in many EU MS and needed revision. The revised instrument (EHIS-PAQ) is based on PA questions which have been used in large-scale health interview surveys before. It was designed considering the respondents’ perspective how to best answer PA questions. The EHIS-PAQ is a domain-specific PA questionnaire, however shorter than the GPAQ and the IPAQ-Long Form and was specifically designed for a multinational health interview survey context. The EHIS-PAQ was tested in different regions and cultural settings in Europe. It follows partly the concept of the GPAQ which was developed mainly for the use in a low and middle income country setting. For this reason it is expected that the EHIS-PAQ can be used in all regions of Europe. Three PA domains are covered by the EHIS-PAQ, more specifically work-related PA, transport-related PA and leisure-time PA. The EHIS-PAQ distinguishes between ‘aerobic’ and ‘muscle-strengthening’ PA and allows to estimate the health-enhancing PA recommendation compliance. The EHIS-PAQ will be implemented in the EHIS wave 2 between 2013 and 2015 in all EU MS.

## Additional files

Additional file 1:
**Contains the booklets of HIS/HES Database physical activity questions.** (PDF 1889 kb) Additional file 2: Contains the descriptive results of the pilot survey for the work-related PA outcome (**Table S1**), the transport-related PA outcomes (**Table S2**), the leisure - time PA outcomes (**Table S3**) and the health-enhancing PA outcomes (**Table S4**). Adobe Acrobat Reader is required to access those files. (PDF 238 kb)
