# Supplementary material for: Development of the European Health Interview Survey - Physical Activity Questionnaire (EHIS-PAQ) to monitor physical activity in the European Union
Source: Arch Public Health. 2015 Dec 2;73:59. doi: 10.1186/s13690-015-0110-z (PMC4667448; doi:10.1186/s13690-015-0110-z)
Supplement: Additional file 1: — Contains the booklets of HIS/HES Database physical activity questions. (PDF 1889 kb) [file 13690_2015_110_MOESM1_ESM.pdf]

Supplemental File 1

The following survey item booklets contain information on survey items in the field of physical activity taken from the **European Health Interview & Health Examination Surveys (HIS/HES) Database. Please note the following copyright and regulations of the HIS/HES Database:**

"**Dissemination and or use of HIS/HES database information and copyright protection**

In respect of the laws of intellectual property and its related copyright protection, a strong effort has been made by the database developers to include adequate information on this subject for each survey.

Usually a health survey can contain several (parts of) standardized instruments of which the original version is protected by copyrights. Users of the database have the possibility to verify for each and every survey if any copyright rules are applicable.

You are therefore kindly requested to verify under "HIS or HES survey methodology" subtitle "Instruments" and field 'submitted to copyrights' before possibly using one of the instruments mentioned in the database.

Each institute responsible for a particular survey has been requested to answer the question of submission to copyrights. There are three answer possibilities incorporated into the database: 'Yes, followed by the specification of the instrument', 'No' and 'Unknown'. In case the answer is 'unknown' the user is advised to contact the responsible institute in order to obtain further information prior to copying any details.

Neither the Scientific Institute of Public Health (IPH) Brussels nor the National Institute of Public Health, Helsinki - responsible for the development of this European Health Surveys Database - can be held responsible for breach of copyright regulations by the users of this database".

Source: Scientific Institute of Public Health, Brussels and National Institute of Public Health, Helsinki: **European Health Interview & Health Examination Surveys Database. Available from:** https://hishes.wiv-isp.be/index.php?hishes=copyright **(accessed: 4 November 2015)**. Brussels: Scientific Institute of Public Health; 2015.

International Survey Items concerning

Work-related Physical Activity

1994 - 2007

TABLE OF CONTENTS

1994
Denmark – DK 01 3

1995
Spain – ES01 4
United Kingdom – UK 10 5

1996
Poland – PL 04 6

1997
Finland – FI 02 7

1998
Germany – DE 05 8
Ireland – IE 01 9
United Kingdom – UK 02 10
United Kingdom – UK 09 11
United Kingdom – UK 13 12

1999
Spain – ES 04 13
France – FR 06 14
Italy – IT 01 16

2000
Canada – CA 01 17
Cyprus – CY 02 18
Finland – FI 01 19
International – EUPASS 20

2001
Spain – ES 02 21
Netherlands – NL 02 22
Netherlands – NL 03 23

2002
Switzerland – CH 02 24
Czech Republic – CZ 01 25
Estonia – EE 04 26
Finland – FI 07 27
Lithuania – LT 02 28
Latvia – LV 03 29
Malta – MT 01 30
Netherlands – NL 05 31
United Kingdom – UK 28 32

2003
Canada – CA 02 33
Spain – ES 03 34
Finland – FI 10 35
Croatia – HR 04 36
International – INT 12 37
Netherlands – NL 08 38

2004
Estonia – EE 07 39
Finland – FI 12 40
International – FINBALT 41
Netherlands – NL 07 42
Poland – PL 06 43

2005

Canada – CA 03	44
Finland – FI 19	45
Italy – IT 08	46

2006

Serbia – CS 01	47
Spain – ES 06	48
France – FR 18	49
Lithuania – LT 05	50
United States – US 04	51

2007

Australia – AU 04	52
Canada – CA 04	53
Canada – CA 05	54
Switzerland – CH 03	55
Finland – FI 21	56
Finland – FI 22	57
Ireland – IE 09	58
Iceland – IS 04	59

Country:	Denmark
Survey:	Danish Health and Morbidity Survey
Survey Code:	DK 01
Year:	1994
Type:	Face to Face

<u>Question 73</u>

How would you describe the physical strain of your chief occupation?

Show card 13

mainly sedentary work which doesn't demand any physical effort
work which to a great extent is performed standing or walking but apart from that doesn't demand any physical effort
standing or walking work with many lifts or much carrying
heavy or speedy work which is strenuous
no information
don't know
not actively employed

Country:	Spain
Survey:	National Health Survey
Survey Code:	ES 01
Year:	1995
Type:	Face to Face

Question 28

We should now like you to tell us what type of physical exercise your work or usual activity involves.
Of the possibilities on this card, which would you say best describes your main activity (at work, educational establishment, doing the housework, etc.)?

(SHOW CARD I)

Seated most of the day
Standing most of the day, not moving about much, not much physical effort
Walking, carrying a weight, frequently moving about
Heavy work, tasks requiring a great deal of physical effort
No answer

Country: United Kingdom

Survey: The Scottish Health Survey

Survey Code: UK 10

Year: 1995

Type: Face to Face + self administered

Question 351

When you're at work are you mainly sitting down,
standing up or walking about?

Sitting down
Standing up
Walking about
Equal time spent doing 2 or more of these

Question 352

Does your work involve you moving between floors?

Yes
No

Question 354

Do you do any (other) climbing in the course of your
work (ladders, scaffolding etc.)?

Yes
No

Question 355

Do you usually have to lift or carry things at work which
you find heavy?
If yes, Is that just lifting or lifting and carrying?

Lift heavy loads
Lift and carry heavy loads
No

Country:	Poland
Survey:	Health Population Status in Poland in 1996
Survey Code:	PL 04
Year:	1996
Type:	Face to Face

Questions 45 - 46

Does the child participate in lessons of physical education?

No, he/she is released
Yes

If so how many hours per week?

...

Country:	Finland
Survey:	FINRISK 97
Survey Code:	FI 02
Year:	1997
Type:	Face to Face + self administered

Question 67

How demanding is your work physically? The activity at work is divided into four groups.

My work is mainly done sitting down and I do not walk much during my working hours (f. ex. a clock smith, radio mechanic and industrial seamstress, office work at a desk).

I walk quite much in my work, but I do not have to lift or carry heavy objects (f. ex. a foreman and store assistant, light industrial worker, office work which requires walking).

I have to walk and lift much or to take the stairs or go uphill (f. ex. a carpenter or cattle minder/dairy work, engineering shop or other heavier industrial work).

My work is heavy manual labour in which I have to lift or carry heavy objects, to dig, shovel or chop (f. ex. forestry, heavy farm work, heavy construction or industrial work).

Country: Germany

Survey: German National Health Examination and Interview Survey

Survey Code: DE 05

Year: 1998

Type: ???

Question 89

Is your present occupation characterised by:

No
Yes
If yes, Do you feel that your health is impaired as a result?
No
Yes

Strenuous physical activity (such as one sided posture, carrying heavy objects...)
noise, dust, gases, vapours, "bad air"
stress at work (such as pressure of time or pressure to do well, hard concentration, bad working atmosphere), job worries
overtime, long working hours
shift / night work; If so:
Changing shifts excl. Night shifts
Changing shifts incl. Night shifts
Night shifts only (not changing shifts)

Question 89A

How long have you had this job?

... years ... Months

Country:	Ireland
Survey:	Survey of Lifestyle, Attitudes and Nutrition (SLÁN)
Survey Code:	IE 01
Year:	1998
Type:	Self administered

Question B6

Thinking about your job in general would you say that you are

Very physically active
Fairly physically active
Not very physically active
Not at all physically active

Country:	United Kingdom
Survey:	Health Education Monitoring Survey
Survey Code:	UK 02
Year:	1998
Type:	Face to Face

Question 102

Thinking about your job in general, (ASK ABOUT MAIN JOB ONLY) would you say that you are

very physically active
fairly physically active
not very physically active
or not at all physically active in your job?

Country:	United Kingdom
Survey:	Health Survey for England
Survey Code:	UK 09
Year:	1998
Type:	Face to Face + self administered

Question 162

Thinking about your job in general would you say that
you are

very physically active,
fairly physically active,
not very physically active,
or, not at all physically active in your job?

Question 163

When you're at work are you mainly sitting down,
standing up or walking about?

Sitting down
Standing up
Walking about

Question 164

Do you do any climbing in the course of your work
(ladders, scaffolding etc)?

Yes
No

Question 165

Do you usually have to lift or carry things at work which
you find heavy?
Is that just lifting or lifting and carrying?

Yes - lift heavy loads
Yes - lift and carry heavy loads
No

Country:	United Kingdom
Survey:	The Scottish health survey
Survey Code:	UK 13
Year:	1998
Type:	Face to Face

Question 177

Thinking about your job in general would you say that you are

… very physically active,
… fairly physically active,
… not very physically active,
… or, not at all physically active in your job?

Country:	Spain
Survey:	Impairments, Disabilities and Health Status Survey
Survey Code:	ES 04
Year:	1999
Type:	Face to Face

Question 11.25

Specify how you perform or what type of physical exercise is involved in your job or main occupation, is, what better describes your main occupation at your place of work, teaching institution, home, etc.

Sitting down for most of the day
Standing for most of the day, with little movement or exertion
Walking, carrying loads, moving about frequently
Hard work, jobs that call for considerable physical Exertion

Country: France

Survey: Health Barometer

Survey Code: FR 06

Year: 1999

Type: Telephone

Question 170

During the LAST SEVEN DAYS, how many hours...
did you spend at SCHOOL, GRAMMAR SCHOOL, UNIVERSITY ?

… HOURS
… MINUTES
NONE)
DON'T KNOW

Athletics (apart from jogging)
Rowing
Badminton
Basket ball
Canoeing-kayaking
Cycling
Dancing
Horse riding
Rock climbing
Football
Golf
Gymnastics
Handball
Jogging
Judo
Karate
Walking (leisure) - Hiking
Body-building
Swimming
Ice skating
Pétanque (bowls)
Windsurfing
Diving
Qi Gong
Roller blading/Roller skating
Rugby
Skiing
Surfing
Tai chi chuan
Tennis
Table tennis (ping-pong)
Archery
Volleyball
Mountain biking
Yoga

Other 1, specify: …
Other 2, specify: …
Other 3, specify: …
Other 4, specify: …
Other 5, specify: …

Question 173

During the LAST SEVEN DAYS, how many hours…
did you spend AT WORK OR DOING THINGS AS PART OF YOUR EMPLOYMENT ?

… HOURS
… MINUTES
NONE
DON'T KNOW

Athletics (apart from jogging)
Rowing
Badminton
Basket ball
Canoeing-kayaking
Cycling
Dancing
Horse riding
Rock climbing
Football
Golf
Gymnastics
Handball
Jogging
Judo
Karate
Walking (leisure) - Hiking
Body-building
Swimming
Ice skating
Pétanque (bowls)
Windsurfing
Diving
Qi Gong
Roller blading/Roller skating
Rugby
Skiing
Surfing
Tai chi chuan
Tennis
Table tennis (ping-pong)
Archery
Volleyball
Mountain biking
Yoga
Other 1, specify: …
Other 2, specify: …
Other 3, specify: …
Other 4, specify: …
Other 5, specify: …

Country:	Italy
Survey:	Health Conditions and the Use of Health Services
Survey Code:	IT 01
Year:	1999
Type:	Face to Face

Question 15.9

Can your work activity mainly be considered a physical activity?

Light, I spend most of the time sitting down
Moderate, I spend most of the time standing up
Heavy, till perspiring at least half of the working hours

Country:	Canada
Survey:	Canadian Community Health Survey Cycle 1.1
Survey Code:	CA 01
Year:	2000
Type:	Face to Face

<u>Question PA4A</u>

Next, some questions about the amount of time you spent in the past 3 months on physical activity at work or while doing daily chores around the house, but not leisure time activity.

In a typical week in the past 3 months, how many hours did you usually spend walking to work or to school or while doing errands?

None
Less than 1 hour
From 1 to 5 hours
From 6 to 10 hours
From 11 to 20 hours
More than 20 hours

Country:	Cyprus
Survey:	Population Risk Factors Survey
Survey Code:	CY 02
Year:	2000
Type:	Face to Face

Question 32

How much physical activity do you have at work?
(If you do not work, mark 1)

My work is mainly sitting work. I do not walk much at work.
(Examples: office work - radio mechanic, tailor)

I walk in my work quite a lot but I do not have to lift or carry heavy things.
(Examples: shop assistant, light industrial work, office work where one has to move, housewife, nurse))

I must walk and carry a lot or often climb stairs or go uphill in my work.
(Examples: carpenters or farmhand, work in engine shop, heavy industrial work)

My work is heavy physical work, where I have to carry or lift heavy things, to shovel or cut a lot.
(I am in construction and industrial work)

Country:	Finland
Survey:	Survey on health behaviour
Survey Code:	FI 01
Year:	2000
Type:	Self administered

<u>Question 90</u>

How physically strenuous is your work? Choose the alternative that best describes your situation. If you do not work, mark 'my work is mainly sedentary and I don't walk much'

- my work is mainly sedentary and I don't walk much
- I walk quite a lot in my work, but do not need to lift or carry heavy objects
- in my work I need to walk a great deal and often need to walk upstairs or uphill
- my work is heavy physical work where I need to lift or carry heavy objects, dig, shovel or chop, etc.

Country: International

Survey: European Physical Activity Surveillance System (EUPASS)

Survey Code:

Year: 2000

Type: Telephone

Question B5a

How much physical activity did you do in your place of work in the last 7 days?

Much
Moderate
Little or no

Country:	Spain
Survey:	National Health Survey
Survey Code:	ES 02
Year:	2001
Type:	Face to Face

Question P39

Please tell us about the kind of physical fitness your work or regular activities demand. Choose from the list on the card. Which option best describes your daily routine? (work, studies, household work, etc.)

(SHOW CARD K)

Sat down most of the day
Stood up most of the day without much movement or effort to make
Walking. Bearing weight, moving frequently
Heavy work, intensive physical force required
Don't know

Country: Netherlands

Survey: Patient survey - Second Dutch National Survey of General Practice

Survey Code: NL 02

Year: 2001

Type: Face to Face

Question LRV44t2

We will continue with physical activities at work and school.

How much time a week do you spend on light to moderately strenuous work? (sitting/standing work, walking from time to time, such as office work)?

Number of hours: ...

Question LRV44t3

Number of minutes: …

Question LRV44u2

And how much time per week do you spend on very strenuous work (on your feet, walking around, regularly lifting heavy objects)?

Number of hours: ...

Question LRV44u3

Number of minutes: …

Country: Netherlands

Survey: Continuous Quality of Life Survey

Survey Code: NL 03

Year: 2001

Type: Self administered

Question 366

Physical activity at work or in school

light and moderately strenuous work (sitting/standing work, with some walking, like desk work or walking with light loads)

heavy work (running work, or work whereby heavy loads have to be carried or lifted)

not applicable

Number of hours per week

… hours

Country:	Switzerland
Survey:	Swiss Health Survey 2002
Survey Code:	CH 02
Year:	2002
Type:	Telephone

Question 44.02

And in your work, whether a professional activity,
household chores or gardening, how many
days a week do you perspire (on average)?

… days a week (between 0 and 7 days)
no reply

Country:	Czech Republic
Survey:	Sample Survey of the Health Status of the Czech Population
Survey Code:	CZ 01
Year:	2002
Type:	Face to Face

Question 24b

Physical activity in your work:
Circle one type of employment according to physical burden:

hard, physically difficult work
easy, physically less difficult work
sedentary
physically not difficult work
unemployed

Country:	Estonia
Survey:	Health Behaviour Among Estonian Adult Population
Survey Code:	EE 04
Year:	2002
Type:	Face to Face

Question 67

How physically strenuous is your work?

Very light (mainly sitting)
Light (mainly walking)
Medium (lifting, carrying light loads)
Heavy manual work (climbing, carrying heavy loads)

Country: Finland

Survey: FINRISK 2002

Survey Code: FI 07

Year: 2002

Type: Self administered

Question 73

How demanding is your work physically?
The activity at work is divided into four groups. If you do not work mark 1.

My work is mainly done sitting down and I do not walk much during my working hours (e.g. a clocksmith, radio mechanic and industrial seamstress, office work at a desk).

I walk quite much in my work, but I do not have to lift or carry heavy objects (e.g. a foreman and store assistant, light industrial worker, office work which requires walking).

I have to walk and lift much or to take the stairs or go uphill (e.g. a carpenter or cattle minder/dairy work, engineering shop or other heavier industrial work).

My work is heavy manual labor in which I have to lift or carry heavy objects, to dig, shovel or chop (e.g. forestry, heavy farm work, heavy construction or industrial work).

Country: Lithuania

Survey: Lithuanian Health Behaviour Monitoring

Survey Code: LT 02

Year: 2002

Type: Self administered

Question 59

How physically strenuous is your work?

very light (mainly sitting)
light (mainly walking)
medium (lifting, carrying light loads)
heavy manual work (climbing, carrying heavy loads)

Country: Latvia

Survey: Health Behaviour Survey of Latvian Adult Population

Survey Code: LV 03

Year: 2002

Type: Self administered

Question 80

Does your work require much physical activity?

Very light (mostly sitting)
Light (sitting and standing work, which requires some walking)
Medium (work which requires much walking, lifting and relocation of heavy objects)
Heavy manual labour (forestry, heavy agriculture, heavy building work, road-works, etc.)

Country: Malta

Survey: First National Health Interview Survey

Survey Code: MT 01

Year: 2002

Type: Face to Face

Question 66

PHYSICAL ACTIVITY AT WORK

Thinking about your job in general, would you say that during work-time you are…

I am not gainfully occupied
Very physically active
Fairly physically active
Not very physically active
Not at all physically active

Country:	Netherlands
Survey:	Continuous Quality of Life Survey
Survey Code:	NL 05
Year:	2002
Type:	Self administered

Question 366

Physical activity at work or in school

light and moderately strenuous work (sitting/standing work, with some walking, like desk work or walking with light loads)

heavy work (running work, or work whereby heavy loads have to be carried or lifted)

not applicable

Number of hours per week

… hours

Country: United Kingdom

Survey: Health Survey for England

Survey Code: UK 28

Year: 2002

Type: Face to Face

Question 145

Can I just check, is (name of child) at school in reception class yet?

Yes
No

Question 166

Thinking about your job in general would you say that you are

...READ OUT…

...very physically active,
...fairly physically active,
...not very physically active,
...or, not at all physically active in your job?

Country:	Canada
Survey:	Canadian Community Health Survey Cycle 2.1
Survey Code:	CA 02
Year:	2003
Type:	Face to Face + Telephone

Question WST_Q408

Your job required a lot of physical effort.

Strongly agree
Agree
Neither agree nor disagree
Disagree
Strongly disagree

Country:	Spain
Survey:	National Health Survey 2003
Survey Code:	ES 03
Year:	2003
Type:	Face to Face

Question 31

Which of the following possibilities best describes your main activity at work, school, home (domestic work), etc.?

Seated the majority of the working day

Standing up most of the working day without carrying out large journeys or efforts

Walking, carrying some weight, frequent journeys which do not require an important physical effort

Carrying out tasks which require an important physical effort

Country: Finland

Survey: Health Behaviour among the Finnish Adult Population

Survey Code: FI 10

Year: 2003

Type: Face to Face

Question 85

How physically strenuous is your work?
Choose the alternative that best describes your situation. If you do not work, mark 1

my work is mainly sedentary and I don't walk much.
my work is mainly sedentary and I don't walk much
I walk quite a lot in my work, but do not need to lift or carry heavy objects
in my work I need to walk a great deal and often need to walk upstairs or uphill
my work is heavy physical work where I need to lift or carry heavy objects, dig, shovel, or chop, etc.

Country: Croatia

Survey: Croatian Health Survey

Survey Code: HR 04

Year: 2003

Type: Face to Face

Question PHA 02

How physically strenuous is your work?

Very light (mainly sitting)
Light (mainly walking)
Moderate (lifting, carrying light loads)
Hard physical work (climbing, carrying heavy loads)

Country: International

Survey: Joint Canada/United States Survey of Health (JCUSH)

Survey Code: INT 12

Year: 2003

Type: Telephone

Question PAC_Q04A

Next, some questions about the amount of time you spent in the past 3 months on physical activity at work or while doing daily chores around the house, but not leisure time activity.

In a typical week in the past 3 months, how many hours did you usually spend walking to work or to school or while doing errands?

None
Less than 1 hour
From 1 to 5 hours
From 6 to 10 hours
From 11 to 20 hours
More than 20 hours

Country:	Netherlands
Survey:	Continuous Quality of Life Survey
Survey Code:	NL 08
Year:	2003
Type:	Self administered

Question 366

Physical activity at work or in school

light and moderately strenuous work (sitting/standing work, with some walking, like desk work or walking with light loads)

heavy work (running work, or work whereby heavy loads have to be carried or lifted)

not applicable

Number of hours per week

… hours

Country: Estonia

Survey: Health behaviour among Estonian adult population

Survey Code: EE 07

Year: 2004

Type: Self administered

Question 74

At your work place at the moment, are the possibilities to engage in physical exercise under favourable conditions (partial return of monthly fees in sport clubs, paying rent to sport facilities etc)?

yes
no
I do not work

Question 75

How physically strenuous is your work?

very light (mainly sitting)
light (mainly walking)
medium (lifting, carrying light loads)
heavy (climbing, carrying heavy loads)

Country: Finland

Survey: Health Behaviour among the Finnish Adult Population

Survey Code: FI 12

Year: 2004

Type: Face to Face

Question 75

How physically strenuous is your work?
Choose the alternative that best describes your situation. If you do not work, mark ‘my work is mainly sedentary and I don’t walk much’.

my work is mainly sedentary and I don’t walk much

I walk quite a lot in my work, but do not need to lift or carry heavy objects

in my work I need to walk a great deal and often need to walk upstairs or uphill

my work is heavy physical work where I need to lift or carry heavy objects, dig, shovel, or chop, etc.

Country: International (Finland, Baltic States)

Survey: FINBALT Health Monitor

Survey Code:

Year: 2004

Type: Self administered

Question 57

How physically strenuous is your work?

1 very light (mainly sitting)
2 light (mainly walking)
3 medium (lifting, carrying light loads)
4 heavy manual work (climbing, carrying heavy loads)

Country:	Netherlands
Survey:	Continuous Quality of Life Survey
Survey Code:	NL 07
Year:	2004
Type:	Self administered

Question 343

Physical activity at work or in school

light and moderately strenuous work (sitting/standing work, with some walking, like desk work or walking with light loads)

heavy work (running work, or work whereby heavy loads have to be carried or lifted)

not applicable

Number of hours per week

… hours

Country:	Poland
Survey:	Health Population Status
Survey Code:	PL 06
Year:	2004
Type:	Face to Face

Question 54

Does the child participate in physical education (gym) in school?

Yes
No, he/she

Country:	Canada
Survey:	National Population Health Survey (NPHS)
Survey Code:	CA 03
Year:	2005
Type:	Face to Face + Telephone

Question PA_Q4A

Next, some questions about the amount of time you spent in the past 3 months on physical activity at work or while doing daily chores around the house, but not leisure time activity.

In a typical week in the past 3 months, how many hours did you usually spend walking to work or to school or while doing errands?

None
Less than 1 hour
From 1 to 5 hours
From 6 to 10 hours
From 11 to 20 hours
More than 20 hours

Country: Finland

Survey: Health Behaviour among the Finnish Adult Population

Survey Code: FI 19

Year: 2005

Type: Self administered

Question 80

How physically strenuous is your work? Choose the alternative that best describes your situation. If you do not work, mark ‘my work is mainly sedentary and I don’t walk much’.

my work is mainly sedentary and I don’t walk much

I walk quite a lot in my work, but do not need to lift or carry heavy objects

in my work I need to walk a great deal and often need to walk upstairs or uphill

my work is heavy physical work where I need to lift or carry heavy objects, dig, shovel or chop, etc.

Country: Italy

Survey: Health Conditions and the Use of Health Services 2004-2005

Survey Code: IT 08

Year: 2005

Type: Face to Face

Question 2.9

(For those who are working)

How much time on average each week do you devote to work (for the occasional workers please refer to an average working week):

Less then one hour
N° of hours ... per week

Question 2.10

Your work activity is characterised by a physical activity that is mainly?

Light ,I spend most of the time sitting
Moderate, I spend most of the time standing or walking
Heavy, till perspiring

Country:	Serbia
Survey:	National Health Survey, Serbia 2006
Survey Code:	CS 01
Year:	2006
Type:	Face to Face + self administered

Question SV4

How physically strenuous is the job that you do?
Answers are given only by employed or self employed (farmers included)

Mainly sitting
Mainly standing/walking, but not carrying heavy loads
Walking a lot, climbing up the stairs and/or lifting loads
Carrying/lifting heavy loads

Question SV4

Do you regularly attend physical education classes at school?

I avoid whenever I can
I am excused
Yes, on a regular basis
There are no conditions

Country: Spain

Survey: National Health Survey

Survey Code: ES 06

Year: 2006

Type: Face to Face

Question 90

Which of the following possibilities best describes your main activity at your place of work, education centre, home (household chores), etc?

- Seated most of the day
- Standing up most of the day, without moving around a lot or making a great deal of effort
- Walking, carrying a bit of weight, moving around frequently
- Doing tasks requiring a great deal of physical effort

Country: France

Survey: National survey on nutrition and health (ENNS)

Survey Code: FR 18

Year: 2006

Type: Face to Face

Question E59

During the past week, did your child have a PE or other sports lessons at school?

Yes,
No, he/she was excused
No, no lesson was scheduled
Doesn't know
Refuses to answer

Country:	Lithuania
Survey:	Lithuanian health behaviour monitoring
Survey Code:	LT 05
Year:	2006
Type:	Self administered

<u>Question 75</u>

How physically strenuous is your work?

very light (mainly sitting)
light (mainly walking)
medium (lifting, carrying light loads)
heavy manual work (climbing, carrying heavy loads)

Country:	United Kingdom
Survey:	Health Survey for England
Survey Code:	UK 33
Year:	2006
Type:	Face to Face

Question Work

I'd like to ask you about some of the things you have done in the past four weeks that involve physical activity, this could be at work/college or in your free time. (Can I just check) were you in paid employment or self-employed in the past four weeks?

Yes
No

Question Active

Thinking about your job in general would you say that you are ...

... very physically active,
... fairly physically active,
... not very physically active,
... or, not at all physically active in your job?

Country: Australia

Survey: National Health Survey

Survey Code: AU 04

Year: 2007

Type: Face to Face

Question EXER_Q24

When [you are/(proxy name) is] at work, which of the following best describes what [you do/he does/she does] on a typical work day?

Mostly sitting
Mostly standing
Mostly walking
Mostly heavy labour or physically demanding work
Don’t know

Country: Canada

Survey: Canadian Health Measures Survey

Survey Code: CA 04

Year: 2007

Type: Face to Face

Question PAC_Q21 I

Next, some questions about the amount of time spent in the past 3 months on physical activity at work, while doing daily chores around the house, or doing errands, but not leisure time activity.

In a typical week in the past 3 months, how many hours did you usually spend walking to work or to school or while doing errands?

None
Less than 1 hour
From 1 to 5 hours
From 6 to 10 hours
From 11 to 20 hours
More than 20 hours

Question CPA_Q14

About how many hours a week does he usually take part in physical activity (that makes him out of breath or warmer than usual) in his class time at school?

Never
Less than 2 hours per week
2 to 3 hours per week
4 to 6 hours per week
7 or more hours per week

Country:	Canada
Survey:	Canadian Community Health Survey (CCHS)
Survey Code:	CA 05
Year:	2007
Type:	Face to Face + Telephone

Question WST_Q408

Your job required a lot of physical effort.

Strongly agree
Agree
Neither agree nor disagree
Disagree
Strongly disagree

Country: Switzerland

Survey: Swiss Health Survey

Survey Code: CH 03

Year: 2007

Type: Telephone + self administered

Question 46.02

And how many days per week do you work up a sweat (on average) while working, either in your job or working at home or in the garden?

... days per week (0 to 7 days)
No answer

Question 27

What about the physical stress/exertion at work?

Picture 2 workplaces:
In workplace A a person is generally exposed to a great deal of physical stress or exertion.
In workplace B a person generally has to cope with practically no physical stress or exertion.

Which of these two workplaces is more similar to yours?

Exactly like workplace A
Similar to workplace A
Between A and B
Similar to workplace B
Exactly like workplace B

Country:	Finland
Survey:	FINRISK 2007
Survey Code:	FI 21
Year:	2007
Type:	Self administered

Question 78

How demanding is your work physically? The activity at work is divided into four groups.

My work is mainly done sitting down and I do not walk much during my working hours (e.g. a clock smith, radio mechanic and industrial seamstress, office work at a desk).

I walk quite much in my work, but I do not have to lift or carry heavy objects (e.g. a foreman and store assistant, light industrial worker, office work which requires walking).

I have to walk and lift much or to take the stairs or go uphill (e.g. a carpenter or cattle minder/dairy work, engineering shop or other heavier industrial work).

My work is heavy manual labour in which I have to lift or carry heavy objects, to dig, shovel or chop (e.g. forestry, heavy farm work, heavy construction or industrial work).

Country:	Finland
Survey:	Health Behaviour and Health among the Finnish Adult Population
Survey Code:	FI 22
Year:	2007
Type:	Self administered

Question 75

How physically strenuous is your work?

my work is mainly sedentary and I don’t walk much
I walk quite a lot in my work, but do not need to lift or carry heavy objects
in my work I need to walk a great deal and often need to walk upstairs or uphill
I do heavy physical work: I need to lift or carry heavy objects, dig, shovel, or chop, etc.

Country: Ireland

Survey: Survey of Lifestyles, Attitudes and Nutrition SLAN

Survey Code: IE 09

Year: 2007

Type: Face to Face

Question B15

Thinking about how active you are in your job, in general would you say you are …?

Very physically active
Not very physically active
Fairly physically active
Not at all physically active
Not applicable

Country: Iceland

Survey: Health and wellbeing of Icelanders

Survey Code: IS 04

Year: 2007

Type: Self administered

Question 56

What of the following would best describe the physical exertion in your work or study?

Mostly sedentary work/study that does not require physical exertion
My work/study requires walking or an upright position but not physical exertion
My work/study requires walking or an upright position and lifting or carrying objects
Manual labour/study requires a lot of physical exertion
Does not apply, I am not employed or studying

International Survey Items concerning

Household Physical Activity

1992 - 2008

TABLE OF CONTENTS

1992
United Kingdom – UK 03 2

1995
United Kingdom – UK 10 3

1998
Ireland – IE 01 6
United Kingdom – UK 02 7
United Kingdom – UK 09 9
United Kingdom – UK 13 13

1999
Italy – IT 01 16

2000
International – EUPASS 17
Italy – IT 03 18
Romania – RO 01 19

2001
Italy – IT 04 20
Netherlands – NL 02 21
Netherlands – NL 03 22

2002
Switzerland – CH 02 23
Italy – IT 05 24
Malta – MT 01 25
Netherlands – NL 05 26
United Kingdom – UK 28 27

2003
Netherlands – NL 08 30

2004
Netherlands – NL 07 31

2005
Italy – IT 08 32
United Kingdom – UK 31 33

2006
United Kingdom – UK 33 35

2007
Switzerland – CH 03 38
Sweden – LNU 39
United Kingdom – UK 35 41

2008
France – FR 20 43
United Kingdom – UK 36 44

Country: United Kingdom

Survey: Health and Lifestyle Survey

Survey Code: UK 03

Year: 1992

Type: Face to Face

Question 86

a. In the last 7 days, have you done any 'do it yourself'
I mean house and car maintenance, building,
carpentry, etc.?

Yes
No

b. How much time, overall, did you spend on 'do it yourself'
in the last 7 days?

… time
Don't know

c. Would you describe any of this work 'heavy'?

Yes
No
Don’t know

Country:	United Kingdom
Survey:	The Scottish Health Survey
Survey Code:	UK 10
Year:	1995
Type:	Face to Face + self administered

Question 190

During an average week, would you normally do any heavy housework of the kind listed on this card, or any similar type of heavy housework?

SHOW CARD C.

Yes
No

Question 191

On how many occasions per week do you usually do this kind of heavy housework?

Less than once a week
Once a week
2-3 times a week
4-5 times a week
6-7 times a week or more

Question 192

How much time do you usually spend doing this kind of heavy housework?

Less than 10 minutes
10 minutes, less than 20 minutes
20 minutes, less than 30 minutes
30 minutes, less than 2 hours
2 hours or longer

Question 193

During an average week in the spring or summer, would you normally do any heavy gardening or DIY of the kind listed on this card, or any similar type of heavy gardening or DIY?

SHOW CARD D.

Yes
No

Question 194

During the spring or summer, on how many occasions per week do you usually do this kind of heavy gardening or DIY?

Less than once a week
Once a week
2-3 times a week
4-5 times a week
6-7 times a week or more

Question 195

How much time do you usually spend doing this kind of heavy gardening or DIY during the spring or summer?

Less than 10 minutes
10 minutes, less than 20 minutes
20 minutes, less than 30 minutes
30 minutes, less than 2 hours
2 hours or longer

Question 196

During an average week in the autumn or winter, would you normally do any heavy gardening or DIY of the kind listed on this card, or any similar type of heavy gardening or DIY?

SHOW CARD D.

Yes
No

Question 197

During the autumn or winter, on how many occasions per week do you usually do this kind of heavy gardening or DIY?

Less than once a week
Once a week
2-3 times a week
4-5 times a week
6-7 times a week or more

Question 198

How much time do you usually spend doing this kind of heavy gardening or DIY during the autumn or winter?

Less than 10 minutes
10 minutes, less than 20 minutes
20 minutes, less than 30 minutes
30 minutes, less than 2 hours
2 hours or longer

Country: Ireland

Survey: Survey of Lifestyle, Attitudes and Nutrition (SLÁN)

Survey Code: IE 01

Year: 1998

Type: Self administered

Question B3

Do you do light household work? (e.g. dusting, washing dishes, repairing clothes)

Seldom/Never
1-3 times per month
Once per week
3-4 times per week
Most days

Question B4

Do you do heavy household work? (e.g. vacuuming/ hoovering, washing floors and windows, carrying rubbish bags)

Seldom/Never
1-3 times per month
Once per week
3-4 times per week
Most days

Country:	United Kingdom
Survey:	Health Education Monitoring Survey
Survey Code:	UK 02
Year:	1998
Type:	Face to Face

Question 103

I'd like you to think about physical activities you have done when you were not doing your paid job.
Have you done any housework in the past 4 weeks?

Yes
No

Have you done any hoovering, dusting or ironing in the past 4 weeks?

Yes
No

Question 104

Some kinds of housework are heavier than others. This card gives examples of heavy housework, it does not include everything, these are just examples. Was any of the housework you did in the past 4 weeks this kind of heavy housework?

SHOW CARD Y

Yes
No

During the past 4 weeks on how many separate days have you done that kind of heavy housework?

Question 105

Have you done any gardening, did you or building in the past 4 weeks?

Yes
No

Have you done any gardening, did you or building work

listed on this card?

SHOW CARD Z

Yes
No

Have you done any gardening, did you or building work from this card, or any similar heavy manual work?

SHOW CARD AA

Yes
No

During the past 4 weeks, on how many days have you done this kind of heavy manual gardening or did you?

Country: United Kingdom

Survey: Health Survey for England

Survey Code: UK 09

Year: 1998

Type: Face to Face + self administered

Question 133

In the last week have you done any housework or gardening which involved pulling or pushing, like hoovering, cleaning a car, mowing grass or sweeping up leaves for at least 15 minutes a time?

Yes
No

Question 134

On how many days in the last week have you done any housework or gardening of this type for at least 15 minutes a time?

One day
Two days
Three days
Four days
Five days
Six days
Every day

Question 135

On each day that you did any housework or gardening of this type for at least 15 minutes a time, how long did you spend?

Show card G

15 minutes, less than 30 minutes
30 minutes, less than 1 hour
1 hour, less than 1½ hours
1½ hours, less than 2 hours
2 hours, less than 2½ hours
2½ hours, less than 3 hours
3 hours, less than 3½ hours
3½ hours, less than 4 hours

4 hours or more

Question 136

How long did you spend doing housework or gardening on each day?

… hours … minutes

Question 166

I'd like you to think about the physical activities you have done in the last few weeks (when you were not doing your paid job.)

Have you done any housework in the past four weeks, that is from (date four weeks ago) up to yesterday?

Yes
No

Question 167

Have you done any housework listed on this card?

Show card K

Yes
No

Question 168

Some kinds of housework are heavier than others. This card gives some examples of heavy housework. It does not include everything, these are just examples. Was any of the housework you did in the last four weeks this kind of heavy housework?

Show card L

Yes
No

Question 169

During the past four weeks on how many days have you done this kind of heavy housework?

… days

Question 170

On the days you did heavy housework, how long did you usually spend?

… hours … minutes

Question 171

Have you done any gardening, do it yourself or building work in the past four weeks, that is since (date four weeks ago)?

Yes
No

Question 172

Have you done any gardening, do it yourself or building work listed on this card?

Show card M

Yes
No

Question 173

Have you done any gardening, do it yourself or building work from this other card, or any similar heavy manual work?

Show card N

Yes
No

Question 174

During the past 4 weeks on how many days have you done this kind of heavy manual gardening or do it yourself?

… days

Question 175

On the days you did heavy manual gardening or do it yourself, how long did you usually spend?

Country: United Kingdom

Survey: The scottish health survey

Survey Code: UK 13

Year: 1998

Type: Face to Face

Question 178

I'd like you to think about the physical activities you have done in the last few weeks (when you were not doing your paid job.) Have you done any housework in the past four weeks, that is from (date four weeks ago) up to yesterday?

Yes
No

Have you done any housework listed on this card?

SHOW CARD F

Yes
No

Some kinds of housework are heavier than others. This card gives some examples of heavy housework. It does not include everything, these are just examples. Was any of the housework you did in the last four weeks this kind of heavy housework?

SHOW CARD G

Yes
No

During the past four weeks on how many days have you done this kind of heavy housework?

…

On the days you did heavy housework, how long did you usually spend?

…

RECORD MINUTES SPENT ON HEAVY HOUSEWORK.

…

Question 179

Have you done any gardening, DIY or building work in the past four weeks, that is since (date four weeks ago)?

Yes
No

Have you done any gardening, DIY or building work listed on this card?

SHOW CARD H

Yes
No

Have you done any gardening, DIY or building work from this other card, or any similar heavy manual work?

SHOW CARD I

Yes
No

During the past 4 weeks on how many days have you done this kind of heavy manual gardening or DIY?

…

On the days you did heavy manual gardening or DIY, how long did you usually spend?

…

RECORD MINUTES SPENT ON GARDENING OR DIY.

Question 184

In the last week have you done any housework or gardening which involved pulling or pushing, like hoovering, cleaning a car, mowing grass or sweeping up leaves for at least 15 minutes a time?

Yes
No

On how many days in the last week have you done any housework or gardening of this type for at least 15 minutes a time?

One day
Two days
Three days
Four days
Five days
Six days

Every day

On each day that you did any housework or gardening of this type for at least 15 minutes a time, how long did you spend?
Please give an answer from this card.

SHOW CARD K

15 minutes, less than 30 minutes
30 minutes, less than 1 hour
1 hour, less than 1½ hours
1½ hours, less than 2 hours
2 hours, less than 2½ hours
2½ hours, less than 3 hours
3 hours, less than 3½ hours
3½ hours, less than 4 hours
4 hours or more, please specify how long: … hours
… minutes

How long did you spend doing housework or gardening on each day?

…

Country: Italy

Survey: Health Conditions and the Use of Health Services

Survey Code: IT 01

Year: 1999

Type: Face to Face

Question 15.7

Can your domestic work mainly be considered a physical activity?

Light
Moderate
Heavy, till perspiring

Country:	International
Survey:	European Physical Activity Surveillance System (EUPASS)
Survey Code:	
Year:	2000
Type:	Telephone

Question B5c

How much physical activity did you do in and around your home including housework, gardening, general maintenance, or caring for your family in the last 7 days?

Much
Moderate
Little or no

Country:	Italy
Survey:	Aspects of daily living
Survey Code:	IT 03
Year:	2000
Type:	Face to Face

Question 4.2

Is your domestic and extradomestic work mainly characterised by scarce, moderate or heavy physical activity? (one answer for each line)

Scarce
Moderate
Heavy

Family or housework
Working activity

Country: Romania

Survey: Health Interview Survey

Survey Code: RO 01

Year: 2000

Type: Face to Face

Question 112

How would you describe the physical effort involved by your domestic activities?

Small
Average
Intense

Country: Italy

Survey: Aspects of daily living

Survey Code: IT 04

Year: 2001

Type: Face to Face

Question 5.2

Is your domestic and extradomestic work mainly characterised by scarce, moderate or heavy physical activity? (one answer for each line)

Scarce
Moderate
Heavy

Family or housework
Working activity

Country: Netherlands

Survey: Patient survey - Second Dutch National Survey of General Practice

Survey Code: NL 02

Year: 2001

Type: Face to Face

Question LRV44s1

And how many days a week do you do more strenuous household chores such as scrubbing floors, carrying heavy shopping, etc?

Question LRV44s2

And how much time on average per day in hours and minutes do you spend on this?

Number of hours: ...

Question LRV44s3

Number of minutes: …

Country: Netherlands

Survey: Continuous Quality of Life Survey

Survey Code: NL 03

Year: 2001

Type: Self administered

Question 367

Domestic activities

light and moderately strenuous domestic work (standing work like cooking, dishwashing, ironing, feeding / bathing children and running work like hoovering, shopping)

heavy domestic work (scrubbing, beating out the rugs, walking with heavy shopping bags)

number of days per week

Days:…

average time per day

hours: … min: …

Country:	Switzerland
Survey:	Swiss Health Survey 2002
Survey Code:	CH 02
Year:	2002
Type:	Telephone

Question 44.02

And in your work, whether a professional activity,
household chores or gardening, how many
days a week do you perspire (on average)?

… days a week (between 0 and 7 days)
no reply

Country: Italy

Survey: Aspects of daily living

Survey Code: IT 05

Year: 2002

Type: Face to Face

Question 5.2

Is your domestic and extradomestic work mainly characterised by scarce, moderate or heavy physical activity? (one answer for each line)

Scarce
Moderate
Heavy

Family or housework
Working activity

Country: Malta

Survey: First National Health Interview Survey

Survey Code: MT 01

Year: 2002

Type: Face to Face

Question 63

HOUSEHOLD ACTIVITIES

Do you do light household work (e.g. dusting, washing dishes, changing light bulbs/plugs)?

Most days
3-4 times per week
Once per week
1-3 times per month
Seldom/never

Question 64

Do you do heavy household work (e.g. washing floors and windows, vacuuming/hoovering, plastering/whitewashing)?

Most days
2-3 times per week
Once per week
1-3 times per month
Seldom/never

Country: Netherlands

Survey: Continuous Quality of Life Survey

Survey Code: NL 05

Year: 2002

Type: Self administered

Question 367

Domestic activities

light and moderately strenuous domestic work (standing work like cooking, dishwashing, ironing, feeding / bathing children and running work like hoovering, shopping)

heavy domestic work (scrubbing, beating out the rugs, walking with heavy shopping bags)

number of days per week

Days:…

average time per day

hours: … min: …

Country: United Kingdom

Survey: Health Survey for England

Survey Code: UK 28

Year: 2002

Type: Face to Face

Question 150

In the last week have you/has (name of child) done any housework or gardening which involved pulling or pushing, like hoovering, cleaning a car, mowing grass or sweeping up leaves for at least 15 minutes a time?

Yes
No

Question 151

On how many days in the last week have you/has (name of child) done any housework or gardening of this type for at least 15 minutes a time?

One day
Two days
Three days
Four days
Five days
Six days
Every day

Question 152

On each day that you/(name of child) did any housework or gardening of this type for at least 15 minutes a time, how long did you/s/he spend?

Please give an answer from this card.

15 minutes, less than 30 minutes
30 minutes, less than 1 hour
1 hour, less than 1 1/2 hours
1 1/2 hours, less than 2 hours
2 hours, less than 2 1/2 hours
2 1/2 hours, less than 3 hours
3 hours, less than 3 1/2 hours
3 1/2 hours, less than 4 hours
4 hours or more, please specify how long: How long did you/(name of child) spend doing housework or gardening on each day?

hours :… minutes: ….

Question 167a

Have you done any housework when you were not doing your paid job/in the past four weeks, that is from (date four weeks ago) up to yesterday?

Yes
No

Question 167b

IF Yes, have you done any housework listed on this card?

Yes
No

Question 168a

Some kinds of housework are heavier than others. This card gives some examples of heavy housework. It does not include everything, these are just examples. Was any of the housework you did in the last four weeks this kind of heavy housework?"

Yes
No

Question168b

IF Yes , during the past four weeks on how many days have you done this kind of heavy housework for at least 30 minutes a time?

… days

Question 169a

Have you done any gardening, Do It Yourself or building work in the past four weeks, that is since (date four weeks ago)?

Yes
No

Question 169b

IF Yes, Have you done any gardening, Do It Yourself or building work listed on this card?

Yes
No

Question 170a

Have you done any gardening, Do It Yourself or building work from this other card, or any similar heavy manual work?

Yes
No

Question 170b

IF Yes, during the past four weeks on how many days have you done this kind of heavy manual gardening or Do It Yourself, for at least 30 minutes a time?

… days

Country:	Netherlands
Survey:	Continuous Quality of Life Survey
Survey Code:	NL 08
Year:	2003
Type:	Self administered

Question 367

Domestic activities

light and moderately strenuous domestic work (standing work like cooking, dishwashing, ironing, feeding / bathing children and running work like hoovering, shopping)

heavy domestic work (scrubbing, beating out the rugs, walking with heavy shopping bags)

number of days per week

Days:…

average time per day

hours: …   min: …

Country: Netherlands

Survey: Continuous Quality of Life Survey

Survey Code: NL 07

Year: 2004

Type: Self administered

Question 344

Domestic activities

light and moderately strenuous domestic work (standing work like cooking, dishwashing, ironing, feeding/bathing children and running work like hoovering, shopping)

heavy domestic work (scrubbing, beating out the rugs, walking with heavy shopping bags)

number of days per week

Days:…

average time per day

hours: … min: …

Country: Italy

Survey: Health Conditions and the Use of Health Services 2004-2005

Survey Code: IT 08

Year: 2005

Type: Face to Face

Question 17.2

How many hours and minutes per week, on average, do you devote to domestic and family-oriented work (housework, shopping, care of other family members)?

Do not carry out activities
N° hours: ...
N° minutes: ...

Question 17.3

Is your domestic and family-oriented work mainly characterised by physical activity?

Yes, light
Yes, moderate
Yes, heavy, till perspiring

Country:	United Kingdom
Survey:	Health Survey for England
Survey Code:	UK 31
Year:	2005
Type:	Face to Face

Question HWkCh

In the last week have you/has (name of child) done any housework or gardening which involved pulling or pushing, like hoovering, cleaning a car, mowing grass or sweeping up leaves for at least 15 minutes a time?

Yes
No

Question DHWkCh

On how many days in the last week have you/has (name of child) done any housework or gardening of this type for at least 15 minutes a time?

One day
Two days
Three days
Four days
Five days
Six days
Every day

Question THWk

On each day that you/(name of child) did any housework or gardening of this type for at least 15 minutes a time, how long did you/s/he spend?
Please give an answer from this card.

15 minutes, less than 30 minutes
30 minutes, less than 1 hour
1 hour, less than 1 1/2 hours
1 1/2 hours, less than 2 hours
2 hours, less than 2 1/2 hours
2 1/2 hours, less than 3 hours
3 hours, less than 3 1/2 hours
3 1/2 hours, less than 4 hours
4 hours or more (please specify how long)

Question HWkHrs

How long did you/(name of child) spend doing housework or gardening on each day?

...

Country:	United Kingdom
Survey:	Health Survey for England
Survey Code:	UK 33
Year:	2006
Type:	Face to Face

Question HWkCh

In the last week have you/has (name of child) done any housework or gardening which involved pulling or pushing, like hoovering, cleaning a car, mowing grass or sweeping up leaves for at least 15 minutes a time?

Yes
No

Question DHWkCh

On how many days in the last week have you/has (name of child) done any housework or gardening of this type for at least 15 minutes a time?

One day
Two days
Three days
Four days
Five days
Six days
Every day

Question THWk

On each day that you/(name of child) did any housework or gardening of this type for at least 15 minutes a time, how long did you/s/he spend?

15 minutes, less than 30 minutes
30 minutes, less than 1 hour
1 hour, less than 1 1/2 hours
1 1/2 hours, less than 2 hours
2 hours, less than 2 1/2 hours
2 1/2 hours, less than 3 hours
3 hours, less than 3 1/2 hours
3 1/2 hours, less than 4 hours
4 hours or more, please specify how long: ...

Question HWkHrs

How long did you/(name of child) spend doing housework or gardening on each day?

RECORD HOURS SPENT BELOW

...

RECORD HERE MINUTES SPENT WALKING.

...

Question HevyHWrk-HvH

Some kinds of housework are heavier than others. This card gives some examples of heavy housework. It does not include everything, these are just examples. Was any of the housework you did in the last four weeks this kind of heavy housework?

Yes
No

During the past four weeks on how many days have you done this kind of heavy housework for at least 30 minutes a time?

...

Question HeavyDay

During the past four weeks on how many days have you done this kind of heavy housework?

...

Question HrsHHW

On the days you did heavy housework, how long did you usually spend?

...

Question Garden

Have you done any gardening, DIY or building work in the past four weeks, that is since (date four weeks ago)?

Yes
No

Have you done any gardening, DIY or building work listed on this card?

Yes
No

Question ManWork

Have you done any gardening, DIY or building work from this other card, or any similar heavy manual work?

Yes
No

During the past four weeks on how many days have you done this kind of heavy manual gardening or DIY, for at least 30 minutes a time?

...

Question ManDays

During the past four weeks on how many days have you done this kind of heavy manual gardening or DIY?

...

Question HrsDIY

On the days you did heavy manual gardening or DIY, how long did you usually spend?

...

Record minutes spent on gardening or DIY

...

Country:	Switzerland
Survey:	Swiss Health Survey
Survey Code:	CH 03
Year:	2007
Type:	Telephone + self administered

Question 46.02

And how many days per week do you work up a sweat (on average) while working, either in your job or working at home or in the garden?

... days per week (0 to 7 days)
No answer

Country:	Sweden
Survey:	Swedish Level of Living Survey
Survey Code:	
Year:	2007
Type:	Face to Face

Question 240a

About how many hours per week on average are spent altogether in your household on buying groceries, cooking and washing dishes?

HOURS PER WEEK

If respondent lives alone: Question 241 a

Question 240b

About how many of these hours do you do this work yourself?

HOURS PER WEEK

Question 241a

About how many hours per week on average are spent altogether in your household on laundry, ironing and other care of clothing?

HOURS PER WEEK

If respondent lives alone: Question 242 a

Question 241b

About how many of these hours do you do this work yourself?

HOURS PER WEEK

Question 242a

About how many hours per week on average are spent altogether in your household on cleaning?

HOURS PER WEEK

If respondent lives alone: Question 243 a

Question 242b

About how many of these hours do you do this work yourself?
HOURS PER WEEK

Question 243a

On average, about how many hours per week are spent altogether on repair and maintenance of your residence, motor vehicle and other property belonging to your household?

HOURS PER WEEK

If respondent lives alone: Question 244

Question 243b

About how many of these hours do you do this work yourself?

HOURS PER WEEK

Country: United Kingdom

Survey: Health Survey for England

Survey Code: UK 35

Year: 2007

Type: Face to Face + self administered

Question HWkCh

In the last week have you/has (name of child) done any housework or gardening which involved pulling or pushing, like hoovering, cleaning a car, mowing grass or sweeping up leaves for at least 15 minutes a time?

Yes
No

Question DHWkCh

On how many days in the last week have you/has (name of child) done any housework or gardening of this type for at least 15 minutes a time?

One day
Two days
Three days
Four days
Five days
Six days
Every day

Question THWk

On each day that you/(name of child) did any housework or gardening of this type for at least 15 minutes a time, how long did you/s/he spend?

15 minutes, less than 30 minutes
30 minutes, less than 1 hour
1 hour, less than 1 1/2 hours
1 1/2 hours, less than 2 hours
2 hours, less than 2 1/2 hours
2 1/2 hours, less than 3 hours
3 hours, less than 3 1/2 hours
3 1/2 hours, less than 4 hours
4 hours or more, please specify how long: ...

Question HWkHrs

How long did you/(name of child) spend doing housework or gardening on each day?

RECORD HOURS SPENT BELOW

...

RECORD HERE MINUTES SPENT WALKING.

...

Country:	France
Survey:	Disabilities and health survey, household part 2008
Survey Code:	FR 20
Year:	2008
Type:	Face to Face

Question MBRIJAR

In the past 12 months have you done any D.I.Y. or gardening? (apart from very small jobs or repairs)

Yes
No
Doesn't know

Was this:

Regularly throughout the year
Occasionally throughout the year
Only at certain periods or during holidays
Occasionally or rarely
Doesn't know

Country: United Kingdom

Survey: Health Survey for England

Survey Code: UK 36

Year: 2008

Type: Face to Face

Question HWkCh

In the last week have you/has (name of child) done any housework or gardening which involved pulling or pushing, like hoovering, cleaning a car, mowing grass or sweeping up leaves for at least 15 minutes a time?

Yes
No

Question DHWkCh

On how many days in the last week have you/has (name of child) done any housework or gardening of this type for at least 15 minutes a time?

One day
Two days
Three days
Four days
Five days
Six days
Every day

Question THWk

On each day that you/(name of child) did any housework or gardening of this type for at least 15 minutes a time, how long did you/s/he spend?

15 minutes, less than 30 minutes
30 minutes, less than 1 hour
1 hour, less than 1 1/2 hours
1 1/2 hours, less than 2 hours
2 hours, less than 2 1/2 hours
2 1/2 hours, less than 3 hours
3 hours, less than 3 1/2 hours
3 1/2 hours, less than 4 hours
4 hours or more, please specify how long: ...

Question HWkHrs

How long did you/(name of child) spend doing housework or gardening on each day?

RECORD HOURS SPENT BELOW

...

RECORD HERE MINUTES SPENT WALKING.

...

International Survey Items concerning

Transport-related Physical Activity

1997 - 2007

TABLE OF CONTENTS

1997
Finland – FI 02 2

2000
Canada – CA 01 3
Finland – FI 01 4
Finland – FI 03 5
International – EUPASS 6

2001
Finland – FI 06 7
Netherlands – NL 02 8
Netherlands – NL 03 10

2002
Estonia – EE 04 11
Finland – FI 07 12
Finland – FI 13 13
International – INT 08 14
Slovakia – SK 02 15

2003
Canada – CA 02 16
Finland – FI 10 17
Croatia – HR 04 18
International – INT 12 19

2004
Estonia – EE 07 20
Finland – FI 12 21
International – FINBALT 22

2005
Canada – CA 03 23
Denmark – DK 03 24
Finland – FI 19 25

2006
Lithuania – LT 05 26

2007
Australia – AU 04 27
Canada – CA 04 28
Canada – CA 05 29
Switzerland – CH 03 30
Finland – FI 21 31
Finland – FI 22 32

Country: Finland

Survey: FINRISK 97

Survey Code: FI 02

Year: 1997

Type: Face to Face + self administered

Question 69

How many minutes do you walk, ride on a bicycle or otherwise exercise to get to work?

(Please count in both travelling to and from work.)

I am not at work or I use only a motorised vehicle
under 15 minutes daily
15 - 29 minutes daily
30 - 44 minutes daily
45 - 59 minutes daily
over an hour daily

Country:	Canada
Survey:	Canadian Community Health Survey Cycle 1.1
Survey Code:	CA 01
Year:	2000
Type:	Face to Face

<u>Question PA4B</u>

In a typical week, how much time did you usually spend bicycling to work or to school or while doing errands?

None
Less than 1 hour
From 1 to 5 hours
From 6 to 10 hours
From 11 to 20 hours
More than 20 hours

Country: Finland

Survey: Survey on health behaviour

Survey Code: FI 01

Year: 2000

Type: Self administered

Question 88

How many minutes do you walk or bicycle on your way to and from work? Please indicate the total time.

I do not work or I work at home
I always travel to and from work by motor vehicle
less than 15 minutes a day
15 - 30 minutes a day
30 - 60 minutes a day
over one hour a day

Country:	Finland
Survey:	Health 2000
Survey Code:	FI 03
Year:	2000
Type:	Self administered

Question 37

How many minutes do you walk or cycle on your daily way to and from work?
Add the times to travel to work and to travel back home.

I do not work or I work at home
I use a motor vehicle for the entire trip
less than 15 minutes a day
from 15 to 29 minutes a day
from 30 to 59 minutes a day
from 1 to 2 hours a day
2 hours or longer a day

Country: International

Survey: European Physical Activity Surveillance System (EUPASS)

Survey Code:

Year: 2000

Type: Telephone

Question B5b

How much physical activity did you do for the purpose of transportation in the last 7 days?

Much
Moderate
Little or no

Country: Finland

Survey: Health Behaviour among the Finnish Adult Population

Survey Code: FI 06

Year: 2001

Type: Face to Face

Question 78

How many minutes do you walk or bicycle on your way to and from work? Please indicate the total time.

I do not work or I work at home
I always travel to and from work by motor vehicle
less than 15 minutes a day
15 – 30 minutes a day
30 – 60 minutes a day
over one hour a day

Country: Netherlands

Survey: Patient survey - Second Dutch National Survey of General Practice

Survey Code: NL 02

Year: 2001

Type: Face to Face

Question LRV44a1

We will now continue by looking at your normal day-to-day activities.
Would you keep in mind a normal week during the past few months? I am going to give a number of activities and then ask you a couple of questions per activity.

We will begin with going to and from work or school.

Walking to/from work or school. How many days a week do you do that?

...

Question LRV44a2

What is the average walking time per day in hours and minutes?

Number of hours: ...

Question LRV44a3

Number of minutes: …

Question LRV44b1

And cycling to/from work or school.
How many days a week do you do that?

...

Question LRV44b2

What is the average cycling time per day in hours and minutes?

Number of hours: ...

Question LRV44b3

Number of minutes: …

Country: Netherlands

Survey: Continuous Quality of Life Survey

Survey Code: NL 03

Year: 2001

Type: Self administered

Question 365

Imagine a normal week during the past months.
Could you indicate how many days per week you performed the activities mentioned below, how much time you spent on them on an average day and how strenuous these activities were?

walking to/from work or school
number of days per week
… Days
average time per day
… hours … min.

cycling to /from work or school
number of days per week
… Days
average time per day
… hours … min..

not applicable

effort

Slow
average
fast

Country:	Estonia
Survey:	Health Behaviour among Estonian Adult Population
Survey Code:	EE 04
Year:	2002
Type:	Face to Face

Question 65

How many minutes a day do you spend walking or riding a bicycle to and from work? (Combine time spent both ways).

I am not working at all or I work at home
I go to work by car
Less than 15 minutes a day
15-30 min. a day
30-60 min. a day
More than an hour a day

Country:	Finland
Survey:	FINRISK 2002
Survey Code:	FI 07
Year:	2002
Type:	Self administered

Question 75

How many minutes do you walk, ride on a bicycle or otherwise exercise to get to work?
(Please count in both travelling to and from work.)

I do not work or I use only a motorised vehicle
less than 15 minutes daily
15 - 29 minutes daily
30 - 44 minutes daily
45 - 59 minutes daily
over an hour daily

Country:	Finland
Survey:	Health Behaviour among the Finnish Adult Population
Survey Code:	FI 13
Year:	2002
Type:	Face to Face

Question 80

How many minutes do you walk or bicycle on your way to and from work?
Please indicate the total time.

I do not work or I work at home
I always travel to and from work by motor vehicle
less than 15 minutes a day
15 – 30 minutes a day
30 – 60 minutes a day
over one hour a day

Country:	International
Survey:	World Health Surveys-WHS-Consolidated multi-country questionnaire
Survey Code:	INT 08
Year:	2002
Type:	Face to Face

Question 4404b

During the last 7 days, on how many days did you walk or use a bicycle for travel for at least 10 minutes continuously?

Days per week

Question 4404c

On a typical day that you walked or used a bicycle for travel, how much time did you spend doing this?

Hours/minutes

Country: Slovakia

Survey: Health Monitor Survey

Survey Code: SK 02

Year: 2002

Type: Face to Face

Question 6.11

How many minutes a day do you spend walking or riding a bicycle to and from work?
(Combine time spent both ways).

I do not work at all or I work at home
I go to work by car
less than 15 minutes a day
15-30 minutes a day
30-60 minutes a day
more than one hour a day

Country: Canada

Survey: Canadian Community Health Survey Cycle 2.1

Survey Code: CA 02

Year: 2003

Type: Face to Face + Telephone

Question PAC_Q4A

In a typical week in the past 3 months, how many hours did you usually spend walking to work or to school or while doing errands?

None
Less than 1 hour
From 1 to 5 hours
From 6 to 10 hours
From 11 to 20 hours
More than 20 hours

Question PAC_Q4B

In a typical week, how much time did you usually spend bicycling to work or to school or while doing errands?

None
Less than 1 hour
From 1 to 5 hours
From 6 to 10 hours
From 11 to 20 hours
More than 20 hours

Country:	Finland
Survey:	Health Behaviour among the Finnish Adult Population
Survey Code:	FI 10
Year:	2003
Type:	Face to Face

Question 83

How many minutes do you walk or bicycle on your way to and from work?
Please indicate the total time.

I do not work or I work at home
I always travel to and from work by motor vehicle
less than 15 minutes a day
15 – 30 minutes a day
30 – 60 minutes a day
over one hour a day

Country:	Croatia
Survey:	Croatian Health Survey
Survey Code:	HR 04
Year:	2003
Type:	Face to Face

Question PHA 01

In part of this questionnaire we examine your physical activity

How many minutes a day do you spend walking or riding bicycle to and from work? (combine time spent both ways)

Does not work at all or works at home
Goes to work by car, public transportation or similar
Walks (ride a bike) less than 15 minutes a day
Walks (ride a bike) between 15 to 30 minutes a day
Walks (ride a bike) for more than 30 minutes a day

Country:	International
Survey:	Joint Canada/United States Survey of Health (JCUSH)
Survey Code:	INT 12
Year:	2003
Type:	Telephone

Question PAC_Q04A

Next, some questions about the amount of time you spent in the past 3 months on physical activity at work or while doing daily chores around the house, but not leisure time activity.

In a typical week in the past 3 months, how many hours did you usually spend walking to work or to school or while doing errands?

None
Less than 1 hour
From 1 to 5 hours
From 6 to 10 hours
From 11 to 20 hours
More than 20 hours

Question PAC_Q04B

In a typical week, how much time did you usually spend bicycling to work or to school or while doing errands?

None
Less than 1 hour
From 1 to 5 hours
From 6 to 10 hours
From 11 to 20 hours
More than 20 hours

Country: Estonia

Survey: Health behaviour among Estonian Adult Population

Survey Code: EE 07

Year: 2004

Type: Self administered

Question 71

How many minutes a day altogether do you spend walking or riding a bicycle to and from work?

less than 15 minutes a day
15–30 minutes a day
30–60 minutes a day
more than 60 minutes a day
I go to work by car/public transport
I am not working at all or I work at home

Country:	Finland
Survey:	Health Behaviour among the Finnish Adult Population
Survey Code:	FI 12
Year:	2004
Type:	Face to Face

Question 73

How many minutes do you walk or bicycle on your way to and from work?
Please indicate the total time.

I do not work or I work at home
I always travel to and from work by motor vehicle
less than 15 minutes a day
15 – 30 minutes a day
30 – 60 minutes a day
over one hour a day

Country:	International (Finland, Baltic States)
Survey:	FINBALT Health Monitor
Survey Code:	
Year:	2004
Type:	Self administered

Question 55

How many minutes a day do you spend walking or riding a bicycle to and from work?
(Combine time spent both ways).

1 I am not working at all or I work at home
2 I go to work by car
3 less than 15 minutes a day
4 15-30 min. a day
5 30-60 min. a day
6 more than an hour a day

Country: Canada

Survey: National Population Health Survey (NPHS)

Survey Code: CA 03

Year: 2005

Type: Face to Face + Telephone

Question PA_Q4A

Next, some questions about the amount of time you spent in the past 3 months on physical activity at work or while doing daily chores around the house, but not leisure time activity.

In a typical week in the past 3 months, how many hours did you usually spend walking to work or to school or while doing errands?

None
Less than 1 hour
From 1 to 5 hours
From 6 to 10 hours
From 11 to 20 hours
More than 20 hours

Question PA_Q4B

In a typical week, how much time did you usually spend bicycling to work or to school or while doing errands?

None
Less than 1 hour
From 1 to 5 hours
From 6 to 10 hours
From 11 to 20 hours
More than 20 hours

Country:	Denmark
Survey:	The Danish Health Interview Survey
Survey Code:	DK 03
Year:	2005
Type:	Face to Face

Question 75

How much of your daily transport time are you walking or cycling?
Transport time means time used for commuting to work or school or other education. First I will ask you to answer about summertime and then wintertime.
(This also includes running, roller-skating, skateboarding, etc. if it is part of daily transport.)

Summer
Winter

0–14 minutes
15–29 minutes
30–59 minutes
1 hour or more

Country:	Finland
Survey:	Health Behaviour among the Finnish Adult Population
Survey Code:	FI 19
Year:	2005
Type:	Self administered

Question 78

How many minutes do you walk or bicycle on your way to and from work?
Please indicate the total time.

I do not work or I work at home
I always travel to and from work by motor vehicle
less than 15 minutes a day
15 – 30 minutes a day
30 – 60 minutes a day
over one hour a day

Country: Lithuania

Survey: Lithuanian health behaviour monitoring

Survey Code: LT 05

Year: 2006

Type: Self administered

Question 73

How many minutes a day do you spend walking or riding a bicycle to and from work? (Combine time spent both ways)

I am not working at all or I work at home
I go to work by car
less than 15 minutes a day
15-30 min. a day
30-60 min. a day
more than an hour a day

Country: Australia

Survey: National Health Survey

Survey Code: AU 04

Year: 2007

Type: Face to Face

Question EXER_Q20

(Excluding walking for fitness, recreation and sport already reported), in the last week, did [you/proxy name] walk for at least 10 minutes continuously to get to and from places?

Yes
NO

Question EXER_Q21

How many days did [you/proxy name] walk for at least 10 minutes continuously to get to and from places in the last week?

...

Question EXER_Q22

How much time in total did [you/proxy name] spend walking to get to and from places in the last week?

hours: ...
minutes: ...

Country:	Canada
Survey:	Canadian Health Measures Survey
Survey Code:	CA 04
Year:	2007
Type:	Face to Face

Question PAC_Q21 I

Next, some questions about the amount of time spent in the past 3 months on physical activity at work, while doing daily chores around the house, or doing errands, but not leisure time activity.

In a typical week in the past 3 months, how many hours did you usually spend walking to work or to school or while doing errands?

None
Less than 1 hour
From 1 to 5 hours
From 6 to 10 hours
From 11 to 20 hours
More than 20 hours

Question PAC_Q22

(In a typical week in the past 3 months,) How many hours did you usually spend bicycling to work or to school or while doing errands?

None
Less than 1 hour
From 1 to 5 hours
From 6 to 10 hours
From 11 to 20 hours
More than 20 hours

Country: Canada

Survey: Canadian Community Health Survey (CCHS)

Survey Code: CA 05

Year: 2007

Type: Face to Face + Telephone

Question PAC_Q7

The last questions were about leisure time activities. Next, some questions about walking and bicycling that you do only as a way of getting to and from work or school.

Other than the (X) times you already reported walking for exercise was there any other time. Was there any time] in the past 3 months when you walked to and from work or school?

Yes
No
Does not work or go to school

How many times?

... Times

About how much time did you spend on each occasion?

1 to 15 minutes
16 to 30 minutes
31 to 60 minutes
More than one hour

Question PAC_Q8

Other than the (X) times you already reported bicycling was there any other time. Was there any other time] in the past 3 months when you bicycled to and from work or school?

Yes
No

How many times?

... Times

About how much time did you spend on each occasion?

1 to 15 minutes
16 to 30 minutes
31 to 60 minutes
More than one hour

Country:	Switzerland
Survey:	Swiss Health Survey
Survey Code:	CH 03
Year:	2007
Type:	Telephone + self administered

Question 46.20

As for your daily journeys, for example to work, to go shopping, to go out: do you usually make them ...

On foot
By bicycle
By public transport
By motor vehicle
No answer

Question 46.21

About how many minutes a day do you travel on foot or by bicycle?

... hours and ... minutes per day
Don't know
No answer

Country:	Finland
Survey:	FINRISK 2007
Survey Code:	FI 21
Year:	2007
Type:	Self administered

Question 80

How many minutes do you walk, ride on a bicycle or otherwise exercise to get to work? (Please count in both travelling to and from work.)

I do not work or I use only a motorized vehicle
less than 15 minutes daily
15 - 29 minutes daily
30 - 44 minutes daily
45 - 59 minutes daily
over an hour daily

Country: Finland

Survey: Health Behaviour and Health among the Finnish Adult Population

Survey Code: FI 22

Year: 2007

Type: Self administered

Question 73

How many minutes do you walk or bicycle on your way to and from work?

I do not work or I work at home
I always travel to and from work by motor vehicle
less than 15 minutes a day
15–30 minutes a day
30–60 minutes a day
over one hour a day

International Survey Items concerning

Leisure Time Physical Activity

1992 - 2008

TABLE OF CONTENTS

1992
United Kingdom – UK 03 4

1994
Denmark – DK 01 6
Italy – IT 02 7

1995
Spain – ES 01 9
Portugal – PT 01 10
United Kingdom – UK 10 11

1996
Estonia – EE 01 20
Iceland – IS 01 22
Denmark – REC 01 23

1997
Austria – AT 04 24
Belgium – BE 01 25
Switzerland – CH 01 26
Finland – FI 02 28

1998
Germany – DE 02 30
Germany – DE 05 32
Ireland – IE 01 33
Norway – NO 01 34
United Kingdom – UK 02 35
United Kingdom – UK 09 37
United Kingdom – UK 13 44

1999
Austria – AT 01 50
Spain – ES 04 51
France – FR 06 52
Italy – IT 01 56

2000
Canada – CA 01 58
Cyprus – CY 02 59
Finland – FI 01 60
Finland – FI 03 61
France – FR 07 62
International – EUPASS 63
Italy – IT 03 63
Romania – RO 01 65
United Kingdom – UK 12 66
United States – US 01 68

2001
Australia – AU 01 69
Finland – FI 06 70
France – FR 11 71
Iceland – IS 03 72
Italy – IT 04 73
Netherlands – NL 02 74
Slovenia – SI 02 76

2002
Switzerland – CH 02 77
Czech Republic – CZ 01 78
Finland – FI 07 80
Finland – FI 13 82
Ireland – IE 03 83
Italy – IT 05 84

Norway – NO 02	85
Norway – NO 03	86
Slovakia – SK 02	87
United Kingdom – UK 07	88
United Kingdom – UK 28	91

2003

Canada – CA 02	97
Cyprus – CY 01	102
Spain – ES 03	103
Finland – FI 10	104
Croatia – HR 04	105
International – INT 12	106
Italy – IT 07	108
Sweden – SE 03	109

2004

Australia – AU 03	110
Estonia – EE 07	113
Finland – FI 12	114
Greece – GR 05	115
International – FINBALT	116
Poland – PL 06	117
Sweden – SE 04	118
United States – US 02	119

2005

Canada – CA 03	122
Germany – DE 07	124
Denmark – DK 03	125
Finland – FI 19	126
Italy – IT 08	127
Italy – IT 09	128
Netherlands – NL 09	129
Sweden – SE 05	130
Sweden – SE 07	131
United Kingdom – UK 31	130

2006

Austria – AT 06	137
Serbia – CS 01	138
Germany – DE 08	140
Estonia – EE 08	141
Spain – ES 06	143
France – FR 18	144
Italy – IT 10	145
Lithuania – LT 05	146
Netherlands – NL 10	147
United Kingdom – UK 33	149
United States – US 04	155

2007

Australia – AU 04	158
Canada – CA 04	160
Canada – CA 05	163
Switzerland – CH 03	167
Finland – FI 21	169
Finland – FI 22	171
Ireland – IE 09	172
Italy – IT 11	173
Luxembourg – LU 04	175
Netherlands – NL 11	175
Sweden – SE 08	176
Sweden – SE 09	177
United Kingdom – UK 35	178

2008

Belgium – BE 06	181
France – FR 20	182
Romania – RO 02	183
United Kingdom – UK 36	185

Country:	United Kingdom
Survey:	Health and Lifestyle Survey
Survey Code:	UK 03
Year:	1992
Type:	Face to Face

Question 85

a. In the last 7 days, have you done any gardening?

Yes
No

b. How much time, overall, did you spend gardening in the last 7 days?

… time
Don't know

c. Would you describe any of the gardening as heavy?

Yes
No

Question 86

a. In the last 7 days, have you done any 'do it yourself'
I mean house and car maintenance, building,
carpentry, etc.?

Yes
No

b. How much time, overall, did you spend on 'do it yourself'
in the last 7 days?

… time
Don't know

c. Would you describe any of this work 'heavy'?

Yes
No
Don’t know

Question 87

a. In the last fortnight have you done any of the activities on this card?

Yes
No

b. Which of these activities have you done in the last fortnight?

c. How many times have you done … in the last fortnight?

… times

d. On average, how long did you spend doing it each time?

SHOW CARD D

Keep fit, aerobics etc.
Yoga
Cycling
Golf
Jogging, Running
Swimming
Table tennis
Basketball
Football
Rugby
Badminton
Tennis
Squash, Fives, Rackets
Cricket
Windsurfing, Sailing
Self defence, Boxing, Wrestling
Back-packing, Hiking, etc.
Walks of 2 miles or more
Dancing

Question 88

a. Have you done any other physical activities in the last fortnight?

Yes
No

b. What did you do?

...

c. How many times have you in the last fortnight?

… times

d. On average, how long did you spend doing it each time?

…

Country: Denmark

Survey: Danish Health and Morbidity Survey

Survey Code: DK 01

Year: 1994

Type: Face to Face

Question 80

If we look back on the past year, what would you say best describes your spare time activities?

Show card 14

heavy training and competitive sports regularly and several times a week
exercise or heavy gardening at least 4 hours a week
walk, bike or other easy exercise at least 4 hours a week (include Sunday excursions, light gardening and biking/walking to work
read, watch TV or other sedentary occupation
don't know
no information

Country: Italy

Survey: Aspects of daily life

Survey Code: IT 02

Year: 1994

Type: Face to Face + self administered

Question 12.1

Over the last twelve months, have you, at least once, taken part in any kind of physical or sports activity (even when not requiring considerable physical exertion) during your free time?

Include activities such as jogging, going for a walk in the woods, or playing bowls. Do not include activities taking place during school hours or for work reasons

NO
YES, but only occasionally
YES, all the time

Question 12.2

Which activity?
(more than one answer may be given)

Football, five-a-side football
Gymnastics, aerobic dancing, body building
Swimming
Tennis
Basketball
Volleyball
Jogging
Walking, hiking
Track and field athletics
Winter sports
Cycling
Hunting
Fishing
Boating, sailing etc. (windsurfing, yachting, water-skiing, canoeing etc.)
Wrestling, weight lifting and martial arts
Bowls
Other outdoor activities (trekking, mountaineering, hang gliding and horse-riding etc.)
Other non-team sports
Other team sports

Question 12.3

Considering all the activities you indicated above as a whole, how often haven you taken part in such activities over the last twelve months?
(only one answer may be given)

1-5 times (about once every quarter)
6-20 times (about once a month)
21-40 times (2-3 times a month, less than once a week)
41-60 times (about once a week)
61-120 times (about twice a week)
More than 120 times (more than twice a week)

Question 12.4

Have you carried out any sporting activity every month of the year?

YES
NO, I only carried out the activity for some months

Question 12.5

Over the last 12 months have you practiced sports in places upon payments?

NO
YES

Question 12.6

Over the last twelve months, have you had private sport classes or courses at your or your family's expenses?

NO
YES

Country: Spain

Survey: National Health Survey

Survey Code: ES 01

Year: 1995

Type: Face to Face

Question 29

What type of physical exercise do you take in your spare time? Tell me which of these possibilities best describes how you spend most of your spare time.

(SHOW CARD J)

I don't take any exercise. I spend virtually all of my free time sitting down (reading, watching television, in the cinema, etc.)
Occasional physical or sporting activity (walking or cycling, gardening, gentle gymnastics, recreational activities requiring slight exertion, etc.)
Regular physical activity, several times a month (tennis, gymnastics, running, swimming, cycling, team games, etc.)
Physical training several times a week
No answer

Country: Portugal

Survey: National Health Survey

Survey Code: PT 01

Year: 1995

Type: Face to Face

Question 8

DO YOU TAKE REGULAR EXERCISE IN ORDER TO KEEP FIT?

Yes
No
Don't know

Question 9

HOW MANY DAYS PER WEEK?

… days
Don't know

Question 10

AND FOR HOW MANY MINUTES EACH DAY ON AVERAGE?

… minutes
Don't know

Country: United Kingdom

Survey: The Scottish Health Survey

Survey Code: UK 10

Year: 1995

Type: Face to Face + self administered

Question 154

On how many occasions per week do you usually do exercises (press ups, sit ups, etc.)?

- Less than once a week
- Once a week
- 2-3 times a week
- 4-5 times a week
- 6-7 times a week or more

Question 155

How much time do you usually spend doing exercises (press ups, sit-ups, etc.) on each occasion?

- Less than 10 minutes
- 10 minutes, less than 20 minutes
- 20 minutes, less than 30 minutes
- 30 minutes, less than 2 hours
- 2 hours or longer

Question 156

When you do exercises (press-ups, sit-ups, etc.) do you usually find yourself

- breathing normally,
- breathing faster than normal,
- or gasping for breath?

Question 157

On how many occasions per week do you usually do aerobics/keep fit/dance for fitness?

Less than once a week
Once a week
2-3 times a week
4-5 times a week
6-7 times a week or more

Question 158

How much time do you usually spend doing aerobics/keep fit/dance for fitness on each occasion?

Less than 10 minutes
10 minutes, less than 20 minutes
20 minutes, less than 30 minutes
30 minutes, less than 2 hours
2 hours or longer

Question 159

When you do aerobics/keep fit/dance for fitness do you usually find yourself

breathing normally,
breathing faster than normal,
or gasping for breath?

Question 160

On how many occasions per week do you usually go dancing?

Less than once a week
Once a week
2-3 times a week
4-5 times a week
6-7 times a week or more

Question 161

How much time do you usually spend dancing?

Less than 10 minutes
10 minutes, less than 20 minutes
20 minutes, less than 30 minutes

30 minutes, less than 2 hours
2 hours or longer

<u>Question 162</u>

When you go dancing do you usually find yourself

breathing normally,
breathing faster than normal,
or gasping for breath?

<u>Question 163</u>

On how many occasions per week do you usually do weight training?

Less than once a week
Once a week
2-3 times a week
4-5 times a week
6-7 times a week or more

<u>Question 164</u>

How much time do you usually spend doing weight training?

Less than 10 minutes
10 minutes, less than 20 minutes
20 minutes, less than 30 minutes
30 minutes, less than 2 hours
2 hours or longer

<u>Question 165</u>

When you do weight training do you usually find yourself

breathing normally,
breathing faster than normal,
or gasping for breath?

<u>Question 166</u>

On how many occasions per week do you usually go swimming?

Less than once a week

Once a week
2-3 times a week
4-5 times a week
6-7 times a week or more

Question 167

How much time do you usually spend swimming?

Less than 10 minutes
10 minutes, less than 20 minutes
20 minutes, less than 30 minutes
30 minutes, less than 2 hours
2 hours or longer

Question 168

When you go swimming do you usually find yourself

breathing normally,
breathing faster than normal,
or gasping for breath?

Question 169

On how many occasions per week do you usually go running/jogging?

Less than once a week
Once a week
2-3 times a week
4-5 times a week
6-7 times a week or more

Question 170

How much time do you usually spend going running/jogging?

Less than 10 minutes
10 minutes, less than 20 minutes
20 minutes, less than 30 minutes
30 minutes, less than 2 hours
2 hours or longer

Question 171

When you go running/jogging do you usually find yourself

breathing normally,
breathing faster than normal,
or gasping for breath?

Question 172

On how many occasions per week do you usually play football/rugby?

Less than once a week
Once a week
2-3 times a week
4-5 times a week
6-7 times a week or more

Question 173

How much time do you usually spend playing football/rugby?

Less than 10 minutes
10 minutes, less than 20 minutes
20 minutes, less than 30 minutes
30 minutes, less than 2 hours
2 hours or longer

Question 174

When you play football/rugby do you usually find yourself

breathing normally,
breathing faster than normal,
or gasping for breath?

Question 175

On how many occasions per week do you usually play badminton or tennis?

Less than once a week
Once a week
2-3 times a week
4-5 times a week
6-7 times a week or more

Question 176

How much time do you usually spend playing badminton or tennis?

Less than 10 minutes
10 minutes, less than 20 minutes
20 minutes, less than 30 minutes
30 minutes, less than 2 hours
2 hours or longer

Question 177

When you play badminton or tennis do you usually find yourself

breathing normally,
breathing faster than normal,
or gasping for breath?

Question 178

On how many occasions per week do you usually play squash?

Less than once a week
Once a week
2-3 times a week
4-5 times a week
6-7 times a week or more

Question 179

How much time do you usually spend playing squash?

Less than 10 minutes
10 minutes, less than 20 minutes
20 minutes, less than 30 minutes
30 minutes, less than 2 hours
2 hours or longer

Question 180

When you play squash do you usually find yourself

breathing normally,
breathing faster than normal,
or gasping for breath?

Question 181

On how many occasions per week do you usually play golf/go hillwalking?

Less than once a week
Once a week
2-3 times a week
4-5 times a week
6-7 times a week or more

Question 182

How much time do you usually spend playing golf/going hill walking?

Less than 10 minutes
10 minutes, less than 20 minutes
20 minutes, less than 30 minutes
30 minutes, less than 2 hours
2 hours or longer

Question 183

When you play golf/go hillwalking do you usually find yourself

breathing normally,
breathing faster than normal,
or gasping for breath?

Question 193

During an average week in the spring or summer, would you normally do any heavy gardening or DIY of the kind listed on this card, or any similar type of heavy gardening or DIY?

SHOW CARD D.

Yes
No

Question 194

During the spring or summer, on how many occasions per week do you usually do this kind of heavy gardening or DIY?

Less than once a week
Once a week
2-3 times a week
4-5 times a week
6-7 times a week or more

Question 195

How much time do you usually spend doing this kind of heavy gardening or DIY during the spring or summer?

Less than 10 minutes
10 minutes, less than 20 minutes
20 minutes, less than 30 minutes
30 minutes, less than 2 hours
2 hours or longer

Question 196

During an average week in the autumn or winter, would you normally do any heavy gardening or DIY of the kind listed on this card, or any similar type of heavy gardening or DIY?

SHOW CARD D.

Yes
No

Question 197

During the autumn or winter, on how many occasions per week do you usually do this kind of heavy gardening or DIY?

Less than once a week
Once a week
2-3 times a week
4-5 times a week
6-7 times a week or more

Question 198

How much time do you usually spend doing this kind of heavy gardening or DIY during the autumn or winter?

Less than 10 minutes
10 minutes, less than 20 minutes
20 minutes, less than 30 minutes

30 minutes, less than 2 hours
2 hours or longer

Country: Estonia

Survey: Estonian Health Interview Survey

Survey Code: EE 01

Year: 1996

Type: Face to Face

Question G35

Have you ever in your life gone regularly in for competitive sports during at least one year?

Yes
No

Question G 36

When did you start with regular competitive sports?

Year 19 ...

Question G 37

How often did you go in for competitive sports in that period when your sporting was most intensive?

Every day (almost every day)
3–4 times in week
1–2 times in week
1–3 times in month

Question G 38

In what year did such period in your life start and when did it end?

Year of beginning 19 ...
Year of ending 19 ...

Question G 39

Next we are going to talk about physical activity that is not aimed to make sports records, but instead is for your health or just to have fun

from the activity, and let us call it recreational sports.

Have you ever in your life participated in recreational sports?

Yes
No

<u>Question G 40</u>

Have you gone in for recreational sports during the last four weeks?

Yes
No

<u>Question G 41</u>

In what year and month did you last go in for recreational sports?

Year 19 ...
Month: ...

<u>Question G 42</u>

How often did you go in for recreational sports during the last four weeks?

Every day (almost every day)
3–4 times in week
1–2 times in week
1–3 times in month

<u>Question G 43</u>

Has such frequency of going in for hobby sports been for you usual during the last 12 months, or do you usually go more or do you go less?

Yes, it is usual
No, usually I go more
No, usually I go less

Country: Iceland

Survey: Omnibus survey

Survey Code: IS 01

Year: 1996

Type: Telephone

Question 7

How often do you exercise to the extent that you sweat,
feel your heart beating faster, or shortness of breath ?

3 times a week or more often
1-2 times a week
2-3 times a month
Seldom/never (Don’t exercise that way regularly)
Deny to answer
Don't know

Country:	Denmark
Survey:	Recommendations WHO-EURO
Survey Code:	REC 01
Year:	1996
Type:	Face to Face

Question 1

What describes best your leisure time activities during the last year?

Hard training and competitive sport more than once a week
Jogging and other recreational sports or heavy gardening, at least 4 hours a week
Walking, bicycling or other light activities at least 4 hours a week
Reading, watching TV or other sedentary activities

Question 2

At least once a week do you engage in any regular activity, such as jogging, cycling, etc. long enough to work up sweat?

No
Yes
If yes:
How many days per week?
… days

Country: Austria

Survey: Sport, Leisure Time and Home Accidents, and Smoking Habits

Survey Code: AT 04

Year: 1997

Type: Face to Face

Question B 27

Which of the following types of sports do you engage in

regularly
occasionally
or never

Coding list A

10 Alpine skiing
11 Snowboarding
12 Ice skating
13 Other winter sports
14 Cycling
15 Mountain biking
16 Running, jogging
17 Hiking, mountaineering
18 Rock climbing
19 Gymnastics
20 Far Eastern martial arts
21 Football
22 Tennis
23 Squash
24 Other ball games
25 Inline skating
26 Skateboarding
27 Riding
28 Water sports
29 Other sports

In types of sports pursued, has there been no accident during the period from 1st December to 30th November 1997

one accident
Two accidents
or more than two accidents

Country:	Belgium
Survey:	Health Interview Survey
Survey Code:	BE 01
Year:	1997
Type:	Face to Face

<u>Questions AP.01</u>

What describes best your leisure time activities during
the last year?

Show card 11 - only one answer is possible

Hard training and competitive sport more than once a week;
Jogging and other recreational sports or gardening, at least 4 hours per week;
Jogging and other recreational sports or gardening, at most 4 hours per week;
Walking, bicycling or other light activities at least 4 hours a week;
Walking, bicycling or other light activities at most 4 hours a week;
Reading, watching TV or other sedentary activities.

Country:	Switzerland
Survey:	Swiss Health Survey
Survey Code:	CH 01
Year:	1997
Type:	Face to Face

<u>Question 48.00</u>

Do you work up a sweat caused by physical activity at least once a week in your leisure time?

Yes
No
No reply

<u>Question 48.10</u>

On how many days a week (on average)?

… day(s) a week (from 1 to 7 days)
No reply

<u>Question 48.40</u>

Do you do gymnastics, fitness or play sports?

Yes
No
No reply

<u>Question 48.50</u>

How do you practice these physical activities or sports?
Do you do them…?

Very intensively
Quite intensively
Averagely intensively
Not so intensively
Not at all intensively
No reply

Question 48.60

And for how many hours a week do you practice them?

No. of hours: …
Don’t know
No reply

Country: Finland

Survey: FINRISK 97

Survey Code: FI 02

Year: 1997

Type: Face to Face + self administered

Question 68

How much do you exercise and stress yourself physically in your leisure time? If it varies much according to different seasons, circle the alternative which best describes the average situation.

In my leisure time I read, watch TV, and work in the household with tasks which do not make me move much and which do not physically tax me.

In my spare time I walk, cycle or exercise otherwise at least 4 hours per week. This includes walking, fishing and hunting, light gardening etc. but excludes travel to work.

In my spare time I exercise to maintain my physical condition, f. ex. running, jogging, skiing, gymnastics, swimming, playing ball games or I do heavy gardening or the like for at least 3 hours per week.

In my spare time I regularly exercise competitive-wise several times a week running, orienteering, skiing, swimming, playing ball games or other heavy sports.

Question 70

How often do you exercise in your leisure time for at least half an hour so that you at least are mildly out of breath and sweaty?

daily
2-3 times a week
once a week
2-3 times a month
a few times a year or seldom
I have a disability or a disease which does not enable me to exercise

---

Question 71

How many times in a week do you exercise in your free time for at least half an hour so that you at least are mildly out of breath and sweaty?

... times a week

Question 72

How long does your usual leisure time activity take?

I do not exercise in my free time
less than 15 minutes
15 - 29 minutes
30 - 59 minutes
one hour or longer

Question 73

How many kilometres do you usually run, jog or ski in a week?

... kilometres per week

Question 74

How many minutes do you daily walk, cycle or engage in a hobby in your leisure time that requires moving about (yard work or gardening, fixing or cleaning the house) ?

Do not count in the activity needed at work, travelling to work or leisure time sports

less than 15 minutes per day
15-29 minutes per day
30-44 minutes daily
45-59 minutes daily
over an hour per day

Country: Germany

Survey: Survey on living conditions, health and environment

Survey Code: DE 02

Year: 1998

Type: Self administered

Question 21

How often do you do sport?

Regularly, more than 2 hours a week
Regularly, 1 to 2 hours a week
Regularly, but less than 1 hour a week
Occasionally, e.g. only on holiday
Never

Question 22

Do you do sport…

on medical advice?
For your own interest?

Question 23

The following list contains some of the things that one can do in one's leisure time. Please enter a cross against the frequency you do these things.

Answer categories:
Daily
At least once a week or at the week-end
Once a month or less often
Occasionally
Not at all any more

Walking or hiking
Actively doing sport
Watching television
Reading for entertainment or relaxation
Doing work on the house
Gardening
Going to the theatre, concert or opera
Going to the cinema

Attending other organised events (e.g. clubs, associations, etc.)
Finishing off office work
Earning additional money/money from a second job
Pursuing voluntary commitments
Pursuing a hobby, such as handicrafts, music making or model building
Going to the restaurant or pub
Meeting friends or acquaintances
Meeting relatives

Country: Germany

Survey: German National Health Examination and Interview Survey

Survey Code: DE 05

Year: 1998

Type: ???

Question 61

When answering the questions about physical activity, please consider the last three months.

How often do you do sports?

Regularly, more than 4 hours per week
Regularly, 2 - 4 hours per week
Regularly, 1 - 2 hours per week
Less than 1 hour per week
No sports activities

Question 63

How often do you do sports or other strenuous activities in your free time, which cause you to sweat or to be out of breath?

answer categories
Daily
3-6 times per week
1-2 times per week
Seldom, about once a month
Never

(Without a break)
Less than 10 minutes
10-20 minutes
20-30 minutes
30 minutes or more

Question 64

When you climb three flights of stairs are you (normally) out of breath and/or do you sweat?

Yes
No
Unable to climb 3 flights of stairs

Country:	Ireland
Survey:	Survey of Lifestyle, Attitudes and Nutrition (SLÁN)
Survey Code:	IE 01
Year:	1998
Type:	Self administered

Question B1

Considering a 7-day period (a week), how many times on average do you do the following kinds of exercise for more than 20 minutes during your free time?

a. STRENUOUS EXERCISE (HEART BEATS RAPIDLY)
   (e.g. running, jogging, hockey, football, soccer, squash, basketball, judo, roller skating, vigorous swimming, vigorous long distance cycling)

   … Times per Week

b. MODERATE EXERCISE (NOT EXHAUSTING)
   (e.g. fast walking, tennis, badminton, easy swimming, easy cycling, volleyball, baseball, popular and folk dancing, heavy gardening)

   … Times per Week

c. MILD EXERCISE (MINIMAL EFFORT)
   (e.g. yoga, golf, easy walking, fishing from river bank, bowling, archery, light gardening)

   … Times per Week

Country:	Norway
Survey:	Survey on Living Conditions
Survey Code:	NO 01
Year:	1998
Type:	Face to Face + self administered

Question H32

How much physical activity are you involved in during a normal week in the past 12 months. The way to and from work is considered leisure time.

Approximately how many hours per week were you engaged in light physical activity (no sweating or heavy breathing)

Not active
Less than 1 hour per week
1-2 hours per week
3 hours or more per week

And approximately how many hours per week were you engaged in hard activity or work-out (sweating/heavy breathing)

Not active
Less than 1 hour per week
1-2 hours per week
3 hours or more per week

Question H33

Do you sometimes exercise ? If so: how many days per week do you usually exercise?

never
less often than once a week
more often than once a week

PLEASE SPECIFY NUMBER OF DAYS DURING A NORMAL WEEK IN THE PAST 12 MONTHS : …

Country: United Kingdom

Survey: Health Education Monitoring Survey

Survey Code: UK 02

Year: 1998

Type: Face to Face

Question 103

I'd like you to think about physical activities you have done when you were not doing your paid job.
Have you done any housework in the past 4 weeks?

Yes
No

Have you done any hoovering, dusting or ironing in the past 4 weeks?

Yes
No

Question 107

Now I'd like you to think about any sports or exercise activities you do. Can you look at this card and tell me if you've done any of these types of activities during the past 4 weeks?

SHOW CARD BB

Yes
No

Which of the activities did you do?
SHOW CARD BB

Aerobics/keep fit/gymnastics
Bowls/Crown bowls
Circuit training/weight training
Cycling
Exercises
Dancing
Football/Rugby
Golf
Hiking
Hockey/Netball/Ice-skating
Jogging/Running/Athletics
Squash
Swimming
Tennis/Badminton

Any other sport or exercise activity like these
Any other sport or exercise activity like these
Other activity : please specify

Can you tell me on how many separate days did you (name of activity) during the past four weeks?
How much time did you usually spend (name of activity) on each day?

… time in minutes

During the past four weeks, was the effort of (name of activity) usually enough to make you feel out of breath or sweaty?

Yes
No

Country: United Kingdom

Survey: Health Survey for England

Survey Code: UK 09

Year: 1998

Type: Face to Face + self administered

Question 137

In the last week, that is last (day) up to yesterday, have you done any sports or exercise activities (not counting things done as part of school lessons)?

Show card H

Yes
No

Question 138

Did you do any of these sports or exercise activities at the weekend, that is last Saturday and Sunday (yesterday and last Sunday)?

Yes
No

Question 139

Was that on Saturday or Sunday or on both days?

Saturday only
Sunday only
Both Saturday and Sunday

Question 140

On Saturday and Sunday when you did these sports or exercise activities, how long did you spend daily?

Show card G

Less than 5 minutes
5 minutes, less than 15 minutes

15 minutes, less than 30 minutes
30 minutes, less than 1 hour
1 hour, less than 1½ hours
1½ hours, less than 2 hours
2 hours, less than 2½ hours
2½ hours, less than 3 hours
3 hours, less than 3½ hours
3½ hours, less than 4 hours
4 hours or more

Question 141

How long did you spend doing these sports or exercise activities?

… hours … minutes

Question 142

Still thinking about last week. On how many of the weekdays did you do any of these sports or exercise activities? (Please remember not to count things done as part of school lessons)

None in last week
1 day
2 days
3 days
4 days
5 days

Question 143

On each weekday that you did these sports or exercise activities, how long did you spend?

Show card G

Less than 5 minutes
5 minutes, less than 15 minutes
15 minutes, less than 30 minutes
30 minutes, less than 1 hour
1 hour, less than 1½ hours
1½ hours, less than 2 hours
2 hours, less than 2½ hours
2½ hours, less than 3 hours
3 hours, less than 3½ hours
3½ hours, less than 4 hours
4 hours or more

Question 144

How long did you spend doing these sports or exercise activities on each weekday?

… hours … minutes

Question 145

Now I would like to know about when you do active things, like the things on this card or other activities like these.

Did you do any active things like these at the weekend, that is last Saturday and Sunday?

Show card I

Yes
No

Question 146

Was that on Saturday or Sunday or on both days?

Saturday only
Sunday only
Both Saturday and Sunday

Question 147

On Saturday/Sunday when you did active things like these, how long did you spend daily?

Show card G

Less than 5 minutes
5 minutes, less than 15 minutes
15 minutes, less than 30 minutes
30 minutes, less than 1 hour
1 hour, less than 1½ hours
1½ hours, less than 2 hours
2 hours, less than 2½ hours
2½ hours, less than 3 hours
3 hours, less than 3½ hours
3½ hours, less than 4 hours
4 hours or more

Question 148

How long did you spend doing active things like these?

… hours … minutes

Question 149

Still thinking about last week. On how many of the weekdays did you do active things, like the things on this card or other activities like these (not counting things done as part of school lessons)?

Show card I

None in last week
1 day
2 days
3 days
4 days
5 days

Question 150

On each weekday that you did active things like these, how long did you spend?

Show card G

Less than 5 minutes
5 minutes, less than 15 minutes
15 minutes, less than 30 minutes
30 minutes, less than 1 hour
1 hour, less than 1½ hours
1½ hours, less than 2 hours
2 hours, less than 2½ hours
2½ hours, less than 3 hours
3 hours, less than 3½ hours
3½ hours, less than 4 hours
4 hours or more

Question 151

How long did you spend doing active things like these on each weekday?

… hours … minutes

Question 166

I'd like you to think about the physical activities you have done in the last few weeks (when you were not doing your paid job.)

Have you done any housework in the past four weeks, that is from (date four weeks ago) up to yesterday?

Yes
No

Question 171

Have you done any gardening, do it yourself or building work in the past four weeks, that is since (date four weeks ago)?

Yes
No

Question 172

Have you done any gardening, do it yourself or building work listed on this card?

Show card M

Yes
No

Question 173

Have you done any gardening, do it yourself or building work from this other card, or any similar heavy manual work?

Show card N

Yes
No

Question 174

During the past 4 weeks on how many days have you done this kind of heavy manual gardening or do it yourself?

… days

Question 175

On the days you did heavy manual gardening or do it yourself, how long did you usually spend?

… days

Question 184

Which have you done in the last four weeks?
Any others?

Swimming,
Cycling,
Workout at a gym/Exercise bike/ Weight training
Aerobics/Keep fit/Gymnastics/ Dance for fitness
Any other type of dancing
Running/jogging
Football/rugby
Badminton/tennis
Squash
Exercises (e.g. press-ups, sit ups)

Question 185

Have you done any other sport or exercise not listed on the card?

Yes
No

Question 186

Can you tell me on how many separate days did you do (name of activity) for at least 15 minutes a time during the past four weeks, that is since (date four weeks ago)?

… hours … minutes

Question 187

How much time did you usually spend doing (name of activity) on each day? (Only count times you did it for at least 15 minutes.)

… hours … minutes

Question 188

During the past four weeks, was the effort of (name of activity) usually enough to make you out of breath or sweaty?

Yes
No

Country:	United Kingdom
Survey:	The Scottish Health Survey
Survey Code:	UK 13
Year:	1998
Type:	Face to Face

Question 178

I'd like you to think about the physical activities you have done in the last few weeks (when you were not doing your paid job.) Have you done any housework in the past four weeks, that is from (date four weeks ago) up to yesterday?

Yes
No

Have you done any housework listed on this card?

SHOW CARD F

Yes
No

Some kinds of housework are heavier than others. This card gives some examples of heavy housework. It does not include everything, these are just examples. Was any of the housework you did in the last four weeks this kind of heavy housework?

SHOW CARD G

Yes
No

During the past four weeks on how many days have you done this kind of heavy housework?

…

On the days you did heavy housework, how long did you usually spend?

…

RECORD MINUTES SPENT ON HEAVY HOUSEWORK.

…

Question 179

Have you done any gardening, DIY or building work in the past four weeks, that is since (date four weeks ago)?

Yes
No

Have you done any gardening, DIY or building work listed on this card?

SHOW CARD H

Yes
No

Have you done any gardening, DIY or building work from this other card, or any similar heavy manual work?

SHOW CARD I

Yes
No

During the past 4 weeks on how many days have you done this kind of heavy manual gardening or DIY?

…

On the days you did heavy manual gardening or DIY, how long did you usually spend?

…

RECORD MINUTES SPENT ON GARDENING OR DIY.

Question 181

Can you tell me if you have done any activities on this card during the last 4 weeks, that is since (date four weeks ago)? Include teaching, coaching, training and practice sessions.

SHOW CARD J

Yes
No

Which have you done in the last four weeks?
PROBE: Any others? CODE ALL THAT APPLY.

Swimming,
Cycling,
Workout at a gym/Exercise bike/ Weight training
Aerobics/Keep fit/Gymnastics/ Dance for fitness
Any other type of dancing
Running/jogging
Football/rugby

Badminton/tennis
Squash
Exercises (e.g. press-ups, sit ups)

Repeat for up to 6 additional sports:

Have you done any other sport or exercise not listed on the card?

Can you tell me on how many separate days did you do (name of activity) for at least 15 minutes a time during the past four weeks, that is since (date four weeks ago)?

How much time did you usually spend doing (name of activity) on each day? (Only count times you did it for at least 15 minutes.)

Question 182

During the past four weeks, was the effort of (name of activity) usually enough to make you out of breath or sweaty?

Yes
No

Child physical activity module (2-15)

IF Age of Respondent is 4 years THEN

Can I just check, is (name of child) at school in reception class yet?

Yes
No

Question 185

I would now like to ask you about any sports or exercise activities that you have done. I will then go on to ask about other active things you may have done like running about, riding a bike, kicking a ball around and things like that.

For the following questions please (include any activities done at a nursery or playgroup/don’t count any activities done as part of school lessons).

In the last week, that is last (day 7 days ago) up to yesterday, have you done any sports or exercise activities (not counting things done as part of school lessons)?

This card shows some of the things you might have done; please also include any other sports or exercise

activities like these.

SHOW CARD L

Yes
No

Question 186

Did you do any of these sports or exercise activities at the weekend, that is last Saturday and Sunday (yesterday and last Sunday)?

Yes
No

Was that on Saturday or Sunday or on both days?

Saturday only
Sunday only
Both Saturday and Sunday

On (Saturday/Sunday/Saturday and Sunday) when you did these sports or exercise activities, how long did you spend (on each day)? Please give an answer from this card.

SHOW CARD K

Less than 5 minutes
5 minutes, less than 15 minutes
15 minutes, less than 30 minutes
30 minutes, less than 1 hour
1 hour, less than 1½ hours
1½ hours, less than 2 hours
2 hours, less than 2½ hours
2½ hours, less than 3 hours
3 hours, less than 3½ hours
3½ hours, less than 4 hours
4 hours or more, please specify how long: … hours
… minutes

Question 187

Still thinking about last week. On how many of the weekdays did you do any of these sports or exercise activities?
(Please remember not to count things done as part of school lessons)

None in last week
1 day
2 days
3 days

4 days
5 days

On each weekday that you did these sports or exercise activities, how long did you spend? Please give an answer from this card.

SHOW CARD K

Less than 5 minutes
5 minutes, less than 15 minutes
15 minutes, less than 30 minutes
30 minutes, less than 1 hour
1 hour, less than 1½ hours
1½ hours, less than 2 hours
2 hours, less than 2½ hours
2½ hours, less than 3 hours
3 hours, less than 3½ hours
3½ hours, less than 4 hours
4 hours or more (please specify how long)

How long did you spend doing these sports or exercise activities on each weekday?

…

Question 188

Now I would like to know about when you do active things, like the things on this card or other activities like these.

Did you do any active things like these at the weekend, that is last Saturday and Sunday (yesterday and last Sunday)?

SHOW CARD M

Yes
No

Was that on Saturday or Sunday or on both days?

Saturday only
Sunday only
Both Saturday and Sunday

On (Saturday/Sunday/Saturday and Sunday) when you did active things like these, how long did you spend (on each day)? Please give an answer from this card.

SHOW CARD K

Less than 5 minutes
5 minutes, less than 15 minutes
15 minutes, less than 30 minutes
30 minutes, less than 1 hour
1 hour, less than 1½ hours
1½ hours, less than 2 hours

2 hours, less than 2½ hours
2½ hours, less than 3 hours
3 hours, less than 3½ hours
3½ hours, less than 4 hours
4 hours or more, please specify how long: … hours
… minutes

Question 189

Still thinking about last week. On how many of the weekdays did you do active things, like the things on this card or other activities like these (not counting things done as part of school lessons)?

SHOW CARD M

None in last week
1 day
2 days
3 days
4 days
5 days

On each weekday that you did active things like these, how long did you spend? Please give an answer from this card.

SHOW CARD D

Less than 5 minutes
5 minutes, less than 15 minutes
15 minutes, less than 30 minutes
30 minutes, less than 1 hour
1 hour, less than 1½ hours
1½ hours, less than 2 hours
2 hours, less than 2½ hours
2½ hours, less than 3 hours
3 hours, less than 3½ hours
3½ hours, less than 4 hours
4 hours or more (please specify how long)

How long did you spend doing active things like these on each weekday?

…

RECORD HERE MINUTES SPENT DOING ACTIVE THINGS LIKE THESE.

Country: Austria

Survey: Health Interview Survey

Survey Code: AT 01

Year: 1999

Type: Face to Face

Question B 42

During your leisure time, do you work up sweat at least once a week as a result of physical exercise, e.g. as a result of jogging, sprint or endurance cycling or aerobics?

If so, how many days a week? Enter number or “no”.

Those in employment: Go on to B 43!

Non-working women with child(ren) up to six years old: Go on to B 44!

Country:	Spain
Survey:	Impairments, Disabilities and Health Status Survey
Survey Code:	ES 04
Year:	1999
Type:	Face to Face

Question 11.26

Specify which type of physical exercise you do regularly in your spare time, that is, which of these possibilities best describes most of your spare time activity.

You do no exercise and spend almost all of your spare time doing sedentary activities (reading, watching television, going to the cinema, etc.)

You do some physical exercise or play sport occasionally (walking or cycling, gardening, leisurely workouts, recreational pursuits involving little exertion, etc.)

You do some physical exercise or play a sport several times a month (tennis, workouts, running, swimming, cycling, team games, etc.)

You do some physical exercise or play a sport several times a week

Country: France

Survey: Health Barometer

Survey Code: FR 06

Year: 1999

Type: Telephone

Question 159

Did you do any sports over the last 7 days ?

Yes
No
DON'T KNOW

If carries out some sports…

Question 160

Which one(s) ?

Athletics (apart from jogging)
Rowing
Badminton
Basket ball
Canoeing-kayaking
Cycling
Dancing
Horse riding
Rock climbing
Football
Golf
Gymnastics
Handball
Jogging
Judo
Karate
Walking (leisure) - Hiking
Body-building
Swimming
Ice skating
Pétanque (bowls)
Windsurfing
Diving
Qi Gong
Rollerblading/Rollerskating
Rugby
Skiing
Surfing
Tai chi chuan

Tennis
Table tennis (ping-pong)
Archery
Volleyball
Mountain biking
Yoga
Other 1, specify: …
Other 2, specify: …
Other 3, specify: …
Other 4, specify: …
Other 5, specify: …
DON'T KNOW
OTHER SPORTS OVER AND ABOVE 5, please
specify:…

Question 167

During the LAST SEVEN DAYS, how many hours…
did you spend in a CLUB ?

… HOURS
… MINUTES
NONE
DON'T KNOW

Athletics (apart from jogging)
Rowing
Badminton
Basket ball
Canoeing-kayaking
Cycling
Dancing
Horse riding
Rock climbing
Football
Golf
Gymnastics
Handball
Jogging
Judo
Karate
Walking (leisure) - Hiking
Body-building
Swimming
Ice skating
Pétanque (bowls)
Windsurfing
Diving
Qi Gong
Rollerblading/Rollerskating
Rugby
Skiing
Surfing
Tai chi chuan
Tennis
Table tennis (ping-pong)
Archery
Volleyball
Mountain biking

Yoga
Other 1, specify: …
Other 2, specify: …
Other 3, specify: …
Other 4, specify: …
Other 5, specify: …

Question 176

During the LAST SEVEN DAYS, how many hours...
were you ALONE OR WITH FRIENDS ? (APART FROM CLUBS, SCHOOL/UNIVERSITY AND WORK)

… HOURS
… MINUTES
NONE
DON'T KNOW

Athletics (apart from jogging)
Rowing
Badminton
Basket ball
Canoeing-kayaking
Cycling
Dancing
Horse riding
Rock climbing
Football
Golf
Gymnastics
Handball
Jogging
Judo
Karate
Walking (leisure) - Hiking
Body-building
Swimming
Ice skating
Pétanque (bowls)
Windsurfing
Diving
Qi Gong
Rollerblading/Rollerskating
Rugby
Skiing
Surfing
Tai chi chuan
Tennis
Table tennis (ping-pong)
Archery
Volleyball
Mountain biking
Yoga
Other 1, specify: …
Other 2, specify: …
Other 3, specify: …
Other 4, specify: …
Other 5, specify: …

Question 179

From amongst the following suggestions, can you generally give me the main reason(s) which most motivates (motivate) you to do sport ?

For pleasure
For health reasons
For slimming purposes
To build up muscles
To meet friends
To win (competitive spirit)
Because you have to (parents, school, family, doctor)
Other reason (s), please specify: ...
DON'T KNOW

Country:	Italy
Survey:	Health Conditions and the Use of Health Services
Survey Code:	IT 01
Year:	1999
Type:	Face to Face

Question 15.1

Over the last 12 months, in your free time, have you done one or more continuous physical activities or sports that required INTENSIVE TRAINING (competitive and non-competitive sports, gym, etc..)?

NO
YES, over 4 hours per week
YES, 4 hours per week
YES, less than 4 hours per week

Question 15.2

Over the last 12 months, in your free time, have you done any physical activity regularly, that made you a little sweaty?(jogging, gym, bike-riding, gardening, etc..)?

NO
YES, over 4 hours per week
YES, 4 hours per week
YES, less than 4 hours per week

Question 15.4

What is the main reason for your doing sports, a exercise or physical activity in your free time?

Therapeutic purposes
To keep in shape
For recreation
For other reasons

Question 15.5

Is your free time usually spent doing a sedentary activity (watching television, reading magazines and/or books, etc..)?

No
Yes, over 4 hours per week
Yes, 4 hours per week
Yes, less than 4 hours per week

Country: Canada

Survey: Canadian Community Health Survey Cycle 1.1

Survey Code: CA 01

Year: 2000

Type: Face to Face

Question PA1

Now I'd like to ask you about some of your physical activities. To begin with, I'll be dealing with physical activities not related to work, that is, leisure time activities.

Have you done any of the following in the past 3 months, that is, from … (date three months ago to yesterday)?
Mark all that apply.

Walking for exercise
Gardening or yard work
Swimming
Bicycling
Popular or social dance
Home exercises
Ice hockey
Ice skating
In-line skating or rollerblading
Jogging or running
Golfing
Exercise class or aerobics
Downhill skiing or snowboarding
Bowling
Baseball or softball
Tennis
Weight-training
Fishing
Volleyball
Basketball
Any other, go to PA1US
No physical activity
DK, R

PA1US What was this activity? (Enter one activity only)

…

Country: Cyprus

Survey: Population Risk Factors Survey

Survey Code: CY 02

Year: 2000

Type: Face to Face

Question 31

In which group do you belong in relation to physical activity?
Including any activity in your leisure time, like physical activity, gardening, dancing etc.

I am not physical active
Light physical activity
Vigorous activity of a minimum of 20 minutes duration for once or twice a week
(sweating, heart beating, out of breath)
Vigorous activity of a minimum of 20 minutes duration for three or more times a Week

---

Question 33

How much physical activity do you have during your leisure-time;
(If it varies with the seasons, mention the group that best represents the average of the year. Mention only one group)

In my leisure time I read, watch television and do things that do not require physical activity.

In my leisure time I walk, ride a bicycle, I go for hunting, I do light gardening for at least 4 hours a week. Going to and coming from work is not included.

In my leisure time I have physical activities to maintain fitness, such as running, gymnastics, swimming, playing football etc.

In my leisure time I do physical activity regularly, because I am involved in physically heavy sports.

Question 34

How many times a week in your leisure time do you do such physical activities that last at least 30 minutes and make you out of breath and sweating?
(If not mark 0)

times a week: ...

Country:	Finland
Survey:	Survey on Health Behaviour
Survey Code:	FI 01
Year:	2000
Type:	Self administered

Question 89

How often in your leisure time do you exercise for at least half an hour so that you are at least slightly out of breath or sweaty?

daily
4 - 6 times a week
2 - 3 times a week
once a week
2 - 3 times a month
a few times a year or less
I cannot exercise due to an illness or injury

Question 91

How much do you exercise and exert yourself physically in your leisure time? If the amount of exertion varies greatly depending on the time of the year, check the alternative that best describes the average situation.

during my leisure time, I read, watch TV and perform chores where I do not need to move much and that do not tire me physically
during my leisure time I walk, bicycle or exercise otherwise at least 4 hours a week
during my leisure time, I do active fitness training at least 3 hours a week on average
during my leisure time I train actively for competitions several times a week

Country:	Finland
Survey:	Health 2000
Survey Code:	FI 03
Year:	2000
Type:	Self administered

Question 27

How much do you exercise and strain yourself physically in your leisure time?
(If there is major seasonal variation, select the option closest to your average situation.)

In my leisure time I read, watch TV and do other activities in which I do not move much and which do not strain me physically
In my leisure time I walk, cycle and move in other ways at least 4 hours per week
In my leisure time I exercise at least 3 hours per week
In my leisure time I practice regularly several times per week for competition (Choose this also if you train full time for competition sports)

Question 28

How often do you exercise in your leisure time so that you are at least slightly out of breath and sweating?

daily
4 - 6 times/week
2 - 3 times/week
once a week
2 - 3 times a month
few times a year or even more rarely

Country:	France
Survey:	Continuous Survey on Households Living Conditions
Survey Code:	FR 07
Year:	2000
Type:	Face to Face

Question A7

Do you carry out any sporting activities (whatever this activity – whether carried out with an organisation or individually)?

Yes, several times a week
Yes, once a week
Yes, twice or three times a month
Yes, once a month
Less than once a month
No, never
Do not know

Country: International

Survey: European Physical Activity Surveillance System (EUPASS)

Survey Code:

Year: 2000

Type: Telephone

Question A3

How often are you engaged in sports or other strenuous activities during your leisure time which causes you to get into a sweat or out of breath. *(Multiple answers are possible)*

without a break

less than 10 minutes
10 till 20 minutes
20 till 30 minutes
30 minutes or more

daily
3 till 6 times per week
1 till 2 times per week
seldom, about once per month
never

Question B5d

How much physical activity did you do in recreation, sport and other leisure-time activities in the last 7 days?

Much
Moderate
Little or no

Country: Italy

Survey: Aspects of daily living

Survey Code: IT 03

Year: 2000

Type: Face to Face

Question 12.3

During your spare time, does it happen to you to practice any type of physical activity such as walking for at least 2 kilometres, swimming, riding a bicycle or other at least a number of times during the year?

NO
Yes, once or more than once a week
Yes, once or more than once a month
Yes, less frequently

Question 12.4

Over the last 12 months, have you practiced sports in places upon payment?

NO
YES

Question 12.5

Over the last 12 months, have you had sports private classes or courses at your or your family's expenses?

NO
YES

Question 12.6

Do you pay a yearly or periodical fee to attend a sports club?

NO
YES

Country: Romania

Survey: Health Interview Survey

Survey Code: RO 01

Year: 2000

Type: Face to Face

Question 108

Which of the following activities describe the best what you have done in your spare time, during the previous 12 months?

Professional sports or intensive training at least once a week
Jogging or other recreation sports, for at least 4 times a week
Walking, riding the bike or other low effort activities, for at least 4 times a week
Reading, watching TV or other sedentary activities

Question 81

Which of the following activities describe best what your child has done in his/her spare time during the passed year?

Professional sports or intensive training – at least once a week
Jogging or other recreation sports – at least 4 hours a week
Walking, riding the bike or other activities involving low physical efforts – at least 4 hours a week
Reading, watching TV or other sedentary activities

Country: United Kingdom

Survey: The Health Survey for England

Survey Code: UK 12

Year: 2000

Type: Face to Face

Question 125

Can you tell me if you have done any activities on this card during the last four weeks, that is since (date four weeks ago)?

Yes
No

Which have you done in the last four weeks?

SHOW CARD L

Swimming
Cycling
Keep fit/other exercises for fitness
Exercises as part of physiotherapy
Dancing
Golf
Yoga
Bowls

Question 126

Have you done any other sport or exercise not listed on the card?

Yes
No

If Yes

…

Question 127

Can you tell me on how many separate days did you do (name of activity) during the past four weeks, that is since (date four weeks ago)?

…

Question 128

How much time did you usually spend doing (name of activity) on each day?

HOURS: …
MINUTES: …

Question 129

During the past four weeks, was the effort of (name of activity) usually enough to make you out of breath or sweaty?

Yes
No

Country: United States

Survey: National Health Interview Survey

Survey Code: US 01

Year: 2000

Type: Face to Face

Question AHB.130

How often do you do physical activities specifically designed to STRENGTHEN your muscles such as lifting weights or doing calisthenics? (Include all such activities even if you have mentioned them before.)

NUMBER:
Never
1-995 times per
Unable to do this type activity
Refused
Don't know

TIME PERIOD:
Day
Week
Month
Year
Unable to do this activity
Refused
Don't know

Country: Australia

Survey: National Health Survey

Survey Code: AU 01

Year: 2001

Type: Face to Face

Question AF207

IN THE LAST 2 WEEKS, HAVE YOU WALKED FOR SPORT, RECREATION OR FITNESS?

Yes
No

Question AF208

HOW MANY TIMES DID YOU WALK IN THE LAST 2 WEEKS?

Number : …

Question AF209

WHAT WAS THE TOTAL AMOUNT OF TIME YOU SPENT WALKING IN THE LAST 2 WEEKS?

Hours/minutes : …
100 hours or more

Country:	Finland
Survey:	Health Behaviour among the Finnish Adult Population
Survey Code:	FI 06
Year:	2001
Type:	Face to Face

Question 79

How often in your leisure time do you exercise for at least half an hour so that you are at least slightly out of breath or sweaty?

daily
4 – 6 times a week
2 – 3 times a week
once a week
2 – 3 times a month
a few times a year or less
I cannot exercise due to an illness or injury

Country: France

Survey: Continuous Survey on Households Living Conditions

Survey Code: FR 11

Year: 2001

Type: Face to Face

Question A7

Do you carry out any sporting activities (whatever this activity – whether carried out with an organisation or individually)?

Yes, several times a week
Yes, once a week
Yes, twice or three times a month
Yes, once a month
Less than once a month
No, never
Do not know

Country:	Iceland
Survey:	Health and Lifestyle of the Icelandic Population
Survey Code:	IS 03
Year:	2001
Type:	Face to Face

Question 23

How often do you practice or do the following, on the average (judging by the part of the year when you are involved in that sport)?

Answer category:
5 or more times/wk
3-4 times/wk
1-2 times/wk
1-3 times/mo.
5-11 times/yr.
Less often
Never

Badminton/tennis/squash
Dancing
Football/handball/basketball
Golf
Hiking (short hikes, e.g. in town)
Riding
Cycling
Yoga
Bowling
Training at exercise centre
Skiing inc. cross-country/snowboarding
Jogging/long-distance running
Angling/hunting
Swimming
Other, specify: …

Country: Italy

Survey: Aspects of Daily Living

Survey Code: IT 04

Year: 2001

Type: Face to Face

Question 12.3

During your spare time, does it happen to you to practice any type of physical activity such as walking for at least 2 kilometres, swimming, riding a bicycle or other at least a number of times during the year?

NO
Yes, once or more than once a week
Yes, once or more than once a month
Yes, less frequently

Question 12.4

Over the last 12 months, have you practiced sports in places upon payment?

NO
YES

Question 12.5

Over the last 12 months, have you had sports private classes or courses at your or your family's expenses?

NO
Yes

Question 12.6

Do you pay a yearly or periodical fee to attend a sports club?

NO
Yes

Country: Netherlands

Survey: Patient survey - Second Dutch National Survey of General Practice

Survey Code: NL 02

Year: 2001

Type: Face to Face

Question LRV44g

How many different kinds of sports do you play in an average week?
(For example: tennis, gymnastics, rollerblading, swimming)

…

Question LRV44h0

Which sport do you play most?

...

Question LRV44h1

How many days a week do you play it?

...

Question LRV44h2

And on average how many hours and minutes a day?

Number of hours: ...

Question LRV44h3

Number of minutes: …

Question LRV44i0

Which sport do you play most often after that?

...

Question LRV44i1

How many days a week?

...

Question LRV44i2

And on average how many hours and minutes per day?

Number of hours: …

Question LRV44i3

Number of minutes: …

Country: Slovenia

Survey: Slovenian Public Health Opinion - SJM01/3

Survey Code: SI 02

Year: 2001

Type: Face to Face

Question 1.01

DO YOU GO IN FOR SPORT OR ANY PHYSICAL RECREATION?

Yes
No

If yes, HOW OFTEN?

once a week
2 to 3 times a week
4 to 6 times a week
each day

Country:	Switzerland
Survey:	Swiss Health Survey 2002
Survey Code:	CH 02
Year:	2002
Type:	Telephone

Question 44.00

During your free time, do you take physical activity at
least once a week which makes you perspire?
(such as running, cycling, fast walking)

yes
no
no reply

Question 44.01

How many days a week on average?

… days a week (between 1 and 7 days)
no reply

Country: Czech Republic

Survey: Sample Survey of the Health Status of the Czech Population

Survey Code: CZ 01

Year: 2002

Type: Face to Face

Question 24a_1

Leisure time physical activity

During the past 7 days, on how many days did you do hard training and competitive sport [more than 10 minutes at a time]?

0-7

What is the total amount of time you spent over the past 7 days doing hard training and competitive sport [how many hours]?

...

Question 24a_2

Leisure time physical activity

During the past 7 days, on how many days did you do jogging and other recreational sports or heavy gardening and work in household [e.g. decorate the flat, more than 10 minutes at a time]?

0-7

What is the total amount of time you spent over the past 7 days doing jogging and other recreational sports or heavy gardening and work in household [how many hours]?

...

Question 24a_3

Leisure time physical activity

During the past 7 days, on how many days did you do walking, bicycling or other light activities [e.g. shopping, clean up the flat, more than 10 minutes at a time?

0-7

What is the total amount of time you spent over the past 7 days doing walking, bicycling or other light activities [how many hours]?

Question 24e_4

Leisure time physical activity

During the past 7 days, on how many days did you do reading, watching TV or other sedentary activities [more than 10 minutes at a time]?

0-7

What is the total amount of time you spent over the past 7 days doing reading, watching TV or other sedentary activities [how many hours]?

Country:	Finland
Survey:	FINRISK 2002
Survey Code:	FI 07
Year:	2002
Type:	Self administered

Question 74

How much do you exercise and stress yourself physically in your leisure time? If it varies much according to different seasons, mark the alternative which best describes the average situation.

In my leisure time I read, watch TV, and work in the household with tasks which do not make me move much and which do not physically tax me.

In my spare time I walk, cycle or exercise otherwise at least 4 hours per week. This includes walking, fishing and hunting, light gardening etc. but excludes travel to work.

In my spare time I exercise to maintain my physical condition, e.g. running, jogging, skiing, gymnastics, swimming, playing ball games or I do heavy gardening or the like for at least 3 hours per week.

In my spare time I regularly exercise several times a week competitive sports such as running, orienteering, skiing, swimming, playing ball games or other heavy sports.

Question 76

How often do you in your leisure time exercise for at least 20-30 minutes so that you at least are mildly out of breath and sweaty?

daily
2-3 times a week
once a week
2-3 times a month
a few times a year or more seldom
I have a disability or a disease which does not enable me to exercise (proceed to question 79)

Question 77

How many times in a week do you exercise in your free time so that you at least are mildly out of breath and sweaty? (if not at all, mark 0.)

… times a week

Question 78

How long does your usual leisure time activity take?

I do not exercise in my free time
less than 15 minutes
15 - 29 minutes
30 - 59 minutes
one hour or longer

Question 79

How many minutes do you daily walk, cycle or engage in a hobby in your leisure time that requires moving about (yard work or gardening, fixing or cleaning the house)? Do not count in the activity needed at work, travelling to work (question 75) or leisure time sports (questions 76-78).

less than 15 minutes per day
15-29 minutes per day
30-44 minutes daily
45-59 minutes daily
over an hour per day

Country:	Finland
Survey:	Health Behaviour among the Finnish Adult Population
Survey Code:	FI 13
Year:	2002
Type:	Face to Face

Question 81

How often in your leisure time do you exercise for at least half an hour so that you are at least slightly out of breath or sweaty?

daily
4 – 6 times a week
2 – 3 times a week
once a week
2 – 3 times a month
a few times a year or less
I cannot exercise due to an illness or injury

Country:	Ireland
Survey:	Survey of Lifestyle, Attitudes and Nutrition (SLAN)
Survey Code:	IE 03
Year:	2002
Type:	Face to Face

Question B1

Considering a 7-day period (a week), how many times on average do you do the following kinds of exercise for more than 20 minutes during your free time?
(Please write the appropriate number on each line)

a) STRENUOUS EXERCISE (HEART BEATS RAPIDLY)
(e.g. running, jogging, hurling, camogie, football, soccer, squash, basketball, judo, roller skating, vigorous swimming, vigorous long distance cycling, advanced aerobics)

Times per Week: …

b) MODERATE EXERCISE (NOT EXHAUSTING)
(e.g. fast walking, tennis, badminton, easy swimming, easy cycling, popular and folk dancing, intermediate aerobics, heavy gardening)

Times per Week: …

c) MILD EXERCISE (MINIMAL EFFORT)
(e.g. yoga, golf, easy walking, fishing from river bank, bowling, beginners aerobics, archery, light gardening)

Times per Week: …

d) LITTLE / NO ACTIVITIES

Times per Week: …

Question B3

Do you attend a Gym/Leisure Centre?

Yes
No

Country: Italy

Survey: Aspects of Daily Living

Survey Code: IT 05

Year: 2002

Type: Face to Face

Question 12.3

During your spare time, does it happen to you to practice any type of physical activity such as walking for at least 2 kilometres, swimming, riding a bicycle or other at least a number of times during the year?

NO
Yes, once or more than once a week
Yes, once or more than once a month
Yes, less frequently

Question 12.4

Over the last 12 months, have you practiced sports in places upon payment?

NO
YES

Question 12.5

Over the last 12 months, have you had sports private classes or courses at your or your family's expenses?

NO
YES

Question 12.6

Do you pay a yearly or periodical fee to attend a sports club?

NO
YES

Country:	Norway
Survey:	Survey on living conditions & health, care and social relations
Survey Code:	NO 02
Year:	2002
Type:	Face to Face

Question H32a

Questions regarding your physical activity in your free time during the past 12 months follow. Use a normal week as your basis and include travel to and from work as free time.

How many hours per week were you engaged in hard physical activity (no sweating/heavy breathing)?

Not active
Less than 1 hour
1-2 hours a week
3 or more hours a week

Question H32b

How many hours per week were you engaged in hard physical activity (sweating/heavy breathing)?

Not active
Less than 1 hour
1-2 hours a week
3 or more hours a week

Question H33

Do you usually exercise…

Never
Less than once a week
Once a week or more

Question H33a

How many days do you exercise during an average week…

NUMBER OF DAYS: …

Country: Norway

Survey: Cohort Norway

Survey Code: NO 03

Year: 2002

Type: Self administered

Question 5a

How has your physical activity during leisure time been over the last year? Think of your weekly average for the year. Time spent going to or from work counts as leisure time

Hours per week
None
Less than 1
1-2
3 or more

Light activity (not sweating or out of breath)

Hard physical activity (sweating/out of breath)

Question 5b

Please note physical activity during the past year in your spare time. If activity varies between summer and wintertime, note a mean value. (Tick one only)

Reading, watching TV or any other sedentary activity?

Walking, cycling, or other activity, other for at least 4 hours a week?
(Count also walking back and forth from work)

Light sports, heavy gardening? (At least 4 hours per week)

Hard exercise, competitive sports? Regularly and several times a week

Country:	Slovakia
Survey:	Health Monitor Survey
Survey Code:	SK 02
Year:	2002
Type:	Face to Face

Question 6.12

In your leisure time, how often do you do physical exercise for at least 30 minutes which makes you at least mildly short of breath or perspire?

daily
4-6 times a week
2-3 times a week
once a week
2-3 times a month
a few times a year or less
I cannot exercise because of illness
I cannot exercise because of disability

Country:	United Kingdom
Survey:	General Household Survey
Survey Code:	UK 07
Year:	2002
Type:	Face to Face

Question 5 Tuition

Over the past twelve months have you received tuition
from an instructor or coach to improve your
performance in (activity)?

Yes
No
Not sure

Question 7 Sport

Did you take part in (activity) in the past four weeks?
Again, please do not count any teaching coaching or
refereeing you may have done.

Yes
No

Question 8 Sprtime

On how many days in the last four weeks have you
played/gone to (activity).

Question 9 Spwhere

At which of these places on this card have you
done/played (activity) in the last four weeks ?

INDIVIDUAL PROMPT. CODE ALL THAT APPLY

Indoors at a facility which is mainly used for sport (e.g
sports centre or gymnasium or indoor swimming
pool or commercial leisure facility)

Indoors at some other location not mainly used for
sport (such as a community centre, village hall or
scout hut

Indoors or outdoors at home or someone else's home

Outdoors on a court, course, pitch or playing field (or outdoor swimming pool)

Outdoors in a natural setting (such as the countryside, rivers, lakes or seaside)

Other - including roads and pathways in towns and Cities

Question 10 SchlCol

Do any of these facilities belong to a school, college or university?

Yes
No

Question 11 SpClub

Over the past four weeks have you been a member of a club, particularly so that you can play/participate in (activity)?

Yes
No

Question 12 ClubTyp

What type of club was this?
(ENTER AT MOST 4 CODES)

Health/fitness
Social club (e.g employee clubs, youth clubs)
Sports club
Other

Question 13 Volunt

Looking back over the last four weeks, have you spent any time helping to organise sport on a voluntary basis (that is, without pay except for expenses)? Please include any teaching, coaching or refereeing you may have done as a volunteer.

Yes
No

Question 14 Voltime

During the last four weeks, how many hours in total have you spent on voluntary sports work?

Less than 1 hour
1 hour less than 2 hours
2 hours less than 5 hours
5 hours or more

Question 15 Sprtnot

Is there any sport or recreational activity that you do not do at the moment but would like to do?

Yes
No

Question 16 Sprtwch

Which one activity would you like to do?

…

Country: United Kingdom

Survey: Health Survey for England

Survey Code: UK 28

Year: 2002

Type: Face to Face

Question 153

I would now like to ask you about any sports or exercise activities that you have/(name of child) has done. I will then go on to ask about other active things you/s/he may have done like running about, riding a bike, kicking a ball around and things like that.
For the following questions please include any activities done at a nursery or playgroup/don't count any activities done as part of school lessons.

In the last week, that is last (date last week) up to yesterday, have you/has (name of child) done any sports or exercise activities, not counting things done as part of school lessons?/?

This card shows some of the things you/s/he might have done; please also include any other sports or exercise activities like these.

Yes
No

Question 154a

Did you/(name of person) do any of these sports or exercise activities at the weekend, that is last Saturday and Sunday/that is yesterday and last Sunday?

Yes
No

Question 154b

IF Yes, Was that on Saturday or Sunday or on both days?

Saturday only
Sunday only
Both Saturday and Sunday

Question 155

On Saturday/Sunday/Saturday and Sunday when you/(name of child) did these sports or exercise activities, how long did you/s/he spend on each day/?

Please give an answer from this card.

15 minutes, less than 30 minutes
30 minutes, less than 1 hour
1 hour, less than 1 1/2 hours
1 1/2 hours, less than 2 hours
2 hours, less than 2 1/2 hours
2 1/2 hours, less than 3 hours
3 hours, less than 3 1/2 hours
3 1/2 hours, less than 4 hours
4 hours or more,(please specify how long: How long did you/ (name of child) spend doing these sports or exercise activities?

hours :… minutes: ….

Question 156

Still thinking about last week. On how many of the weekdays did you/ (name of child) do any of these sports or exercise activities?
Please remember not to count things done as part of school lessons.

None in last week
1 day
2 days
3 days
4 days
5 days

Question 157

On each weekday that you/(name of child) did these sports or exercise activities, how long did you/s/he spend?

Please give an answer from this card.

15 minutes, less than 30 minutes
30 minutes, less than 1 hour
1 hour, less than 1 1/2 hours
1 1/2 hours, less than 2 hours
2 hours, less than 2 1/2 hours
2 1/2 hours, less than 3 hours
3 hours, less than 3 1/2 hours
3 1/2 hours, less than 4 hours
4 hours or more,(please specify how long: How long did you/(name of child) spend doing these sports or exercise activities?

hours :… minutes: ….

Question 158

Now I would like to know about when you do/(name of child) does active things, like the things on this card or other activities like these.

Did you/s/he do any active things like these at the weekend, that is last Saturday and Sunday/that is yesterday and last Sunday?

Yes
No

Question 159

Was that on Saturday or Sunday or on both days?

Saturday only
Sunday only
Both Saturday and Sunday

Question 160

On Saturday/Sunday/Saturday and Sunday when you/(name of child) did active things like these, how long did you/s/he spend on each day/?

15 minutes, less than 30 minutes
30 minutes, less than 1 hour
1 hour, less than 1 1/2 hours
1 1/2 hours, less than 2 hours
2 hours, less than 2 1/2 hours
2 1/2 hours, less than 3 hours
3 hours, less than 3 1/2 hours
3 1/2 hours, less than 4 hours
4 hours or more,(please specify how long: How long did you/(name of child) spend doing active things like these?

hours :… minutes: ….

Question 161

Still thinking about last week. On how many of the weekdays did you/(name of child) do active things on this card or other activities like these not counting things done as part of school lessons?

None in last week
1 day
2 days
3 days
4 days
5 days

Question 162

On each weekday that you/(name of child) did active things like these, how long did you/s/he spend? Please give an answer from this card.

15 minutes, less than 30 minutes
30 minutes, less than 1 hour
1 hour, less than 1 1/2 hours
1 1/2 hours, less than 2 hours
2 hours, less than 2 1/2 hours
2 1/2 hours, less than 3 hours
3 hours, less than 3 1/2 hours
3 1/2 hours, less than 4 hours
4 hours or more, please specify how long: How long did you/(name of child) spend doing active things like these on each weekday?

Question 164

Were the activities you/(name of child) did last week different from what you/s/he would usually do for any reason?

No - same as usual
Yes different - usually do more
Yes different - usually do less

Question 169a

Have you done any gardening, Do It Yourself or building work in the past four weeks, that is since (date four weeks ago)?

Yes
No

Question 169b

IF Yes, Have you done any gardening, Do It Yourself or building work listed on this card?

Yes
No

Question 170a

Have you done any gardening, Do It Yourself or building work from this other card, or any similar heavy manual work?

Yes
No

Question 170b

IF Yes, during the past four weeks on how many days have you done this kind of heavy manual gardening or Do It Yourself, for at least 30 minutes a time?

… days

Question 173a

Can you tell me if you have done any activities on this card during the last four weeks, that is since (date four weeks ago)? Include teaching, coaching, training and practice sessions.

Yes
No

Question 173b

IF Yes, which have you done in the last four weeks?

Swimming
Cycling
Workout at a gym/Exercise bike/ Weight training
Aerobics/Keep fit/Gymnastics/ Dance for fitness
Any other type of dancing
Running/jogging
Football/rugby
Badminton/tennis
Squash
Exercises (e.g. press-ups, sit ups)

Question 173c

Have you done any other sport or exercise not listed on the card?

Yes
No

Question 173d

IF Yes: ……………………….

Question 174

Can you tell me on how many separate days did you do (activity) for

at least 15 minutes a time during the past four weeks, that is since (date four weeks ago)?

… days

Question 175

How much time did you usually spend doing (activity) on each day?
(Only count times you did it for at least 15 minutes.)

Hours: … Minutes: …

Question 176

During the past four weeks, was the effort of (activity) usually enough to make you out of breath or sweaty?

Yes
No

Country:	Canada
Survey:	Canadian Community Health Survey Cycle 2.1
Survey Code:	CA 02
Year:	2003
Type:	Face to Face + Telephone

Question PAC_Q1

Have you done any of the following in the past 3 months, that is, from [date three months ago] to yesterday?

Walking for exercise
Gardening or yard work
Swimming
Bicycling
Popular or social dance
Home exercises
Ice hockey
Ice skating
In-line skating or rollerblading
Jogging or running
Golfing
Exercise class or aerobics
Downhill skiing or snowboarding
Bowling
Baseball or softball
Tennis
Weight-training
Fishing
Volleyball
Basketball
Soccer
Any other
No physical activity

Question PAC_Q1VS

What was this activity?

...

Question PAC_Q1X

In the past 3 months, did you do any other physical activity for leisure?

Yes
No

Question PAC_Q1XS

What was this activity?

...

Question PAC_Q1Y

In the past 3 months, did you do any other physical activity for leisure?

Yes
No

Question PAC_Q1YS

What was this activity?

...

Question PAC_Q2n

In the past 3 months, how many times did you [participate in identified activity]?

... Times
(MIN: 1) (MAX: 99 for each activity except the following: Walking: MAX = 270
Bicycling: MAX = 200 Other activities: MAX = 200)

Question PAC_Q3n

About how much time did you spend on each occasion?

1 to 15 minutes
16 to 30 minutes
31 to 60 minutes
More than one hour

Question UPE_Q2A

When in-line skating or rollerblading, how often do you wear a helmet?

Always
Most of the time
Rarely
Never

Question UPE_Q2B

How often do you wear wrist guards or wrist protectors?

Always
Most of the time
Rarely
Never

Question UPE_Q2C

How often do you wear elbow pads?

Always
Most of the time
Rarely
Never

Question UPE_Q3A

Earlier, you mentioned going downhill skiing or snowboarding in the past 3 months.

Was that:

… downhill skiing only?
… snowboarding only?
… both ?

Question UPE_Q3B

In the past 12 months, did you do any downhill skiing or snowboarding?

Downhill skiing only
Snowboarding only
Both
Neither

Question UPE_Q4A

When downhill skiing, how often do you wear a helmet?

Always
Most of the time
Rarely
Never

Question UPE_Q5A

When snowboarding, how often do you wear a helmet?

Always
Most of the time
Rarely
Never

Question UPE_Q5B

How often do you wear wrist guards or wrist protectors?

Always
Most of the time
Rarely
Never

Question UPE_Q6

In the past 12 months, have you done any skateboarding?

Yes
No

Question UPE_Q6A

How often do you wear a helmet?

Always
Most of the time
Rarely
Never

Question UPE_Q6B

How often do you wear wrist guards or wrist protectors?

Always
Most of the time
Rarely
Never

Question UPE_Q6C

How often do you wear elbow pads?

Always
Most of the time
Rarely
Never

Question_STR_Q6_6

How often do you jog or do other exercise to deal with stress?

Often
Sometimes
Rarely
Never

Question ETA_Q13

You think about burning up calories when you exercise.

Always
Usually
Often
Sometimes
Rarely
Never

Country:	Cyprus
Survey:	Health Survey 2003
Survey Code:	CY 01
Year:	2003
Type:	Face to Face

Question 17

Do you do any of the following activities in your leisure time?

Hard training and competitive sports more than once a week
Jogging and other recreational sports or heavy gardening at least 4 hours a week
Walking, bicycling or other light activities at least 4 hours a week
Reading, watching TV or other sedentary activities

Country: Spain

Survey: National Health Survey 2003

Survey Code: ES 03

Year: 2003

Type: Face to Face

Question 32

Which of the following possibilities best describes the frequency with which you carry out any physical activity in your free time?

Do not carry out any physical activity whatsoever

Carry out some type of physical or sporting activity less than once a month

Carry out some type of physical or sporting activity once or various times a month, but less than once a week

Carry out some type of physical or sporting activity once or various times a week

---

Question 25

Which of the following possibilities best describes the frequency with which the child carries out any physical activity in their free time?

They do not carry out any physical activity whatsoever

They carry out some type of physical or sporting activity less than once a month

They carry out some type of physical or sporting activity once or various times a month but less than once a week

They carry out some type of physical or sporting activity once or various times a Week

Country: Finland

Survey: Health Behaviour among the Finnish Adult Population

Survey Code: FI 10

Year: 2003

Type: Face to Face

Question 84

How often in your leisure time do you exercise for at least half an hour so that you are at least slightly out of breath or sweaty?

daily
4–6 times a week
2–3 times a week
once a week
2–3 times a month
a few times a year or less
I cannot exercise due to an illness or injury

Question 86

How much do you exercise and exert yourself physically in your leisure time? If the amount of exertion varies greatly depending on the time of the year, check the alternative that best describes the average situation.

during my leisure time, I read, watch TV and perform chores where I do not need to move much and that do not tire me physically

during my leisure time I walk, bicycle or exercise otherwise at least 4 hours a week
during my leisure time, I do active fitness training at least 3 hours a week on average
during my leisure time, I train actively for competitions several times a week

Country:	Croatia
Survey:	Croatian Health Survey
Survey Code:	HR 04
Year:	2003
Type:	Face to Face

Question PHA 03

In your leisure time, how often do you do physical exercise for at least 30 minutes which makes you at least mildly short of breath or perspire?

Not physically active
Several times a year
2 to 3 times a month
Once a week
2 to 3 times a week
4 to 6 times a week
Every day

Country: International

Survey: Joint Canada/United States Survey of Health (JCUSH)

Survey Code: INT 12

Year: 2003

Type: Telephone

Question PAC_Q01

Now I'd like to ask you about some of your physical activities. To begin with, I'll be dealing with physical activities not related to work, that is, leisure time activities.

Have you done any of the following in the past 3 months, that is, from <date three months ago> to yesterday?

Walking for exercise
Gardening or yard work
Swimming
Bicycling
Popular or social dance
Home exercises
Ice hockey
Ice skating
In-line skating or rollerblading
Jogging or running
Golfing
Exercise class or aerobics
Downhill skiing
Bowling
Baseball or softball
Tennis
Weight-training
Fishing
Volleyball
Basketball
Soccer
Any other
No physical activity

What was this activity?

...

Question PAC_Q02

In the past 3 months, how many times did you participate in <identified activity>?

... Times (MIN: 1) (MAX: 99 for each activity except the following:
Walking: MAX = 270
Bicycling: MAX = 200
Other activities: MAX = 200)

Question PAC_Q03

About how much time did you spend on each occasion?

1 to 15 minutes
16 to 30 minutes
31 to 60 minutes
More than one hour

Country: Italy

Survey: Aspects of Daily Living

Survey Code: IT 07

Year: 2003

Type: Face to Face + self administered

Question 12.3

(If you do not practice any sport, otherwise go to question 12.4)

During your spare time, does it happen to you to practice any type of physical activity such as walking for at least 2 kilometres, swimming, riding a bicycle or other at least a number of times during the year?

NO
YES, once or more than once a week
YES, once or more than once a month
YES, less frequently

Question 12.4

(If you practice sport regularly or occasionally, otherwise go to question 13.1)

Over the last 12 months, have you practiced sports in places upon payment?

NO
YES

Question 12.5

Over the last 12 months, have you had sports private classes or courses at your or your family's expenses?

NO
YES

Question 12.6

Do you pay a yearly or periodical fee to attend a sports club?

NO
YES

Country:	Sweden
Survey:	Living Condition Survey
Survey Code:	SE 03
Year:	2003
Type:	Face to Face

Question 63 B

I would now like to ask about how much exercise you get in your leisure time.
Which of these alternatives fits you best?

Practically no exercise at all
Some exercise now and then (e.g. short walks)
Exercise regularly about once a week
Exercise regularly about twice a week
Exercise regularly and fairly vigorously at least twice a week

Country: Australia

Survey: National Health Survey

Survey Code: AU 03

Year: 2004

Type: Face to Face

Question 4

The next few questions are about walking for sport, recreation and fitness.

Please do not include any other walking that you may have done for other reasons. This will be recorded later.

In the last 2 weeks have you walked for sports, recreation or fitness?

Yes
No

Question 5

How many times did you walk for sports, recreation or fitness in the last 2 weeks?

...
Don't know

Question 6

What was the total amount of time you walked for sports, recreation or fitness in the last 2 weeks?

Time in minutes: ...
Don't know

Time in hours: ...
Don't know

Time in hours/minutes: ...
Don't know

Question 7

The next few questions are about moderate and vigorous exercise apart from walking that you may have done for exercise, sport or fitness. I will ask about moderate exercise first, then about vigorous exercise.

In the last 2 weeks did you do any exercise which caused a moderate increase in your heart rate or breathing, that is, moderate exercise?

Yes
No

Question 8

How many times did you do any moderate exercise in the last 2 weeks?

...
Don't know

Question 9

What was the total amount of time you spent doing moderate exercise in the last 2 weeks?

Time in minutes: ...
Don't know

Time in hours: ...
Don't know

Time in hours: ...
Don't know

Question 10

In the last 2 weeks did you do any other exercise which caused a large increase in your heart rate or breathing, that is, vigorous exercise?

Yes
No

Question 11

How many times did you do any vigorous exercise in the last 2 weeks?

...
Don't know

Question 12

What was the total amount of time you spent doing vigorous exercise in the last 2 weeks?

Time in minutes: ...
Don't know

Time in hours: ...
Don't know

Time in hours: ...
Don't know

Country: Estonia

Survey: Health behaviour among Estonian adult population

Survey Code: EE 07

Year: 2004

Type: Self administered

Question 72

In you leisure time, how often do you do physical exercises (Sunday sports) that makes you at least mildly short of breath or perspire for at least 30 minutes?

daily
4–6 times a week
2–3 times a week
once a week
2–3 times a month
a few times a year or less
I cannot exercise because of damage or illness

Country: Finland

Survey: Health Behaviour among the Finnish Adult Population

Survey Code: FI 12

Year: 2004

Type: Face to Face

Question 74

How often in your leisure time do you exercise for at least half an hour so that you are at least slightly out of breath or sweaty?

daily
4–6 times a week
2–3 times a week
once a week
2–3 times a month
a few times a year or less
I cannot exercise due to an illness or injury

Question 76

How much do you exercise and exert yourself physically in your leisure time? If the amount of exertion varies greatly depending on the time of the year, check the alternative that best describes the average situation.

during my leisure time, I read, watch TV and perform chores where I do not need to move much and that do not tire me physically

during my leisure time I walk, bicycle or exercise otherwise at least 4 hours a week

during my leisure time, I do active fitness training at least 3 hours a week on average

during my leisure time, I train actively for competitions several times a week

Country: Greece

Survey: National Survey: Psychosocial factors and Health

Survey Code: GR 05

Year: 2004

Type: Face to Face

Question EP27

During a regular week, how many days on the whole do you physical exercise?

None
1 day
2 days
3 days
4 days
5 days
6 days
7 days
Do not answer

Question EP28

For how many minutes do you exercise?

...
Do not answer

Country: International (Finland, Baltic States)

Survey: FINBALT Health Monitor

Survey Code:

Year: 2004

Type: Self administered

Question 56

In you leisure time, how often do you do physical exercise for at least 30 min. which makes you at least mildly short of breath or perspire?

1 daily
2 4-6 times a week
3 2-3 times a week
4 once a week
5 2-3 times a month
6 a few times a year or less
7 I cannot exercise because of an illness
8 I cannot exercise because of disability

Country: Poland

Survey: Health Population Status

Survey Code: PL 06

Year: 2004

Type: Face to Face

Question 88

Do you perform the following activities in your free time?

Do you perform these activities?

Yes
No

If yes , how many hours per week, on the average?

Types of activities:

Reading, watching television, listening to the radio or other activities not requiring movement and physical strain

Walks, gymnastics, bike riding, roller blades or other non-intensive physical activity

Running, swimming, aerobics or other recreational sport, seasonal working in the garden

Intensive physical workout (for ex. weight room), sport

Question 58

Does the child regularly participate in organized sport trainings (in -school after lessons, at a sporting club, at the pool)?

Yes
No

Question 59

Please, give the number of hours per week

...

Country: Sweden

Survey: Survey of Living Conditions

Survey Code: SE 04

Year: 2004

Type: Face to Face

Question 60c

I would now like to ask about how much exercise you get in your leisure time.
Which of these alternatives fit you best?

Practically no exercise at all
Some exercise now and then (e.g. short walks)
Exercise regularly about once a week
Exercise regularly about twice a week
Exercise regularly and fairly vigorously at least twice a week

Country: United States

Survey: National Health Interview Survey

Survey Code: US 02

Year: 2004

Type: Face to Face + Telephone

Question AHB.090

The next questions are about physical activities (exercise, sports, physically active hobbies...) that you may do in your LEISURE time.

How often do you do VIGOROUS leisure-time physical activities for AT LEAST 10 MINUTES that cause HEAVY sweating or LARGE increases in breathing or heart rate?

* Read if necessary: How many times per day, per week, per month, or per year do you do these activities?
* Enter number for vigorous leisure-time physical activities.
* Enter '0' for Never.
* Enter '996' if unable to do this type of activity.

Never
1-995 time(s)
Unable to do this type activity
Refused
Don't know

* Enter time period for vigorous leisure-time physical activities.

Never
Per day
Per week
Per month
Per year
Unable to do this activity
Refused
Don't know

Question AHB.100

About how long do you do these vigorous leisure-time physical activities each time?

* Enter number for length of vigorous leisure-time physical activities.

1-995
Refused
Don't know

* Enter time period for length of vigorous leisure-time physical activities.

Minutes
Hours
Refused
Don't know

Question AHB.110

How often do you do LIGHT OR MODERATE LEISURE-TIME physical activities for AT LEAST 10 MINUTES that cause ONLY LIGHT sweating or a SLIGHT to MODERATE increase in breathing or heart rate?

* If necessary, prompt with: How many times per day, per week, per month, or per year do you do these activities?
* Enter number of light or moderate leisure-time physical activities.
* Enter '0' for Never.
* Enter '996' if unable to do this type of activity.

Never
1-995 time(s)
Unable to do this type activity
Refused
Don't know

* Enter time period for light or moderate leisure-time physical activities

Never
Per day
Per week
Per month
Per year
Unable to do this activity
Refused
Don't know

Question AHB.120

About how long do you do these light or moderate leisure-time physical activities each time?

* Enter number for length of light or moderate leisure-time physical activities.

1-995
Refused
Don't know

* Enter time period for length of light or moderate leisure-time physical activities.

Minutes
Hours
Refused
Don't know

Question AHB.130

How often do you do LEISURE-TIME physical activities specifically designed to STRENGTHEN your muscles such as lifting weights or doing calisthenics?
(Include all such activities even if you have mentioned them before.)

* Read if necessary: How many times per day, per week, per month, or per year do you do these activities?
* Enter number for strengthening activities.
* Enter '0' for Never.
* Enter '996' for Unable to do this type activity

Never
1-995 time(s)
Unable to do this type activity
Refused
Don't know

* Enter time period for strengthening activities

Never
Per day
Per week
Per month
Per year
Unable to do this activity
Refused
Don't know

Country:	Canada
Survey:	National Population Health Survey (NPHS)
Survey Code:	CA 03
Year:	2005
Type:	Face to Face + Telephone

Question PA_Q1

Now I'd like to ask you about some of your physical activities. To begin with, I'll be dealing with physical activities not related to work, that is, leisure time activities.

Have you done any of the following in the past 3 months, that is, from [date three months ago] to yesterday?

Walking for exercise
Gardening or yard work
Swimming
Bicycling
Popular or social dance
Home exercises
Ice hockey
Ice skating
In-line skating or rollerblading
Jogging or running
Golfing
Exercise class or aerobics
Downhill skiing or snowboarding
Bowling
Baseball or softball
Tennis
Weight-training
Fishing
Volleyball
Basketball
Any other
No physical activity
DK, R

Question HS_Q1US

What was this activity?

...

Question PA_Q1W

In the past 3 months, did you do any other activity for leisure?

Yes
No
DK, R

What was this activity?

...

Question PA_Q1X

In the past 3 months, did you do any other activity for leisure?

Yes
No
DK, R

What was this activity?

...

Question PA_Q2

In the past 3 months, how many times did you participate in [identified activity]?

... Times

(MIN: 1) (MAX: 99) for each activity except the following:
Walking: MAX = 270
Bicycling: MAX = 200
Other activities: MAX = 200
DK, R

Question PA_Q3

About how much time did you spend on each occasion?

1 to 15 minutes
16 to 30 minutes
31 to 60 minutes
More than one hour

Country: Germany

Survey: Telephone Health Survey 2004-2005

Survey Code: DE 07

Year: 2005

Type: Telephone

Question 231

Thinking back over the last 3 months, have you done any sport during that time?

yes
no
d.k.
n.a.

Question 232

... and how many hours in the weeks was it?

Was it ...

up to an hour
up to two hours
up to four hours
more than four hours
d.k.
n.a./answer refused

Country: Denmark

Survey: The Danish Health Interview Survey

Survey Code: DK 03

Year: 2005

Type: Face to Face

Question 24

Which types of exercise training or sports have you participated in during the past year?

...
Have not participated in exercise training or sports

Question 24a

If you have participated in exercise training or sports during the past year:

What are the two most important reasons that you participate in exercise training or sports?

To stay in shape, get in shape
For the sake of my appearance
To be together with other people
To lose weight
To have fun
To unwind
Other, write here: ...

Country: Finland

Survey: Health Behaviour among the Finnish Adult Population

Survey Code: FI 19

Year: 2005

Type: Self administered

Question 79

How often in your leisure time do you exercise for at least half an hour so that you are at least slightly out of breath or sweaty?

daily
4 – 6 times a week
2 – 3 times a week
once a week
2 – 3 times a month
a few times a year or less
I cannot exercise due to an illness or injury

Question 81

How much do you exercise and exert yourself physically in your leisure time? If the amount of exertion varies greatly depending on the time of the year, check the alternative that best describes the average situation.

during my leisure time I read, watch TV and perform chores where I do not need to move much and that do not tire me physically

during my leisure time I walk, bicycle or exercise otherwise at least 4 hours a week

during my leisure time I do active fitness training at least 3 hours a week on average

during my leisure time I train actively for competitions several times a week

---

Question 101

Are you a member in any society or organization?

no
yes

health and/or patient-organization
gymnastics and/or sports association
other organization

Country:	Italy
Survey:	Health Conditions and the Use of Health Services 2004-2005
Survey Code:	IT 08
Year:	2005
Type:	Face to Face

Question 17.1

During your free-time, do you practice, at least once a week:
(Read all answers and fill for each row)

Physical activity with heavy physical effort (sport at competitive or not competitive level, cycling, jogging, etc)
Moderate physical activity, that is until perspiring (gym, moderate speed bicycle rides, etc)
Light physical activity (at least 1 Km walks, light gym , etc)

No

Yes

(If yes)

How many days a week: ...

How long every time on average

Less than 20 minutes
20 minutes and more, specify in minutes: …

Country: Italy

Survey: Aspects of Daily Living

Survey Code: IT 09

Year: 2005

Type: Face to Face + self administered

Question 12.3

(If you do not practice any sport, otherwise go to question 12.4)

During your spare time, does it happen to you to practice any type of physical activity such as walking for at least 2 kilometres, swimming, riding a bicycle or other at least a number of times during the year?

NO
YES, once or more than once a week
YES, once or more than once a month
YES, less frequently

Question 12.4

(If you practice sport regularly or occasionally, otherwise go to question 13.1)

Over the last 12 months, have you practiced sports in places upon payment?

NO
YES

Question 12.5

Over the last 12 months, have you had sports private classes or courses at your or your family's expenses?

NO
YES

Question 12.6

Do you pay a yearly or periodical fee to attend a sports club?

NO
YES

Country:	Netherlands
Survey:	Continuous Quality of Life Survey
Survey Code:	NL 09
Year:	2005
Type:	Face to Face + self administered

Question Wandelen

Do you walk regularly for pleasure?

Yes
No
Doesn't know
Refuses to answer

Question UurWand

How many hours do you spend walking?

5 or more hours a week
1-5 hours a week
Less than 1 hour a week
Never
Doesn't know
Refuses to answer

Question Fietsen

Do you cycle regularly for pleasure?

Yes
No
Doesn't know
Refuses to answer

Question UurFiets

How many hours do you spend cycling?

5 or more hours a week
1-5 hours a week
Less than 1 hour a week
Never
Doesn't know
Refuses to answer

Country: Sweden

Survey: National Survey of Public Health

Survey Code: SE 05

Year: 2005

Type: ???

Question 36

How much have you moved about and exerted yourself physically in your free time during the past 12 months?
If your activity varies between e.g. summer and winter, try and give an average.
Note! Mark only one alternative.

Free time spent sitting down
You spend time mostly reading, with TV, cinema or other sedentary occupation during your free time. You walk, cycle or move about in other ways for less than 2 hours a week.

Moderate exercise in free time
You walk, cycle or move about in other ways for at least two hours a week without sweating. This includes cycling to and from work, other walks, normal gardening, fishing, table-tennis, bowling.

Moderate regular exercise in free time
You exercise regularly 1-2 times a week for at least 30 minutes at a time. running, swimming, tennis, badminton or other activity that makes you sweat.

Regular exercise and training
You spend time for example running, swimming, tennis, badminton, exercise gymnastics or similar on at least three occasions a week. Each occasion lasts at least 30 minutes.

Country:	Sweden
Survey:	Survey of Living Conditions
Survey Code:	SE07
Year:	2005
Type:	Face to Face

Question 60c

I would now like to ask about how much exercise you get in your leisure time.
Which of these alternatives fit you best?

Practically no exercise at all
Some exercise now and then (e.g. short walks)
Exercise regularly about once a week
Exercise regularly about twice a week
Exercise regularly and fairly vigorously at least twice a week

Country: United Kingdom

Survey: Health Survey for England

Survey Code: UK 31

Year: 2005

Type: Face to Face

Question Sport

I would now like to ask you about any sports or exercise activities that you have/ (name of child) has done. I will then go on to ask about other active things you/s/he may have done like running about, riding a bike, kicking a ball around and things like that.

For the following questions please include any activities done at a nursery or playgroup/don't count any activities done as part of school lessons.
(Continue)

In the last week, that is last (date last week) up to yesterday, have you/has (name of child) done any sports or exercise activities, not counting things done as part of school lessons?
This card shows some of the things you/s/he might have done; please also include any other sports or exercise activities like these.

Yes
No

Question WESpDo

Did you/(name of person) do any of these sports or exercise activities at the weekend, that is last Saturday and Sunday/that is yesterday and last Sunday?

Yes
No

Question DWESp

Was that on Saturday or Sunday or on both days?

Saturday only
Sunday only
Both Saturday and Sunday

Question WeSpor

On Saturday/Sunday/Saturday and Sunday when you/(name of child) did these sports or exercise activities, how long did you/s/he spend on each day/?
Please give an answer from this card.

Less than 5 minutes
5 minutes, less than 15 minutes
15 minutes, less than 30 minutes
30 minutes, less than 1 hour
1 hour, less than 1 1/2 hours
1 1/2 hours, less than 2 hours
2 hours, less than 2 1/2 hours
2 1/2 hours, less than 3 hours
3 hours, less than 3 1/2 hours
3 1/2 hours, less than 4 hours
4 hours or more (please specify how long)

Question WeSpH

How long did you/(name of child) spend doing these sports or exercise activities?

...

Question DaySp

Still thinking about last week. On how many of the weekdays did you/(name of child) do any of these sports or exercise activities? Please remember not to count things done as part of school lessons.

None in last week
1 day
2 days
3 days
4 days
5 days

Question WkSpor

On each weekday that you/(name of child) did these sports or exercise activities, how long did you/s/he spend? Please give an answer from this card.

Less than 5 minutes
5 minutes, less than 15 minutes
15 minutes, less than 30 minutes
30 minutes, less than 1 hour
1 hour, less than 1 1/2 hours
1 1/2 hours, less than 2 hours
2 hours, less than 2 1/2 hours
2 1/2 hours, less than 3 hours
3 hours, less than 3 1/2 hours
3 1/2 hours, less than 4 hours
4 hours or more (please specify how long)

Question WkSpH

How long did you/(name of child) spend doing these sports or exercise activities on each weekday?

...

Question WEActDo

Now I would like to know about when you do/(name of child) does active things, like the things on this card or other activities like these. Did you/s/he do any active things like these at the weekend, that is last Saturday and Sunday/that is yesterday and last Sunday?

Yes
No

Question DWEAct

Was that on Saturday or Sunday or on both days?

Saturday only
Sunday only
Both Saturday and Sunday

Question WeAct

On Saturday/Sunday/Saturday and Sunday when you/(name of child) did active things like these, how long did you/s/he spend on each day/?
Please give an answer from this card.

Less than 5 minutes
5 minutes, less than 15 minutes
15 minutes, less than 30 minutes
30 minutes, less than 1 hour
1 hour, less than 1 1/2 hours
1 1/2 hours, less than 2 hours
2 hours, less than 2 1/2 hours
2 1/2 hours, less than 3 hours
3 hours, less than 3 1/2 hours
3 1/2 hours, less than 4 hours
4 hours or more (please specify how long)

Question WeActH

How long did you/(name of child) spend doing active things like these?

...

Question WkActDo

Still thinking about last week. On how many of the weekdays did you/(name of child) do active things on this card or other activities like these not counting things done as part of school lessons?

None in last week
1 day
2 days
3 days
4 days
5 days

Question WkAct

On each weekday that you/(name of child) did active things like these, how long did you/s/he spend? Please give an answer from this card.

Less than 5 minutes
5 minutes, less than 15 minutes
15 minutes, less than 30 minutes
30 minutes, less than 1 hour
1 hour, less than 1 1/2 hours
1 1/2 hours, less than 2 hours
2 hours, less than 2 1/2 hours
2 1/2 hours, less than 3 hours
3 hours, less than 3 1/2 hours
3 1/2 hours, less than 4 hours
4 hours or more (please specify how long)

Question WkActH

How long did you/(name of child) spend doing active things like these on each weekday?

...

Question DaysTot

Now thinking about all the activities during the past week you have just told me about including any walking, gardening, housework, sports or other active things. On how many days in the last week in total did you/(name of child) do any of these activities not counting things done as part of school lessons?

None
One day
Two days
Three days
Four days
Five days
Six days
Every day

Question Usual

Were the activities you/(name of child) did last week different from what you/s/he would usually do for any reason?

IF YES PROBE: Would you/s/he usually do more physical activity or less?

No - same as usual
Yes different - usually do more
Yes different - usually do less

Country: Austria

Survey: Health Interview Survey

Survey Code: AT 06

Year: 2006

Type: Face to Face

Question D5.7

What describes best your leisure time activities during the last year?

Hard training and competitive sport more than once a week
Jogging and other recreational sports or heavy gardening, at least 4 hours a week
Jogging and other recreational sports or heavy gardening, less than 4 hours a week
Walking, bicycling or other light activities at least 4 hours a week
Walking, bicycling or other light activities less 4 hours a week
Reading, watching TV or other sedentary activities

Country: Serbia

Survey: National Health Survey, Serbia 2006

Survey Code: CS 01

Year: 2006

Type: Face to Face + self administered

Question SV2

How much physical activity do you get in your leisure time? (One answer.)
If examinee states more than one answer, only one which relates to the most intensive, i.e. activity which requires highest physical effort should be marked.

Read, watch TV or similar
Mainly walking, drive bicycle or similar (walking, fishing, hunting) at least 4 hours per week
I keep fit (running, swimming, skiing, ball playing, hard work in the garden or similar) at least 4 hours per week
I practice sport at least few times a week

Question SV3

How often are you engaged in physical activity exercises at least 30 minutes in leisure time, so that you start breathing faster or start sweating? (One answer)

Every day
4-6 times a week
2-3 times a week
Once a week
2-3 times per month
Few times a year/never
Can not exercise due to illness/invalidity

Question SV5

How often do you engage in physical exercises, in leisure time, so that you start breathing faster or start sweating?

Almost every day
3 times a week
1-2 times a week
2-3 times a month
A few times a year/never
Cannot exercise due to illness/invalidity

Question SV6

How many hours a week do you practice physical activity until you start sweating and start breathing faster (excluding school activities)?

Up to 1 hour
2-3 hours
4-6 hours
7 or more than 7 hours

Country: Germany

Survey: Telephone Health Survey 2005-2006

Survey Code: DE 08

Year: 2006

Type: Telephone

Question 138

Thinking back over the last 3 months, have you done any sport during that time?

yes
no
d.k.
n.a.

Question 139

... and how many hours in the weeks was it?

Was it ...

up to an hour
up to two hours
up to four hours
more than four hours
d.k.
n.a./answer refused

Country: Estonia

Survey: Estonian Health Interview Survey 2006

Survey Code: EE 08

Year: 2006

Type: Face to Face

Question T13

Now we are going to talk about physical activity and exercises that can be called active exercises. These include jogging, ball games, swimming, skating, dancing and also harder garden work.

Have you ever in your life been involved in active exercises? These include jogging, other recreational sports and harder garden work.

Yes
No

Question T14

Have you participated in active exercises during the last 4 weeks?

Yes
No

Question T15

How often did you participate in active exercises during the last 4 weeks?

Almost every day
3 – 4 times a week
1 – 2 times a week
1 – 3 times a month

Question T16

How many hours per week did you participate in active exercises during the last 4 weeks?

... hours

Question T17

The following questions are connected with intensive sports activities.

Have you ever in your life been involved in intensive training or competitive sports, i.e. at least 3-4 training sessions per week, during at least one year?

Yes
No

Question T18

Have you participated in intensive training or competitive sports, i.e. at least 3-4 training sessions per week, during the last 4 weeks?

Yes
No

Question T19

How often did you participate in intensive training or competitive sports, i.e. 3-4 training sessions a week, during the last 4 weeks?

Every day/almost every day
3 – 4 times a week

Country: Spain

Survey: National Health Survey

Survey Code: ES 06

Year: 2006

Type: Face to Face

Question 91

Do you normally do all the physical exercise you would like to do in your free time?

Yes
No

Question 92

Why not?

Because my health problems prevent me from doing so
Because I prefer to do other kinds of leisure activities (go to the cinema, read a book, watch TV, etc.)
Because I haven't got a place to do the physical activities I would like to do
Due to lack of time
Due to a lack of will power
Other reasons

Question 93

Do you regularly do any physical activity like walking, doing sport or going to the gym in your free time?

Yes
No, I don't normally do physical exercise

Question 94

Could you tell me how often you have done the activities set out below for over 20 minutes in the last two weeks?

N° of times: ...

A light physical activity like walking, gardening, easy gym activities, games that require little effort or similar activities
A moderate physical activity like riding a bicycle, gymnastics, aerobics, running or swimming

An intense physical activity like football, basketball, competitive cycling or swimming, judo karate or similar activities

---

<u>Question 57</u>

Which of the following possibilities best describes the frequency with which the child performs any kind of physical activity in his/her free time?

He/She doesn’t do exercise. His/Her free time is almost completely taken up by sedentary activities (reading, watching television, going to the cinema, lying in bed or the cot, etc.)
He/she does some kind of physical activity or sport (walking, riding a bike, light gym activities, light recreational activities, etc.)
He/she does physical activities several times a week (sports, gymnastics, running, swimming, cycling, team sports, etc.)
Does sport or physical training several times a week

Country: France

Survey: National Survey on Nutrition and Health (ENNS)

Survey Code: FR 18

Year: 2006

Type: Face to Face

Question E58

During the past week, on how many days did your child play outdoors apart from at school? This means unsupervised activities such as playing in the square, outside the house, going rollerblading, etc.

School days: … days /5 days max.
Days with little or no school: … days/3 days max.
Doesn't know
Refuses to answer

Question E60

During the past week, during how many hours did your child have sports lessons outside of school (team sports, dance, tennis, judo, etc.)?

… days/7 days
Refuses to answer
Doesn't know

Country: Italy

Survey: Aspects of Daily Living

Survey Code: IT 10

Year: 2006

Type: Face to Face + self administered

Question 12.3

(If you do not practice any sport, otherwise go to question 12.4)

During your spare time, does it happen to you to practice any type of physical activity such as walking for at least 2 kilometres, swimming, riding a bicycle or other at least a number of times during the year?

NO
YES, once or more than once a week
YES, once or more than once a month
YES, less frequently

Question 12.4

(If you practice sport regularly or occasionally, otherwise go to question 13.1)

Over the last 12 months, have you practiced sports in places upon payment?

NO
YES

Question 12.5

Over the last 12 months, have you had sports private classes or courses at your or your family's expenses?

NO
YES

Question 12.6

Do you pay a yearly or periodical fee to attend a sports club?

NO
YES

Country:	Lithuania
Survey:	Lithuanian Health Behaviour Monitoring
Survey Code:	LT 05
Year:	2006
Type:	Self administered

Question 74

In you leisure time, how often do you do physical exercise for at least 30 min which makes you at least mildly short of breath or perspire?

daily
4-6 times a week
2-3 times a week
once a week
2-3 times a month
a few times a year or less
I cannot exercise because of an illness

Country:	Netherlands
Survey:	Continuous Quality of Life Survey
Survey Code:	NL 10
Year:	2006
Type:	Face to Face + self administered

Question Wandelen

Do you walk regularly for pleasure?

Yes
No
Doesn't know
Refuses to answer

Question UurWand

How many hours do you spend walking?

5 or more hours a week
1-5 hours a week
Less than 1 hour a week
Never
Doesn't know
Refuses to answer

Question Fietsen

Do you cycle regularly for pleasure?

Yes
No
Doesn't know
Refuses to answer

Question UurFiets

How many hours do you spend cycling?

5 or more hours a week
1-5 hours a week
Less than 1 hour a week
Never
Doesn't know
Refuses to answer

Country: United Kingdom

Survey: Health Survey for England

Survey Code: UK 33

Year: 2006

Type: Face to Face

Question SportDo

I would now like to ask you about any sports or exercise activities that you have/(name of child) has done. I will then go on to ask about other active things you/s/he may have done like running about, riding a bike, kicking a ball around and things like that. For the following questions please include any activities done at a nursery or playgroup/don't count any activities done as part of school lessons.

In the last week, that is last (date last week) up to yesterday, have you/has (name of child) done any sports or exercise activities, not counting things done as part of school lessons?
This card shows some of the things you/s/he might have done; please also include any other sports or exercise activities like these.

Yes
No

Question WESpDo

Did you/(name of person) do any of these sports or exercise activities at the weekend, that is last Saturday and Sunday/that is yesterday and last Sunday?

Yes
No

Question DWESp

Was that on Saturday or Sunday or on both days?

Saturday only
Sunday only
Both Saturday and Sunday

Question WeSpor

On Saturday/Sunday/Saturday and Sunday when you/(name of child) did these sports or exercise activities, how long did you/s/he spend on each day?

Less than 5 minutes
5 minutes, less than 15 minutes
15 minutes, less than 30 minutes
30 minutes, less than 1 hour
1 hour, less than 1 1/2 hours
1 1/2 hours, less than 2 hours
2 hours, less than 2 1/2 hours
2 1/2 hours, less than 3 hours
3 hours, less than 3 1/2 hours
3 1/2 hours, less than 4 hours
4 hours or more (please specify how long)

Question WeSpH-WeSpM

4 hours or more THEN

How long did you/(name of child) spend doing these sports or exercise activities?

RECORD HOURS SPENT BELOW

...

RECORD HERE MINUTES SPENT WALKING.

...

Question DaySp

Still thinking about last week. On how many of the weekdays did you/(name of child) do any of these sports or exercise activities? Please remember not to count things done as part of school lessons.

None in last week
1 day
2 days
3 days
4 days
5 days

Question WkSpor

On each weekday that you/(name of child) did these sports or exercise activities, how long did you/s/he spend?

Less than 5 minutes
5 minutes, less than 15 minutes
15 minutes, less than 30 minutes
30 minutes, less than 1 hour
1 hour, less than 1 1/2 hours
1 1/2 hours, less than 2 hours
2 hours, less than 2 1/2 hours
2 1/2 hours, less than 3 hours

3 hours, less than 3 1/2 hours
3 1/2 hours, less than 4 hours
4 hours or more, please specify how long: ...

Question WkSpH-WkSpM

4 hours or more THEN

How long did you/(name of child) spend doing these sports or exercise activities on each weekday?

RECORD HOURS SPENT BELOW

...

RECORD HERE MINUTES SPENT WALKING.

...

Question DaysTot

Now thinking about all the activities during the past week you have just told me about including any walking, gardening, housework, sports or other active things. On how many days in the last week in total did you/(name of child) do any of these activities not counting things done as part of school lessons?

None
One day
Two days
Three days
Four days
Five days
Six days
Every day

Question WESitDo-DSit

Now I'd like to know about when you/(name of child) spend time sitting down doing things like the ones on this card. Did you/s/he spend time sitting down doing any things like these for at least 5 minutes a time at the weekend

Yes
No

Was that on Saturday or Sunday or on both days?

Saturday only
Sunday only
Both Saturday and Sunday

Question Garden

Have you done any gardening, DIY or building work in the past four weeks, that is since (date four weeks ago)?

Yes
No

Have you done any gardening, DIY or building work listed on this card?

Yes
No

Question ManWork

Have you done any gardening, DIY or building work from this other card, or any similar heavy manual work?

Yes
No

During the past four weeks on how many days have you done this kind of heavy manual gardening or DIY, for at least 30 minutes a time?

...

Question ManDays

During the past four weeks on how many days have you done this kind of heavy manual gardening or DIY?

...

Question HrsDIY

On the days you did heavy manual gardening or DIY, how long did you usually spend?

...

Record minutes spent on gardening or DIY

...

Question ActPhy

Can you tell me if you have done any activities on this card during the last four weeks, that is since (date four weeks ago)? Include teaching, coaching, training

and practice sessions.

Yes
No

Question WhtAct

Which have you done in the last four weeks? Any others?

Swimming
Cycling
Workout at a gym/Exercise bike/ Weight training
Aerobics/Keep fit/Gymnastics/ Dance for fitness
Any other type of dancing
Running/jogging
Football/rugby
Badminton/tennis
Squash
Exercises (e.g. press-ups, sit ups)

Question OActQ

Have you done any other sport or exercise not listed on the card?

Yes
No

Probe for name of sport or exercise.

...

Question DayExc

Can you tell me on how many separate days did you do (activity) for at least 15 minutes a time during the past four weeks, that is since (date four weeks ago)?

...

Question ExcHrs

How much time did you usually spend doing (activity) on each day? (Only count times you did it for at least 15 minutes.)

...

RECORD MINUTES HERE

...

During the past four weeks, was the effort of (activity) usually enough to make

you out of breath or sweaty?

Yes
No

Country: United States

Survey: National Health Interview Survey

Survey Code: US 04

Year: 2006

Type: Face to Face + Telephone

Question AHB.090

The next questions are about physical activities (exercise, sports, physically active hobbies...) that you may do in your LEISURE time.

How often do you do VIGOROUS leisure-time physical activities for AT LEAST 10 MINUTES that cause HEAVY sweating or LARGE increases in breathing or heart rate?
How many times per day, per week, per month, or per year do you do these activities?

Never
1-995 time(s)
Unable to do this type activity
Refused
Don't know

Enter time period for vigorous leisure-time physical activities.

Never
Per day
Per week
Per month
Per year
Unable to do this activity
Refused
Don't know

Question AHB.100

About how long do you do these vigorous leisure-time physical activities each time?
Enter number for length of vigorous leisure-time physical activities.

1-995
Refused
Don't know

Enter time period for length of vigorous leisure-time physical activities.

Minutes
Hours
Refused
Don't know

Question AHB.110

How often do you do LIGHT OR MODERATE LEISURE-TIME physical activities for AT LEAST 10 MINUTES that cause ONLY LIGHT sweating or a SLIGHT to MODERATE increase in breathing or heart rate?

If necessary, prompt with: How many times per day, per week, per month, or per year do you do these activities?

Never
1-995 time(s)
Unable to do this type activity
Refused
Don't know

Enter time period for light or moderate leisure-time physical activities

Never
Per day
Per week
Per month
Per year
Unable to do this activity
Refused
Don't know

Question AHB.120

About how long do you do these light or moderate leisure-time physical activities each time?
Enter number for length of light or moderate leisure-time physical activities.

1-995
Refused
Don't know

Enter time period for length of light or moderate leisure-time physical activities.

Minutes
Hours
Refused
Don't know

Question AHB.130

How often do you do LEISURE-TIME physical activities specifically designed to STRENGTHEN your muscles such as lifting weights or doing calisthenics? (Include all such activities even if you have mentioned them before.)

How many times per day, per week, per month, or per year do you do these activities?

Never

1-995 time(s)
Unable to do this type activity
Refused
Don't know

Enter time period for strengthening activities

Never
Per day
Per week
Per month
Per year
Unable to do this activity
Refused
Don't know

Country: Australia

Survey: National Health Survey

Survey Code: AU 04

Year: 2007

Type: Face to Face

Question EXER_Q01

The next few questions are about walking for fitness, recreation and sport. Please do not include any other walking that [you/proxy name] may have done for other reasons. This will be recorded later.

In the last 2 weeks [have you/has (proxy name)] walked for fitness, recreation or sport?

Yes
No
Permanently unable to walk

Question EXER_Q02

How many times did [you/proxy name] walk (for fitness, recreation or sport) in the last 2 weeks.

...

Question EXER_Q03

What was the total amount of time [you/proxy name] spent walking (for fitness, recreation or sport) in the last 2 weeks?

hours: ...
minutes: ...

Question EXER_Q04

How many days in the last week did [you/proxy name] walk for fitness, recreation or sport?

...

Question EXER_Q06

What was the total amount of time [you/proxy name] spent walking for fitness, recreation or sport in the last week?

hours: ...
minutes: ...

Country:	Canada
Survey:	Canadian Health Measures Survey
Survey Code:	CA 04
Year:	2007
Type:	Face to Face

Question PAC_Q11

Now I'd like to ask you about some of your physical activities. To begin with, I'll be dealing with physical activities not related to work, that is, leisure time activities.

Have you done any of the following in the past 3 months, that is, from [date three months ago] to yesterday? Mark all that apply.

Walking for exercise
Gardening or yard work
Swimming
Bicycling
Popular or social dance
Home exercises
Ice hockey
Ice skating
In-line skating or rollerblading
Jogging or running
Golfing
Exercise class or aerobics
Downhill skiing or snowboarding
Bowling
Baseball or softball
Tennis
Weight-training
Fishing
Volleyball
Basketball
Soccer
Any other
No physical activity
Don't know
Refusal

If "Any other" is chosen as a response
What was this activity?
...

Question PAC_Q11X

In the past 3 months, did you do any other physical activity for leisure?

Yes
No

What was this activity?
...

For each activity identified

Question PAC_Q12

In the past 3 months, how many times did you [participate in identified activity]?

... Times
(MIN: 1) (MAX: 95) for each activity except the following:
Walking: MAX = 270
Bicycling: MAX = 200
Other activities: MAX = 200
Don't know
Refusal

About how much time did you spend on each occasion?

1 to 15 minutes
16 to 30 minutes
31 to 60 minutes
More than one hour

Question PAC_Q12

In the past 3 months, how many times did you [participate in identified activity]?

... Times
(MIN: 1) (MAX: 95) for each activity except the following:
Walking: MAX = 270
Bicycling: MAX = 200
Other activities: MAX = 200
Don't know
Refusal

Question CPA_Q15

About how many hours a week does he usually take part in physical activity (that makes him out of breath or warmer than usual):
… outside of school while participating in lessons or league or team sports?

Never
Less than 2 hours per week
2 to 3 hours per week

4 to 6 hours per week
7 or more hours per week

Question CPA_Q16

About how many hours a week does he usually take part in physical activity (that makes him out of breath or warmer than usual):
… outside of school while participating in unorganized activities, either on his own or with friends?

Never
Less than 2 hours per week
2 to 3 hours per week
4 to 6 hours per week
7 or more hours per week

Country: Canada

Survey: Canadian Community Health Survey (CCHS)

Survey Code: CA 05

Year: 2007

Type: Face to Face + Telephone

Question PAC_Q1

Now I'd like to ask you about some of your physical activities. To begin with, I'll be dealing with physical activities not related to work, that is, leisure time activities.

Have you done any of the following in the past 3 months, that is, from [date three months ago] to yesterday?

Walking for exercise
Gardening or yard work
Swimming
Bicycling
Popular or social dance
Home exercises
Ice hockey
Ice skating
In-line skating or rollerblading
Jogging or running
Golfing
Exercise class or aerobics
Downhill skiing or snowboarding
Bowling
Baseball or softball
Tennis
Weight-training
Fishing
Volleyball
Basketball
Soccer
Any other
No physical activity

What was this activity?

....

Question PAC_Q1X

In the past 3 months, did you do any other physical activity for leisure?

Yes
No

What was this activity?

...

Question PAC_Q2n

In the past 3 months, how many times did you [participate in identified activity]?

... Times

Question PAC_Q3n

About how much time did you spend on each occasion?

1 to 15 minutes
16 to 30 minutes
31 to 60 minutes
More than one hour

Question UPE_Q2A-C

When in-line skating or rollerblading, how often do you wear a helmet?

Always
Most of the time
Rarely
Never

How often do you wear wrist guards or wrist protectors?

Always
Most of the time
Rarely
Never

How often do you wear elbow pads?

Always
Most of the time
Rarely
Never

Question UPE_Q3A

Earlier, you mentioned going downhill skiing or snowboarding in the past 3 months. Was that:

… downhill skiing only?
… snowboarding only?
… both ?

Question UPE_Q3B

In the past 12 months, did you do any downhill skiing or snowboarding?

Downhill skiing only
Snowboarding only
Both
Neither

Question UPE_Q4A-5AB

When downhill skiing, how often do you wear a helmet?

Always
Most of the time
Rarely
Never

When snowboarding, how often do you wear a helmet?

Always
Most of the time
Rarely
Never

How often do you wear wrist guards or wrist protectors?

Always
Most of the time
Rarely
Never

Question UPE_Q6

In the past 12 months, have you done any skateboarding?

Yes
No

Question STC_Q1_6

How often do you jog or do other exercise to deal with stress?

Often
Sometimes
Rarely
Never

Country: Switzerland

Survey: Swiss Health Survey

Survey Code: CH 03

Year: 2007

Type: Telephone + self administered

Question 46.00

Do you work up a sweat at least once a week as a result of physical exercise in your free time? (e.g. brisk walking, running, cycling, etc)

Yes
No
No answer

Question 46.01

How many days per week (on average)?

... days per week (1 to 7 days)
No answer

Question 46.30

Do you do regular exercise, follow a fitness programme or practise a sport?

Yes
No
No answer

Question 46.31

How often do you do regular exercise, follow a fitness programme or practise a sport?

(Almost) every day
Several times a week
About once a week
About once to three times a month
Less than once a month
Don't know
No answer

Question 46.32

And what about these physical activities or sports? Do you practise them ...

Very intensively
Pretty intensively
With an average intensity
Not very intensively
Not at all intensively
No answer

Question 46.33

On average how many hours per week does that represent?

... hours and ... minutes per day
Don't know
No answer

Country: Finland

Survey: FINRISK 2007

Survey Code: FI 21

Year: 2007

Type: Self administered

Question 79

How much do you exercise and stress yourself physically in your leisure time?
If it varies much according to different seasons, mark the alternative which best describes the average situation.

In my leisure time I read, watch TV, and work in the household with tasks which do not make me move much and which do not physically tax me.

In my spare time I walk, cycle or exercise otherwise at least 4 hours per week. This includes walking, fishing and hunting, light gardening etc. but excludes travel to work.

In my spare time I exercise to maintain my physical condition, e.g. running, jogging, skiing, gymnastics, swimming, playing ball games or I do heavy gardening or the like for at least 3 hours per week.

In my spare time I regularly exercise several times a week competitive sports such as running, orienteering, skiing, swimming, playing ball games or other heavy sports.

Question 81

How often do you in your leisure time exercise for at least 20 minutes so that you at least are mildly out of breath and sweaty? (the exercise of travelling to and from work not included)

I have a disability or a disease which does not enable me to exercise
less than once a week
once a week
2 times a week
3 times a week
4 times a week
5 times a week or more often

Question 82

How long does your usual leisure time activity take?

I do not exercise in my free time
less than 15 minutes

15 - 29 minutes
30 - 59 minutes
one hour or longer

Question 83

How many minutes do you daily walk, cycle or engage in a hobby in your leisure time that requires moving about (yard work or gardening, fixing or cleaning the house)? Do not count in the activity needed at work, travelling to work or leisure time sports.

less than 15 minutes per day
15-29 minutes per day
30-44 minutes daily
45-59 minutes daily
over an hour per day

Question 85

Evaluate how your leisure time physical activity is divided into different environments.

... % in indoor sport facilities (e.g. swimming hall, gym)
... % home, indoors or in the yard
... % outdoors in the artificial surroundings (streets, cycle roads, outdoors sport facilities)
... % outdoors in the nature near home
... % outdoors in nature near leisure-time apartment
... % outdoors in another natural environment

Question 129

Let's assume that you have decided to start a new sport. Your friend recommends you a programme involving practice twice a week an hour at a time. The best time for your friend is in the morning at 7:00-8:00. Considering only the daily rhythm that feels right for you, how do you think you would perform?

I would be in good condition
I would be in moderate condition
it would feel quite difficult
it would feel very difficult

Country: Finland

Survey: Health Behaviour and Health among the Finnish Adult Population

Survey Code: FI 22

Year: 2007

Type: Self administered

Question 74

In your leisure time, how often do you take physical activity for at least 30 min, which makes you at least moderately out of breath and causes you to perspire?

daily
4-6 times a week
3 times a week
2 times a week
once a week
2–3 times a month
a few times a year or less
I cannot exercise due to an illness or injury

Question 76

How much do you exercise and exert yourself physically in your leisure time? If the amount of exertion varies much depending on the time of the year, choose the alternative that best describes the average situation.

during my leisure time I read, watch TV and perform chores where I do not need to move much and that do not tire me physically
during my leisure time I walk, bicycle or exercise otherwise at least 4 hours a week
during my leisure time, I do active fitness training at least 3 hours a week on average
during my leisure time, I train actively for competitions several times a week

Country:	Ireland
Survey:	Survey of Lifestyles, Attitudes and Nutrition SLAN
Survey Code:	IE 09
Year:	2007
Type:	Face to Face

Question B1

We are interested in finding out about the kinds of physical activities that people do as part of their everyday lives.

First, consider a 7 day period (a week). How many times on average do you do the following kinds of exercise for more than 20 minutes during your free time?

Strenuous exercise (heart beats rapidly) (e.g. running, jogging, hurling, camogie, football, soccer, squash, basketball, judo, roller skating, vigorous swimming, vigorous long distance cycling, advanced aerobics)
Moderate exercise (not exhausting) (e.g. fast walking, tennis, badminton, easy swimming, easy cycling, popular and folk dancing, intermediate aerobics, heavy gardening)
Mild exercise (minimal effort) (e.g. yoga, golf, easy walking, fishing from river bank, bowling, beginners aerobics, archery, light gardening)

... times

Country: Italy

Survey: Aspects of Daily Living

Survey Code: IT 11

Year: 2007

Type: Face to Face + self administered

Question 12.3

(If you do not practice any sport, otherwise go to question 12.4)

During your spare time, does it happen to you to practice any type of physical activity such as walking for at least 2 kilometres, swimming, riding a bicycle or other at least a number of times during the year?

NO
YES, once or more than once a week
YES, once or more than once a month
YES, less frequently

Question 12.4

(If you practice sport regularly or occasionally, otherwise go to question 13.1)

Over the last 12 months, have you practiced sports in places upon payment?

NO
YES

Question 12.5

Over the last 12 months, have you had sports private classes or courses at your or your family's expenses?

NO
YES

Question 12.6

Do you pay a yearly or periodical fee to attend a sports club?

NO
YES

Country: Luxembourg

Survey: PSELL/EU-SILC

Survey Code: LU 04

Year: 2007

Type: Face to Face

Question D187

Do you do a physical activity?

Yes, regularly
Yes, sometimes
No, because of health problems
No, for other reasons

Question D188

Do you do a physical activity or an artistic activity out of your home?

Yes, regularly
Yes, sometimes
No, because of health problems
No, for other reasons

Country: Netherlands

Survey: Continuous Quality of Life Survey

Survey Code: NL 11

Year: 2007

Type: Face to Face + self administered

Question Wandelen

Do you walk regularly for pleasure?

Yes
No
Doesn't know
Refuses to answer

Question UuWand

How many hours do you spend walking?

5 or more hours a week
1-5 hours a week
Less than 1 hour a week
Never
Doesn't know
Refuses to answer

Question Fietsen

Do you cycle regularly for pleasure?

Yes
No
Doesn't know
Refuses to answer

Question UuFiets

How many hours do you spend cycling?

5 or more hours a week
1-5 hours a week
Less than 1 hour a week
Never
Doesn't know
Refuses to answer

Country: Sweden

Survey: Living Conditions Survey

Survey Code: SE 08

Year: 2007

Type: Face to Face

Question 91c

I would now like to ask about how much exercise you get in your leisure time.

Which of these alternatives fit you best?

Practically no exercise at all
Some exercise now and then (e.g short walks)
Exercise regularly about once a week
Exercise regularly about twice a week
Exercise regularly and fairly vigorously at least twice a week

Country: Sweden

Survey: National Survey of Public Health

Survey Code: SE 09

Year: 2007

Type: ???

Question 36

How much have you moved about and exerted yourself physically in your free time during the past 12 months?
If your activity varies between e.g. summer and winter, try and give an average.
Note! Mark only one alternative.

Free time spent sitting down
You spend time mostly reading, with TV, cinema or other sedentary occupation during your free time. You walk, cycle or move about in other ways for less than 2 hours a week.

Moderate exercise in free time
You walk, cycle or move about in other ways for at least two hours a week without sweating. This includes cycling to and from work, other walks, normal gardening, fishing, table-tennis, bowling.

Moderate regular exercise in free time
You exercise regularly 1-2 times a week for at least 30 minutes at a time. running, swimming, tennis, badminton or other activity that makes you sweat.

Regular exercise and training
You spend time for example running, swimming, tennis, badminton, exercise gymnastics or similar on at least three occasions a week. Each occasion lasts at least 30 minutes.

Country: United Kingdom

Survey: Health Survey for England

Survey Code: UK 35

Year: 2007

Type: Face to Face + self administered

Question SportDo

I would now like to ask you about any sports or exercise activities that you have/(name of child) has done. I will then go on to ask about other active things you/s/he may have done like running about, riding a bike, kicking a ball around and things like that. For the following questions please include any activities done at a nursery or playgroup/don't count any activities done as part of school lessons.

In the last week, that is last (date last week) up to yesterday, have you/has (name of child) done any sports or exercise activities, not counting things done as part of school lessons?
This card shows some of the things you/s/he might have done; please also include any other sports or exercise activities like these.

Yes
No

Question WESpDo

Did you/(name of person) do any of these sports or exercise activities at the weekend?

Yes
No

Was that on Saturday or Sunday or on both days?

Saturday only
Sunday only
Both Saturday and Sunday

Question DWESp

Was that on Saturday or Sunday or on both days?

Saturday only
Sunday only
Both Saturday and Sunday

Question WeSpor

On (Saturday/Sunday) when (you/child's name) did these
sports or exercise activities, how long did you/he/she spend (on each day)?

15 minutes, less than 30 minutes
30 minutes, less than 1 hour
1 hour, less than 1 1/2 hours
1 1/2 hours, less than 2 hours
2 hours, less than 2 1/2 hours
2 1/2 hours, less than 3 hours
3 hours, less than 3 1/2 hours
3 1/2 hours, less than 4 hours
4 hours or more (please specify how long)

Question WeSpH-WeSpM

More than four hours THEN

How long did (you/child's name) spend doing these sports or exercise activities on
(that/each) day?

RECORD HOURS SPENT BELOW

...

RECORD HERE MINUTES DOING SPORTS OR EXERCISE ACTIVITIES.

...

Question DaySp

Still thinking about last week. On how many of the weekdays did (you/child's name) do any
of these sports or exercise activities? Please remember not to
count things done as part of school lessons.

None in last week
1 day
2 days
3 days
4 days
5 days

Question WkSpor

On each weekday that (you/he/she) did these sports or exercise activities,
how long did you/he/she spend?

Less than 5 minutes
15 minutes, less than 30 minutes
30 minutes, less than 1 hour

1 hour, less than 1 1/2 hours
1 1/2 hours, less than 2 hours
2 hours, less than 2 1/2 hours
2 1/2 hours, less than 3 hours
3 hours, less than 3 1/2 hours
3 1/2 hours, less than 4 hours
4 hours or more (please specify how long)

Question WkSpH-WkSpM

4 hours or more THEN

How long did (you/child's name) spend doing these sports or exercise activities on each weekday?

RECORD HOURS SPENT BELOW.

...

RECORD HERE MINUTES SPENT DOING SPORTS OR EXERCISE ACTIVITIES.

...

Question DaysTot

Now thinking about all the activities during the past week you have just told me about including any walking, (gardening, housework), sports or other active things. On how many days in the last week in total did (you/child's name) do any of these activities not counting things done as part of school lessons?

None
One day
Two days
Three days
Four days
Five days
Six days
Every day

Country:	Belgium
Survey:	Health Interview Survey
Survey Code:	BE 06
Year:	2008
Type:	Self administered

Question PA08

Leisure physical activities

The next two questions are about your physical activity during leisure time only

Which proposition best describes your leisure activities during the past year?

Intensive training and competitive sports more than once a week
Jogging, other leisure sport or gardening, at least 4 hours a week
Jogging, other leisure sport or gardening, less than 4 hours a week
Walking, cycling or other mild activity, at least 4 hours a week
Walking, cycling, other mild activity, less than 4 hours a week.
Reading, TV or other sedentary activity
I don't know

Question PA09

Do you practice, at least once a week, during your leisure time, a physical activity such as jogging, cycling, etc. for long enough to perspire?

Yes: … number of days/week
No
I don't know

Country:	France
Survey:	Disabilities and health survey, household part 2008
Survey Code:	FR 20
Year:	2008
Type:	Face to Face

<u>Question MSPORT</u>

In the past 12 months, have you practiced a sport [whether or not with a sports club]? (include hiking and sports walking, dance, etc)

Yes
No
Doesn't know

Was this:

Regularly throughout the year
Occasionally throughout the year
Only at certain periods or during holidays
Occasionally or rarely
Doesn't know

<u>Question MBRIJAR</u>

In the past 12 months have you done any D.I.Y. or gardening? (apart from very small jobs or repairs)

Yes
No
Doesn't know

Was this:

Regularly throughout the year
Occasionally throughout the year
Only at certain periods or during holidays
Occasionally or rarely
Doesn't know

Country: Romania

Survey: Health Interview Survey

Survey Code: RO 02

Year: 2008

Type: Face to Face + self administered

Question 114

Physical activity

Now I would like to ask you about the time you spent on physical activity during the last 7 days. Please answer each question even if you do not see yourself as an active person.
Think of any physical activity, such as: housekeeping or gardening, activities at work, physical exercises, walks or sports.
I will ask separate questions about intense activities, moderate activities and walking. Let us talk first about intense physical activities.
Intense physical activities involve high physical effort and heavier breathing than usual. They can include activities such as: lifting heavy objects, digging, wood cutting, working in constructions, going up the stairs for work, gymnastics or bicycle riding at high speed.
Think only of those physical activities that you performed for at least 10 minutes.

During the last 7 days, on how many days did you have intense physical activities?

total days with vigorous physical activities: ... days week
I don't know/I'm not sure
Refusal

Question 115

On the overall, during these last 7 days, how much time did you spend on intense physical activities?

total time of intense physical activities/week:
... hours ... minutes/week
I don't know/I'm not sure
Refusal

Question 116

Now I would like to ask you about the time you spend on moderate physical activities.
A moderate physical activity is an activity that involves moderate physical effort and that makes you breathe a bit heavier then usual and may include activities such as: lifting of light weights, sweeping the floor, washing windows, riding a

bicycle (at moderate speed), tennis or swimming. Do not include strolls.
Now think of only those moderate physical activities that you performed for at least 10 minutes.

During the last 7 days, on how many days did you have moderate physical activities?

total days with moderate physical activities: ... days/week
I don't know/I'm not sure
Refusal

Question 117

On the overall, during these last 7 days, how much time did you spend on moderate physical activities?

total time of moderate physical activities/week:
... hours ... minutes/week
I don't know/I'm not sure
Refusal

Question 118

Let us talk about the time spent on walking or strolling.
Strolling is a separate activity and includes walking to or from work/school, walking from one place to another or any other strolls you take as physical exercises, sports or only for recreation.
Now think only of those strolls you took for at least 10 minutes.

During the last 7 days, on how many days did you have strolls or walks, for at least 10 minutes?

total days with strolls: ... days/week
I don't know/I'm not sure
Refusal

Question 119

On the overall, during these last 7 days, how much time did you spend on walking or strolling?

total strolling time/day: ... hours ... minutes/day
I don't know/I'm not sure
Refusal

Country:	United Kingdom
Survey:	Health Survey for England
Survey Code:	UK 36
Year:	2008
Type:	Face to Face

Question SportDo

I would now like to ask you about any sports or exercise activities that you have/(name of child) has done. I will then go on to ask about other active things you/s/he may have done like running about, riding a bike, kicking a ball around and things like that. For the following questions please include any activities done at a nursery or playgroup/don't count any activities done as part of school lessons.

In the last week, that is last (date last week) up to yesterday, have you/has (name of child) done any sports or exercise activities, not counting things done as part of school lessons?
This card shows some of the things you/s/he might have done; please also include any other sports or exercise activities like these.

Yes
No

Question WESpDo

Did you/(name of person) do any of these sports or exercise activities at the weekend?

Yes
No

Was that on Saturday or Sunday or on both days?

Saturday only
Sunday only
Both Saturday and Sunday

Question DWESp

Was that on Saturday or Sunday or on both days?

Saturday only
Sunday only
Both Saturday and Sunday

Question WeSpor

On (Saturday/Sunday) when (you/child's name) did these
sports or exercise activities, how long did you/he/she spend (on each day)?

15 minutes, less than 30 minutes
30 minutes, less than 1 hour
1 hour, less than 1 1/2 hours
1 1/2 hours, less than 2 hours
2 hours, less than 2 1/2 hours
2 1/2 hours, less than 3 hours
3 hours, less than 3 1/2 hours
3 1/2 hours, less than 4 hours
4 hours or more (please specify how long)

Question WeSpH-WeSpM

More than four hours THEN

How long did (you/child's name) spend doing these sports or exercise activities on (that/each) day?

RECORD HOURS SPENT BELOW

...

RECORD HERE MINUTES DOING SPORTS OR EXERCISE ACTIVITIES.

...

QuestionDaySp

Still thinking about last week. On how many of the weekdays did (you/child's name) do any
of these sports or exercise activities? Please remember not to
count things done as part of school lessons.

None in last week
1 day
2 days
3 days
4 days
5 days

Question WkSpor

On each weekday that (you/he/she) did these sports or exercise activities,
how long did you/he/she spend?

Less than 5 minutes
15 minutes, less than 30 minutes
30 minutes, less than 1 hour

1 hour, less than 1 1/2 hours
1 1/2 hours, less than 2 hours
2 hours, less than 2 1/2 hours
2 1/2 hours, less than 3 hours
3 hours, less than 3 1/2 hours
3 1/2 hours, less than 4 hours
4 hours or more (please specify how long)

Question WkSpH-WkSpM

4 hours or more THEN

How long did (you/child's name) spend doing these sports or exercise activities on each weekday?

RECORD HOURS SPENT BELOW.

...

RECORD HERE MINUTES SPENT DOING SPORTS OR EXERCISE ACTIVITIES.

...

Question DaysTot

Now thinking about all the activities during the past week you have just told me about including any walking, (gardening, housework), sports or other active things. On how many days in the last week in total did (you/child's name) do any of these activities not counting things done as part of school lessons?

None
One day
Two days
Three days
Four days
Five days
Six days
Every day

International Survey Items concerning

Sedentary Behaviour

1995 - 2008

TABLE OF CONTENTS

1995
Portugal – PT 01 2

1998
United Kingdom – UK 09 3

1999
Italy – IT 01 5

2000
International - EUPASS 6

2001
Croatia – HR 02 7
Macedonia – MK 03 8
Slovenia – SI 01 9

2002
International – INT 08 10
Malta – MT 01 11
Slovakia – SK 02 12

2004
Belgium – BE 05 13
International – FINBALT 14

2005
Denmark – DK 03 15
France – FR 14 16
Portugal – PT 05 17
United Kingdom – UK 31 18

2006
Serbia – CS 01 20
Estonia – EE 08 21
France – FR 18 22
United Kingdom – UK 33 23

2007
Finland – FI 21 25
Iceland – IS 04 26
United Kingdom – UK 35 27

2008
United Kingdom – UK 36 29

Country:	Portugal
Survey:	National Health Survey
Survey Code:	PT 01
Year:	1995
Type:	Face to Face

Question 2

DO YOU DO THINGS THAT REQUIRE LITTLE PHYSICAL ACTIVITY, SUCH AS READING, KNITTING, WATCHING TV ETC.?

Yes
No
Don't know

Question 3

HOW MANY DAYS PER WEEK?

… days
Don't know

Question 4

AND FOR HOW MANY MINUTES EACH DAY ON AVERAGE?

… minutes
Don't know

Country:	United Kingdom
Survey:	Health Survey for England
Survey Code:	UK 09
Year:	1998
Type:	Face to Face + self administered

Question 153

Did you spend time sitting down doing any things like these for at least 5 minutes a time at the weekend, that is last Saturday and Sunday?

Show card J

Yes
No

Question 154

Was that on Saturday or Sunday or on both days?

Saturday only
Sunday only
Both Saturday and Sunday

Question 155

On Saturday/Sunday when you spent time sitting down doing things like these, how long did you spend daily?

Show card G

5 minutes, less than 15 minutes
15 minutes, less than 30 minutes
30 minutes, less than 1 hour
1 hour, less than 1½ hours
1½ hours, less than 2 hours
2 hours, less than 2½ hours
2½ hours, less than 3 hours
3 hours, less than 3½ hours
3½ hours, less than 4 hours
4 hours or more

Question 156

How long did you spend sitting down doing things like these?

… hours … minutes

Question 157

On how many weekdays last week did you spend time sitting down doing things like the ones on this card for at least 5 minutes a time, (not counting things you did as part of school lessons)?

None in last week
1 day
2 days
3 days
4 days
5 days

Question 158

On each weekday that you spent time sitting down doing things like these, how long did you spend?

Show card G

5 minutes, less than 15 minutes
15 minutes, less than 30 minutes
30 minutes, less than 1 hour
1 hour, less than 1½ hours
1½ hours, less than 2 hours
2 hours, less than 2½ hours
2½ hours, less than 3 hours
3 hours, less than 3½ hours
3½ hours, less than 4 hours
4 hours or more

Question 159

How long did you spend sitting down doing things like these?
s
… hours … minutes

Country: Italy

Survey: Health Conditions and the Use of Health Services

Survey Code: IT 01

Year: 1999

Type: Face to Face

Question 15.5

Is your free time usually spent doing a sedentary activity (watching television, reading magazines and/or books, etc..)?

No
Yes, over 4 hours per week
Yes, 4 hours per week
Yes, less than 4 hours per week

Country:	International
Survey:	European Physical Activity Surveillance System (EUPASS)
Survey Code:	
Year:	2000
Type:	Telephone

<u>Question B4a</u>

The next questions are about the time you spend sitting each day while at work, at home, while doing course work and during leisure time. This may include time spent sitting at a desk, visiting friends, reading or sitting or lying down to watch television.

During the last 7 days, how much time in total did you usually spend sitting on a week day?

____ hours ___ minutes

<u>Question B4b</u>

[Interviewer clarification: Include time spent lying down (awake) as well as sitting]

[Interviewer probe: An average time per day is being sought. If the respondent can't answer because the pattern of time spent varies widely from day to day, ask:
How much time in total would you spend sitting in a usual week?

____ hours ___ minutes per week [skip to end]

During the last 7 days, how much time in total did you usually spend sitting on a weekend day?

____ hours ___ minutes

Country: Croatia

Survey: Health Interview Survey

Survey Code: HR 02

Year: 2001

Type: Face to Face

Question 91

Now think about how much time you spent sitting in the last 7 days. Include time spent at work, at home, during education and leisure time. This includes time while sitting at a desk, visiting friends, reading or sitting/lying down to watch television.

How much time, you usually spend sitting, at one average weekday during the last week?

... hours ... minutes

Country:	Macedonia
Survey:	EUROHIS of Republic of Macedonia
Survey Code:	MK 03
Year:	2001
Type:	Face to Face

Question 91

Now think about the time you spent sitting on weekdays during the past 7 days. Include time spent at work, at home, while doing course work and during leisure time. This may include time spent sitting at a desk, visiting friends, traveling on a bus, reading or sitting or lying down to watch television.

During the past 7 days, how much time in total did you usually spend sitting on a weekday?

... hours ... minutes

Interviewer probe: An average time per day is being sought. If the respondent can’t answer because the pattern of time spent varies widely from day to day, ask: "What is the total amount of time you spent sitting last Wednesday?"

... hours ... minutes

Country:	Slovenia
Survey:	CINDI Health Monitor Questionnaire
Survey Code:	SI 01
Year:	2001
Type:	???

<u>Question 68</u>

On any one weekday during the last 7 days how much time did you usually spend sitting? Include time spent sitting at work, when travelling or visiting friends, and when sitting/lying down while reading or watching television.

... hours ... minutes/day

(An average time per day is being sought. If you can’t answer because the pattern of time spent varied widely from day to day what was the total amount of time you spent sitting last Wednesday?)

Country:	International
Survey:	World Health Surveys-WHS-Consolidated multi-country questionnaire
Survey Code:	INT 08
Year:	2002
Type:	Face to Face

Question 4403

Next question (4403) is about the time you spent sitting while at work, at home, while doing course work and during leisure time. This includes time spent sitting at a desk, visiting friends, reading travelling on a bus or sitting or lying down to watch television.

During the last 7days, how much time in total did you usually spend on one of those days sitting ?

Hours/minutes

Travel-related Activity
Now I am going to ask you about the way you travel to and from places (work, market church etc.).

Country:	Malta
Survey:	First National Health Interview Survey
Survey Code:	MT 01
Year:	2002
Type:	Face to Face

Question 59

Now think about the time you spend sitting on weekdays during the past 7 days.
Include time spent at work, at home, while doing course work and during leisure time.
This may include time spent sitting at a desk, visiting friends, travelling in a car or bus, reading or sitting or lying down to watch television.

During the past 7 days, how much time in total did you usually spend sitting on a weekday (Do not include Saturdays and Sundays and the time spent sleeping)?

... hours ... minutes

Country:	Slovakia
Survey:	Health Monitor Survey
Survey Code:	SK 02
Year:	2002
Type:	Face to Face

Question 6.10

How much time do you spend sitting on a usual week day? Include sitting at the desk, visiting friends, reading, travelling on a bus or sitting or lying down to watch television.

... hours ... minutes

Country:	Belgium
Survey:	Health Interview Survey
Survey Code:	BE 05
Year:	2004
Type:	Self administered

Question PA10

Sitting

During the last 7 days, how much time did you spend sitting on a week day?

... hour
... minutes/day
I don’t know

Country:	International (Finland, Baltic States)
Survey:	FINBALT Health Monitor
Survey Code:	
Year:	2004
Type:	Self administered

Question 31

How much time do you spend sitting on a usual week day? Include sitting at the desk, visiting friends, reading, travelling on a bus or sitting or lying down to watch television.

… hours … minutes

Country: Denmark

Survey: The Danish Health Interview Survey

Survey Code: DK 03

Year: 2005

Type: Face to Face

Question 23c

How much time during the last 7 days have you spent sitting while at work, at home, while doing course work and during leisure time? This includes time spent sitting at a desk, visiting friends, reading, travelling on a bus or sitting or lying down to watch television

Typical time per day:
Hours: ...
Minutes: ...

Country: France

Survey: Health Barometer

Survey Code: FR 14

Year: 2005

Type: Telephone

Question Q80

Finally, let us look at how long you spent seated during one day of the week (weekend excluded) during the past seven days. This includes time spent seated at work, at home, when studying and during your free time. This can be time seated at a desk, on transport, at friends, when reading, when seated or lying down to watch television, or when using a computer.

During the past seven days, how long did you spend seated during an average day?

… hours and … minutes a day

Question Q81

Finally, let us look at how long you spent seated during one day of the week (weekend excluded) during the past seven days. This includes time spent seated at work, at home, when studying and during your free time. This can be time seated at a desk, on transport, at friends, when reading, when seated or lying down to watch television, or when using a computer.

During the past seven days, how long did you spend seated during an average day?

… hours and … minutes a day

Question Q91

Let us finally look at the time you spent seated. This includes time spent seated at work, at home, when studying and during your free time. It can be time spent seated at a desk, on transport, at friends' houses, when reading, when seated or lying watching television, or using a computer

Yesterday, how long did you spend seated?

… hours and … minutes

Country:	Portugal
Survey:	Fourth National Health Interview Survey
Survey Code:	PT 05
Year:	2005
Type:	Face to Face

Question 0

ARE YOU] IS HE/SHE] ALWAYS BEDRIDDEN, SITTING ON A CHAIR ALL DAY OR CONFINED TO YOUR HOUSE?

Yes
No
Don’t know

Question 10

THINK ABOUT THE TIME SPENT ON WEEKDAYS IN THE LAST 7 DAYS. INCLUDE TIME SPENT AT WORK, AT HOME, DOING USUAL TASKS AND LEISURE TIME. INCLUDE ALSO THE TIME SPENT ON THINGS LIKE SITTING AT A DESK, VISITING FRIENDS, READING AND SITTING OR RESTING WHILE WATCHING TV OR LISTENING TO MUSIC.
(IT COMPREHENDS THE TIME SPENT LAYING DOWN WHILE AWAKE)

IN TOTAL, HOW MUCH TIME DID YOU/HE/SHE USUALLY SPEND SITTING IN ONE OF THESE WEEKDAYS, IN THE LAST 7 DAYS?

... h ... m per day
Don’t know because the pattern varies a lot day to day

Question 11

IN TOTAL, HOW MUCH TIME DID YOU/HE/SHE SPEND SITTING LAST WEDNESDAY?

... h ... m on Wednesday
Don’t know

Country:	United Kingdom
Survey:	Health Survey for England
Survey Code:	UK 31
Year:	2005
Type:	Face to Face

Question WESitDo

Now I'd like to know about when you/(name of child) spend time sitting down doing things like the ones on this card. Did you/s/he spend time sitting down doing any things like these for at least 5 minutes a time at the weekend

Yes
No

Question DSitWE

Was that on Saturday or Sunday or on both days?

Saturday only
Sunday only
Both Saturday and Sunday

Question SitWE

When you/(name of child) spent time sitting down doing things like these, how long did you/s/he spend on each day Please give an answer from this card.

Less than 5 minutes
5 minutes, less than 15 minutes
15 minutes, less than 30 minutes
30 minutes, less than 1 hour
1 hour, less than 1 1/2 hours
1 1/2 hours, less than 2 hours
2 hours, less than 2 1/2 hours
2 1/2 hours, less than 3 hours
3 hours, less than 3 1/2 hours
3 1/2 hours, less than 4 hours
4 hours or more (please specify how long)

Question WeSith

How long did you/(name of child) spend sitting down doing things like these?

...

Question WkSitDo

On how many weekdays last week did you/(name of child) spend time sitting down doing things like the ones on this card for at least 5 minutes a time?

None in last week,
1 day,
2 days,
3 days,
4 days,
5 days.

Question WkSitHrs

On each weekday that you/(name of child) spent time sitting down doing things like these, how long did you/s/he spend? Please give an answer from this card:

Less than 5 minutes
5 minutes, less than 15 minutes
15 minutes, less than 30 minutes
30 minutes, less than 1 hour
1 hour, less than 1 1/2 hours
1 1/2 hours, less than 2 hours
2 hours, less than 2 1/2 hours
2 1/2 hours, less than 3 hours
3 hours, less than 3 1/2 hours
3 1/2 hours, less than 4 hours
4 hours or more (please specify how long)

Question WkSitH

How long did you/(name of child) spend sitting down doing things like these?

...

Country: Serbia

Survey: National Health Survey, Serbia 2006

Survey Code: CS 01

Year: 2006

Type: Face to Face + self administered

Question SV8

Now think about time you spent sitting during the work days in the last 7 days. This includes sitting at school/work and at home. This includes sitting at a desk, table, sitting/lying while watching TV, reading, visiting friends, travelling by car, bus.

How many hours did you spend sitting during the work days in the last week?

... hours ... minutes

Question SV12

Now think about time you spent sitting during the work days in the last 7 days. This includes sitting at school/work and at home. This includes sitting at a desk, table, sitting/lying while watching TV, reading, visiting friends, travelling by car, bus.

How many hours did you spend sitting during the work days in the last week?

... hours ... minutes

Country: Estonia

Survey: Estonian Health Interview Survey 2006

Survey Code: EE 08

Year: 2006

Type: Face to Face

Question T20

Are you physically inactive for more than two hours in a row (for example reading, sitting at the computer, watching TV)?

Yes
No

Question T21

How many hours a week are you usually physically inactive (for example reading, sitting at the computer, watching TV)?

... hours

Country: France

Survey: National survey on nutrition and health (ENNS)

Survey Code: FR 18

Year: 2006

Type: Face to Face

Question E53

The following questions concern the physical activity and sedentariness of your child during the past week, that is, on the seven days including yesterday.

It is possible that you do not know exactly how to answer the questions; try to be as close as possible to what you have seen.
II. During the past week, how much time did your child spend watching television in all (video cassette and DVD included)?

For children attending neither school or crèche, or interviewed during holidays

Total time per day: … Hours … minutes
Refusal
Doesn't know

Question E54

The following questions concern the physical activity and sedentariness of your child during the past week, that is, on the seven days including yesterday.

It is possible that you do not know exactly how to answer the questions; try to be as close as possible to what you have seen.
II. During the past week, how much time did your child spend watching television in all (video cassette and DVD included)?

For children attending school (or a crèche)

School days (Monday, Tuesday, Thursday, Friday) … Hours … minutes
Days with little school (Wednesday or Saturday) … Hours … minutes
Days with no school (Wednesday or Saturday, Sunday) … Hours … minutes
Refuses to answer
Doesn't know

Country:	United Kingdom
Survey:	Health Survey for England
Survey Code:	UK 33
Year:	2006
Type:	Face to Face

Question WESitDo-DSit

Now I'd like to know about when you/(name of child) spend time sitting down doing things like the ones on this card. Did you/s/he spend time sitting down doing any things like these for at least 5 minutes a time at the weekend

Yes
No

Was that on Saturday or Sunday or on both days?

Saturday only
Sunday only
Both Saturday and Sunday

Question SitWE-WeSitH

When you/(name of child) spent time sitting down doing things like these, how long did you/s/he spend on each day

Less than 5 minutes
5 minutes, less than 15 minutes
15 minutes, less than 30 minutes
30 minutes, less than 1 hour
1 hour, less than 1 1/2 hours
1 1/2 hours, less than 2 hours
2 hours, less than 2 1/2 hours
2 1/2 hours, less than 3 hours
3 hours, less than 3 1/2 hours
3 1/2 hours, less than 4 hours
4 hours or more, please specify how long: ...

How long did you/(name of child) spend sitting down doing things like these?
RECORD HOURS SPENT BELOW.

...

RECORD HERE MINUTES SPENT SITTING DOWN DOING THINGS LIKE THESE

...

Question WkSitDo

On how many weekdays last week did you/(name of child) spend time sitting down doing things like the ones on this card for at least 5 minutes a time?

None in last week,
1 day,
2 days,
3 days,
4 days,
5 days.

Question WkSitHrs

On each weekday that you/(name of child) spent time sitting down doing things like these, how long did you/s/he spend?

Less than 5 minutes
5 minutes, less than 15 minutes
15 minutes, less than 30 minutes
30 minutes, less than 1 hour
1 hour, less than 1 1/2 hours
1 1/2 hours, less than 2 hours
2 hours, less than 2 1/2 hours
2 1/2 hours, less than 3 hours
3 hours, less than 3 1/2 hours
3 1/2 hours, less than 4 hours
4 hours or more, please specify how long: ...

How long did you/(name of child) spend sitting down doing things like these?

...

Country: Finland

Survey: FINRISK 2007

Survey Code: FI 21

Year: 2007

Type: Self administered

Question 86

How many hours on average do you sit in a weekday?

During the workday in office or equivalent ... t ... min
At home watching television or videos ... t ... min
At home at a computer ... t ... min
In a vehicle ... t ... min
Elsewhere ... t ... min

Country: Iceland

Survey: Health and wellbeing of Icelanders

Survey Code: IS 04

Year: 2007

Type: Self administered

Question 53

As a general rule, how much time a day did you spend sitting down in the previous week. This applies only to weekdays. Include the time that you spend sitting down at work, at home and during leisure. (This could include time spent sitting down at a desk, dinner table, visiting, reading or in front of the television or computer.)

Less than one hour a day
About 1 hour a day
About 2-3 hours a day
About 4-5 hours a day
About 6-7 hours a day
About 8-10 hours a day
About 11-13 hours a day
About 14-16 hours a day
More than 16 hours a day

Country: United Kingdom

Survey: Health Survey for England

Survey Code: UK 35

Year: 2007

Type: Face to Face + self administered

Question WESitDo-DSit

Now I'd like to know about when (you/child's name) spend(s) time sitting down doing things like the ones on this card. Did (you/child's name) spend time sitting down doing any things like these for at least 5 minutes a time at the weekend?

Yes
No

Was that on Saturday or Sunday or on both days?

Saturday only
Sunday only
Both Saturday and Sunday

Question SitWE-WeSitH

On (Saturday/Sunday) when (you/child's name) spent time sitting down doing things like these, how long did (you/he/she) spend on each day.

15 minutes, less than 30 minutes
30 minutes, less than 1 hour
1 hour, less than 1 1/2 hours
1 1/2 hours, less than 2 hours
2 hours, less than 2 1/2 hours
2 1/2 hours, less than 3 hours
3 hours, less than 3 1/2 hours
3 1/2 hours, less than 4 hours
4 hours or more (please specify how long)

More than four hours THEN

How long did (you/child's name) spend sitting down doing things like these on (each/that) day?
RECORD HOURS SPENT BELOW.

...

RECORD HERE MINUTES SPENT SITTING DOWN DOING THINGS LIKE THESE

...

Question WkSitDo

On how many weekdays last week did (you/child's name) spend time sitting down doing things like the ones on this card for at least 5 minutes a time not counting things (you/he/she) did as part of school lessons?

None in last week
1 day
2 days
3 days
4 days

Question WkSitHrs

On each weekday that (you/child's name) spent time sitting down doing things like these, how long did (you/he/she) spend?

15 minutes, less than 30 minutes
30 minutes, less than 1 hour
1 hour, less than 1 1/2 hours
1 1/2 hours, less than 2 hours
2 hours, less than 2 1/2 hours
2 1/2 hours, less than 3 hours
3 hours, less than 3 1/2 hours
3 1/2 hours, less than 4 hours
4 hours or more (please specify how long)

More than four hours THEN

How long did (you/child's name) spend sitting down doing things like these?

...

Country: United Kingdom

Survey: Health Survey for England

Survey Code: UK 36

Year: 2008

Type: Face to Face

Question WESitDo-DSitWESitDo

Now I'd like to know about when (you/child's name) spend(s) time sitting down
doing things like the ones on this card. Did (you/child's name) spend time sitting down
doing any things like these for at least 5 minutes a time at the weekend?

Yes
No

Was that on Saturday or Sunday or on both days?

Saturday only
Sunday only
Both Saturday and Sunday

Question SitWE-WeSitH

On (Saturday/Sunday) when (you/child's name) spent time sitting down doing things like
these, how long did (you/he/she) spend on each day.

15 minutes, less than 30 minutes
30 minutes, less than 1 hour
1 hour, less than 1 1/2 hours
1 1/2 hours, less than 2 hours
2 hours, less than 2 1/2 hours
2 1/2 hours, less than 3 hours
3 hours, less than 3 1/2 hours
3 1/2 hours, less than 4 hours
4 hours or more (please specify how long)

More than four hours THEN

How long did (you/child's name) spend sitting down doing things like these on (each/that)
day?
RECORD HOURS SPENT BELOW.

...

RECORD HERE MINUTES SPENT SITTING DOWN DOING THINGS LIKE
THESE

...

Question WkSitDo

On how many weekdays last week did (you/child's name) spend time sitting down doing things like the ones on this card for at least 5 minutes a time not counting things (you/he/she) did as part of school lessons?

None in last week
1 day
2 days
3 days
4 days

Question WkSitHrs

On each weekday that (you/child's name) spent time sitting down doing things like these, how long did (you/he/she) spend?

15 minutes, less than 30 minutes
30 minutes, less than 1 hour
1 hour, less than 1 1/2 hours
1 1/2 hours, less than 2 hours
2 hours, less than 2 1/2 hours
2 1/2 hours, less than 3 hours
3 hours, less than 3 1/2 hours
3 1/2 hours, less than 4 hours
4 hours or more (please specify how long)

More than four hours THEN

How long did (you/child's name) spend sitting down doing things like these?

...

International Survey Items concerning

generic and other physical activity questions

1992 - 2008

TABLE OF CONTENTS

1992
United Kingdom – UK 03 3

1994
Denmark – DK 01 6

1995
Portugal – PT 01 7
United Kingdom – UK 10 9

1996
Finland – FI 05 12
International – INT 03 13
Luxembourg – LU 01 14

1997
Belgium – BE 01 15
Switzerland – CH 01 16

1998
Germany – DE 05 17
Ireland – IE 01 18
United Kingdom – UK 02 20
United Kingdom – UK 09 23
United Kingdom – UK 13 28

1999
Estonia – EE 05 30
Italy – IT 01 31
Lithuania – LT 01 32
Latvia – LV 01 33
Portugal – PT 03 34

2000
Canada – CA 01 35
Cyprus – CY 02 36
Denmark – DK 02 37
Estonia – EE 06 38
Finland – FI 01 39
Finland – FI 03 40
Hungary – HU 01 42
International – EUPASS 43
Italy – IT 03 48
Romania – RO 01 49
United Kingdom – UK 12 50
United States – US 01 52

2001
Australia – AU 01 55
Belgium – BE 02 57
Finland – FI 06 59
Finland – FI 09 60
Croatia – HR 02 62
Iceland – IS 03 64
Italy – IT 04 65
Macedonia – MK 03 66
Netherlands – NL 02 68
Netherlands – NL 03 69
Slovenia – SI 02 71

2002
Switzerland – CH 02 73
Finland – FI 07 75
Finland – FI 13 76
France – FR 09 77
Ireland – IE 03 78
International – INT 06 79
International – INT 08 81
Italy – IT 05 83
Lithuania – LT 02 84
Latvia – LV 03 86
Malta – MT 01 88
Netherlands – NL 05 91
Denmark – REC 02 92
Slovakia – SK 02 95

United Kingdom – UK 07 97
United Kingdom – UK 28 98

2003

Canada – CA 02 101
Spain – ES 03 102
Finland – FI 10 103
Finland – FI 11 104
Croatia – HR 03 106
Croatia – HR 04 110
Hungary – HU 05 111
Latvia – LV 02 113
Netherlands – NL 08 114
Sweden – SE 03 115
Turkey – TR 01 116
Turkey – TR 05 117

2004

Australia – AU 03 121
Belgium – BE 122
Estonia – EE 07 124
Finland – FI 12 125
International – FINBALT 126
Netherlands – NL 07 128
Sweden – SE 04 129

2005

Canada – CA 03 130
Germany – DE 07 131
Denmark – DK 03 132
Finland – FI 19 134
Finland – FI 20 135
France – FR 14 137
Italy – IT 08 140
Lithuania – LT 06 141
Poland – PL 05 143
Portugal – PT 05 144
Sweden – SE 05 147
United Kingdom – UK 31 148

2006

Austria – AT 06 150
Serbia – CS 01 153
Germany – DE 08 156
Estonia – EE 08 157
Spain – ES 06 160
France – FR 18 161
Lithuania – LT 05 165
International – REC 04 166
United Kingdom – UK 33 168
United States – US 04 171

2007

Australia – AU 04 172
Canada – CA 04 175
Canada – CA 05 176
Switzerland – CH 03 177
Germany – DE 09 178
Finland – FI 21 181
Finland – FI 22 182
Finland – FI 23 183
Ireland – IE 09 185
Iceland – IS 04 189
Luxembourg – LU 04 192
Sweden – SE 09 193
Sweden – LNU 194
Slovakia – SK 04 195
United Kingdom – UK 35 197

2008

Belgium – BE 06 202
Bulgaria – BG 04 204
Cyprus – CY 03 206
Czech Republic – CZ 04 208
Latvia – LV 06 210
Malta – MT 02 212
Romania – RO 02 214
Turkey – TR 06 216

Country:	United Kingdom
Survey:	Health and Lifestyle Survey
Survey Code:	UK 03
Year:	1992
Type:	Face to Face

Question 82

a. Overall, do you think that you get enough exercise?

Yes
No
Don't know

b. In general, compared with men/women of your own age, are you physically

more active
less active
or, about average
Don’t know

Question 83

a. On weekdays (working days) when not at work, how much time on average per day do you spend walking – getting work to work, shopping, walking the dog, for pleasure and so on?

… time per day
Don't know

b. At weekends (rest days) how much time on average per day do you spend walking?

… time per day
Don’t know

Question 84

Compared to people of your own age, which of the following best describes your usual walking pace?

slow
average
fairly brisk
or fast

It depends/Don't know

Question 88

a. Have you done any other physical activities in the last fortnight?

Yes
No

b. What did you do?

...

c. How many times have you in the last fortnight?

… times

d. On average, how long did you spend doing it each time?

…

Question 89

a. Compared with 7 years ago, do you nowadays spend more, less or about the same amount of time on sport and physical activities?

Now spend more time
Now spend less time
About the same time
Don’t know

b. Would you say that you now spend

a bit more time
or a lot more time
Can't say

Question 90

Why do you now spend more time than seven years ago on sport or physical activities?

Will power/to get or keep fit
More leisure time
To take part with family
More money
Better facilities
Better health
Other, please specify: ...
Can't say

Question 91

a. Would you say that you now spend

a bit less time
or a lot less time
Can't say

b. Why do you spend less time on sport or physical activities than seven years ago?

Family ties
Less leisure time
Companions not available
Less money
Less facilities
Poor health/injuries
Other, please specify: ...
Can't say

Country: Denmark

Survey: Danish Health and Morbidity Survey

Survey Code: DK 01

Year: 1994

Type: Face to Face

Question 79

What do you do to stay healthy or to improve your health?

several answers acceptable

I don't do anything special
I exercise/am physically active
I eat healthy food
I eat less
I don't smoke
I try to quit smoking/smoke less
I don't drink alcohol
I try to limit my alcohol consumption
I try to live less stressed
I try to get enough sleep
I keep in touch with my family, friends and acquaintances
other, what: …
no information

Question 80

If we look back on the past year, what would you say best describes your spare time activities?

Show card 14

heavy training and competitive sports regularly and several times a week
exercise or heavy gardening at least 4 hours a week
walk, bike or other easy exercise at least 4 hours a week (include Sunday excursions, light gardening and biking/walking to work
read, watch TV or other sedentary occupation
don't know
no information

Country: Portugal

Survey: National Health Survey

Survey Code: PT 01

Year: 1995

Type: Face to Face

Question 1

WHICH OF THE FOLLOWING BEST DESCRIBES THE AMOUNT OF EXERCISE YOU USUALLY TAKE EACH DAY?
(Show card No 9)

I tend to be sedentary and do little walking
I am on my feet quite a lot but do not have to lift or carry objects very often
I usually have to lift or carry light objects or go up and down stairs several times a day
I do heavy physical work or carry very heavy loads
I don't know

Question 2

DO YOU DO THINGS THAT REQUIRE LITTLE PHYSICAL ACTIVITY, SUCH AS READING, KNITTING, WATCHING TV ETC.?

Yes
No
Don't know

Question 3

HOW MANY DAYS PER WEEK?

… days
Don't know

Question 4

AND FOR HOW MANY MINUTES EACH DAY ON AVERAGE?

… minutes
Don't know

Question 5

DO YOU TAKE LIGHT EXERCISE SUCH AS WALKING,
CYCLING ETC.?

Yes
No
Don't know

Question 6

HOW MANY DAYS PER WEEK?

… days
Don't know

Question 7

AND FOR HOW MANY MINUTES EACH DAY ON AVERAGE?

… minutes
Don't know

Country:	United Kingdom
Survey:	The Scottish Health Survey
Survey Code:	UK 10
Year:	1995
Type:	Face to Face + self administered

Question 149

I'd like to ask you about some of the things you do at work or in your free time that involve physical activity. Which of the activities on this card would you normally take part in during an average week?

SHOW CARD B

Cycling/exercise bike
Exercises (press ups, sit ups, etc)
Aerobics/keep fit/gymnastics/dance for fitness
Other types of dancing
Weight training
Swimming
Running/jogging
Football/rugby
Badminton/tennis
Squash
Golf/hill walking
Other walking of 1 mile or more
Other, 'Which others?' ...
None of these

Question 151

On how many occasions per week do you usually go cycling/use your exercise bike?

Less than once a week
Once a week
2-3 times a week
4-5 times a week
6-7 times a week or more

Question 152

How much time do you usually spend cycling/using your exercise bike on each occasion?

Less than 10 minutes
10 minutes, less than 20 minutes
20 minutes, less than 30 minutes
30 minutes, less than 2 hours
2 hours or longer

Question 153

When you go cycling/use your exercise bike do you usually find yourself

breathing normally,
breathing faster than normal,
or gasping for breath?

Question 184

On how w many occasions per week do you usually go for walks of 1 mile or more?

Less than once a week
Once a week
2-3 times a week
4-5 times a week
6-7 times a week or more

Question 185

How much time do you usually spend walking?

Less than 10 minutes
10 minutes, less than 20 minutes
20 minutes, less than 30 minutes
30 minutes, less than 2 hours
2 hours or longer

Question 186

When you go walking do you usually find yourself

breathing normally,
breathing faster than normal,
or gasping for breath?

Question 200

Do you think you get enough exercise for your age and health?

Yes
No

Question 201

Would you like to take more exercise?

Yes
No

Question 203

Would you consider taking more exercise for any of the reasons shown on this card?

SHOW CARD E.
What other reason?

To feel healthier or fitter
To lose weight
To look better
To prevent disease or ill-health
To enjoy myself
To reduce stress
None of these reasons

Question 353

Do you mainly take the lift or climb the stairs?

Lift
Stairs
Lift up/stairs down

Country: Finland

Survey: Finnish Health Care Survey

Survey Code: FI 05

Year: 1996

Type: Face to Face

Question C6K9

HOW OFTEN DO YOU ENGAGE IN PHYSICAL ACTIVITY FOR AT LEAST HALF AN HOUR SO THAT YOU ARE AT LEAST MILDLY SHORT OF BREATH AND SWEATING?

Daily
2 - 5 times a week
Once a week
2 - 3 times a month
About once a month or less frequently
Never, because of an illness

Question C7K6

TAKING YOUR BREATHING INTO ACCOUNT, TO WHAT EXTENT CAN YOU TOLERATE PHYSICAL EXERCISE?

WELL,
FAIRLY WELL
NOT AT ALL?

Country: International

Survey: Eurobarometer

Survey Code: INT 03

Year: 1996

Type: Face to Face

Question 129

Here are some factors which may be linked to a lesser or greater extent to heart disease. In your opinion, which is the main factor?
And which comes second?

SHOW CARD

Main factor
Second factor

Unhealthy diet
Lack of exercise
Excessive alcohol consumption
Stress
Being overweight
Don't know

Country:	Luxembourg
Survey:	Panel Living in Luxembourg
Survey Code:	LU 01
Year:	1996
Type:	Face to Face

Question 49

To look after your health, do you:

do some sports?
avoid smoking
undergo a regular medical check-up

YES
NO

Country: Belgium

Survey: Health Interview Survey

Survey Code: BE 01

Year: 1997

Type: Face to Face

Question AP.02

For at least once a week, do you engage in any regular physical activity (such as jogging, cycling,. etc.) long enough to work up sweat?

Yes, ... days/week
No

Country: Switzerland

Survey: Swiss Health Survey

Survey Code: CH 01

Year: 1997

Type: Face to Face

Question 48.21

Do you normally walk and/or use your bicycle for 20
minutes or more every day?

Yes
No
Don’t know
No reply

Question 48.30

How often do you work up a sweat at work, i.e. at
your place of work or doing housework or gardening?

Very frequently
Frequently
Occasionally
Rarely
Very rarely
Never
No reply

Country: Germany

Survey: German National Health Examination and Interview Survey

Survey Code: DE 05

Year: 1998

Type: ???

Question 62

What is the average time per day (24 hours) you spend doing the following:

Monday-Friday ... hours (total=24,0 hours)
Saturday/Sunday ... hours (total=24,0 hours)

Sleeping, relaxing
Sitting down (e.g. in the office, in the car, watching TV, or reading)
Light activities (e.g. cooking, walking, shopping, tidying up, personal hygiene, sales)
Moderate activities (e.g. jogging, decorating, cleaning, cycling, swimming, building work)
Strenuous activity (e.g. carrying heavy things, strenuous gardening, chopping wood, competitive sport, ball sports)

Question 64

When you climb three flights of stairs are you (normally) out of breath and/or do you sweat?

Yes
No
Unable to climb 3 flights of stairs

Country: Ireland

Survey: Survey of Lifestyle, Attitudes and Nutrition (SLÁN)

Survey Code: IE 01

Year: 1998

Type: Self administered

Question B1

Considering a 7-day period (a week), how many times on average do you do the following kinds of exercise for more than 20 minutes during your free time?

a. STRENUOUS EXERCISE (HEART BEATS RAPIDLY) (e.g. running, jogging, hockey, football, soccer, squash, basketball, judo, roller skating, vigorous swimming, vigorous long distance cycling)

… Times per Week

b. MODERATE EXERCISE (NOT EXHAUSTING) (e.g. fast walking, tennis, badminton, easy swimming, easy cycling, volleyball, baseball, popular and folk dancing, heavy gardening)

… Times per Week

c. MILD EXERCISE (MINIMAL EFFORT) (e.g. yoga, golf, easy walking, fishing from river bank, bowling, archery, light gardening)

… Times per Week

Question B2

At least once a week do you engage in any regular activity, such as jogging or cycling long enough to work up sweat?

No
Yes

If yes, how many days per week?

… days

Question B3

Do you do light household work? (e.g. dusting, washing dishes, repairing clothes)

- Seldom/Never
- 1-3 times per month
- Once per week
- 3-4 times per week
- Most days

Question B4

Do you do heavy household work? (e.g. vacuuming/ hoovering, washing floors and windows, carrying rubbish bags)

- Seldom/Never
- 1-3 times per month
- Once per week
- 3-4 times per week
- Most days

Country: United Kingdom

Survey: Health Education Monitoring Survey

Survey Code: UK 02

Year: 1998

Type: Face to Face

Question 104

Some kinds of housework are heavier than others. This card gives examples of heavy housework, it does not include everything, these are just examples. Was any of the housework you did in the past 4 weeks this kind of heavy housework?

SHOW CARD Y

Yes
No

During the past 4 weeks on how many separate days have you done that kind of heavy housework?

Question 105

Have you done any gardening, did you or building in the past 4 weeks?

Yes
No

Have you done any gardening, did you or building work listed on this card?

SHOW CARD Z

Yes
No

Have you done any gardening, did you or building work from this card, or any similar heavy manual work?

SHOW CARD AA

Yes
No

During the past 4 weeks, on how many days have you done this kind of heavy manual gardening or did you?

Question 106

I'd like you to think now about all the walking you've done in the past 4 weeks, either locally or away from here. Include any country walks and any walking to and from work, and any other walks that you have done.

In the past four weeks have you done any walks that lasted between 5 and 10 minutes?.

Yes
No
Can't walk at all/ Housebound

Did you do any walks that lasted for at least 15 minutes but less than 30 minutes?

Yes
No

During the last 4 weeks, how many times have you done a walk that lasted for at least 15 minutes but less than 30 minutes?

… times

Did you do any walks that lasted for 30 minutes or more?

Yes
No

During the past 4 weeks how many times did you do any walks lasting for 30 minutes or more?

… times

Which of the following best describes your usual walking pace

a slow pace
a steady average pace
a fairly brisk pace
or a fast pace - at least 4 mph?

Question 110

Now I would like you to look at this card, and say which of the statements best describes you.

SHOW CARD CC

I intend to take more exercise within the next month
I intend to take more exercise within the next six months
I intend to take more exercise within the next year
I intend to take more exercise, but not in the next year

I'm unlikely to take more exercise

Question 111

Now I would like you to look at this card, and say which of the statements best describes you.

SHOW CARD DD

I'm unlikely to take more exercise
I intend to take more exercise within the next month
I intend to take more exercise within the next six months
I intend to take more exercise within the next year
I intend to take more exercise but not in the next year

Country: United Kingdom

Survey: Health Survey for England

Survey Code: UK 09

Year: 1998

Type: Face to Face + self administered

Question 128

Now I'd like to ask you about some of the things you have done in the last week. By last week I mean in the seven days up to yesterday.

In the last week, have you done a continuous walk that lasted at least 5 minutes
(not counting things done as part of school lessons)?

Yes
No

Question 129

On how many days in the last week did you do a continuous walk that lasted at least 5 minutes (not counting things done as part of school lessons)?

One day
Two days
Three days
Four days
Five days
Six days
Every day

Question 130

On each day that you did a walk like this for at least 5 minutes, how long did you spend walking altogether? Please give an answer from this card.

Show card G

5 minutes, less than 15 minutes
15 minutes, less than 30 minutes
30 minutes, less than 1 hour
1 hour, less than 1½ hours
1½ hours, less than 2 hours
2 hours, less than 2½ hours

2½ hours, less than 3 hours
3 hours, less than 3½ hours
3½ hours, less than 4 hours
4 hours or more

Question 131

How long did you spend walking on each day?

… hours … minutes

Question 132

Which of the following describes your usual walking pace

a slow pace,
a steady average pace,
a fairly brisk pace,
or, a fast pace - at least 4 mph?
None of these

… days

Question 137

In the last week, that is last (day) up to yesterday, have you done any sports or exercise activities (not counting things done as part of school lessons)?

Show card H

Yes
No

Question 149

Still thinking about last week. On how many of the weekdays did you do active things, like the things on this card or other activities like these (not counting things done as part of school lessons)?

Show card I

None in last week
1 day
2 days
3 days
4 days
5 days

Question 150

On each weekday that you did active things like these, how long did you spend?

Show card G

Less than 5 minutes
5 minutes, less than 15 minutes
15 minutes, less than 30 minutes
30 minutes, less than 1 hour
1 hour, less than 1½ hours
1½ hours, less than 2 hours
2 hours, less than 2½ hours
2½ hours, less than 3 hours
3 hours, less than 3½ hours
3½ hours, less than 4 hours
4 hours or more

Question 151

How long did you spend doing active things like these on each weekday?

… hours … minutes

Question 152

Now thinking about all the activities during the past week you have just told me about including any walking, (gardening, housework,) sports or other active things.

On how many days in the last week in total did you do any of these activities (not counting things done as part of school lessons)?

None
One day
Two days
Three days
Four days
Five days
Six days
Every day

Question 160

Were the activities you did last week different from what

you would usually do for any reason?
Would you usually do more physical activity or less?

NO - same as usual
YES DIFFERENT - usually do MORE
YES DIFFERENT - usually do LESS

Question 176

I'd like you to think about all the walking you have done in the past 4 weeks either locally or away from here. Please include any country walks, walking to and from work and any other walks that you have done. In the past four weeks, that is since (date four weeks ago), have you done a continuous walk that lasted at least 5 minutes?

Yes
No
Can't walk at all

Question 177

In the past four weeks, have you done a continuous walk that lasted at least 15 minutes? (That is since (date four weeks ago))

Yes
No

Question 178

During the past four weeks, on how many days did you do a walk of at least 15 minutes? (That is since (date four weeks ago))

… days

Question 179

On that day (any of those days) did you do more than one walk lasting at least 15 minutes?

Yes, more than one walk of 15+mins (on at least one day)
No, only one walk of 15+ mins a day

Question 180

On how many days in the last four weeks did you do more than one walk that lasted at least 15 minutes?

… days

Question 181

How long did you usually spend walking each time you did a walk for 15 minutes or more?

… hours … minutes

Question 182

Which of the following best describes your usual walking pace

a slow pace,
a steady average pace,
a fairly brisk pace,
or, a fast pace - at least 4 mph?

Country: United Kingdom

Survey: The Scottish Health Survey

Survey Code: UK 13

Year: 1998

Type: Face to Face

Question 180

I'd like you to think about all the walking you have done in the past 4 weeks either locally or away from here. Please include any country walks, walking to and from work and any other walks that you have done. In the past four weeks, that is since (date four weeks ago), have you done a continuous walk that lasted at least 5 minutes?

Yes
No
Can't walk at all

In the past four weeks, have you done a continuous walk that lasted at least 15 minutes?
(That is since (date four weeks ago))

Yes
No

During the past four weeks, on how many days did you do a walk of at least 15 minutes?
(That is since (date four weeks ago))

…

On that day (any of those days) did you do more than one walk lasting at least 15 minutes?

Yes, more than one walk of 15+ mins (on at least one day)
No, only one walk of 15+ mins a day

On how many days in the last four weeks did you do more than one walk that lasted at least 15 minutes?

How long did you usually spend walking each time you did a walk for 15 minutes or more?

…

RECORD HERE MINUTES SPENT WALKING.

Question 190

Now thinking about all the activities during the past week you have just told me about including any walking, (gardening, housework,) sports or other active things. On how many days in the last week in total did you do any of these activities (not counting things done as part of school lessons)?

None
One day
Two days
Three days
Four days
Five days
Six days
Every day

Were the activities you did last week different from what you would usually do for any reason?
IF YES PROBE: Would you usually do more physical activity or less?

NO - same as usual
YES DIFFERENT - usually do MORE
YES DIFFERENT - usually do LESS

Country:	Estonia
Survey:	Living Conditions Survey (NORBALT)
Survey Code:	EE 05
Year:	1999
Type:	Face to Face

Question G 12

I will now give you a list of things people can do to improve their health status. Do you on a regular basis take any of these steps in order to strengthen your health? (Several answers possible)

None of these
Try to spend much time in fresh air
Try to eat healthy and regularly
Have resting days
Drink tea from healing herbs
Walk a lot
Exercise/do gymnastics
Take vitamins regularly
Read literature about health care and health protection
No answer

Country:	Italy
Survey:	Health Conditions and the Use of Health Services
Survey Code:	IT 01
Year:	1999
Type:	Face to Face

<u>Question 15.3</u>

Do you habitually carry out a LIGHT physical, exercise activity (taking at least a one-kilometre walk, climbing stairs, etc..)?

NO
YES, over 4 hours per week
YES, 4 hours per week
YES, less than 4 hours per week

<u>Question 15.7</u>

Can your domestic work mainly be considered a physical activity?

Light
Moderate
Heavy, till perspiring

<u>Question 15.9</u>

Can your work activity mainly be considered a physical activity?

Light, I spend most of the time sitting down
Moderate, I spend most of the time standing up
Heavy, till perspiring at least half of the working hours

Country:	Lithuania
Survey:	Living Conditions Survey
Survey Code:	LT 01
Year:	1999
Type:	Face to Face

Question G 12

I will now give you a list of things people can do to improve their health status. Do you on a regular basis take any of these steps in order to strengthen your health? (Several answers possible)

None of these
Try to spend much time in fresh air
Try to eat healthy and regularly
Have resting days
Drink tea from healing herbs
Walk a lot
Exercise/do gymnastics
Take vitamins regularly
Read literature about health care and health protection
No answer

Country: Latvia

Survey: Living Conditions Survey

Survey Code: LV 01

Year: 1999

Type: Face to Face

<u>Question G 12</u>

I will now give you a list of things people can do to improve their health status. Do you on a regular basis take any of these steps in order to strengthen your health? (Several answers possible)

None of these
Try to spend much time in fresh air
Try to eat healthy and regularly
Have resting days
Drink tea from healing herbs
Walk a lot
Exercise/do gymnastics
Take vitamins regularly
Read literature about health care and health protection
No answer

Country:	Portugal
Survey:	National Health Interview Survey
Survey Code:	PT 03
Year:	1999
Type:	Face to Face

Question 1

For 15-years-old and older people

ABOUT EVERYDAY ACTIVITIES, AT WORK, AT SCHOOL,
AT HOME OR SOMEWHERE ELSE, TELL ME…

WHICH OF THE FOLLOWING SITUATIONS DESCRIBES THE BEST THE EFFORT REQUIRED BY [YOUR/HER/HIS] EVERYDAY ACTIVITIES? MENTION ONLY ONE.

(Use card No.9)

USUALLY SITTING AND FEW WALKING

STANDING UP OR WALKING QUITE ENOUGH, WITHOUT LIFTING OR CARRYING OBJECTS MANY TIMES,

LIFTING OR CARRYING LIGHT WEIGHTS OR GOING UP AND DOWN STAIRS OFTEN.

HARD PHYSICAL WORK OR CARRYING HEAVY WEIGHTS.

Don't know

BESIDES THESE QUESTIONS WE WOULD STILL LIKE TO KNOW SOMETHING ABOUT OTHER ACTIVITIES OF THIS FAMILY'S MEMBERS (15-years-old and older)

Country: Canada

Survey: Canadian Community Health Survey Cycle 1.1

Survey Code: CA 01

Year: 2000

Type: Face to Face

Question PA6

Thinking back over the past 3 months, which of the following best describes your usual daily activities or work habits?

Usually sit during the day and don’t walk around very much
Stand or walk quite a lot during the day but don’t have to carry or lift things very often
Usually lift or carry light loads, or have to climb stairs or hills often
Do heavy work or carry very heavy loads

Country: Cyprus

Survey: Population Risk Factors Survey

Survey Code: CY 02

Year: 2000

Type: Face to Face

Question 31

In which group do you belong in relation to physical activity?
Including any activity in your leisure time, like physical activity, gardening, dancing etc.

I am not physical active
Light physical activity
Vigorous activity of a minimum of 20 minutes duration for once or twice a week (sweating, heart beating, out of breath)
Vigorous activity of a minimum of 20 minutes duration for three or more times a Week

Country: Denmark

Survey: Health and Morbidity in Denmark

Survey Code: DK 02

Year: 2000

Type: Face to Face

Question 80A

Are you generally more or less physically active,
compared to 5 years ago?

More active
Unchanged
Less active
Don't know

Question 80B

Why did you change your level of physical activity?
What is the most important reason for the change?

Write:…

Country: Estonia

Survey: Time Use Survey

Survey Code: EE 06

Year: 2000

Type: Face to Face

Question I52

During the past 4 weeks did you participate in any of the following sports?
If yes, then how many times? If no, then how many times during the year.
NB! For professionals sport is work – when they are paid.

Yes
How many times: ...

No
But during the year how many times

running, jogging
cycling
skiing
swimming
gymnastics, aerobics
ball games
sailing, rowing wind-surfing
tennis
thought games
other sports which?

...

Country: Finland

Survey: Survey on health behaviour

Survey Code: FI 01

Year: 2000

Type: Self administered

Question 92

In the past year (12 months), have any of the persons mentioned below advised you to exercise more?

yes
no

physician
other health care provider
family member
other

Country: Finland

Survey: Health 2000

Survey Code: FI 03

Year: 2000

Type: Self administered

Question 29

On how many days during an ordinary week do you do something that demands strenuous physical effort at least 10 minutes at a time; e.g. running, aerobics, heavy gardening or other activity that makes you breathe faster and speeds up your heart rate (choose 0 if you do not exercise like this on any day of the week).

... days per week

Question 30

How much time altogether do you use in strenuous exercise during the days when you practice strenuous exercise at least 10 minutes at a time?

... hours ... minutes

Question 31

On how many days during an ordinary week do you do something that demands moderate physical effort at least 10 minutes at a time, such as cycling, hoovering, gardening or other activity that slightly speeds up your breath and pulse? (Do not include walking)

... days per week

Question 32

How much time during such a day do you spend exercising moderately?

... hours ... minutes

Question 33

On how many days during an ordinary week do you walk quickly at least 10 minutes at a time to move from one place to another or for recreation, satisfaction, or to keep fit?

... days a week

Question 34

How much time do you spend walking quickly on days when you walk quickly at least 10 minutes at a time?

... hours ... minutes

Country: Hungary

Survey: National Health Interview Survey

Survey Code: HU 01

Year: 2000

Type: Face to Face

Question 44

Do you regularly do physical work or sport activities (i.e.: including your work) for at least 10 minutes that cause significant sweating or increase heart rate?

Yes
No
Unable to do this type of activity
Does not know/Not sure
Refused

Question 45

And how often did you do these activities during the past 12 months?

At least once a day
Several times a week, but not every day
At least once a week
At least once a month
Less frequently
Does not know/Not sure
Refused

Country: International

Survey: European Physical Activity Surveillance System (EUPASS)

Survey Code:

Year: 2000

Type: Telephone

Question A1

Please consider for the following questions about your physical activity only the last three months.
How often do you participate in sports?

regularly, more than 4 hours per week O
regularly, 2-4 hours per week O
regularly, 1-2 hours per week O
less than 1 hour per week O
no sports activities O

Question A2

How much time on average do you spend per day (24 hours) on:
(Round up time to full or half hours – Like 0,5 Hrs. This concerns only physical activities or efforts. Please try to distribute all 24 hours over the 5 categories)

sleeping, resting:

Monday to Friday __,__ h Saturday/Sunday __,__ h

Sitting (like at the office, in the car, watching television, eating, reading):

Monday to Friday __,__ h Saturday/Sunday __,__ h

light activities (like cooking, walking at low pace, shopping, tiding up the room, body care, selling):

Monday to Friday __,__ h Saturday/Sunday __,__ h

moderate activities (like jogging, renovating, cleaning, construction work):

Monday to Friday __,__ h Saturday/Sunday __,__ h

strenuous activities (like carrying heavy weights, strenuous gardening, chopping wood, competitive sport, Ball games):

Monday to Friday __,__ h Saturday/Sunday __,__ h

Question A4

Do you normally get out of breath or into a sweat when you climb stairs over three floors?

yes
no
I am not able to climb three floors.

Question B1a

The next questions are about the time you spent being physically active in the last 7 days. They include questions about activities you do at work, to get from place to place, as part of your house and yard work, and in your spare time for recreation, exercise or sport.

Now, think about all the vigorous activities which take hard physical effort that you did in the last 7 days. Vigorous activities make you breathe much harder than normal and may include heavy lifting, digging, aerobics, or fast bicycling. Think about only those physical activities that you did for at least 10 minutes at a time.

During the last 7 days, on how many days did you do vigorous physical activities?

[Interviewer clarification: Think about only those physical activities that you do for at least 10 minutes at a time.]

[Interviewer: Include all jobs.]

________ days per week
Refused [Interviewer: Do not read]
Don't know [Interviewer: Do not read]

[Interviewer: If respondent answers zero, refuses or does not know, skip to Question 2a]

Question B1b

How much time in total did you usually spend on one of those days doing vigorous physical activities?

____ hours ___ minutes

[Interviewer clarification: Think about only those physical activities that you do for at least 10 minutes at a time.]

[Interviewer probe: An average time per day is being sought. If the respondent can't answer because the pattern of time spent varies widely from day to day, ask:
" How much time in total would you spend in a usual week doing vigorous physical activities?"

____ hours ___ minutes per week]

Question B2a

Now think about activities which take moderate physical effort that you did in the last 7 days? Moderate physical activities make you breathe somewhat harder than normal and

may include carrying light loads, bicycling at a regular pace, or doubles tennis. Do not include walking. Again, think about only those physical activities that you did for at least 10 minutes at a time.

During the last 7 days, on how many days did you do moderate physical activities?

[Interviewer clarification: Think about only those physical activities that you do for at least 10 minutes at a time.]

[Interviewer: Include all jobs.]

________ days per week
Refused [Interviewer: Do not read]
Don't know [Interviewer: Do not read]

[Interviewer: If respondent answers zero, refuses or does not know, skip to Question 3a]

Question B2b

How much time in total did you usually spend on one of those days doing moderate physical activities?

____ hours ___ minutes

[Interviewer clarification: Think about only those physical activities that you do for at least 10 minutes at a time.]

[Interviewer probe: An average time per day is being sought. If the respondent can't answer because the pattern of time spent varies widely from day to day, or includes time spent in multiple jobs, ask: How much time in total would you spend in a usual week doing moderate physical activities during a usual week?

____ hours ___ minutes per week]

Question B3a

Now think about the time you spent walking in the last 7 days. This includes walking at work and at home, walking to travel from place to place, and any other walking that you might do solely for recreation, sport, exercise or leisure.

During the last 7 days, on how many days did you walk for at least 10 minutes at a time?

[Interviewer clarification: Think about only the walking that you do for at least 10 minutes at a time.]

[Interviewer: Include all jobs.]

________ days per week
Refused [Interviewer: Do not read]
Don't know [Interviewer: Do not read]

[Interviewer: If respondent answers zero, refuses or does not know, skip to Question 4a]

Question B3b

How much time in total did you usually spend walking on one of those days?

____ hours ___ minutes

[Interviewer probe: An average time per day is being sought. If the respondent can't answer because the pattern of time spent varies widely from day to day, ask: How much time in total would you spend walking in a usual week?

____ hours ___ minutes per week

Question B3c

At what pace did you usually walk? Did you walk at:

___ a Vigorous pace, that makes you breathe much harder than normal;
___ a Moderate pace that makes you breathe somewhat harder than normal; or
___ a Slower pace where there is no change in your breathing.

[Interviewer probe: A usual pace is being sought. If the respondent can't answer because the pace varies widely from day to day, or from across job, transportation and leisure categories, ask: How much time would you spend in a usual week walking at a slow pace?

____ hours ___ minutes per week]

Question B5a

How much physical activity did you do in your place of work in the last 7 days?

Much
Moderate
Little or no

Question C1

The next statements are about being physically active regularly for 30 minutes in different situations. For each item, please indicate how sure you are that you would exercise in that situation. Give one answer for each item.

I'm sure I could
I'm rather sure I could
Maybe I could
I'm rather sure I could not
I'm sure I could not

How certain are you that you could do 30 minutes of moderate physical activity (such as brisk walking) most days of the week?

How certain are you that you could do 30

minutes of moderate physical activity if
you were sad or tired?

How certain are you that you could do 30 minutes of moderate physical activity if you had a lot of work or demands from friends or family?

Question C2

To what extent have you been motivated to do sport and physical activity ...

Very much
Much
Not much/not little
A little
Not at all

by your family or friends

by the media including newspapers, magazines, TV & radio

by local authorities and/or politicians

by health care personnel (like doctors or nurses) or an insurance company

at work or at school

Question C3

To what extent are the following statements concerning sport and physical activity true for your personally?

definitely true
true
partly true
not true
not true
at all

The area where I live offers many opportunities to be physically active.

Local sport clubs and other providers in my neighbourhood offer many opportunities to be physical active.

My municipality/city does enough for its citizens concerning their physical activities.

Country: Italy

Survey: Aspects of daily living

Survey Code: IT 03

Year: 2000

Type: Face to Face

Question 4.2

Is your domestic and extradomestic work mainly characterised by scarce, moderate or heavy physical activity? (one answer for each line)

Scarce
Moderate
Heavy

Family or housework
Working activity

Country: Romania

Survey: Health Interview Survey

Survey Code: RO 01

Year: 2000

Type: Face to Face

Question 109

Do you perform, regularly, at least once a week, any activity as: jogging, riding the bike, team sports, long enough to sweat?

Yes, How many days?
... days/week
No

Question 110

What is the main reason for performing such a physical activity?

Recreation
Keeping in shape
Therapeutically
Other reason: ...

Question 112

How would you describe the physical effort involved by your domestic activities?

Small
Average
Intense

Country: United Kingdom

Survey: The Health Survey for England

Survey Code: UK 12

Year: 2000

Type: Face to Face

Question 121

I'd like you to think about all the walking you have done in the past four weeks either locally or away from here. Please include any country walks and any other walks that you have done. In the past four weeks, that is since (date four weeks ago), have you done a continuous walk that lasted at least 5 minutes?

Yes
No
Can't walk at all

IF Yes
In the past 4 weeks, have you done a continuous walk that lasted at least 15 minutes? (That is since (date four weeks ago))

Yes
No

IF Yes
During the past 4 weeks, on how many days did you do a walk of at least 15 minutes? (That is since (date four weeks ago))

…

Question 122

How long did you usually spend walking each time you did a walk for 15 minutes or more?

… hours
… minutes

Question 123

In the past four weeks, that is since (date 4 weeks ago) have you done a short walk, of less than 5 minutes continuous walking?

Yes
No

Question 124

Which of the following best describes your usual walking pace

… a slow pace,
… a steady average pace,
… a fairly brisk pace,
… or, a fast pace - at least 4 mph?
none of these

Country: United States

Survey: National Health Interview Survey

Survey Code: US 01

Year: 2000

Type: Face to Face

Question AHB.090

How often do you do VIGOROUS activities for AT LEAST 10 MINUTES that cause HEAVY sweating or LARGE increases in breathing or heart rate?

NUMBER:
Never
1-995 times
Unable to do this type activity
Refused
Don't know

TIME PERIOD:
Day
Week
Month
Year
Unable to do this type activity
Refused
Don't know

Question AHB.100

About how long do you do these vigorous activities each time?

NUMBER:
1-995
Refused
Don’t know

TIME PERIOD:
Minutes
Hours
Refused
Don't know

Question AHB.108

Each time you do these vigorous activities, do you do them 20 minutes or more, or less than 20 minutes?

Less than 20 minutes
20 minutes or more
Refused
Don't know

Question AHB.110

How often do you do LIGHT OR MODERATE activities for AT LEAST 10 MINUTES that cause ONLY LIGHT sweating or a SLIGHT to MODERATE increase in breathing or heart rate?

NUMBER:
Never
1-995 times
Unable to do this type activity
Refused
Don't know

TIME PERIOD:
Day
Week
Month
Year
Unable to do this type activity
Refused
Don't know

Question AHB.120

About how long do you do these light or moderate activities each time?

NUMBER:
1-995
Refused
Don't know

TIME PERIOD:
Minutes
Hours
Refused
Don't know

Question AHB.128

Each time you do these light or moderate activities, do you do them 20 minutes or more, or less than 20 minutes?

Less than 20 minutes
20 Minutes or more
Refused
Don’t know

Country: Australia

Survey: National Health Survey

Survey Code: AU 01

Year: 2001

Type: Face to Face

Question AF210

I WILL NOW ASK YOU ABOUT MODERATE AND VIGOROUS EXERCISE APART FROM WALKING.

IN THE LAST 2 WEEKS, DID YOU DO ANY EXERCISE WHICH CAUSED A MODERATE INCREASE IN YOUR HEART RATE OR BREATHING, THAT IS, MODERATE EXERCISE?

Yes
No

Question AF211

HOW MANY TIMES DID YOU DO ANY MODERATE EXERCISE IN THE LAST 2 WEEKS?

Number: …

Question AF212

WHAT WAS THE TOTAL AMOUNT OF TIME YOU SPENT DOING MODERATE EXERCISE IN THE LAST 2 WEEKS?

Hours/minutes
100 hours or more

Question AF213

IN THE LAST 2 WEEKS, DID YOU DO ANY (OTHER) EXERCISE WHICH CAUSED A LARGE INCREASE IN YOUR HEART RATE OR BREATHING, THAT IS, VIGOROUS EXERCISE?

Yes
No

Question AF214

HOW MANY TIMES DID YOU DO ANY VIGOROUS EXERCISE IN THE LAST 2 WEEKS?

Number: …

Question 215

WHAT WAS THE TOTAL AMOUNT OF TIME YOU SPENT DOING VIGOROUS EXERCISE IN THE LAST 2 WEEKS?

Hours/minutes
100 hours or more

Country:	Belgium
Survey:	Health Interview Survey
Survey Code:	BE 02
Year:	2001
Type:	Face to Face

Question AP02

At least once a week do you engage in any regular activity such as jogging, cycling, … etc. long enough to work up sweat?

Yes: …. Days/week
No
Don't know

Question AP03

During the last 7 days, on how many days did you do vigorous physical activities like heavy lifting, digging, aerobics, or fast bicycling?

… days per week
Does not know
No answer

Question AP04

How much time in total did you usually spend on one of those days doing vigorous physical activities?

… hours … minutes
Does not know
No answer

Question AP05

During the last 7 days, on how many days did you do moderate physical activities?

… Days per week
Does not know
No answer

Question AP06

How much time in total did you do usually spend on one of those days doing moderate physical activities?
Indicate an average time per day

- Hours: … Minutes: ...
- Does not know
- No answer

Question AP07

During the last 7 days, on how many days did you walk for at least 10 minutes at a time?
This includes walking at work and at home, walking to travel from place to place, and any other walking that you might do solely for recreation, sport, exercise or leisure.

- … days per week
- Does not know
- No answer

Question AP08

How much time in total did you usually spend walking on one of those days?

- … hours … minutes
- Does not know
- No answer

Question AP09

At what pace did you usually walk?
Did you walk at:

- A Vigorous pace, that makes you breathe much harder than normal
- A Moderate pace that makes you breathe somewhat harder than normal
- A Slower pace where there is no change in your breathing
- Does not know
- No answer

Survey: Health Behaviour among the Finnish Adult Population

Survey Code: FI 06

Year: 2001

Type: Face to Face

Question 82

In the past year (12 months), have any of the persons mentioned below advised you to exercise more?

yes
no

physician
other health care provider
family member
other

Country: Finland

Survey: Health Behaviour among the Finnish Elderly Population

Survey Code: FI 09

Year: 2001

Type: Face to Face

Question 53

How often do you walk outdoors for at least half an hour?

daily
4 – 6 times a week
2 – 3 times a week
once a week
2 – 3 times a month
a few times a year or less
I cannot walk at all due to an illness or injury

Question 67

Which of the activities mentioned below do you pursue outside your home?

yes

visiting friends, acquaintances and relatives in my home district
travelling (as a tourist)
sports events (as a spectator or participant)
dancing
clubs and associations
parish activities
theatre, cinema, concerts
games (chess, cards, ball games, etc.)
fishing or hunting
cottage life, camping
studying
homemaking, child care
light work, voluntary work
nothing
other, please specify:

Question 74

Exercising is always worthwhile

Fully agree
More or less agree
Don't know
disagree to some extent
Fully disagree

Country:	Croatia
Survey:	Health Interview Survey
Survey Code:	HR 02
Year:	2001
Type:	Face to Face

Question 85

Think about all activities which take hard physical effort that you did in the last 7 days, regardless whether there were part of your regular job or for recreation. This includes activities as heavy lifting, digging, wood chipping, aerobics, playing football or basketball, fast cycling etc. (Only activities lasting for at least 10 minutes at a time should be included).

On how many days, during the last 7 days, did you do vigorous physical activities?

... days
Refusal
Don't know

Question 86

Think about all activities which take hard physical effort that you did in the last 7 days, regardless whether there were part of your regular job or for recreation. This includes activities as heavy lifting, digging, wood chipping, aerobics, playing football or basketball, fast cycling etc. (Only activities lasting for at least 10 minutes at a time should be included).

How much time in total, in a day, did you usually spend doing vigorous physical activities?

... hours ... minutes/day

Question 87

Now think about activities which take moderate physical effort that you did in the past 7 days.
This could be carrying light loads, ironing, house-cleaning, cycling at a regular pace, sewing etc   Do not include walking. Again, think about those physical activities lasting for at least 10 minutes at a time.

During the last 7 days, on how many days did you do moderate physical activities?

... days

Refusal
Don't know

Question 88

Now think about activities which take moderate physical effort that you did in the past 7 days.
This could be carrying light loads, ironing, house-cleaning, cycling at a regular pace, sewing etc   Do not include walking. Again, think about those physical activities lasting for at least 10 minutes at a time.

How much time in total, in a day, did you usually spend doing moderate physical activities?

... hours ... minutes

Question 89

Now think about the time you spent walking in the last 7 days. (Including walking at work and at home, walking because of travel from place to place, and any other walking for recreation, sport, exercise or leisure).

On how many days, during the last 7 days, did you walk for at least 10 minutes at a time?

... days
Refusal
Don't know

Question 90

Now think about the time you spent walking in the last 7 days. (Including walking at work and at home, walking because of travel from place to place, and any other walking for recreation, sport, exercise or leisure).

How much time in total, in a day, did you usually spend walking?

... hours ... minutes

Country:	Iceland
Survey:	Health and lifestyle of the Icelandic population
Survey Code:	IS 03
Year:	2001
Type:	Face to Face

Question 20

Relative to your age, are you in good or poor bodily condition?

Very good
Rather good
Neither good nor poor
Rather poor
Very poor

Country: Italy

Survey: Aspects of daily living

Survey Code: IT 04

Year: 2001

Type: Face to Face

Question 5.2

Is your domestic and extradomestic work mainly characterised by scarce, moderate or heavy physical activity? (one answer for each line)

Scarce
Moderate
Heavy

Family or housework
Working activity

Country: Macedonia

Survey: EUROHIS of Republic of Macedonia

Survey Code: MK 03

Year: 2001

Type: Face to Face

Question 85

Think about all the vigorous activities which take hard physical effort that you did in the past 7 days. Vigorous activities make you breathe much harder than normal and may include heavy lifting, digging, aerobics, or fast bicycling. Think about only those physical activities that you did for at least 10 minutes at a time.

During the past 7 days, on how many days did you do vigorous physical activities?
Think about only those physical activities that you do for at least 10 minutes at a time.

... days
Refusal
Don’t know

Question 86

How much time in total did you usually spend on one of those days doing vigorous physical activities?

... hours ... minutes/day

Interviewer probe: An average time per day is being sought. If the respondent can’t answer because the pattern of time spent varies widely from day to day, ask: "What is the total amount of time you spent over the past 7 days doing vigorous physical activities?"

... hours ... minutes

Question 87

Now think about activities which take moderate physical effort that you did in the past 7 days. Moderate physical activities make you breathe somewhat harder than normal and may include carrying light loads, bicycling at a regular pace, or doubles tennis. Do not include walking. Again, think about only those physical activities that you did for at least 10 minutes at a time.

During the past 7 days, on how many days did you do moderate physical activities?

... days
none
Refusal
Don't know

Question 88

How much time in total did you usually spend on one of those days doing moderate physical activities?

... hours ... minutes

Interviewer probe: An average time per day is being sought. If the respondent can't answer because the pattern of time spent varies widely from day to day, or includes time spent in multiple jobs, ask: "What is the total amount of time you spent over the last 7 days doing moderate physical activities?

... hours ... minutes

Question 89

Now think about the time you spent walking in the past 7 days. This includes walking at work and at home, walking to travel from place to place, and any other walking that you did solely for recreation, sport, exercise or leisure.

During the past 7 days, on how many days did you walk for at least 10 minutes at a time?

... days
none
Refusal
Don't know

Question 90

How much time in total did you usually spend walking on one of those days?

... hours ... minute

Interviewer probe: An average time per day is being sought. If the respondent can't answer because the pattern of time spent varies widely from day to day, ask: "What is the total amount of time you spent walking over the past 7 days?"

... hours ... minutes

Country: Netherlands

Survey: Patient survey - Second Dutch National Survey of General Practice

Survey Code: NL 02

Year: 2001

Type: Face to Face

Question LRV44s1

And how many days a week do you do more strenuous household chores such as scrubbing floors, carrying heavy shopping, etc?

Question LRV44s2

And how much time on average per day in hours and minutes do you spend on this?

Number of hours: ...

Question LRV44s3

Number of minutes: …

Country: Netherlands

Survey: Continuous Quality of Life Survey

Survey Code: NL 03

Year: 2001

Type: Self administered

Question 365

Imagine a normal week during the past months.
Could you indicate how many days per week you performed the activities mentioned below, how much time you spent on them on an average day and how strenuous these activities were?

walking to/from work or school
number of days per week
… Days
average time per day
… hours … min.

cycling to /from work or school
number of days per week
… Days
average time per day
… hours … min..

not applicable

effort

Slow
average
fast

Question 367

Domestic activities

light and moderately strenuous domestic work (standing work like cooking, dishwashing, ironing, feeding / bathing children and running work like hoovering, shopping)

heavy domestic work (scrubbing, beating out the rugs, walking with heavy shopping bags)

number of days per week

Days:…

average time per day

hours: …   min: …

Country: Slovenia

Survey: CINDI Health Monitor Questionnaire

Survey Code: SI 01

Year: 2001

Type: ???

Question 65

During the last 7 days, on how many days did you do vigorous physical activities?

Think only about physical activities that you did for at least 10 minutes at a time.
(Please mark 0 if you did not do any vigorous physical activity)

On ... days during the last 7 days

Question 66

Moderate physical activity: This refers to activity that requires moderate physical effort that makes you breathe somewhat harder than normal and may include carrying light loads, bicycling at a regular pace, or playing tennis doubles. Do not include walking.
During the last 7 days, on how many days did you do moderate physical activities?
Think only about physical activities that you did for at least 10 minutes at a time.
(Please mark 0 if you did not do any moderate physical activity.)

On ... days during the last 7 days

Question 67

During the last 7 days, on how many days did you walk for at least 10 minutes at a time? Include walking at work, at home, to commute from place to place, walking for recreation, and in connection with sport, exercise or leisure.
(Please mark 0 if you did not walk at least 10 minutes at a time)

On .. days during the last 7 days

Question 69

During the last year (12 months) have you been advised by any of the under mentioned to increase your physical activity?

yes
no

a doctor
other health care personnel
a family member
other

Country: Switzerland

Survey: Swiss Health Survey 2002

Survey Code: CH 02

Year: 2002

Type: Telephone

Question 44.02

And in your work, whether a professional activity,
household chores or gardening, how many
days a week do you perspire (on average)?

… days a week (between 0 and 7 days)
no reply

Question 44.10

The following question is not only concerned with physical activities which make you perspire, but also other forms of less intensive movement:
If you think of physical activities in the course of which you become more or less out of breath, such as walking quickly, hiking, dancing, gardening or various sports:
How many days a week do you do physical activity of this kind?

… days a week (between 1 and 7 days)
never
don't know
no reply

Question 44.11

What is the average duration of these physical activities on these days?

… hours … minutes a day
don't know
no reply

Question 44.40

Do you think you do enough physical activity for

your health?

yes
no
don't know
no reply

Country: Finland

Survey: FINRISK 2002

Survey Code: FI 07

Year: 2002

Type: Self administered

Question 80

How do you consider your current physical condition?

very good
quite good
fair
quite bad
very bad

Country: Finland

Survey: Health Behaviour among the Finnish Adult Population

Survey Code: FI 13

Year: 2002

Type: Face to Face

Question 84

In the past year (12 months), have any of the persons mentioned below advised you to exercise more?

yes
no

physician
other health care provider
family member
other

Question 85

How would you assess your own exercise habit?
Do you exercise for the maintenance of health in your own opinion

Far too little
A little too little
Properly
Too much

Country:	France
Survey:	National survey on health and national health insurance (ESPS)
Survey Code:	FR 09
Year:	2002
Type:	Face to Face

<u>Question 07</u>

Has your doctor asked you to take physical exercise?

Yes
no
doesn't know

Country: Ireland

Survey: Survey of Lifestyle, attitudes and nutrition (SLAN)

Survey Code: IE 03

Year: 2002

Type: Face to Face

Question B1

Considering a 7-day period (a week), how many times on average do you do the following kinds of exercise for more than 20 minutes during your free time?
(Please write the appropriate number on each line)

a) STRENUOUS EXERCISE (HEART BEATS RAPIDLY)
(e.g. running, jogging, hurling, camogie, football, soccer, squash, basketball, judo, roller skating, vigorous swimming, vigorous long distance cycling, advanced aerobics)

Times per Week: …

b) MODERATE EXERCISE (NOT EXHAUSTING)
(e.g. fast walking, tennis, badminton, easy swimming, easy cycling, popular and folk dancing, intermediate aerobics, heavy gardening)

Times per Week: …

c) MILD EXERCISE (MINIMAL EFFORT)
(e.g. yoga, golf, easy walking, fishing from river bank, bowling, beginners aerobics, archery, light gardening)

Times per Week: …

d) LITTLE / NO ACTIVITIES

Times per Week: …

Question B2

How many days, if any, in an average week do you walk for 30 minutes or more?

… days

Country:	International
Survey:	Eurobarometer October 2002
Survey Code:	INT 06
Year:	2002
Type:	Face to Face

Question 27

The next questions are about the time you spent being physically active in the last 7 days. They include questions about activities you do at work, to get from place to place, as part of your house and yard work, and in your spare time for recreation, exercise or sport.

In answering the following questions, Vigorous physical activities refer to activities that take hard physical effort and make you breathe much harder than normal. Moderate activities refer to activities that take moderate physical effort and make you breathe somewhat harder than normal.

In answering the questions, think about only those physical activities that you did for at least 10 minutes at a time.

During the last 7 days, on how many days did you do vigorous physical activities like heavy lifting, digging, aerobics or fast bicycling ?

… days per week
None (Go to question 32.)

Question 28

How much time in total did you usually spend on one of those days doing vigorous physical activities ?

… hours
… minutes

Question 29

During the last 7 days, on how many days did you do moderate physical activity like carrying light loads, bicycling at a regular pace or doubles tennis ?

Do not include walking.

… days per week
None

Question 30

How much time in total did you usually spend on one of those days doing moderate physical activities ?

... Hours
... Minutes

Question 31

During the last 7 days, on how many days did you walk for at least 10 minutes at a time ?

This includes walking at work and at home, walking to travel from place to place, and any other walking that you might do solely for recreation, sport, exercise or leisure.

... days per week
None

Question 32

How much time in total did you usually spent walking on one of those days ?

… hours
… minutes

Question 34

In the last 7 days how much physical activity did you get …?

Much
Moderate
Little or no

At work
For the purposes of transportation
In and around your home, including housework, gardening, general maintenance, or caring for your family
In recreation, sport and leisure-time activities

Country: International

Survey: World Health Surveys-WHS-Consolidated multi-country questionnaire

Survey Code: INT 08

Year: 2002

Type: Face to Face

Question 4400a

Now I am going to ask you about the time you spent being physically active in the last 7 days. Please answer each question even if you do not consider yourself to be an active person. Think about the activities you do at work, as part of your house and yard work, to get from places to place, and in your spare time for recreation, exercise or sport.

(Show Physical Activity card to respondent ---- see Appendix D/4)

Vigorous Activity
During the last 7 days, on how many days did you do vigorous physical activities like heavy lifting, digging, aerobics, or fast bicycling? Think about only those physical activities that you did for at least 10 minutes at a time.

Days per week: …

Question 4400b

How much time in total did you usually spend on one of those days doing vigorous physical activities?

Hours / minutes

Question 4401a

Moderate Activity
Again, think only about those physical activities that you did for at least 10 minutes at a time. During the last 7days, on how many days, did you do moderate physical activities like carrying light loads, bicycling at a regular pace, or double tennis? Do not include walking.

Days per week

Question 4401b

How much time in total did you usually spend on one of those days doing moderate physical activities?

Hours/minutes

Question 4402a

During the last 7 days, on how many days did you walk for at least 10 minutes at a time? This includes walking at work and at home, walking to travel from place to place, and any other walking that you did solely for recreation, sport, exercise or leisure.

Days per week: …

Question 4402b

How much time in total did you usually spend walking on one of those days?

Hours/Minutes

Country: Italy

Survey: Aspects of daily living

Survey Code: IT 05

Year: 2002

Type: Face to Face

Question 5.2

Is your domestic and extradomestic work mainly characterised by scarce, moderate or heavy physical activity? (one answer for each line)

Scarce
Moderate
Heavy

Family or housework
Working activity

Country: Lithuania

Survey: Lithuanian Health Behaviour Monitoring

Survey Code: LT 02

Year: 2002

Type: Self administered

Question 60

Vigorous physical activities: refer to activities that take hard physical effort and make you breathe much harder that normal.

During the last 7 days, on how many days did you do vigorous physical activities like heavy lifting, digging, aerobics, or fast bicycling? Include only those physical activities that you did for at least 10 minutes at a time. (please mark 0 if you did not do any vigorous physical activity)

On ... days

Question 61

How much time did you usually spend on one of those days doing vigorous physical activity?

... hours ... minutes

Question 62

Moderate physical activities: refer to activities that take moderate physical effort and make you breathe somewhat harder than normal.

During the last 7 days, on how many days did you do moderate physical activities like carrying light loads, bicycling at a regular pace, or doubles tennis?

Include only those physical activities that you did for at least 10 minutes at a time. Do not include walking. (please mark 0 if you did not do any moderate physical activity)

On ... days

Question 63

How much time in total did you usually spend on one of those days doing moderate physical activity?

... hours ... minutes

Question 67

During the last year (12 months) have you been advised to increase your physical activity by any of the following:

yes
no

a doctor
other health care personnel
a family member
others

Country: Latvia

Survey: Health Behaviour Survey of Latvian Adult Population

Survey Code: LV 03

Year: 2002

Type: Self administered

Question 81

Have you been advised to increase your physical activity by any of the following:

yes
no

a doctor
nurse
a dentist
other health care personnel
a family member
others

Question 82

Vigorous physical activities: refer to activities that take hard physical effort and make you breathe much harder that normal.

During the last 7 days, on how many days did you do vigorous physical activities like heavy lifting, digging, aerobics, or fast bicycling? Include only those physical activities that you did for at least 10 minutes at a time.

... days

Question 83

How much time did you usually spend on one of those days doing vigorous physical activity?

... hours ... minutes

Question 84

Moderate physical activities: refer to activities that take moderate physical effort and make you breathe somewhat harder than normal.

During the last 7 days, on how many days did you do moderate physical

activities like carrying light loads, bicycling at a regular pace, or doubles tennis? Include only those physical activities that you did for at least 10 minutes at a time. Do not include walking.

... days

Country: Malta

Survey: First National Health Interview Survey

Survey Code: MT 01

Year: 2002

Type: Face to Face

Question 53

PHYSICAL ACTIVITY

We are also interested in the physical activities that people do as part of their everyday lives.
I am going to ask you about the time you spend being physically active in the past 7 days. Please answer each question even if you do not consider yourself to be an active person. Think about the activities you do at work or school, at home or in your garden, to get from place to place and the spare time for recreation, exercise or sport.

Think about all the vigorous activities that take hard physical effort that you did in the past 7 days.
Vigorous activities make you breathe much harder than normal and may include heavy lifting, digging, football, vigorous swimming, aerobics or fast bicycling. Think about only those physical activities that you did for at least 10 minutes at a time.

During the past 7 days, on how many days did you do vigorous physical activities?

Question 54

How much time in total did you usually spend on one of those days doing vigorous physical activities?

... hours ... minutes

Question 55

Now think about activities which take moderate physical effort that you did in the past 7 days.
Moderate physical activities make you breathe somewhat harder than normal and may include carrying light loads, easy swimming, easy cycling or doubles tennis. Do not include walking. Again, think about only those physical activities that you did for at least 10 minutes at a time.

During the past 7 days, on how many days did you do moderate physical activities?

Question 56

How much time in total did you usually spend on one of those days doing moderate physical activities?

... hours ... minutes

Question 57

Now think about the time you spent walking in the past 7 days.
This includes walking at work, at school and at home, walking to travel from place to place and any other walking that you did solely for recreation, sport, exercise or leisure.

During the past 7 days, on how many days did you walk for at least 10 minutes at a time?

Question 58

How much time in total did you usually spend walking on one of those days?

Question 60

At least once a week do you engage in any regular activity such as jogging or cycling for long enough to work up sweat?

Yes
No

Question 61

How many days per week do you engage in this activity?

Question 62

What is this activity?

...

Question 63

HOUSEHOLD ACTIVITIES

Do you do light household work (e.g. dusting, washing dishes, changing light bulbs/plugs)?

Most days
3-4 times per week
Once per week
1-3 times per month
Seldom/never

Question 64

Do you do heavy household work (e.g. washing floors and windows, vacuuming/hoovering, plastering/whitewashing)?

Most days
2-3 times per week
Once per week
1-3 times per month
Seldom/never

Country:	Netherlands
Survey:	Continuous Quality of Life Survey
Survey Code:	NL 05
Year:	2002
Type:	Self administered

Question 367

Domestic activities

light and moderately strenuous domestic work (standing work like cooking, dishwashing, ironing, feeding / bathing children and running work like hoovering, shopping)

heavy domestic work (scrubbing, beating out the rugs, walking with heavy shopping bags)

number of days per week

Days:…

average time per day

hours: …   min: …

Country:	Denmark
Survey:	EUROHIS Recommended HIS Field Test Questionnaire - Long version
Survey Code:	REC 02
Year:	2002
Type:	Face to Face

<u>Question 114</u>

Interviewer: We are also interested in the physical activities that people do as part of their everyday lives. I am going to ask you about the time you spend being physically active in the past 7 days. Please answer each question even if you do not consider yourself to be an active person. Think about the activities you do at work, at home or in your garden, to get from place to place and the spare time for recreation, exercise or sport.

Think about all the vigorous activities which take hard physical effort that you did in the past 7 days. Vigorous activities make you breathe much harder than normal and may include heavy lifting, digging, aerobics, or fast bicycling. Think about only those physical activities that you did for at least 10 minutes at a time.

During the past 7 days, on how many days did you do vigorous physical activities?

… days
none
Refusal
Don't know

[Interviewer clarification: Think about only those physical activities that you do for at least 10 minutes at a time.]

[Interviewer: Include all activities and jobs.]

<u>Question 115</u>

How much time in total did you usually spend on one of those days doing vigorous physical activities?

... Hours ... minutes/day

[Interviewer clarification: Think about only those physical activities that you do for at least 10 minutes at a time.]

[Interviewer probe: An average time per day is being sought. If the respondent can't answer because the pattern of time spent varies widely from day to day, ask: "What is the total amount of time you spent over the past 7 days doing vigorous physical activities?"

... Hours … minutes

Question 116

Now think about activities which take moderate physical effort that you did in the past 7 days?

Moderate physical activities make you breathe somewhat harder than normal and may include carrying light loads, bicycling at a regular pace, or doubles tennis. Do not include walking. Again, think about only those physical activities that you did for at least 10 minutes at a time.

During the past 7 days, on how many days did you do moderate physical activities?

… days
none
Refusal
Don't know

[Interviewer clarification: Think about only those physical activities that you do for at least 10 minutes at a time.]

[Interviewer: Include all activities and jobs.]

Question 117

How much time in total did you usually spend on one of those days doing moderate physical activities?

... Hours … minutes

[Interviewer clarification: Think about only those physical activities that you do for at least 10 minutes at a time.]

[Interviewer probe: An average time per day is being sought. If the respondent can't answer because the pattern of time spent varies widely from day to day, or includes time spent in multiple jobs, ask:

What is the total amount of time you spent over the last 7 days doing moderate physical activities?

… hours … minutes

Question 118

Now think about the time you spent walking in the past 7 days. This includes walking at work and at home, walking to travel from place to place, and any other walking that you did solely for recreation, sport, exercise or leisure.

During the past 7 days, on how many days did you walk for at least 10 minutes at a time?

… days
none
Refusal
Don't know

[Interviewer clarification: Think about only the walking that you do for at least 10 minutes at a time.]

[Interviewer: Include all activities and jobs.]

Question 119

How much time in total did you usually spend walking on one of those days?

... Hours … minutes

Interviewer probe: An average time per day is being sought. If the respondent can't answer because the pattern of time spent varies widely from day to day, ask:

What is the total amount of time you spent walking over the past 7 days?

… hours … minutes

Country: Slovakia

Survey: Health Monitor Survey

Survey Code: SK 02

Year: 2002

Type: Face to Face

Question 6.4

Vigorous physical activities: refer to activities that take hard physical effort and make you breathe much harder that normal.

During the last 7 days, on how many days did you do vigorous physical activities like heavy lifting, digging, aerobics, or fast bicycling? Include only those physical activities that you did for at least 10 minutes at a time. (please mark 0 if you did not do any vigorous physical activity)

On ... days

Question 6.5

How much time did you usually spend on one of those days doing vigorous physical activity?

... hours ... minutes

Question 6.6

Moderate physical activities: refer to activities that take moderate physical effort and make you breathe somewhat harder than normal.

During the last 7 days, on how many days did you do moderate physical activities like carrying light loads, bicycling at a regular pace, or doubles tennis? Include only those physical activities that you did for at least 10 minutes at a time. Do not include walking. (please mark 0 if you did not do any moderate physical activity)

On ... days

Question 6.7

How much time in total did you usually spend on one of those days doing moderate physical activity?

... hours ... minutes

Question 6.8

During the last 7 days, on how many days did you walk for at least 10 minutes at the time?

Include walking at work and at home, walking to travel from place to place, and any other walking that you did solely for recreation, sport, exercise or leisure. (please mark 0 if you did not do any vigorous physical activity)

On ... days

Question 6.9

How much time in total did you usually spend walking on one of those days?

... hours ... minutes per day

Question 6.14

During the last year (12 months) have you been advised to increase your physical activity by any of the following?

yes
no

doctor
other health care personnel
family member
other

Country:	United Kingdom
Survey:	General Household Survey
Survey Code:	UK 07
Year:	2002
Type:	Face to Face

Question 15 Sprtnot

Is there any sport or recreational activity that you do
not do at the moment but would like to do?

Yes
No

Question 16 Sprtwch

Which one activity would you like to do?

…

Country: United Kingdom

Survey: Health Survey for England

Survey Code: UK 28

Year: 2002

Type: Face to Face

Question 147

On how many days in the last week did you/(name of child) do a continuous walk that lasted at least 5 minutes not counting things done as part of school lessons??

One day
Two days
Three days
Four days
Five days
Six days
Every day

Question 148

On each day that you/(name of child) did a walk like this for at least 5 minutes, how long did you/s/he spend walking altogether?

Please give an answer from this card

5 minutes, less than 15 minutes
15 minutes, less than 30 minutes
30 minutes, less than 1 hour
1 hour, less than 1 1/2 hours
1 1/2 hours, less than 2 hours
2 hours, less than 2 1/2 hours
2 1/2 hours, less than 3 hours
3 hours, less than 3 1/2 hours
3 1/2 hours, less than 4 hours
4 hours or more, please specify how long (How long did you/ (name of child) spend walking on each day?)

hours :… minutes: ….

Question 149

Which of the following describes your usual walking pace

... READ OUT ...

... a slow pace,
... a steady average pace,
... a fairly brisk pace,
... or, a fast pace - at least 4 mph?
(None of these)

Question 164

Were the activities you/(name of child) did last week different from what you/s/he would usually do for any reason?

No - same as usual
Yes different - usually do more
Yes different - usually do less

Question 165

I'd like to ask you about some of the things you have done in the past four weeks that involve physical activity, this could be at work/college or in your free time.

Can I just check, were you in paid employment or self-employed in the past four weeks?

Yes
No

Question 171a

I'd like you to think about all the walking you have done in the past four weeks either locally or away from here. Please include any country walks, walking to and from work and any other walks that you have done. In the past four weeks, that is since (date four weeks ago), have you done a continuous walk that lasted at least 5 minutes?

Yes,
No,
Can't walk at all

Question 171b

IF Yes, in the past four weeks, that is since (date four weeks a go), have you done a continuous walk that lasted at least 30 minutes?

Yes
No

Question 171c

IF Yes, during the past four weeks, that is since (date four weeks ago), on how many days did you do a walk of at least 30 minutes?

… days

Question 172

Which of the following best describes your usual walking pace

...READ OUT...

...a slow pace,
...an average pace,
...a fairly brisk pace,
...or, a fast pace - at least 4 mph?
None of these

Country: Canada

Survey: Canadian Community Health Survey Cycle 2.1

Survey Code: CA 02

Year: 2003

Type: Face to Face + Telephone

Question PAC_Q6

Thinking back over the past 3 months, which of the following best describes your usual daily activities or work habits?

Usually sit during the day and don't walk around very much

Stand or walk quite a lot during the day but don't have to carry or lift things very often

Usually lift or carry light loads, or have to climb stairs or hills often

Do heavy work or carry very heavy loads

Question_STR_Q6_6

How often do you jog or do other exercise to deal with stress?

Often
Sometimes
Rarely
Never

Question ETA_Q13

You think about burning up calories when you exercise.

Always
Usually
Often
Sometimes
Rarely
Never

Country:	Spain
Survey:	National Health Survey 2003
Survey Code:	ES 03
Year:	2003
Type:	Face to Face

Question 30

Could you indicate approximately how many hours a day you usually sleep?

Number of hours per day: ...

Country: Finland

Survey: Health Behaviour among the Finnish Adult Population

Survey Code: FI 10

Year: 2003

Type: Face to Face

Question 87

In the past year (12 months), have any of the persons mentioned below advised you to exercise more?

yes
no

physician
other health : care provider
family member
other

Country: Finland

Survey: Health Behaviour among the Finnish Elderly Population

Survey Code: FI 11

Year: 2003

Type: Face to Face

Question 54

How often do you perform other physical activities than walking for at least half an hour (e.g., skiing, bicycling, swimming, gymnastics, games, dance)?

daily
4 – 6 times a week
2 – 3 times a week
once a week
2 – 3 times a month
a few times a year or less
I cannot exercise at all due to an illness or injury

Question 69

Which of the activities mentioned below do you pursue outside your home?

yes

visiting friends, acquaintances and relatives in my home district
travelling (as a tourist)
sports events (as a spectator or participant)
dancing
clubs and associations
parish activities
theatre, cinema, concerts
games (chess, cards, ball games, etc.)
fishing or hunting
cottage life, camping
studying
homemaking, child care
light work, voluntary work
nothing
other, please specify:

Question 77

Exercising is always worthwhile

Fully agree
More or less agree
Don't know
disagree to some extent
Fully disagree

Country: Croatia

Survey: World Health Survey

Survey Code: HR 03

Year: 2003

Type: Face to Face

Question 2011

In the last 30 days, how much difficulty did you have in vigorous activities, such as running 3 km (or equivalent) or cycling?

None
Mild
Moderate
Severe
Extreme/Cannot do

Question 2104

[Mary] has no problems with walking, running or using her hands, arms and legs. She jogs 4 kilometres twice a week.

In the last 30 days, how much difficulty did [name of person] have in vigorous activities, such as running 3 km (or equivalent) or cycling?

None
Mild
Moderate
Severe
Extreme/Cannot do

Question 2106

[Anton] does not exercise. He cannot climb stairs or do other physical activities because he is obese. He is able to carry the groceries and do some light household work.

In the last 30 days, how much difficulty did [name of person] have in vigorous activities, such as running 3 km (or equivalent) or cycling?

None
Mild
Moderate
Severe
Extreme/Cannot do

Question 2108

[David] is paralyzed from the neck down. He is unable to move his arms and legs or to shift body position. He is confined to bed.

In the last 30 days, how much difficulty did [name of person] have in vigorous activities, such as running 3 km (or equivalent) or cycling?

None
Mild
Moderate
Severe
Extreme/Cannot do

Question 2112

[Rob] is able to walk distances of up to 200 metres without any problems but feels tired after walking one kilometre or climbing up more than one flight of stairs. He has no problems with day-to-day physical activities, such as carrying food from the market.

In the last 30 days, how much difficulty did [name of person] have in vigorous activities, such as running 3 km (or equivalent) or cycling?

None
Mild
Moderate
Severe
Extreme/Cannot do

Question 2114

[Vincent] has a lot of swelling in his legs due to his health condition. He has to make an effort to walk around his home as his legs feel heavy.

In the last 30 days, how much difficulty did [name of person] have in vigorous activities, such as running 3 km (or equivalent) or cycling?

None
Mild
Moderate
Severe
Extreme/Cannot do

Question 3006

Please try to imagine what it would be like to live in the following health state: having limited long-distance vision - able to read and recognize objects at arm's length but not to distinguish faces across a room (i.e., at distance of 5 meters); no glasses available.

How much difficulty would a person in this state have with vigorous activities, such as running 3 km (or equivalent) or cycling?

None
Mild
Moderate
Severe
Extreme/Cannot do

Questions 4030 - 4032

Now I am going to ask you about the time you spent being physically active in the last 7 days. Please answer each question even if you do not consider yourself to be an active person. Think about the activities you do at work, as part of your house and yard work, to get from places to place, and in your spare time for recreation, exercise or sport.

Vigorous activity

Now, think about all the vigorous activities which take hard physical effort that you did in the last 7 days. Vigorous activities make you breathe much harder than normal and may include heavy lifting, digging, aerobics, or fast bicycling. Think only about those physical activities that you did for at least 10 minutes at a time. During the last 7 days, on how many days did you do vigorous physical activities?

How much time did you usually spend doing vigorous physical activities on one of those days?

Hours per day : ...
Minutes per day: ...

Questions 4033 – 4035

Moderate Activity
Now think about activities which take moderate physical effort that you did in the last 7 days. Moderate physical activities make you breathe somewhat harder than normal and may include carrying light loads, bicycling at a regular pace, or doubles tennis. Do not include walking. Again, think about only those physical activities that you did for at least 10 minutes at a time. During the last 7 days, on how many days did you do moderate physical activities?

How much time did you usually spend doing moderate physical activities on one of those days?

Hours per day: ...
Minutes per day: ...

Questions 4036 – 4038

Walking
Now think about the time you spent walking in the last 7 days. This includes at work and at home, walking to travel from place to place, and any other walking

that you might do solely for recreation, sport, exercise, or leisure. During the last 7 days, on how many days did you walk for at least 10 minutes at a time?

How much time did you usually spend walking on one of those days?

Hours per day: ...
Minutes per day: ...

Country: Croatia

Survey: Croatian Health Survey

Survey Code: HR 04

Year: 2003

Type: Face to Face

Question PHA 04

During the last year (12 months) have you been advised to increase your physical activity by any of the following?

No
Yes

Doctor
Other health care personnel
Family member
Other

Country: Hungary

Survey: National Health Interview Survey

Survey Code: HU 05

Year: 2003

Type: Face to Face

Question 39

During the last 7 days, on how many days did you do vigorous physical activities? Consider activities done for at least 10 minutes.

... days
None of the days
Does not know/Not sure
Refused

Question 40

How much time did you usually spend doing vigorous physical activities on one of those days?

Hours per day?

… hrs

Minutes per day?

… mts

Does not know/Not sure
Refused

Question 41

During the last 7 days, on how many days did you do moderate physical activities?
Consider activities done for at least 10 minutes.

... days
None of the days
Does not know/Not sure
Refused

Question 42

How much time did you usually spend doing moderate physical activities on one of those days?

Hours per day?

… hrs

Minutes per day?

… mts

Does not know/Not sure
Refused

Question 43

During the last 7 days, on how many days did you walk for at least 10 minutes at a time? Consider the occasions when you walked for at least 10 minutes at a time.

... days
None of the days
Does not know
Refused

Question 44

How much time did you usually spend walking on one of those days?

Hours per day?

... hrs

Minutes per day?

... mts

Does not know
Refused

Country: Latvia

Survey: Health Interview Survey

Survey Code: LV 02

Year: 2003

Type: Face to Face

Question S 05

Over the last 3 weeks, what was your daily physical activity?

mostly sitting or lying
mostly standing or walking
daily physical effort
hard daily physical effort

Question S 06

Over the last 3 months, did you spend at least 30 minutes doing physical exercises?

yes, every day
4-6 times per week
2-3 times per week
once a week
2-3 times per month
once a month or more seldom
I cannot do it because of my health
no, I do not do it

Country: Netherlands

Survey: Continuous Quality of Life Survey

Survey Code: NL 08

Year: 2003

Type: Self administered

Question 367

Domestic activities

light and moderately strenuous domestic work (standing work like cooking, dishwashing, ironing, feeding / bathing children and running work like hoovering, shopping)

heavy domestic work (scrubbing, beating out the rugs, walking with heavy shopping bags)

number of days per week

Days:…

average time per day

hours: … min: …

Country: Sweden

Survey: Living Condition Survey

Survey Code: SE 03

Year: 2003

Type: Face to Face

Question 50

Can you run a short distance, say 100 meters, if you are in a hurry?

Yes Question 53 c if age 55+, question 55 if age below 55
No

Question 63 A

a) How tall are you?
Number of centimetres…..

b) How much do you weigh?
Number of kilograms…..

Country: Turkey

Survey: Turkey Demographic and Health Survey

Survey Code: TR 01

Year: 2003

Type: Face to Face

Question 772

Now I want to ask some questions about your daily life.

YES
NO

Do you make a branch of sports regularly?
Do you participate the activities of any society/club/association regularly?
Do you go to holiday other than your homeland/town?
Do you go outside for meal with your family?
Do you go to picnic?
Do you put on make up?
Do you wear head scarf when you go outside the street?

Country: Turkey

Survey: National Burden of Disease and Cost Effectiveness Study: National Health Survey

Survey Code: TR 05

Year: 2003

Type: Face to Face

Question 2011

In the last 30 days, how much difficulty did you have in vigorous activities, such as running 3km (or equivalent) or cycling?

None
Mild
Moderate
Severe
Extreme/Can not do›

Question 2103

Meryem has no problems with walking, running or using her hands, arms and legs. She jogs 4 kilometres twice a week.

In the last 30 days, how much difficulty did Meryem have in vigorous activities, such as running 3 km (or equivalent) or cycling?

None
Mild
Moderate
Severe
Extreme/Cannot do

Question 2105

Ali does not exercise. He cannot climb stairs or do other physical activities because he is obese. He is able to carry the groceries and do some light household work.

In the last 30 days, how much difficulty did Ali have in vigorous activities, such as running 3 km (or equivalent) or cycling?

None
Mild
Moderate
Severe
Extreme/Cannot do

Question 2107

Davut is paralyzed from the neck down. He is unable to move his arms and legs or to shift body position. He is confined to bed.

In the last 30 days, how much difficulty did Davut have in vigorous activities, such as running 3 km (or equivalent) or cycling?

None
Mild
Moderate
Severe
Extreme/Cannot do

Question 2112

Veli has a lot of swelling in his legs due to his health condition. He has to make an effort to walk around his home as his legs feel heavy.

Overall in the last 30 days, how much of a problem did Veli have with moving around?

None
Mild
Moderate
Severe
Extreme/Cannot do

Question 2113

Veli has a lot of swelling in his legs due to his health condition. He has to make an effort to walk around his home as his legs feel heavy.

In the last 30 days, how much difficulty did Veli have in vigorous activities, such as running 3 km (or equivalent) or cycling?

None
Mild
Moderate
Severe
Extreme/Cannot do

Question 3006

Having limited long-distance vision - able to read and recognize objects at arm's length but not to distinguish faces across a room (i.e., at distance of 5 meters); no glasses available.

How much difficulty would a person in this state have with vigorous activities, such as running 3 km (or equivalent) or cycling?

None
Mild
Moderate
Severe
Extreme/Cannot do

Questions 4030 – 4032

Now I am going to ask you about the time you spent being physically active in the last 7 days. Please answer each question even if you do not consider yourself to be an active person. Think about the activities you do at work, as part of your house and yard work, to get from places to place, and in your spare time for recreation, exercise or sport.

Vigorous Activity

Now, think about all the vigorous activities which take hard physical effort that you did in last 7 days. Vigorous activities make you breath much harder than normal and may include heavy lifting, digging, aerobics or fast Within the last one week, how many days have you done any vigorous activity which lasted at least 10 minutes once started bicycling? Think only about those physical activities that you did for at least 10 minutes at a time. During the last 7 days, on how many days did you do vigorous physical activities?

... days
No
If “No”, go to 4033

How much time did you usually spend doing vigorous physical activities on one of those days;

hours per day?

... hours

minutes per day?

... minutes

Questions 4033 – 4035

Moderate Activity

Now, think about activities which take moderate physical effort that you did in last 7 days. Moderate physical activities make you breath somewhat harder than normal and may include carrying light loads, bicycling at a regular pace, or doubles tennis. Do not include walking. Again, think about only those physical activities that you did for at least 10 minutes at a time. During the last 7 days, on how many days did you do moderate physical activities? (Show physical activity card to respondent)

... days
No
If "No" got to 4036

How much time did you usually spend doing moderate physical activities on one of those days;

hours per day?

... hours

minutes per day?

... minutes

Questions 4036 – 4038

Walking

Now, think about the time you spent walking in the last 7 days. This includes at work and at home, walking to travel from place to place , and any other walking that you might do solely for recreation, sport, exercise, or leisure. During the last 7 days, on how many days did you walk for at least 10 minutes at a time.

... days
No
If "No" go to 6000

How much time did you usually spend doing walking on one of those days;

hours per day?

... hours

minutes per day?

... minutes

Country: Australia

Survey: National Health Survey

Survey Code: AU 03

Year: 2004

Type: Face to Face

Question 19

Apart from insulin injections, medication or being on a special diet
In the last 2 weeks have you taken any other action to manage your [condition]?

Yes
No

Which other actions have you taken to manage your [condition]?
(Multiple response)

Losing weight
Exercised most days
Taken vitamins/mineral supplements
Taken natural/herbal medicines
Other

Country: Belgium

Survey: Health Interview Survey

Survey Code: BE 05

Year: 2004

Type: Self administered

Question PA02

At least once a week do you engage in any regular activity such as jogging, cycling, etc. long enough to work up sweat?

Yes
If yes, specify: ... days per week

No
I don't know

Question PA03

Vigorous physical efforts

During the last 7 days, on how many days did you do vigorous physical activities like heavy lifting, digging, aerobics, or fast bicycling?

... day(-s) per week

Question PA04

Vigorous physical efforts

How much time did you usually spend doing vigorous physical activities on one of those days?

... hours
... minutes/day
I don't know

Question PA05

Moderate physical efforts

During the last 7 days, on how many days did you do moderate physical activities like carrying light loads, bicycling at a regular place, or doubles tennis? (Do not include walking)

... days(-s) per week

Question PA06

Moderate physical efforts

How much time did you usually spend doing moderate physical activities on one of those days?

... hours
... minutes/day
I don't know

Question PA07

Walking

During the last 7 days, on how many days did you walk for at least 10 minutes at a time?

... day(-s) per week

Question PA08

Walking

How much time did you usually spend walking on one of those days?

... hours
... minutes/day
Don't know

Question PA09

Walking

At what pace did you usually walk?
Did you walk at:

a vigorous pace, that makes you breathe much harder than normal
a moderate pace that makes you breathe somewhat harder than normal
a slower pace where there is no change in your breathing .
Don't know

Country:	Estonia
Survey:	Health behaviour among Estonian adult population
Survey Code:	EE 07
Year:	2004
Type:	Self administered

Question 73

During the last 12 months, have you done any physical exercise that was completely or partly paid for by your own place of work or by that of your family member?

yes, regularly
yes, sometimes
no
I do not work

Question 76

How would you rate your current (physical) fitness status?

very good
fairly good
satisfactory
fairly poor
very poor

Question 77

Have you been advised to increase your physical activity by any of the following during the last 12 months?

yes
no

physician
other health care personnel
family member
other: ...

Country:	Finland
Survey:	Health Behaviour among the Finnish Adult Population
Survey Code:	FI 12
Year:	2004
Type:	Face to Face

Question 77

How would you rate your current (physical) fitness status?

very good
quite good
satisfactory
rather poor
very poor

Question 78

In the past year (12 months), have any of the persons mentioned below advised you to exercise more?

yes
no

physician
other health: ...
care provider
family member
other

Country: International (Finland, Baltic States)

Survey: FINBALT Health Monitor

Survey Code:

Year: 2004

Type: Self administered

Question 58

How would you rate your current (physical) fitness status? OR: How would you describe your present physical fitness status?

1 very good
2 fairly good
3 satisfactory
4 fairly poor
5 very poor

Question 59

During the last year (12 months) have you been advised to increase your physical activity by any of the following:

yes no

a doctor
other health care personnel
a family member
others

Question 25

Vigorous physical activities: refer to activities that take hard physical effort and make you breathe much harder that normal.
During the last 7 days, on how many days did you do vigorous physical activities like heavy lifting, digging, aerobics, or fast bicycling? Include only those physical activities that you did for at least 10 minutes at a time. (please mark 0 if you did not do any vigorous physical activity)

On … days

Question 26

How much time did you usually spend on one of those days doing vigorous physical activity?

… hours … minutes

Question 27

Moderate physical activities: refer to activities that take moderate physical effort and make you breathe somewhat harder than normal.
During the last 7 days, on how many days did you do moderate physical activities like carrying light loads, bicycling at a regular pace, or doubles tennis? Include only those physical activities that you did for at least 10 minutes at a time. Do not include walking. (please mark 0 if you did not do any moderate physical activity)

On … days

Question 28

How much time in total did you usually spend on one of those days doing moderate physical activity?

… hours … minutes

Question29

During the last 7 days, on how many days did you walk for at least 10 minutes at the time?
Include walking at work and at home, walking to travel from place to place, and any other walking that you did solely for recreation, sport, exercise or leisure. (please mark 0 if you did not do any vigorous physical activity)

On … days

Question 30

How much time in total did you usually spend walking on one of those days?

… hours … minutes

Country: Netherlands

Survey: Continuous Quality of Life Survey

Survey Code: NL 07

Year: 2004

Type: Self administered

Question 344

Domestic activities

light and moderately strenuous domestic work (standing work like cooking, dishwashing, ironing, feeding/bathing children and running work like hoovering, shopping)

heavy domestic work (scrubbing, beating out the rugs, walking with heavy shopping bags)

number of days per week

Days:…

average time per day

hours: … min: …

Country:	Sweden
Survey:	Survey of Living Conditions
Survey Code:	SE 04
Year:	2004
Type:	Face to Face

Question 50a

Can you run a short distance, say 100 meters, if you are in a hurry?

Yes Question 54
No

Question 60a

How tall are you?
Number of centimetres…..

Question 60b

How much do you weigh?
Number of kilograms…..

Country: Canada

Survey: National Population Health Survey (NPHS)

Survey Code: CA 03

Year: 2005

Type: Face to Face + Telephone

Question PA_Q6

Thinking back over the past 3 months, which of the following best describes your usual daily activities or work habits?

Usually sit during the day and don't walk around very much

Stand or walk quite a lot during the day but don't have to carry or lift things very Often

Usually lift or carry light loads, or have to climb stairs or hills often

Do heavy work or carry very heavy loads

Country: Germany

Survey: Telephone Health Survey 2004-2005

Survey Code: DE 07

Year: 2005

Type: Telephone

Question 234

In addition to sport, are you physically active in any way which helps to keep you healthy and fit?

Note: Meant here are physical activities which the interviewee would not count as sport, although it is regarded by some others as sport; for example, swimming, bicycling, hiking

yes
no
d.k.
n.a./answer refused

Question 235

Leaving sport aside, are you physically active in some other way which helps to keep you healthy and fit?

Note: Meant here are physical activities which the interviewee would not count as sport, although it is regarded by some others as sport; for example, swimming, bicycling, hiking

yes
no
d.k.
n.a./answer refused

Country: Denmark

Survey: The Danish Health Interview Survey

Survey Code: DK 03

Year: 2005

Type: Face to Face

Question 82

The next questions concern physical activity.

How many days a week are you physically active more than 30 minutes? Include moderate or vigorous physical activity where you increase your respiration, such as sports activities, strenuous gardening, walking rapidly, cycling at a moderate or rapid speed or physically strenuous work. Include work and leisure.
(The respondent should answer according to his or her current situation.)

0 days a week
1 day a week
2 days a week
3 days a week
4 days a week
5 days a week
6 days a week
7 days a week

Question 87

Do you know what the National Board of Health recommends for physical activity? (Until 1999, the National Board of Health recommended 4 hours of physical activity a week to positively affect health. The recommendation has since changed to 30 minutes of physical activity a day.
When the interviewer asks about the content of the recommendation, the interviewer must not indicate or mention the correct answer).

Yes
No

Question 87a

If yes:

What does the recommendation say?

That people should be physically active more than 30 minutes a day
That people should be physically active more than 4 hours a week
Other, write: ...
Do not know

Question 23 a-b

The next questions focus on how physically active you are. These questions differ from those you answered in the interview.

We are interested in finding out about the kinds of physical activities you do as part of your everyday life. The questions are about the time you spent being physically active in the last 7 days. They include questions about activities you do at work, as part of your house and yard work, to get from place to place, and in your spare time for recreation, exercise or sport.

State only the physical activities that you did for at least 10 minutes.

Vigorous physical activities refer to activities that take hard physical effort and make you breathe much harder than normal. Moderate activities refer to activities that take moderate physical effort and make you breathe somewhat harder than normal.

State how many days you were physical active

... days per week

State how much time you usually used per day

... hours
... minutes

How often during the last 7 days:

Did you do vigorous physical activities like heavy lifting, digging, aerobics, or fast bicycling?
Did you do moderate physical activities like carrying light loads, bicycling at a regular pace, or doubles tennis? Do not include walking
Did you walk? This includes walking at work and at home, walking to travel from place to place, and any other walking that you did solely for recreation, sport, exercise or leisure

Country: Finland

Survey: Health Behaviour among the Finnish Adult Population

Survey Code: FI 19

Year: 2005

Type: Self administered

Question 82

How would you rate your current (physical) fitness status?

very good
quite good
satisfactory
rather poor
very poor

Question 83

In the past year (12 months), have any of the persons mentioned below advised you to exercise more?

yes
no

physician
other health care provider
family member
other

Question 101

Are you a member in any society or organization?

no
yes

health and/or patient-organization
gymnastics and/or sports association
other organization

Country: Finland

Survey: Health Behaviour among the Finnish Elderly Population

Survey Code: FI 20

Year: 2005

Type: Face to Face

Question 53

How often do you walk outdoors for at least half an hour?

daily
4 – 6 times a week
2 – 3 times a week
once a week
2 – 3 times a month
a few times a year or less
I cannot walk at all due to an illness or injury

Question 54

How often do you perform other physical activities than walking for at least half an hour (e.g., skiing, bicycling, swimming, gymnastics, games, dance)?

daily
4 – 6 times a week
2 – 3 times a week
once a week
2 – 3 times a month
a few times a year or less
I cannot exercise at all due to an illness or injury

Question 55

How would you rate your current (physical) fitness status?

good
rather good
average
rather poor
poor

Question 70

Which of the activities mentioned below do you
pursue outside your home?

yes

visiting friends, acquaintances and relatives in my home district
travelling (as a tourist)
sports events (as a spectator or participant)
dancing
clubs and associations
parish activities
theatre, cinema, concerts
games (chess, cards, ball games, etc.)
fishing or hunting
cottage life, camping
studying
homemaking, child care
light work, voluntary work
nothing
other, please specify: ...

Question 78

Exercising is always worthwhile

Fully agree
More or less agree
Don't know
Disagree to some extent
Fully disagree

Country: France

Survey: Health Barometer

Survey Code: FR 14

Year: 2005

Type: Telephone

Question Q74

We are interested in the different types of physical activity you have engaged in during the past seven days, at work, at home or in your garden, for travelling and during your free time.

First consider intense activities that require a major physical effort and that make you breathe much more deeply than normally.
Think only of activities you engaged in for at least 10 minutes at a time.

During the past seven days, on how many days did you engage in intense physical activities such as carrying heavy loads, digging, cross-country cycling or playing football? Please do not include walking.

… days a week

Question Q75

In general, how long did you spend doing this intense physical activity during any one of these days (an average day)

… hours and … minutes a day

Question Q76

Let us now turn to moderate activities that require a moderate physical effort and that make you breath a little more deeply than normally.
Think only of activities you engaged in for at least 10 minutes at a time.

During the past seven day, on how many days did you engage in moderate physical activity such as carrying light bags, vacuum cleaning, cycling slowly? Please do not include walking.

… days a week

Question Q77

In general, how long did you spend doing this moderate physical activity during any one of these days (an average day)

… hours and … minutes a day

Question Q78

Let us now turn to walking.
This includes walking at work and at home, walking from one place to another, and any other kind of walking you may have done during your free time for leisure, sport, relaxation.

During the past seven days, on how many days did you walk for at least 10 minutes at a time?

… days a week

Question Q79

In general, how long did you walk during one of these days?

… hours and … minutes a day

Question 82

We are interested in different types of physical activity you engaged in yesterday at work, at home or in your garden, for getting from one place to another and during your free time. First consider intense activities that required a major physical effort and that made you breathe much more deeply than normally. Think only of activities you engaged in for at least 10 minutes at a time.

Yesterday, did you engage in intense physical activity such as carrying a heavy load, digging, cross-country cycling or playing football? Please do not include walking.

Yes
No
DK

Question Q83

If Yes,

How long did you spend on these intense physical activities yesterday?

… hours and … minutes

Question Q84

Let us now turn to moderate activities that require a moderate physical effort and that make you breath a little more deeply than normally.
Think only of activities you engaged in for at least 10 minutes at a time.

Yesterday, did you engage in moderate physical activity such as carrying light bags, vacuum cleaning, cycling slowly? Please do not include walking.

Yes
No
DK

Question Q85

How long did you spend on these moderate activities yesterday?

… hours and … minutes

Question Q88

Let us now turn to walking.
This includes walking at work and at home, walking from one place to another, and any other kind of walking you may have done during your free time for leisure, sport, relaxation.

Yesterday, did you walk for at least 10 minutes at a time?

Yes
No
DK

Question Q89

For how long did you walk?

… hours and … minutes

Country: Italy

Survey: Health Conditions and the Use of Health Services 2004-2005

Survey Code: IT 08

Year: 2005

Type: Face to Face

Question 17.2

How many hours and minutes per week, on average, do you devote to domestic and family-oriented work (housework, shopping, care of other family members)?

Do not carry out activities
N° hours: ...
N° minutes: ...

Question 17.3

Is your domestic and family-oriented work mainly characterised by physical activity?

Yes, light
Yes, moderate
Yes, heavy, till perspiring

Country:	Lithuania
Survey:	Health Interview Survey of Lithuanian population in 2005
Survey Code:	LT 06
Year:	2005
Type:	Face to Face

Question N01

We are interested in finding out about the kinds of physical activity that people do as a part of their everyday lives. I am going to ask you about the time spent being physically active in the last 7 days. Please answer each question even if you do not consider yourself to be an active person. I will be asking you about activities you do at work, to get from place to place, as a part of your house and yard work, in your spare time for recreation, exercise or sport.
Think about all vigorous activities that take hard physical effort that you did in the last 7 days. Vigorous activities make you breathe much harder than normal and may include heavy lifting, digging, fast bicycling or other hard physical work.
Think about those physical activities that you did for at least 10 minutes at a time.

During the last 7 days on how many days did you do vigorous physical activities?

... day(s)
None
Don‘t know/No answer

Question N02

How much time on the average per day do you spend doing vigorous physical activities? Think about those physical activities that you do at least 10 minutes at a time.

Interviewer: if the respondent cannot answer because the pattern of time spent varies widely from day to day, ask about the average time per week is being spent on vigorous physical activities?

... hours ... minutes per day
... hours ... minutes per week
Don’t know/No answer

Question N03

Think about activities that take moderate physical effort that you did in the last 7 days. These activities make your breath and heart beat somewhat harder than normal and may include carrying light loads, bicycling at a regular pace, doubles tennis. Think about those physical activities that you do at least 10 minutes at a time.

During the last 7 days on how many days did you do moderate physical activities?

... day(s)
None
Don‘t know/No answer

Question N04

How much time in the average per day do you spend doing moderate physical activities? Think about those physical activities that you do at least 10 minutes at a time.

Interviewer: if the respondent cannot answer because the pattern of time spent varies widely from day to day, ask about the average time per week is being spent on moderate physical activities.

... hours ... minutes per day
... hours ... minutes per week
Don’t know/No answer

Question N05

Now think about the time you spent walking in the last 7 days. This includes walking to go to work and return from it, walking to travel from one place to another, from which you usually go by some transport, also any other walking.

During the last 7 days on how many days did you walk for at least 10 minutes at a time?

... day(s)
None
Don‘t know/No answer

Question N06

How much time on the average per day did you spend walking? Think about walking that you do at least 10 minutes at a time.

Interviewer: if the respondent cannot answer because the pattern of time spent varies widely from day to day, ask about the average time per week is being spent walking.

... hours ... minutes per day
... hours ... minutes per week
Don’t know/No answer

Country: Poland

Survey: National Multicenter Health Survey - Project WOBASZ

Survey Code: PL 05

Year: 2005

Type: Face to Face

Question 5

Do you ever exercise (run, cycle, swim, train, garden) for at least 30 minutes without break?

yes
no

Question 6

How often do you exercise like that?

every day
almost every day (4-6 times a week)
every second or third day
once a week
twice or three times a month
once a month or rarely

Question 7

Why don’t you exercises? (Please choose only one answer)

lack of time
I don’t want to
no need to do it
because of my health status
I work very hard physically

Country: Portugal

Survey: Fourth National Health Interview Survey

Survey Code: PT 05

Year: 2005

Type: Face to Face

Question 0

ARE YOU] IS HE/SHE] ALWAYS BEDRIDDEN, SITTING ON A CHAIR ALL DAY OR CONFINED TO YOUR HOUSE?

Yes
No
Don't know

Question 1

THINK ABOUT EVERY VIGOROUS (ENERGIC) ACTIVITY, INCLUDING PROFESSIONAL ACTIVITY, YOU HAVE HE/SHE HAS DONE IN THE LAST 7 DAYS, WHICH REQUIRED HARD PHYSICAL EFFORT.

VIGOROUS ACTIVITIES ARE THOSE THAT LEAD TO BREATHING FASTER THAN NORMAL AND MAY INCLUDE LIFTING HEAVY WEIGHTS, DIGGING THE SOIL, PRACTICING AEROBIC GYMNASTICS, RUNNING, SWIMMING, PLAYING FOOTBALL OR CYCLING QUICKLY.

SO, I'D LIKE YOU TO THINK ABOUT JUST THOSE PHYSICAL ACTIVITIES, YOU HAVE HE/SHE HAS ☐ DONE AT LEAST DURING 10 MIN

HOW MANY DAYS IN THE LAST 7 DAYS, HAVE YOU HAS HE/SHE DONE VIGOROUS PHYSICAL ACTIVITIES?

No day
Don't know

Question 2

IN TOTAL, HOW MUCH TIME DID ☐YOU/HE/SHE☐USUALLY SPEND
THOSE DAYS, DOING VIGOROUS PHYSICAL ACTIVITIES?

... h ... m per day
Don't know because the pattern varies a lot day to day

Question 3

IN TOTAL, HOW MUCH TIME DID YOU/HE/SHE SPEND IN THE LAST 7 DAYS,DOING VIGOROUS PHYSICAL ACTIVITIES?

... h ... m in the week
Don't know

Question 4

NOW, THINK ABOUT THE ACTIVITIES, INCLUDING PROFESSIONAL ACTIVITY, YOU HAVE HE/SHE HAS DONE IN THE LAST 7 DAYS, WHICH REQUIRED MODERATE PHYSICAL EFFORT MODERATE PHYSICAL ACTIVITIES LEAD TO BREATHING A LITTLE FASTER THAN NORMAL AND MAY INCLUDE CARRYING SOFT WEIGHTS, CYCLING SLOWLY, HOUSE CLEANING, HOOVERING, GARDENING, HUNTING. WALKING IS NOT INCLUDED.

ONCE AGAIN, THINK ABOUT JUST THOSE PHYSICAL ACTIVITIES YOU HAVE HE/SHE HAS ☐ DONE AT LEAST DURING 10 MINUTES AT O

HOW MANY DAYS IN THE LAST 7 DAYS, HAVE YOU HAS HE/SHE DONE MODERATE PHYSICAL ACTIVITIES?

No day
Don't know

Question 5

IN TOTAL, HOW MUCH TIME DID YOU/HE/SHE USUALLY SPEND IN 1 OF THOSE DAYS, DOING MODERATE PHYSICAL ACTIVITIES?

... h ... m per day
Don't know because the pattern varies a lot day to day

Question 6

IN TOTAL, HOW MUCH TIME DID YOU/HE/SHE SPEND IN THE LAST 7 DAYS, DOING MODERATE PHYSICAL ACTIVITIES?

... h ... m in the week
Don't know

Question 7

THINK ABOUT THE TIME SPENT WALKING IN THE LAST 7 DAYS.
THIS INCLUDES WALKING AT WORK AND AT HOME, MOVING FROM ONE PLACE TO ANOTHER, GOING FOR A WALK OR WALKING FOR EXERCISE.

HOW MANY DAYS IN THE LAST 7 DAYS, HAVE YOU HAS HE/SHE WALKED AT LEAST DURING 10 MINUTES AT ONCE?

No day
Don’t know

Question 8

IN TOTAL, HOW MUCH TIME DID YOU/HE/SHE USUALLY SPEND WALKING IN 1 OF THOSE DAYS?

... h ... m per day
Don’t know because the pattern varies a lot day to day

Question 9

IN TOTAL, HOW MUCH TIME DID YOU/HE/SHE SPEND WALKING IN THE LAST 7 DAYS?

... h ... m in the week
Don’t know

Country: Sweden

Survey: National Survey of Public Health

Survey Code: SE 05

Year: 2005

Type: ???

<u>Question 37</u>

How much time in a normal week do you do moderately strenuous activities that make you hot? E.g. walks at a brisk pace, gardening, fairly hard housework, cycling, swimming. This may vary during the year, but try and give some kind of an average. Mark one alternative.

5 hours a week or more
More than 3 hours but less than 5 hours a week
Between 1 and 3 hours a week
At most one hour a week
Not at all

Country: United Kingdom

Survey: Health Survey for England

Survey Code: UK 31

Year: 2005

Type: Face to Face

Question Wlk5Ch

Now I'd like to ask you about some of the things you have/(name of child) has done in the last week. By last week I mean last (day one week ago) up to yesterday. In the last week, have you/has s/he done a continuous walk that lasted at least 5 minutes not counting things done as part of school lessons?

Yes
No

Question DaysWlk

On how many days in the last week did you/(name of child) do a continuous walk that lasted at least 5 minutes not counting things done as part of school lessons?

One day
Two days
Three days
Four days
Five days
Six days
Every day

Question DayWlkT

On each day that you/(name of child) did a walk like this for at least 5 minutes, how long did you/s/he spend walking altogether?
Please give an answer from this card

5 minutes, less than 15 minutes
15 minutes, less than 30 minutes
30 minutes, less than 1 hour
1 hour, less than 1 1/2 hours
1 1/2 hours, less than 2 hours
2 hours, less than 2 1/2 hours
2 1/2 hours, less than 3 hours
3 hours, less than 3 1/2 hours
3 1/2 hours, less than 4 hours
4 hours or more (please specify how long)

Question WlkHrs

How long did you/(name of child) spend walking on each day?

...

Question ChPace

Which of the following describes your usual walking pace

... a slow pace,
... a steady average pace,
... a fairly brisk pace,
... or, a fast pace - at least 4 mph?
(None of these)

Question Usual

Were the activities you/(name of child) did last week different from what you/s/he would usually do for any reason?

IF YES PROBE: Would you/s/he usually do more physical activity or less?

No - same as usual
Yes different - usually do more
Yes different - usually do less

Country:	Austria
Survey:	Health Interview Survey
Survey Code:	AT 06
Year:	2006
Type:	Face to Face

Question D5.1

We are interested in finding out about the kinds of physical activity that people do as part of their everyday lives. I am going to ask you about the time you spent being physically active in the past seven days. Please answer each question, even if you do not consider yourself to be an active person. I will be asking you about activities you do at work, to get from place to place, as part of your house and yard work, and in your spare time for recreation, exercise or sport.
Think about all the vigorous activities that take hard physical effort that you did in the last seven days. Vigorous activities make you breathe much harder than normal and may include heavy lifting, digging, aerobics or fast bicycling. Think about only those physical activities that you did for at least 10 minutes at a time.

During the last seven days, on how many days did you do vigorous physical activities?

… day(s) per week

Question D5.2

How much time in total did you usually spend on one of these days doing vigorous physical activities?

… hours … minutes/day
don't know

Question D5.2a

How much time in total would you spend in a usual week doing vigorous physical activities?

… hours … minutes/week

Question D5.3

Now think about all the vigorous activities that take moderate physical effort that you did in the last seven days. Moderate physical activities make you breathe somewhat harder than normal and may include light loads, bicycling at a regular pace, or doubles tennis. Do not include walking. Again, think about only those physical activities that you did for at least 10 minutes at a time.

During the last seven days, on how many days did you do moderate physical activities?

… day(s) per week

Question D5.4

How much time in total did you usually spend on one of those days doing moderate physical activities?

… hours … minutes/day
don't know

Question D5.4a

How much time in total would you spend in a usual week doing moderate physical activities?

… hours … minutes/week

Question D5.5

Now think about the time you spent walking in the last seven days. This includes walking at work and at home, walking to travel from place to place, and any other walking that you might do solely for recreation, sport, exercise or leisure.

During the last seven days, on how many days did walk for at least 10 minutes at a time?

… day(s) per week

Question D5.6

How much time in total did you usually spend walking on one of those days?

… hours … minutes/day
don't know

Question D5.6a

How much time in total would you spend in a usual week walking?

… hours … minutes/week

Question D5.7

What describes best your leisure time activities during the last year?

Hard training and competitive sport more than once a week
Jogging and other recreational sports or heavy gardening, at least 4 hours a week
Jogging and other recreational sports or heavy gardening, less than 4 hours a week
Walking, bicycling or other light activities at least 4 hours a week
Walking, bicycling or other light activities less 4 hours a week
Reading, watching TV or other sedentary activities

Question D5.8

At least once a week do you engage in any regular activity, such as jogging, cycling, etc. long enough to work up sweat?

Yes
No

Question D5.9

How many days per week?

On … Day(s) per week

Country:	Serbia
Survey:	National Health Survey, Serbia 2006
Survey Code:	CS 01
Year:	2006
Type:	Face to Face + self administered

Question SV45 a-b

Think about all the vigorous activities which demand heavy physical effort that you did in the last 7 days. Vigorous activities make you breathe much harder than normal and may include heavy lifting, digging, aerobics or fast bicycling. Think only about those physical activities that you did for at least 10 minutes at the time.

During the last 7 days, on how many days did you do vigorous physical activities last week?

... days
Do not want to answer
Do not know

How much time did you usually spend doing vigorous physical activities on one of those days?

... hours ... minutes

Question SV6 a-b

Think about activities which take moderate physical effort that you did in the last 7 days. Moderate activities make you breathe slightly somewhat harder than normal and include carrying light loads bicycling at a regular pace, or doubles tennis. Do not include walking. Again, think only those physical activities that you did for at least 10 minutes at the time.

During the last 7 days, on how many days did you do moderate physical activities last week?

... days
Do not want to answer
Do not know

How much time did you usually spend doing moderate physical activities on one of those days?

... hours ... minutes

Question ZZ4

How do you estimate your physical activity? (One answer)

Very bad
Bad
Fair
Good
Very good

Question SV6

How many hours a week do you practice physical activity until you start sweating and start breathing faster (excluding school activities)?

Up to 1 hour
2-3 hours
4-6 hours
7 or more than 7 hours

Question SV7 a-b

Now think about time you spent walking in the last 7 days. This includes at work/school and at home, walking to travel to place to place, and any other walking that you might do solely for recreation, sports, exercise or leisure.

During the last 7 days, on how many days did you walk for at least 10 minutes at a time?

... days
Do not want to answer
Do not know

How much time did you usually spend walking on one of those days?

... hours ... minutes

Question SV9

Think about all the vigorous activities which demand heavy physical effort that you did in the last 7 days. Vigorous activities make you breathe much harder than normal and may include heavy lifting, digging, aerobics or fast bicycling. Think only about those physical activities that you did for at least 10 minutes at the time.

During the last 7 days, on how many days did you do vigorous physical activities last week?

... days
Do not want to answer
Do not know

How much time did you usually spend doing vigorous physical activities on one of those days?

... hours ... minutes

Question SV10

Think about activities which take moderate physical effort that you did in the last 7 days. Moderate activities make you breathe slightly somewhat harder than normal and include carrying light loads bicycling at a regular pace, or doubles tennis. Do not include walking. Again, think only those physical activities that you did for at least 10 minutes at the time.

During the last 7 days, on how many days did you do moderate physical activities last week?

... days
Do not want to answer
Do not know

How much time did you usually spend doing moderate physical activities on one of those days?

... hours ... minutes

Question SV11

Now think about time you spent walking in the last 7 days. This includes at work/school and at home, walking to travel to place to place, and any other walking that you might do solely for recreation, sports, exercise or leisure.

During the last 7 days, on how many days did you walk for at least 10 minutes at a time?

... days
Do not want to answer
Do not know

How much time did you usually spend walking on one of those days?

... hours ... minutes

Country: Germany

Survey: Telephone Health Survey 2005-2006

Survey Code: DE 08

Year: 2006

Type: Telephone

Question 141

In addition to sport, are you physically active in any way which helps to keep you healthy and fit?

Meant here are physical activities which the interviewee would not count as sport, although it is regarded by some others as sport; for example, swimming, bicycling, hiking

yes
no
d.k.
n.a./answer refused

Question 142

Leaving sport aside, are you physically active in some other way which helps to keep you healthy and fit?

Meant here are physical activities which the interviewee would not count as sport, although it is regarded by some others as sport; for example, swimming, bicycling, hiking

yes
no
d.k.
n.a./answer refused

Country: Estonia

Survey: Estonian Health Interview Survey 2006

Survey Code: EE 08

Year: 2006

Type: Face to Face

Question T11

Physical activity is any activity that causes your heart to palpitate and makes you gasp for a period. Physical activity can be an intensive sports training that is aimed for high results or recreational sports for enjoying the activity. The activities that are connected with your daily work should also be included in physical activity. These activities should last at least for 10 minutes during a half an hour period and for at least 30 minutes per day. In addition to sports, physical activity involves also several leisure time activities connected with physical movements, such as Nordic walking, longer walks and cycling (also to and from work) and other physical activities of the same level of difficulty.

Have you had moderate-intensity physical exercises such as Nordic walking, longer walks (also to and from work), cycling (also to and from work) or been involved in any other easier physical activity during the last 4 weeks?

Yes
No

Question T12

How many hours per week have you been involved in moderate-intensity physical activity during the last 4 weeks?

... hours

Question T13

Now we are going to talk about physical activity and exercises that can be called active exercises. These include jogging, ball games, swimming, skating, dancing and also harder garden work.

Have you ever in your life been involved in active exercises? These include jogging, other recreational sports and harder garden work.

Yes
No

Question T14

Have you participated in active exercises during the last 4 weeks?

Yes
No

Question T15

How often did you participate in active exercises during the last 4 weeks?

Almost every day
3 – 4 times a week
1 – 2 times a week
1 – 3 times a month

Question T16

How many hours per week did you participate in active exercises during the last 4 weeks?

... hours

Question T17

The following questions are connected with intensive sports activities.

Have you ever in your life been involved in intensive training or competitive sports, i.e. at least 3-4 training sessions per week, during at least one year?

Yes
No

Question T18

Have you participated in intensive training or competitive sports, i.e. at least 3-4 training sessions per week, during the last 4 weeks?

Yes
No

Question T19

How often did you participate in intensive training or competitive sports, i.e. 3-4 training sessions a week, during the last 4 weeks?

Every day/almost every day
3 – 4 times a week

Question T20

Are you physically inactive for more than two hours in a row (for example reading, sitting at the computer, watching TV)?

Yes
No

Question T21

How many hours a week are you usually physically inactive (for example reading, sitting at the computer, watching TV)?

... hours

Country: Spain

Survey: National Health Survey

Survey Code: ES 06

Year: 2006

Type: Face to Face

Question 90

Which of the following possibilities best describes your main activity at your place of work, education centre, home (household chores), etc?

Seated most of the day
Standing up most of the day, without moving around a lot or making a great deal of effort
Walking, carrying a bit of weight, moving around frequently
Doing tasks requiring a great deal of physical effort

Question 93

Do you regularly do any physical activity like walking, doing sport or going to the gym in your free time?

Yes
No, I don’t normally do physical exercise

Question 94

Could you tell me how often you have done the activities set out below for over 20 minutes in the last two weeks?

N° of times: ...

A light physical activity like walking, gardening, easy gym activities, games that require little effort or similar activities
A moderate physical activity like riding a bicycle, gymnastics, aerobics, running or swimming
An intense physical activity like football, basketball, competitive cycling or swimming, judo karate or similar activities

Country: France

Survey: National survey on nutrition and health (ENNS)

Survey Code: FR 18

Year: 2006

Type: Face to Face

Question PA48

The questions I am going to ask you first of all concern the time you spent being physically active during the past week. This includes the seven previous days up until yesterday included (including the weekend).
This includes activities at work or school, at home or in your garden, to get from one place to another, or during leisure or sports activities.
Answer all the questions even if you do not consider yourself to be a physically active person.

The time devoted to intense physical activities
The first question concerns intense physical activity. This means activities that required you to make a major physical effort and that made you breathe with much more difficulty than normally.

Think of moments when, for example, you carried heavy loads, dug your garden, went cross-country cycling, played football or did step aerobics.

During the past week, including the weekend, on how many days do you undertake this kind of intense physical activity?

Only include activities you engaged in for at least 10 minutes without interruption.

… days/7 days
Refuses to answer
Doesn't know

Question PA49

Now think of one of these days in the past week when you engaged in one or more intense physical activities. How much time in total did you spend on them?

on one of these days … hours … minutes
Refuses to answer
Doesn't know

Question PA50

The time spent on moderate physical activity
The second question concerns moderate physical activity. This means activities that required you to make a moderate physical effort and that made you breathe with a little more difficulty than usual.
Think of times when you carried moderate loads (5-10 kg), did the vacuum cleaning, went for a quiet bicycle ride, or played some volley ball.
Again, think only of activities you engaged for at least 10 minutes without interruption.

During the past week, including the weekend, on how many days did you engage in this type of moderate physical activity? Do not include walking

… days/7 days
Refuses to answer
Doesn't know

Question PA51

Now think of one of these days in the past week when you engaged in moderate physical activity. How much time in total did you spend engaged in this activity?

On one of these days … hours … minutes
Refuses to answer
Doesn't know

Question PA52

During the past week, including the weekend, on how many days did you walk for at least 10 minutes without interruption?

… days/7 days
Refuses to answer
Doesn't know

Question PA53

Now think of one of these days in the past week when you walked.
For how long did you walk?

on one of these days … hours … minutes
Refuses to answer
Doesn't now

Question E17

Do you believe you are physically active?

Yes
No
Refuses to answer

Question E53

The following questions concern the physical activity and sedentariness of your child during the past week, that is, on the seven days including yesterday.

It is possible that you do not know exactly how to answer the questions; try to be as close as possible to what you have seen.
II. During the past week, how much time did your child spend watching television in all (video cassette and DVD included)?

For children attending neither school or crèche, or interviewed during holidays

Total time per day: … Hours … minutes
Refusal
Doesn't know

Question E54

The following questions concern the physical activity and sedentariness of your child during the past week, that is, on the seven days including yesterday.

It is possible that you do not know exactly how to answer the questions; try to be as close as possible to what you have seen.
II. During the past week, how much time did your child spend watching television in all (video cassette and DVD included)?

For children attending school (or a crèche)

School days (Monday, Tuesday, Thursday, Friday) … Hours … minutes
Days with little school (Wednesday or Saturday) … Hours … minutes
Days with no school (Wednesday or Saturday, Sunday) … Hours … minutes
Refuses to answer
Doesn't know

Question E61

In your opinion, is your child active?

Yes
No
Doesn't know
Refuses to answer

Question A8

Do you regularly engage in a physical activity to reduce your blood pressure?

(fast walking, sport, gardening, etc.)

Yes
No

Question A13

Do you regularly engage in a physical activity to reduce your cholesterol?
(fast walking, sport, gardening, etc.)

Yes
No

Question A21

Do you regularly engage in a physical activity to reduce your glycaemia?
(fast walking, sport, gardening, etc.)

Yes
No

Question PA.67

How did you try to loose weight ? (more than one answer possible)

By modifying your feeding behaviour
By increasing your physical activity
Other :meal substitutes, diet complements, medicinal herbs, laxatives, etc (Explain): ...

Question PA.69

How did you try not to gain weight? (more than one answer possible)

By modifying your feeding behaviour
By increasing your physical activity
Other: meal substitutes, diet complements, medicinal herbs, laxatives, etc (Explain) ...

Country: Lithuania

Survey: Lithuanian health behaviour monitoring

Survey Code: LT 05

Year: 2006

Type: Self administered

Question 77

During the last year (12 months) have you been advised to increase your physical activity by any of the following:

yes
no

a doctor
other health care personnel
a family member
others

Country: International

Survey: European Core Health Interview Survey - ECHIS

Survey Code: REC 04

Year: 2006

Type: Face to Face

Question PE.1

Now I am going to ask you about the time you spent being physically active in the past 7 days. Please answer each question even if you do not consider yourself to be an active person. Think about the activities you do at work, as part of your house and yard work, to get from place to place, and in your spare time for recreation, exercise or sport.

Think about all the vigorous activities which take hard physical effort that you did in the last 7 days. Vigorous activities make you breathe much harder than normal and may include heavy lifting, digging, aerobics, or fast bicycling. Think only about those physical activities that you did for at least 10 minutes at a time.

During the past 7 days, on how many days did you do vigorous physical activities?

... Days per week
don't know
refusal

Question PE.2

During the past 7 days, how much time did you spend doing vigorous physical activities?

... hours ... minutes
don't know
refusal

Question PE.3

Now think about activities which take moderate physical effort that you did in the past 7 days. Moderate physical activities make you breathe somewhat harder than normal and may include carrying light loads, bicycling at a regular pace, or doubles tennis. Do not include walking. Again, think about only those physical activities that you did for at least 10 minutes at a time.

During the past 7 days, on how many days did you do moderate physical activities?

... Days per week
don't know
refusal

Question PE.4

During the past 7 days, how much time did you spend doing moderate physical activities?

... hours ... minutes
don't know
refusal

Question PE.5

Now think about the time you spent walking in the past 7 days. This includes at work and at home, walking to travel from place to place, and any other walking that you might do solely for recreation, sport, exercise, or leisure.

During the past 7 days, on how many days did you walk for at least 10 minutes at a time?

... Days per week
don't know
refusal

Question PE.6

INTERVIEWER CLARIFICATION: THINK ONLY ABOUT THE WALKING THAT YOU DO FOR AT LEAST 10 MINUTES AT A TIME.

During the past 7 days, how much time did you spend walking?

... hours ... minutes
don't know
refusal

Country:	United Kingdom
Survey:	Health Survey for England
Survey Code:	UK 33
Year:	2006
Type:	Face to Face

Question Wlk5Ch

Now I'd like to ask you about some of the things you have/(name of child) has done in the last week. By last week I mean last (day one week ago) up to yesterday. In the last week, have you/has s/he done a continuous walk that lasted at least 5 minutes not counting things done as part of school lessons?

Yes
No

Question DaysWlk

On how many days in the last week did you/(name of child) do a continuous walk that lasted at least 5 minutes not counting things done as part of school lessons?

One day
Two days
Three days
Four days
Five days
Six days
Every day

Question DayWlkT

On each day that you/(name of child) did a walk like this for at least 5 minutes, how long did you/s/he spend walking altogether?

What we want recorded is the average time spent walking per weekday.
If the respondent walked for over 5 minutes on more than one day in the last week, take an average of the time spent per day

5 minutes, less than 15 minutes
15 minutes, less than 30 minutes
30 minutes, less than 1 hour
1 hour, less than 1 1/2 hours
1 1/2 hours, less than 2 hours
2 hours, less than 2 1/2 hours
2 1/2 hours, less than 3 hours
3 hours, less than 3 1/2 hours
3 1/2 hours, less than 4 hours
4 hours or more (please specify how long)

Question WlkHrs

How long did you/(name of child) spend walking on each day?
RECORD HOURS SPENT BELOW

...

RECORD HERE MINUTES SPENT WALKING.

...

Question ChPace

Which of the following describes your usual walking pace ...

... a slow pace,
... a steady average pace,
... a fairly brisk pace,
... or, a fast pace - at least 4 mph?
... None of these

Question Wlk5Int

I'd like you to think about all the walking you have done in the past four weeks either locally or away from here. Please include any country walks, walking to and from work and any other walks that you have done. In the past four weeks, that is since (date four weeks ago), have you done a continuous walk that lasted at least 5 minutes?

Yes
No
Can't walk at all

Question Wlk30M

In the past four weeks, have you done a continuous walk that lasted at least 30 minutes? (That is since (date four weeks a go)

Yes
No

During the past four weeks, on how many days did you do a walk of at least 30 minutes? (That is since (date four weeks ago)

...

Question Wlk15M

In the past four weeks, have you done a continuous walk that lasted at least 15 minutes? (That is since (date four weeks a go)

Yes
No

Question DayWlk

During the past four weeks, on how many days did you do a walk of at least 15 minutes? (That is since (date four weeks a go)

...

On that day, did you do more than one walk lasting at least 15 minutes?

Yes, more than one walk of 15 + mins (on at least one day)
No, only one walk of 15 + mins a day

On how many days in the last four weeks did you do more than one walk that lasted at least 15 minutes?

...

Question HrsWlk

How long did you usually spend walking each time you did a walk for 15 minutes or more? If very different lengths, probe for most regular. Record hours spent below.

...

Record here minutes spent walking.

...

Question WalkPace

Which of the following best describes your usual walking pace ...

... a slow pace,
... an average pace,
... a fairly brisk pace,
... or, a fast pace - at least 4 mph?
None of these

Country: United States

Survey: National Health Interview Survey

Survey Code: US 04

Year: 2006

Type: Face to Face + Telephone

Question ACN.290_02

Has a doctor or other health professional EVER suggested physical activity or exercise to help your arthritis or joint symptoms?

Yes
No
Refused
Don't know

Question ACN.535_02

DURING THE PAST 12 MONTHS, have you been told by a doctor or health professional to increase your physical activity or exercise?

Yes
No
Refused
Don't know

Question ACN.540_02

DURING THE PAST 12 MONTHS, have you

... increased your physical activity or exercise?

Yes
No
Refused
Don't know

Country: Australia

Survey: National Health Survey

Survey Code: AU 04

Year: 2007

Type: Face to Face

Question EXER_Q07

The next few questions are about moderate and vigorous exercise. Please exclude walking that you may have done for fitness, recreation or sport, and household chores, gardening or yardwork.

In the last 2 weeks, did [you/proxy name] do any exercise which caused a moderate increase in [your/his/her] heart rate or breathing, that is, moderate exercise?

Yes
No

Question EXER_Q08

How many times did [you/proxy name] do any moderate exercise in the last 2 weeks?

...

Question EXER_Q09

What was the total amount of time [you/proxy name] spent doing moderate exercise in the last 2 weeks?

hours: ...
minutes: ...

Question EXER_Q10

How many days in the last week did [you/proxy name] do any moderate exercise?

...

Question EXER_Q12

What was the total amount of time [you/proxy name] spent doing moderate exercise in the last week?

hours: ...
minutes: ...

Question EXER_Q13

In the last 2 weeks, did [you/proxy name] do any (other) exercise which caused a large increase in [your/his/her] heart rate or breathing, that is, vigorous exercise?

Yes
No

Question EXER_Q14

How many times did [you/proxy name] do any vigorous exercise in the last 2 weeks?

...

Question EXER_Q15

What was the total amount of time [you/proxy name] spent doing vigorous exercise in the last 2 weeks?

hours: ...
minutes: ...

Question EXER_Q17

How many days last week did [you/proxy name] do any vigorous exercise?

...

Question EXER_Q19

What was the total amount of time [you/proxy name] spent doing vigorous exercise in the last week?

hours: ...
minutes: ...

Question EXER_Q19a

Thinking about the types of exercise you have already told me about, that is (walking for fitness, recreation or sport, moderate or vigorous exercise), how many days in the last week did [you/proxy name] exercise?

...

Question EXER_Q19aa

Thinking about all the physical activity [you/proxy name] typically [do/does] now, would you say [you are/he is/she is] more active than this time last year, less active or stayed about the same as this time last year?

More active
Less active
Stayed about the same
Don.t know

Question DIABMED_Q12

(Apart from insulin, medication or being on a special diet.)
In the last 2 weeks, [have you/has (proxy name)] taken any other action to manage [your/his/her] [diabetes/high sugar levels]?

Yes
No

Which ones? More than one response is allowed.

Losing weight
Exercised most days
Taken vitamin/mineral supplements
Taken natural/herbal treatments
Other

Country: Canada

Survey: Canadian Health Measures Survey

Survey Code: CA 04

Year: 2007

Type: Face to Face

Question PAC_Q23

Thinking back over the past 3 months, which of the following best describes your usual daily activities or work habits?

Usually sit during the day and don’t walk around very much
Stand or walk quite a lot during the day but don’t have to carry or lift things very often
Usually lift or carry light loads, or have to climb stairs or hills often
Do heavy work or carry very heavy loads

Question CPA_Q11

Physical activity is any activity that increases your heart rate and makes you get out of breath some of the time. It can be done in sports, school activities, playing with friends, or walking to school.

Some examples of physical activity are running, brisk walking, dancing, swimming, rollerblading, skateboarding, biking, soccer, basketball and, football.

For these next two questions, add up all the time [RESPONDENT NAME] spends in physical activity each day.

Over the past 7 days, on how many days was he physically active for a total of at least 60 minutes per day?

None (zero days)
1 day
2 to 3 days
4 days or more

Question CPA_Q12

Over a typical or usual week, on how many days is he physically active for a total of at least 60 minutes per day?

None (zero days)
1 day
2 to 3 days
4 days or more

Country:	Canada
Survey:	Canadian Community Health Survey (CCHS)
Survey Code:	CA 05
Year:	2007
Type:	Face to Face + Telephone

Question STC_Q1_6

How often do you jog or do other exercise to deal with stress?

Often
Sometimes
Rarely
Never

Country: Switzerland

Survey: Swiss Health Survey

Survey Code: CH 03

Year: 2007

Type: Telephone + self administered

Question 46.02

And how many days per week do you work up a sweat (on average) while working, either in your job or working at home or in the garden?

... days per week (0 to 7 days)
No answer

Question 46.10

The next question is not about activities that make you work up a sweat but about less intensive forms of physical activity:
Think of physical activities in which you get at least a little out of breath, such as brisk walking, hiking, dancing, gardening and various sports:

How many days per week do you engage in these kinds of physical activities?

... days per week (1 to 7 days)
Never
Don't know
No answer

Question46.11

What is the average duration of these physical activities on these days?

... hours and ... minutes per day
Don't know
No answer

Question 46.40

Do you think that you do enough physical exercise for your health?

Yes
No
Don't know
No answer

Country: Germany

Survey: Telephone Health Survey

Survey Code: DE 09

Year: 2007

Type: Telephone

Questions 228-229

The following questions deal with physical activity in day-to-day life, leisure time and occupation. We will ask you about very strenuous activities and moderately strenuous activities. “Very strenuous” are those activities where you have to breathe much harder than usual. “Moderately strenuous” are those activities where you have to breathe a little harder than usual. Finally, we will ask you about the time you spend walking or running in everyday life, leisure time or occupation.

... we will start out with very strenuous activities where you have to breathe much harder than usual, e.g. fast bike riding, heavy construction work or work in the forest, digging soil (e.g. when cultivating a garden) or shovelling snow. How many days out of the last 7 days did you do these types of activities? Please only think of those activities that lasted at least 10 minutes.

...
never
d.k.
n.a.

Questions 230-231-232

... on the days when you are doing these very strenuous activities, how many minutes or hours do you usually spend doing them?

...
d.k.
n.a.

... on the days when you are doing these very strenuous activities, how many minutes or hours do you usually spend doing them?

...
d.k
n.a.

Can you tell me approximately how many minutes or hours total you spent doing this, within the last 7 days?

...
d.k.
n.a.

Question 234

And now on to the moderately strenuous activities where you have to breathe a little harder than usual, e.g. riding a bike at a moderate speed, painting rooms, house cleaning or scrubbing the floor. How many days out of the last 7 days did you do these types of activities? Please think only of those activities that lasted at least 10 minutes.

...
never
d.k.
n.a.

Questions 235-236-237

... the days that you are doing these moderately strenuous activities, how many minutes or hours do you usually spend doing them?

...
d.k.
n.a.

... the days that you are doing these moderately hard activities, how many minutes or hours do you usually spent doing them?

...
d.k.
n.a.

Can you tell me within the last 7 days, how many minutes or hours in total you spent doing this?

...
d.k.
n.a.

Question 239

Now we are going to talk about walking in everyday life, leisure time and occupation. How many days within the last 7 days did you walk for at least 10 consecutive minutes?

...
never
d.k.
n.a.

Questions 240-241-242

... and on the days when you are walking for at least 10 consecutive minutes, how many minutes or hours did you usually spend doing this?

...
d.k.
n.a.

... and on the days when you are walking for at least 10 consecutive minutes, how many minutes or hours did you usually spend doing this?

...
d.k.
n.a.

Can you tell me the total time in minutes or hours you spent doing this within the last 7 days?

...
d.k.
n.a.

Question 244

All together, do you think you do not get enough physical activity, enough physical activity or too much physical activity?

not enough
enough
too much
d.k.
n.a.

Country:	Finland
Survey:	FINRISK 2007
Survey Code:	FI 21
Year:	2007
Type:	Self administered

Question 84

How do you consider your current physical condition?

very good
quite good
fair
quite bad
very bad

Country:	Finland
Survey:	Health Behaviour and Health among the Finnish Adult Population
Survey Code:	FI 22
Year:	2007
Type:	Self administered

Question 77

How would you rate your current (physical) fitness status?

very good
quite good
satisfactory
rather poor
very poor

Question 78

In the past year (12 months), have any of the persons mentioned below advised you to exercise more?

yes
no

physician
other health care provider
family member
other

Country: Finland

Survey: Health Behaviour and Health among the Finnish Elderly Population

Survey Code: FI 23

Year: 2007

Type: Self administered

Question 46

In the past year (12 months), have you changed your diet or other habits for health reasons?

yes

reduced the amount of fat
switched to a different type of fat
increased the consumption of vegetables
reduced the use of sugar
reduced the use of salt
quit smoking
reduced the use of alcohol
exercised more
been on weight reducing diet

Question 54

How often do you perform other physical activities than walking for at least half an hour (e.g. skiing, bicycling, swimming, gymnastics, games, dance)?

daily
4–6 times a week
2–3 times a week
once a week
2–3 times a month
a few times a year or less
I cannot exercise at all due to an illness or injury

Question 55

How would you rate your current (physical) fitness status?

good
rather good
average
rather poor
poor

Question 58

In the past year (12 months), has a health provider (e.g. physician, a public health nurse) advised you to:

yes

reduce the amount of fat in your food
switch to a different type of fat in your food
increase the consumption of vegetables
reduce the use of sugar
reduce the use of salt
quit smoking
reduce the use of alcohol
exercise more
lose weight

Question 78

Exercising is always worthwhile

fully agree
more or less agree
don't know
disagree to some extent
fully disagree

Country: Ireland

Survey: Survey of Lifestyles, Attitudes and Nutrition SLAN

Survey Code: IE 09

Year: 2007

Type: Face to Face

Question B1

We are interested in finding out about the kinds of physical activities that people do as part of their everyday lives.

First, consider a 7 day period (a week). How many times on average do you do the following kinds of exercise for more than 20 minutes during your free time?

Strenuous exercise (heart beats rapidly) (e.g. running, jogging, hurling, camogie, football, soccer, squash, basketball, judo, roller skating, vigorous swimming, vigorous long distance cycling, advanced aerobics)
Moderate exercise (not exhausting) (e.g. fast walking, tennis, badminton, easy swimming, easy cycling, popular and folk dancing, intermediate aerobics, heavy gardening)
Mild exercise (minimal effort) (e.g. yoga, golf, easy walking, fishing from river bank, bowling, beginners aerobics, archery, light gardening)

... times

Question B2

How many days, if any, in an average week do you walk for 30 minutes or more?

... days

Question B3

I am going to ask you about the time you spent being physically active in the last 7 days.
Please answer each question even if you do not consider yourself to be an active person. Think about the activities you do at work, as part of your housework and in the garden, to get from place to place, and in your spare time for recreation, exercise or sport.

Think about all the vigorous activities that you did in the last 7 days. Vigorous physical activities refer to activities that take hard physical effort and make you breathe much harder than normal. Think only about those physical activities that you did for at least 10 minutes at a time.

During the last 7 days, on how many days did you do vigorous physical activities like heavy lifting, digging, aerobics, or fast bicycling?

... days

Question B4

How much time did you usually spend doing vigorous physical activities on one of those days?

... / ... hours and minutes per day
Not sure/don't know

Question B5

Think about all the moderate activities that you did in the last 7 days. Moderate activities refer to activities that take moderate physical effort and make you breathe somewhat harder than normal. Think only about those physical activities that you did for at least 10 minutes at a time.

During the last 7 days, on how many days did you do moderate physical activities like carrying light loads, bicycling at a regular pace, or doubles tennis? [Do not include walking].

... days
None

Question B6

How much time did you usually spend doing moderate physical activities on one of those days?

... /... hours and minutes per day
Not sure/don't know

Question B7a

Think about the time you spent walking in the last 7 days. This includes at work and at home, walking to travel from place to place, and any other walking that you might do solely for recreation, sport, exercise, or leisure.

During the last 7 days, on how many days did you walk at for at least 10 minutes at a time?

... days per week
None

Question B7b

Which of the following best describes your usual walking pace?

A slow pace
A steady average pace
A fairly brisk pace
A fast pace – at least 4 mph

Question B8

How much time did you usually spend walking on one of those days?

... / ... hours and minutes per day
Not sure/don't know

Question B9

Thinking now about regular physical activity, by that I mean: taking part in exercise or sports 2-3 times per week for a minimum of 20 minutes at a time, or more general activities like walking, cycling or dancing 4-5 times per week accumulating to at least 30 minutes per day.

With this in mind, could you look at this card and tell me which statement best describes how physically active you have been over the last six months?

I am not regularly physically active and do not intend to be so in the next six months
I am not regularly physically active but am thinking about starting to do so in the next six months
I do some physical activity but not enough to meet the description of regular physical activity
I am regularly physically active but only began in the last six months
I am regularly physically active and have been so for longer than six months

---

Question B10

What would you say is the main reason why you are not (more) physically active at this time?

Not interested
Interested but not willing to spend the time
No time to do it
No facilities to exercise/be active
Injury/disability/medical condition
Other, specify: ...

Question B13

Are you using any of the following to lose/maintain weight?

Eating fewer calories
Eating less fat

Taking exercise

Yes
No

Country: Iceland

Survey: Health and wellbeing of Icelanders

Survey Code: IS 04

Year: 2007

Type: Self administered

Question 47

You are asked about both the number of days that you exercised in the last 7 days, and for how long you exercised in general the days in question. You are only asked about exercise that lasted 10 minutes or longer.

How many days in the last 7 days did you perform a strenuous exercise for at least 10 minutes that made your breathing become faster than usual, causing you to get winded. (An example of a strenuous exercise is aerobics, lifting a heavy object, ball sports, brisk mountaineering, running, bicycling or swimming fast.)

1 day in the previous week
2 days in the previous week
3 days in the previous week
4 days in the previous week
5 days in the previous week
6 days in the previous week
7 days in the previous week
I didn't do any strenuous exercise last week

Question 48

How much time, in minutes, did you generally spend doing strenuous exercise for the days in the previous week that question 47 asks?

... minutes per day

Question 49

How many days of the last 7 days did you do a moderately strenuous exercise for at least 10 minutes, that made your breathing become a little faster than usual. Do not include hikes or walks. (Examples of a moderately strenuous exercise are home cleaning, playing golf, slow bicycling, slow jogging or slow swimming.)

1 day in the previous week
2 days in the previous week
3 days in the previous week
4 days in the previous week
5 days in the previous week

6 days in the previous week
7 days in the previous week
I didn’t do any moderately strenuous exercise last week

Question 50

How much time, in minutes, did you generally spend doing moderately strenuous exercise for the days in the previous week that question 49 asks?

... minutes a day

Question 51

How many days of the last 7 days did you walk for at least 10 minutes a day? (Example: Walking between two places, walking to work and strolls, hiking is not included.)

1 day in the previous week
2 days in the previous week
3 days in the previous week
4 days in the previous week
5 days in the previous week
6 days in the previous week
7 days in the previous week
I didn’t walk at all in the previous week

Question 52

How much time a day did you generally spend walking for the days in the previous week that question 51 asks?

About 10-15 minutes
About 16-30 minutes
Just over half an hour
About 1 hour
About 2 hours
About 3 hours
About 4 hours or more
Does not apply

Question 55

How frequently, if any, do you generally take advantage of the following outdoor areas?

Every day
Three to six times a week
One to two times a week
One to three times a month
Less than once a month

Never

Outdoor area (such as bicycle or walking trails by a beach or in a vegetation area)
Open natural terrain

Question 57

As a general rule, how would you consider your physical endurance (stamina) in comparison to your peers of the same sex?

A lot better than my peers
Considerably better than my peers
Similar to my peers
Considerably worse than my peers
A lot worse than my peers

Question 58

As a general rule, how would you consider your physical strength in comparison to your peers of the same sex?

A lot better than my peers
Considerably better than my peers
Similar to my peers
Considerably worse than my peers
A lot worse than my peers

Country: Luxembourg

Survey: PSELL/EU-SILC

Survey Code: LU 04

Year: 2007

Type: Face to Face

Question D187

Do you do a physical activity?

Yes, regularly
Yes, sometimes
No, because of health problems
No, for other reasons

Question D188

Do you do a physical activity or an artistic activity out of your home?

Yes, regularly
Yes, sometimes
No, because of health problems
No, for other reasons

Country:	Sweden
Survey:	National Survey of Public Health
Survey Code:	SE 09
Year:	2007
Type:	???

Question 37

How much time in a normal week do you do moderately strenuous activities that make you hot? E.g. walks at a brisk pace, gardening, fairly hard housework, cycling, swimming. This may vary during the year, but try and give some kind of an average. Mark one alternative.

5 hours a week or more
More than 3 hours but less than 5 hours a week
Between 1 and 3 hours a week
At most one hour a week
Not at all

Country: Sweden

Survey: Swedish Level of Living Survey

Survey Code:

Year: 2007

Type: Face to Face

Question 283

Do you pursue any sports, outdoor or exercise activities, e.g. long walks?

How often?

YES, SEVERAL TIMES A WEEK
YES, ABOUT ONCE A WEEK
YES, 1–3 TIMES A MONTH
YES, BUT LESS OFTEN
NO, NEVER

Country: Slovakia

Survey: European Health Interview Survey

Survey Code: SK 04

Year: 2007

Type: Face to Face

Question PE.1

Now I will ask you questions about the time you actively spent carrying out physical activities in the last 7 days. Please answer each question, even when you do not consider yourself to be a physically active wo/man. Think of the activities carried out at work, activities that are part of your housework or work in the garden, the activities undertaken in free time, your sporting activities and how you transport yourself from place to place. Remember all your energy-intensive activities, in other words, when you had to invest a considerable physical effort and that you performed during the last 7 days. During the intensive physical activity (done at an accelerated pace) you breathe faster, than normal. These activities may include, for example, lifting heavy loads, spading, aerobics or fast cycling. Consider only those activities that you have performed continuously at least 10 minutes.

How many days have you performed intense physical activity during the last 7 days?

... Number of days per week
I do not know
Refused to answer

INTERVIEWER CLARIFIES: THINK ONLY OF PHYSICAL ACTIVITY YOU HAVE BEEN DOING CONTINUOUSLY FOR AT LEAST 10 MINUTES.

Question PE.2

How much time did you spend carrying out vigorous physical activity during the last 7 days? „ESTIMATION IS ACCEPTABLE“.

... hours ... minutes
I do not know
Refused to answer

Question PE.3

And now try to think back all your activities, which needed to be done with only mild physical effort, and you have done them over the last 7 days. We mean activities undertaken with only modest effort or slow tempo cause that you breathe just a little faster than normal and may include carrying light loads such as cycling in recreational tempo. Do not take into account walking but consider only those activities that were carried out continuously for at least 10 minutes.

How many days have you performed moderately intense physical activity during the last 7 days?

... Number of days per week
I do not know
Refused to answer

Question PE.4

How much time have you spent by performing moderately intense physical activity during the last 7 days? „ ESTIMATION IS ACCEPTABLE “.

... hours ... minutes
I do not know
Refused to answer

Question PE.5

And now try to think back the time you spent walking. Consider walking at work, walking in the house (apartment) and walking when you needed to get somewhere or any other walk, you carried out only as a recreational sport, exercise or relaxation.

How many days did you take a walk, which lasted continuously for at least 10 minutes during the last 7 days?

... Number of days per week
I do not know
Refused to answer

Question PE.6

INTERVIEWER CLARIFIES: THINK ONLY OF SUCH A WALK YOU HAVE BEEN DOING CONTINUOUSLY FOR AT LEAST 10 MINUTES.

How much time did you spend walking in the last 7 days? „ ESTIMATION IS ACCEPTABLE “.

... hours ... minutes
I do not know
Refused to answer

Country: United Kingdom

Survey: Health Survey for England

Survey Code: UK 35

Year: 2007

Type: Face to Face + self administered

Question Wlk5Ch

Now I'd like to ask you about some of the things you have/(name of child) has done in the last week. By last week I mean last (day one week ago) up to yesterday. In the last week, have you/has s/he done a continuous walk that lasted at least 5 minutes not counting things done as part of school lessons?

Yes
No

Question DaysWlk

On how many days in the last week did you/(name of child) do a continuous walk that lasted at least 5 minutes not counting things done as part of school lessons?

One day
Two days
Three days
Four days
Five days
Six days
Every day

Question DayWlkT

On each day that you/(name of child) did a walk like this for at least 5 minutes, how long did you/s/he spend walking altogether?

What we want recorded is the average time spent walking per weekday.
If the respondent walked for over 5 minutes on more than one day in the last week, take an average of the time spent per day

5 minutes, less than 15 minutes
15 minutes, less than 30 minutes
30 minutes, less than 1 hour
1 hour, less than 1 1/2 hours
1 1/2 hours, less than 2 hours
2 hours, less than 2 1/2 hours
2 1/2 hours, less than 3 hours
3 hours, less than 3 1/2 hours
3 1/2 hours, less than 4 hours
4 hours or more (please specify how long)

Question WlkHrs

How long did you/(name of child) spend walking on each day?
RECORD HOURS SPENT BELOW

...

RECORD HERE MINUTES SPENT WALKING.

...

Question ChPace

Which of the following describes your usual walking pace ...

... a slow pace,
... a steady average pace,
... a fairly brisk pace,
... or, a fast pace - at least 4 mph?
... None of these

Question Q41

How many days a week do you think people of your age should do physical activity? Include all moderate physical activity, including physical activity as part of a job.
By week we mean the whole week including weekends.

Please write in NUMBER ... Days
OR TICK I don't know

Question Q42

On each of the days someone of your age does moderate physical activity, how many minutes a day should they do it for it to be good for their health?

Please write in NUMBER ... Minutes per day
OR TICK I don't know

Question Q43

Here are some statements about physical activity and exercise.
Please indicate how strongly you agree or disagree with the statements.

a) You can get enough physical activity in your daily life without doing sport or exercise such as jogging or going to the gym
b) Physical activity is good for your health even if it is only for 10 minutes at a time
c) Physical activity is good for your health even if it is moderate, such as walking briskly, gardening (for example digging) and housework (for example vacuuming)
d) Physical activity is better for your health if you keep it up for at least 30 minutes at a time

e) Physical activity is better for your health if it gets you out of breath

Strongly agree
Agree
Neither agree or disagree
Disagree
Strongly disagree
Can't choose

Question Q44

There are many reasons for taking part in physical activity, exercise or sport. Which, if any, on the following list apply to you?
Tick ALL that apply

I don't participate in any physical activity: Go to Q 4 5

To release tension or relax
To be out of doors
To maintain good health
To socialise with other people
To lose or control weight
I have a physically demanding job
To have fun or adventure or excitement
To get or feel fit
To gain a sense of achievement
To enjoy the competition
To work harder or concentrate better
I have to walk or cycle to get around
Something else

Question Q45

Compared to other people of your own age would you describe yourself as…
Tick one box

Very physically active
Fairly physically active
Not very physically active
Not at all physically active -> Go to Q48

Question Q46

Which of the following activities do you think contribute most to keeping you fit?

Walking or cycling to get around
Climbing stairs instead of taking a lift or escalator
Keeping active at home with housework, gardening or childcare
Having a physically active job
Going for walks or going hiking
Going out for cycle rides or going mountain biking

Going to the gym
Going jogging, running or swimming
Playing golf
Taking part in sport
Doing keep fit, exercise or dance
Something else

Question Q47

Do you think you do enough physical activity, exercise, or sport to keep you as fit as you would like?

Yes
No

Question Q48

Would you like to do more exercise or physical activity than you do at the moment?

Yes
No -> Go to Q51
At the moment I am unable to

Question Q49

Which of the following statements best describes you?
Tick one box

I'm unlikely ever to do more physical activity, exercise or sport -> Go to Q51
I expect to do more physical activity, exercise or sport in the next year
I expect to do more physical activity, exercise or sport but not in the next year
I don't know

Question Q50

Which types of physical activity, exercise or sport do you expect to take more of in the future?
Tick all that apply

Walking or cycling to get around
Climbing stairs instead of taking a lift or escalator
Keeping active at home with housework, gardening or childcare
Having a physically active job Going for walks or going hiking
Going out for cycle rides or going mountain biking Going to the gym Going jogging, running or swimming
Playing golf
Taking part in sport
Doing keep fit, exercise or dance Something else

Question Q51

What stops you from doing more physical activity, exercise or sport than you do now?
Tick ALL that apply

I don't need to do more -> Go to the end
My work commitments
I don't have enough leisure time
Caring for children or older people
I have no one to exercise with
I don't have enough money
There are no suitable places to do it in my area
I haven't got the right clothes or equipment
Poor health or physical limitations
I have injuries which prevent me
None of these

Question Q52

Many other factors can prevent people from doing more physical activity, exercise or sport.
From the following list which, if any, apply to you?
Tick ALL that apply

I'm not the sporty type
I'm too shy or embarrassed
I'm worried about injury
I'm too old
I prefer to do other things
I think exercise is a waste of time
I'm too overweight
I am not motivated to do more
I don't enjoy physical activity
Something else
None of these
Don't know

Question Q53

What would encourage you to do more physical activity, exercise or sport?
Tick ALL that apply

I don't need to do more
Advice from a doctor or a nurse
Advice from a family member
My own ill health
Family member's ill health
Increased income
More leisure time
Self-motivation
Having someone to do it with
Having a physical activity
I am capable of
Clearer advice from the government
Something else
None of these

Country: Belgium

Survey: Health Interview Survey

Survey Code: BE 06

Year: 2008

Type: Self administered

Question PE01-PA01

Think of the physical activities you engage in at work, in the house or garden, and physical activities in connection with getting from place to place and during your free time, such as leisure activities, exercise sessions and sport.

Intense physical activities

Intense physical activities are those that require a major physical effort and cause you to breathe much more quickly and deeply than usually.

In the past 7 days, on how many days did you engage in intense physical activities such as lifting heavy objects, digging, aerobics, jogging or football?

... days a week

Question PA02

On one of these days, for how long did you engage in this kind of intense physical activity?

... hours a day
... minutes a day
I don't know

Question PE03-PA03

Moderate physical activity

When we speak of moderate physical activity we are thinking of activities that require a moderate physical effort and that make you breathe slightly more quickly than usually.

In the past 7 days, on how many days did you engage in moderate physical activity such as lifting not too heavy objects, cycling at a moderate rate, playing doubles tennis, swimming, …?
!!! Do not include walking!!!

... days a week

Question PA04

On one of these days, for how long did you engage in this kind of moderate physical activity?

... hours a day
... minutes a day
I don't know

Question PE05-PA05

Walking

Think now of the time you spent walking, whether to home or back home, walking from one place to another, and any other kind of walking you may have one for relaxation, sport, exercise or leisure.

During the past 7 days, on how many days did you walk for more than 10 minutes continuously?

... days a week

Question PA06

On one of these days, for how long in total did you walk?

... hours a day
... minutes a day
I don't know

Question PA07

In general, how fast did you walk? You walked …

Quickly, causing you to breathe much more quickly than usually
At a moderate pace, causing you to breathe a little more quickly than usually
Slowly, which did not cause you to change your breathing
I don't know

Country: Bulgaria

Survey: EHIS 2008

Survey Code: BG 04

Year: 2008

Type: Face to Face

Question PE.1

I would like to ask you about the time you have spent being physically active in the last seven days. Please answer each question even if you don't think you are a physically active person. Think about activities you do at work; as part of your housework and gardening; moving from place to place; in your spare time for leisure, exercise or sport.
Please think about the vigorous activities you did during the last 7 days. Vigorous physical activities take hard physical effort and make you breathe much harder than normal. They may include things like heavy lifting, digging, aerobics or fast cycling. Please think about only tjose activities you did for at least 10 minutes at a time.

During the last seven days, on how many days did you do any vigorous physical activities? Do not include walking.

... days per week
Don't know
Refusal

Question PE.2

About how much time, in total, did you spend over the last seven days doing vigorous physical activity? An estimate is acceptable

... hours ... min
Don't know
Refusal

Question PE.3

I'd like you to think about activities requiring moderate physical effort. Moderate physical activities make you breathe somewhat harder than normal and may include light loads such as cycling at a regular pace, or playing doubles tennis. Do not include walking. Please think about only those physical activities that you did for at least 10 minutes at a time.

During the last 7 days, on how many days did you do any moderate physical activities? Do not include walking.

... days per week
Don't know
Refusal

Question PE.4

About how much time, in total, did you spend over those seven days doing moderate physical activity? An estimate is acceptable

... hours ... min
Don't know
Refusal

Question PE.5

Think about the time you spent walking in the last 7 days, for at least 10 minutes at a time. This includes walking wile at work or at home; walking from place to place; and any other walking that you might do solely for leisure, sport, exercise or in your spare time.

During the last 7 days, on how many days did you walk for at least 10 minutes at a time?

... days per week
Don't know
Refusal

Question PE.6

About how much time did you spend walking over the last 7 days?
An estimate is acceptable

... hours ... min
Don't know
Refusal

Country:	Cyprus
Survey:	EHIS 2008
Survey Code:	CY 03
Year:	2008
Type:	Face to Face

Question PE.1

Questions PE.1-PE.6, are about the time you spend for sport/physical activities in the past 7 days. Please answer every question even if you do not consider your self an active person.
Please include all kind of activities, such as housework and gardening, as well as activities you do at work or as an exercise or sport.

Intensive activities need great physical effort and harder breathing than normal. They include heavy lifting, construction works, agricultural works, aerobics, fast bicycling etc.

Please think only those activities that you did for at least 10 minutes during the past 7 days.

During the past 7 days, how many days did you do intensive activities for at least 10 minutes per time?

No of days: ...
Do not know/not sure/do not remember
I refuse to answer

Question PE.2

During the past 7 days, how much time did you spend in total doing intensive physical activities?
Interviewer, inform the interviewee that an estimate is acceptable only if necessary

Hours/Minutes ... : ...
Do not know/not sure/do not remember
I refuse to answer

Question PE.3

Moderate activities need harder breathing than normal. They include moderate lifting, tennis (double game), normal speed cycling etc. Walking is not included.

Please think only those activities that you did for at least 10 minutes during the past 7 days.

During the past 7 days, how many days did you do moderate activities for at least 10 minutes per time?

No of days ...
Do not know/not sure/do not remember
I refuse to answer

Question PE.4

During the past 7 days, how much time did you spend in total doing moderate physical activities? Interviewer, inform the interviewee that an estimate is acceptable only if necessary.

Hours/Minutes ... : ...
Do not know/not sure/do not remember
I refuse to answer

Question PE.6

During the past 7 days, how much time did you spend walking? Interviewer, inform the interviewee that an estimate is acceptable only if necessary.

Hours/Minutes ... : ...
Do not know/not sure/do not remember
I refuse to answer

Country: Czech Republic

Survey: EHIS CR 2008

Survey Code: CZ 04

Year: 2008

Type: Face to Face

Question PE.1

Now I would like to ask you about time that you spent doing physical activity in the last seven days. Please answer all questions, even if you do not consider yourself physically active person. Consider all your activities that you do at work, at home or in the garden, moving from place to place, free time activities or sports activities.
Consider all intense, physically demanding physical activities that require hard physical straining you have done within the last 7 days. Hard physical activity markedly increases frequency of breathing compared to normal. It may include, for instance, lifting heavy weights, excavation work, aerobic or fast bicycle ride. Please, consider only those physical activities that you were performing for at least 10 minutes at a time.

In how many days within the last 7 days did you perform any hard physical activity?

... days a week
does not know
refused

Question PE.2

Instruction for the interviewer: Read: “Consider only those activities that the respondent performed for at least 10 minutes at a time.“

Please, can you state how much time have you spent in these days doing hard physical activity?
If necessary, you may tell the respondent: “An estimate is acceptable.“

... hours ... minutes
does not know
refused

Question PE.3

Consider all medium-intensity physical activities that you have been doing within the last 7 days. Medium-intensity physical activities increases breathing frequency compared to normal and may include carrying light weight loads, riding a bike at regular pace or playing doubles in tennis. Do not include walking. Please consider only those physical activities that you were performing for at least 10

minutes at a time.

In how many days within the last 7 days did you perform any medium-intensity physical activities?

... days a week
does not know
refused

Question PE.4

Instruction for the interviewer: Read: “Consider only those activities that you performed for at least 10 minutes at a time.“

Please, can you state how much time have you spent in these days doing medium-intensity physical activities?
If necessary, you may tell the respondent: “An estimate is acceptable.“

... hours ... minutes
does not know
refused

Question PE.5

Now, consider all the time spent by walking in the last 7 days. This means walking at work or at home, walking while moving from place to place and any other walking that you do during recreation, sports, tourism or walks in your free time.

In how many days (within the last 7 days) did you spent at least 10 minutes at a time walking?

... days a week
does not know
refused

Question PE.6

Instruction for the interviewer: Read: “Consider only walking you performed for at least 10 minutes at a time.“

Please, could you state how much time did you spend in these days walking?
If necessary, you may tell the respondent: “An estimate is acceptable.“

... hours ... minutes
does not know
refused

Country:	Latvia
Survey:	EHIS 2008
Survey Code:	LV 06
Year:	2008
Type:	Face to Face

Question FS_01

Now, I will ask you questions concerning the time you have spent on physical activities over the last 7 days.
Recall the intensive physical activities you have had over the last 7 days (heavy physical effort, picking up heavy things, digging, aerobics etc.). Please take into account only those intensive physical activities that were continuous for at least 10 minutes.

Questions on physical activities

How often (how many days) have you had any intensive physical activities over the last 7 days?

... days per week
None
I do not know
Refuses to respond

Question FS_02

How much time have you spent on intensive physical activities over the last 7 days?

... hours ... minutes
I do not know
Refuses to respond

Question FS_03

Now, think of moderate physical activities (e.g. carrying light weights, cycling and other activities requiring moderate physical effort). Please take into account only those activities that were continuous for at least 10 minutes.

How often (how many days) did you have any moderate physical activities over the last 7 days?

... days per week
None
I do not know
Refuses to respond

Question FS_04

During the past 7 days, how much time did you spend in total doing moderate physical activities? Interviewer, inform the interviewee that an estimate is acceptable only if necessary.

Hours/Minutes ... : ...
Do not know/not sure/do not remember
I refuse to answer

<u>Question FS_05</u>

Now, think of the time you have spent on walking over the last 7 days. This includes moving from one place to another at work or at home, and any kind of walk performed for the purposes of training, resting or sports. Please take into account only those activities that were continuous for at least 10 minutes.

How often (how many days) have you walked for at least 10 continuous minutes over the last 7 days?

... days per week
None
I do not know
Refuses to respond

<u>Question FS_06</u>

How much time have you spent on walking over the last 7 days?

... hours ... minutes
I do not know
Refuses to respond

Country:	Malta
Survey:	EHIS 2008
Survey Code:	MT 02
Year:	2008
Type:	Face to Face

Question PE.1

Now I'd like to ask you about the time spent doing physical activity in these last 7 days.

Kindly answer each question even if you don't think you're that active. Think about the activity you do at work, as part of housework or hobby, to get from one place to another, and during your free recreational time, exercise or sport.

Think about the most intense activities, which require a great physical effort, which you've done in these last 7 days. Intense activities require you to take more deep breaths, and they can include carrying heavy weight, digging, aerobics, or riding a bike with a certain velocity. Think only of those physical activities on which you spent at least 10 minutes every time while doing them.

In these last 7 days, how many days did you do very intense physical activities?

... Days in a week
Don't know
I'd rather not tell you

Question PE.2

THINK ONLY OF THOSE PHYSICAL ACTIVITIES ON WHICH YOU SPEND AT LEAST 10 MINUTES EVERY TIME WHILE DOING THEM.

In these last 7 days, how much time in all did you spend in very intense physical activities?

... hours ... minutes
Don't know
I'd rather not tell you

Question PE.3

Now think about the activities of moderate intensity which you have done in these last 7 days.

Physical activities of moderate intensity enable you to take deeper breaths than usual, and they can include carrying light weight, riding a bike with a normal velocity, or tennis in doubles. Don't include walking. Just like before, only think

about those physical activities on which you spend at least 10 minutes every time.

In these last 7 days, how many days did you spend on physical activities of moderate intensity?

... Days a week
Don’t know
I’d rather not tell you

Question PE.4

THINK ONLY OF THOSE PHYSICAL ACTIVITIES ON WHICH YOU SPEND AT LEAST 10 MINUTES EVERY TIME WHILE DOING THEM.

In these last 7 days, how many days did you spent doing physical activities of moderate intensity?

... hours ... minutes
Don’t know
I’d rather not tell you

Question PE.5

Now think of the time you’ve spent walking in these last 7 days.

This includes walking either from work or from home, walking from one place to another, or any other type of walking that you do for your recreation, sport and exercise or in your free time.

In these last 7 days, how many days did you walk at least 10 minutes every time?

... Days in a week
Don’t know
I’d rather not tell you

Country: Romania

Survey: Health Interview Survey

Survey Code: RO 02

Year: 2008

Type: Face to Face + self administered

Question 114

Physical activity

Now I would like to ask you about the time you spent on physical activity during the last 7 days. Please answer each question even if you do not see yourself as an active person.
Think of any physical activity, such as: housekeeping or gardening, activities at work, physical exercises, walks or sports.
I will ask separate questions about intense activities, moderate activities and walking. Let us talk first about intense physical activities.
Intense physical activities involve high physical effort and heavier breathing than usual. They can include activities such as: lifting heavy objects, digging, wood cutting, working in constructions, going up the stairs for work, gymnastics or bicycle riding at high speed.
Think only of those physical activities that you performed for at least 10 minutes.

During the last 7 days, on how many days did you have intense physical activities?

total days with vigorous physical activities: ... days week
I don't know/I'm not sure
Refusal

Question 115

On the overall, during these last 7 days, how much time did you spend on intense physical activities?

total time of intense physical activities/week:
... hours ... minutes/week
I don't know/I'm not sure
Refusal

Question 116

Now I would like to ask you about the time you spend on moderate physical activities.
A moderate physical activity is an activity that involves moderate physical effort and that makes you breathe a bit heavier then usual and may include activities such as: lifting of light weights, sweeping the floor, washing windows, riding a

bicycle (at moderate speed), tennis or swimming. Do not include strolls.
Now think of only those moderate physical activities that you performed for at least 10 minutes.

During the last 7 days, on how many days did you have moderate physical activities?

total days with moderate physical activities: ... days/week
I don't know/I'm not sure
Refusal

Question 117

On the overall, during these last 7 days, how much time did you spend on moderate physical activities?

total time of moderate physical activities/week:
... hours ... minutes/week
I don't know/I'm not sure
Refusal

Question 118

Let us talk about the time spent on walking or strolling.
Strolling is a separate activity and includes walking to or from work/school, walking from one place to another or any other strolls you take as physical exercises, sports or only for recreation.
Now think only of those strolls you took for at least 10 minutes.

During the last 7 days, on how many days did you have strolls or walks, for at least 10 minutes?

total days with strolls: ... days/week
I don't know/I'm not sure
Refusal

Question 119

On the overall, during these last 7 days, how much time did you spend on walking or strolling?

total strolling time/day: ... hours ... minutes/day
I don't know/I'm not sure
Refusal

Country: Turkey

Survey: Health Interview Survey 2008

Survey Code: TR 06

Year: 2008

Type: Face to Face

Question PE.1

How many times did you do vigorous bodily activity in the past 7 days?

In one week ... day(s)
Never
I do not know
I do not wish to respond

Question PE.2

How much time did you spend on vigorous bodily activity in the past 7 days?

... hours ... minutes
I do not know
I do not wish to respond

Question PE.3

Think about activities requiring moderate physical force in the past 7 days. In moderate activities, a person breathes slightly harder than normal, and such activities include carrying a light weight, riding a bicycle at normal speed or playing a tennis match. Do not include walking in this group. Think about when you do these physical activities for at least 10 minutes on just one occasion.

How many times did you do moderate bodily activity in the past 7 days?

In one week: ... day(s)
Never
I do not know
I do not wish to respond

Question PE.4

How much time did you spend on moderate physical force in the past 7 days?

hours ... minutes ...
I do not know
I do not wish to respond

Question PE.5

How many times did you do vigorous bodily activity in the past 7 days?

In one week ... day(s)
Never
I do not know
I do not wish to respond

Question PE.6

How much time did you spend walking in the past 7 days?

... hours ... minutes
I do not know
I do not wish to respond

Country:	United Kingdom
Survey:	Health Survey for England
Survey Code:	UK 36
Year:	2008
Type:	Face to Face

Question Wlk5Ch

Now I'd like to ask you about some of the things you have/(name of child) has done in the last week. By last week I mean last (day one week ago) up to yesterday. In the last week, have you/has s/he done a continuous walk that lasted at least 5 minutes not counting things done as part of school lessons?

Yes
No

Question DaysWlk

On how many days in the last week did you/(name of child) do a continuous walk that lasted at least 5 minutes not counting things done as part of school lessons?

One day
Two days
Three days
Four days
Five days
Six days
Every day

Question DayWlkT

On each day that you/(name of child) did a walk like this for at least 5 minutes, how long did you/s/he spend walking altogether?

What we want recorded is the average time spent walking per weekday.
If the respondent walked for over 5 minutes on more than one day in the last week, take an average of the time spent per day

5 minutes, less than 15 minutes
15 minutes, less than 30 minutes
30 minutes, less than 1 hour
1 hour, less than 1 1/2 hours
1 1/2 hours, less than 2 hours
2 hours, less than 2 1/2 hours
2 1/2 hours, less than 3 hours
3 hours, less than 3 1/2 hours
3 1/2 hours, less than 4 hours
4 hours or more (please specify how long)

Question WlkHrs

How long did you/(name of child) spend walking on each day?
RECORD HOURS SPENT BELOW

...

RECORD HERE MINUTES SPENT WALKING.

...

Question ChPace

Which of the following describes your usual walking pace ...

... a slow pace,
... a steady average pace,
... a fairly brisk pace,
... or, a fast pace - at least 4 mph?
... None of these
