# Supplementary material for: Development of the European Health Interview Survey - Physical Activity Questionnaire (EHIS-PAQ) to monitor physical activity in the European Union
Source: Arch Public Health. 2015 Dec 2;73:59. doi: 10.1186/s13690-015-0110-z (PMC4667448; doi:10.1186/s13690-015-0110-z)
Supplement: Additional file 2: — Contains the descriptive results of the pilot survey for the work-related PA outcome (Table S1), the transport-related PA outcomes (Table S2), the leisure - time PA outcomes (Table S3) and the health-enhancing PA outcomes (Table S4). Adobe Acrobat Reader is required to access those files. (PDF 238 kb) [file 13690_2015_110_MOESM2_ESM.pdf]

Supplemental File 2

Table 1. Proportion of respondents work-related physical activity according to selected characteristics, workforce population – pooled analysis Belgium, Germany, Estonia , ImpEHIS II 2011

Characteristic	No. in sample	Mostly sitting or standing		Mostly walking or tasks of light to moderate physical effort		Mostly heavy labour or physically demanding work	
		%	N	%	N	%	N
**Total**	97	59.8	58	26.8	26	13.4	13
**Age group (yrs)**							
15-29	10	30.0	3	30.0	3	40.0	4
30-39	24	66.7	16	29.2	7	4.2	1
40-49	28	60.7	17	32.1	9	7.1	2
50-59	25	60.0	15	20.0	5	20.0	5
≥60	10	70.0	7	20.0	2	10.0	1
**Sex**							
Men	52	59.6	31	19.2	10	21.2	11
Women	45	60.0	27	35.6	16	4.4	2
**Education**							
Primary / lower secondary	5	40.0	2	20.0	1	40.0	2
Upper secondary	35	37.1	13	37.1	13	25.7	9
Post secondary or tertiary	57	75.4	43	21.1	12	3.5	2
**Marital status**							
Married	57	57.9	33	33.3	19	8.8	5
Previously married	14	71.4	10	14.3	2	14.3	2
Never married	26	57.7	15	19.2	5	23.1	6
**Perceived Health**							
(Very) good	73	65.8	48	21.9	16	12.3	9
(Very) poor to fair	23	39.1	9	43.5	10	17.4	4
**Country**							
Belgium	24	75.0	18	12.5	3	12.5	3
Germany (PAPI)	29	72.4	21	27.6	8	0	0
Germany (CATI)	19	52.6	10	26.3	5	21.5	4
Estonia	25	36.0	9	40.0	10	24.0	6

Table 2. Means of transport-related walking, bicycling and overall active transportation according to selected characteristics – pooled analysis Belgium, Germany, Estonia

Characteristic	Walking minutes per week		Bicycling minutes per week		Active transportation MET* minutes per week	
	m	N	m	N	m	N
**Total**	251.8	164	51.8	167	1145.0	164
**Age group (yrs)**						
15-29	342.0	33	49.4	33	1424.9	33
30-39	221.5	30	60.9	33	1120.0	30
40-49	245.9	35	82.9	35	1308.5	35
50-59	150.6	35	26.9	35	658.0	35
≥60	306.1	31	37.7	31	1236.7	31
**Sex**						
Men	255.1	82	59.9	83	1205.4	82
Women	248.5	82	43.8	84	1084.7	82
**Education**						
Primary / Lower secondary	261.9	21	1.0	21	870.0	21
Upper secondary	312.0	55	51.7	57	1343.8	55
Post secondary or tertiary	211.8	88	63.9	89	1086.4	88
**Marital status**						
Married	210.6	84	49.6	86	996.4	84
Previously married	250.2	21	12.7	22	900.1	21
Never married	311.0	59	69.6	59	1443.8	59
**Employment status**						
Employed	214.1	100	51.4	102	1019.6	100
Student, trainee	330.3	19	79.2	19	1565.1	19
Retired	228.5	26	38.8	26	987.0	26
Others	418.6	18	35.3	19	1589.8	18
**Perceived Health**						
(Very) good	257.3	119	56.9	122	1195.8	119
(Very) poor to fair	235.5	44	38.9	44	1010.2	44
**Country**						
Belgium	204.7	48	27.9	50	847.3	48
Germany (PAPI)	259.2	42	97.6	42	1441.0	42
Germany (CATI)	258.6	40	43.0	40	1111.5	40
Estonia	301.2	34	41.0	35	1239.2	34

* Overall active transportation, assessed on the basis of self-reported walking and bicycling for transportation, in metabolic equivalents (MET) kcal/kg, 1 MET = persons caloric consumption at complete rest.

Table 3. Means of leisure-time, health-enhancing and muscle-strengthening physical activity according to selected characteristics – pooled analysis Belgium, Germany, Estonia

	Sports, fitness and recreational (leisure) PA minutes per week		HEPA (leisure PA + cycling) minutes per week		Muscle-strengthening activity in times per week	
**Characteristic**	**m**	**N**	**m**	**N**	**m**	**N**
**Total**	156.1	166	208.5	165	0.7	165
**Age group (yrs)**						
15-29	217.2	32	268.1	32	1.2	32
30-39	138.6	32	201.4	32	0.5	33
40-49	157.7	35	240.6	35	0.7	35
50-59	119.0	35	145.9	35	0.6	35
≥60	151.0	31	188.7	31	0.4	31
**Sex**						
Men	180.8	83	240.7	83	0.7	83
Women	131.0	82	175.9	82	0.7	83
**Education**						
Primary / Lower secondary	135.5	20	136.5	20	0.5	20
Upper secondary	132.4	57	184.0	57	0.6	57
Post-secondary or tertiary	176.1	88	240.7	88	0.8	89
**Marital status**						
Married	131.4	85	181.6	85	0.4	86
Previously married	137.7	22	150.5	22	0.3	22
Never married	199.1	58	269.9	58	1.2	58
**Employment status**						
Employed	147.9	102	199.3	102	0.7	102
Student, trainee	240.0	18	323.6	18	0.9	18
Retired	161.2	25	201.6	25	0.4	26
Others	121.8	19	157.1	19	0.6	19
**Perceived health**						
(Very) good	174.0	121	231.4	121	0.7	121
(Very) poor to fair	109.1	43	148.8	43	0.8	44
**Country**						
Belgium	104.4	50	132.3	50	0.2	50
Germany (PAPI)	188.2	42	285.8	42	1.1	42
Germany (CATI)	207.7	39	251.8	39	1.2	40
Estonia	133.1	34	175.3	34	0.3	34

Table 4. Proportion of respondents meeting the health-enhancing physical activity (HEPA) guidelines, by type of physical activity and selected characteristics – pooled analysis Belgium, Germany, Estonia

	Aerobic PA compliance (HEPA ≥150 min/week)		Muscle-strengthening PA compliance (MSPA ≥ 2 times/week)		Overall PA recommendation compliance (meeting both guidelines, HEPA and MSPA)	
**Characteristic**	**%**	**N**	**%**	**N**	**%**	**N**
**Total**	48.5	165	19.4	165	16.4	165
**Age group (yrs)**						
15-29	53.1	17	37.5	12	31.3	10
30-39	53.1	17	15.6	5	15.6	5
40-49	48.6	17	14.3	5	14.3	5
50-59	48.6	17	17.1	6	14.3	5
≥60	38.7	12	12.9	4	6.5	2
**Sex**						
Men	54.2	45	18.1	15	18.1	15
Women	42.7	35	20.7	17	14.6	12
**Education**						
Primary / Lower secondary	25.0	5	15.0	3	15.0	3
Upper secondary	45.6	26	19.3	11	17.5	10
Post secondary or tertiary	55.7	49	20.5	18	15.9	14
**Marital status**						
Married	45.9	39	12.9	11	9.4	8
Previously married	50.0	11	9.1	2	9.1	2
Never married	51.7	30	32.8	19	29.3	17
**Employment status**						
Employed	47.1	48	18.6	19	15.7	16
Student, trainee	61.1	11	33.3	6	27.8	5
Retired	40.0	10	16.0	4	12.0	3
Others	56.7	17	20.0	6	16.7	5
**Perceived health**						
(Very) good	52.9	64	18.2	22	15.7	19
(Very) poor to fair	37.2	16	23.2	10	18.6	8
**Country**						
Belgium	34.0	17	8.0	4	8.0	4
Germany (PAPI)	61.9	26	26.2	11	23.9	10
Germany (CATI)	59.0	23	35.9	14	25.6	10
Estonia	41.2	14	8.8	3	8.8	3
